# High-Throughput Single-Entity Electrochemistry with Microelectrode Arrays

**DOI:** 10.1021/acs.analchem.4c01092

**Published:** 2024-05-23

**Authors:** Sasha E. Alden, Lingjie Zhang, Yunong Wang, Nickolay V. Lavrik, Scott N. Thorgaard, Lane A. Baker

**Affiliations:** † Department of Chemistry, Texas A&M University, College Station, Texas 77843, United States; ‡ Center for Nanophase Materials Sciences, Oak Ridge National Laboratory, Oakridge, Tennessee 37830, United States; § Department of Chemistry, Grand Valley State University, Allendale, Michigan 49401, United States

## Abstract

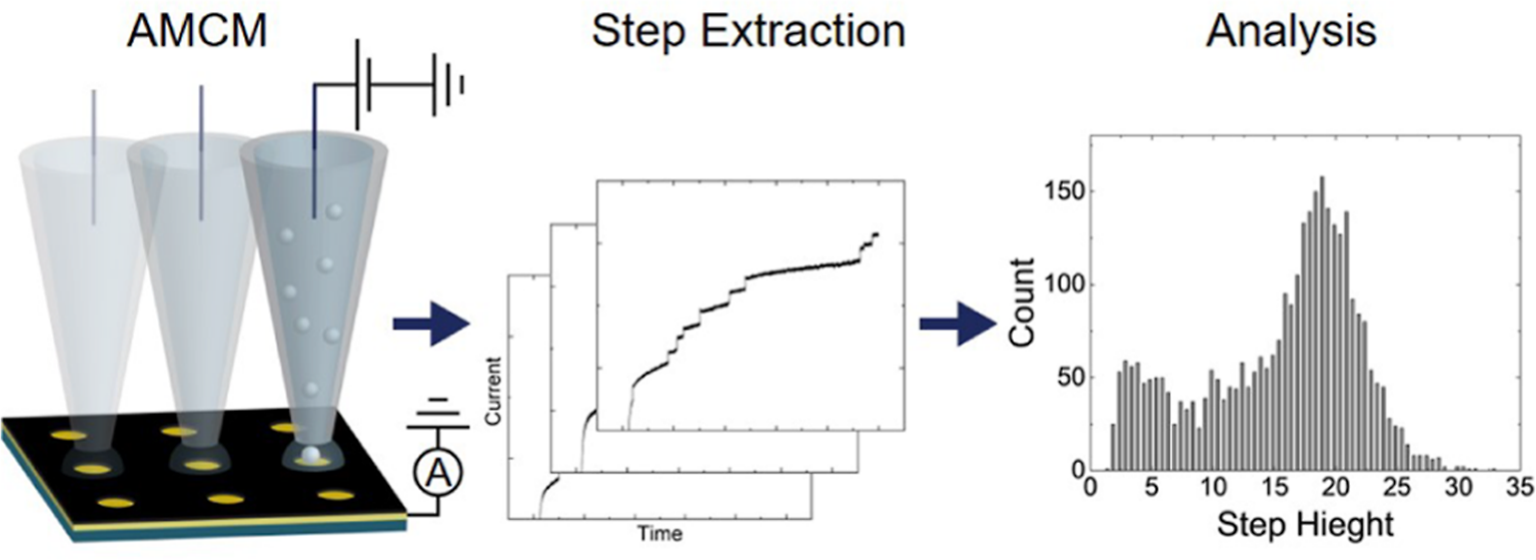

We describe micro- and nanoelectrode array analysis with an automated version of the array microcell method (AMCM). Characterization of hundreds of electrodes, with diameters ranging from 100 nm to 2 μm, was carried out by using AMCM voltammetry and chronoamperometry. The influence of solvent evaporation on mass transport in the AMCM pipette and the resultant electrochemical response were investigated, with experimental results supported by finite element method simulations. We also describe the application of AMCM to high-throughput single-entity electrochemistry in measurements of stochastic nanoparticle impacts. Collision experiments recorded 3270 single-particle events from 671 electrodes. Data collection parameters were optimized to enable these experiments to be completed in a few hours, and the collision transient sizes were analyzed with a U-Net deep learning model. Elucidation of collision transient sizes by histograms from these experiments was enhanced due to the large sample size possible with AMCM.

## Introduction

Single-entity electrochemistry (SEE) entails the one-at-a-time characterization of individual entities of interest, such as cells, (nano)particles, molecules, etc., with a discrete electrochemical response assignable to each entity measured.^1−4^ SEE measurements provide a means to capture the complexity and variability of a population sampled. Among SEE experiments, particle collision electrochemistry has received much attention since the 2004 report by Lemay and co-workers.^5^ Collision experiment studies include fundamental electrochemistry,^6−10^ sensing,^11,12^ and catalysis.^6,13−15^ Reports of entities of just a few nanometers in size^16,17^ with transient current magnitudes as low as ∼200 fA^13^ have also been described. Collision studies can be categorized by the event type such as blocking, catalytic, or particle coulometry^18^ and are typically performed using individual microelectrodes to study a relatively small number of collisions. Only a few reports have been published containing >1000 events for a single system.^19^ Specifically for blocking-type collisions, the maximum number of events that can be recorded at a single electrode may be as low as 10 or fewer because of the larger size of the colliding entities (e.g., polymer microspheres or bacteria) relative to the size of the electrode. These small sample sizes (typically less than 300 events) limit the applicability of collision methods to problems involving heterogeneous analyte particle populations. High-throughput methods for electrochemical analysis using arrays of macroscale electrodes exist,^20−22^ but tools applicable to micro- and nanoscale systems are limited.^23^ Beyond the extension of serial electrochemical methods to new analytes, high-throughput approaches also provide opportunities to improve statistical validation, theoretical simulations, and machine learning approaches. Here, we describe the application of the array microcell method (AMCM) to high-throughput SEE measurements of nanoparticle collisions.^24^ Findings offer insight into collision experiments and suggest broader application of AMCM in high-throughput electroanalysis.

AMCM is a scanning droplet method analogous to probe-based electrochemical platforms like scanning capillary microscopy,^25^ scanning microcapillary contact method,^26−28^ and scanning electrochemical cell microscopy.^29^ Such techniques have found use in high-throughput SEE studies^23^ including single-particle characterization, surface modification, electrodeposition, and corrosion.^4,24,25,30,31^ AMCM uses a relatively large pipette [30–50 μm inner diameter (I.D.)] and operates in a two-electrode configuration consisting of a quasi-reference counter electrode inside of the pipette (loaded with electrolyte solution), with the working electrode being a single micro- or nanodisk in a microelectrode array (MEA).^32^ Electrodes in the MEA are nominally an order of magnitude smaller than the opening of the pipette. A small volume electrochemical cell is formed between an electrolyte droplet at the pipette tip and an electrode in the MEA, with the microelectrode disk defining the working electrode area, and the droplet and pipette shank defining the volume of the electrochemical cell. Moving the pipette between disks in the MEA allows each electrode to be individually addressable without complex wiring, facilitating serial electrochemical measurements. A version of AMCM was nominally described in 2018 for corrosion applications^33^ and expanded to studies of electrodeposition^24^ and characterization of combinatorial materials.^34^

During AMCM, each electrode can be controlled independently (i.e., potential program, sweep rate, etc.). When the droplet contacts a new electrode of the array, a new electrochemical experiment can be carried out in a serial fashion. With known array spacing, pitch, and dimension, the pipette positioning protocol can be optimized to match the array.^31^ Here, we take advantage of these features to automate pipette movement with a field-programmable gate array (FPGA), which enables high-throughput analysis.

We benchmark the performance of automated AMCM for high-throughput measurements via characterization of nanoelectrode arrays (NEAs) and MEAs by cyclic voltammetry and chronoamperometry (CA). Diffusion/evaporation effects in the AMCM droplet geometry are considered with finite element method (FEM) simulations.^35^ High-throughput SEE particle impact experiments with 500 nm polystyrene (PS) beads at 2.1 μm diameter Au disk electrode arrays were then performed and analyzed. Collision experiments yielded 3270 single-particle events from 671 electrodes. Data collection parameters were optimized to perform experiments in a few hours, and the results were amenable to evaluation with a U-Net deep learning model.

## Materials and Methods

### Chemicals

Solutions were prepared with deionized water (resistivity = 18.2 MΩ cm, Thermo Scientific). The following chemicals and materials were used as received: hydroxymethylferrocene (FcMeOH, 99%, Strem Chemicals), H_2_SO_4_ (Macron Chemicals), KCl (VWR Analytical grade), HClO_4_ (concentrated, Fischer Scientific), chlorotrimethylsilane (Sigma-Aldrich), ethanol (200 proof, Decon Laboratories), and PS beads (500 nm diameter, PS03N, Bangs Laboratories, Inc.).

### Microfabrication

Platinum nanoelectrode arrays (Pt NEAs) and Au MEAs were fabricated by lithography and deposition techniques, as summarized in Figures S1 and S2, with layout as shown in Figure S3.

### Array Microcell Method

**Figure 1:**
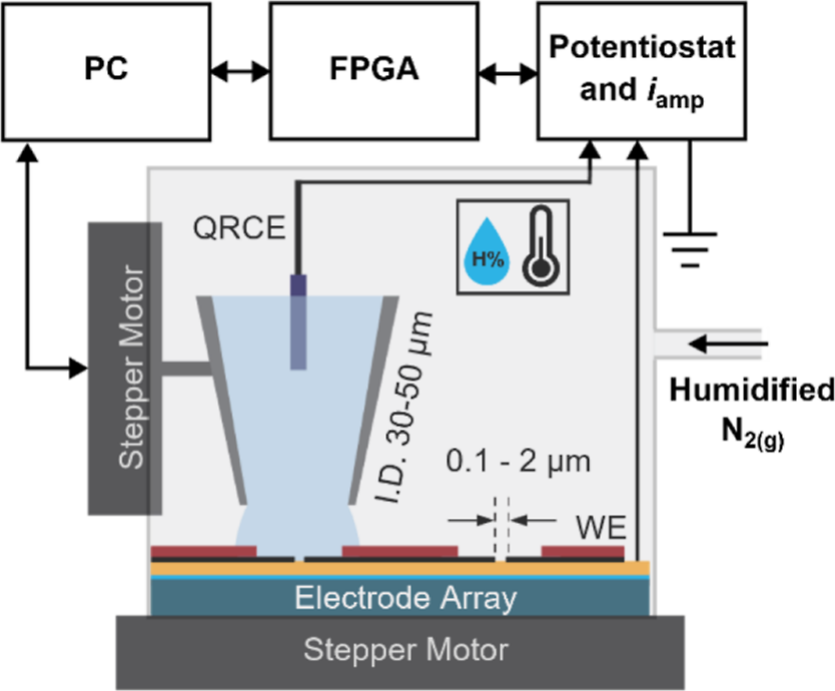
AMCM schematic for fully automated scanning and electrochemical measurements at an electrode array. Pipette approaches with *E*_app_ applied to trigger current response upon droplet contact with the electrode, measured via *i*_WE_. Environmental control consists of a humidity chamber with flowing humidified N_2_ gas and a temperature/humidity sensor.

Micropipettes and electrode arrays were fabricated, as described in Supporting Information. The automated AMCM setup was as shown in Figure 1. The electrode array was positioned before scanning via *x,y,z* stepper motors (T-JOY3 joystick control, Zaber). Positioning of the pipette was performed using a second set of *x,y,z* stepper motors with fine control (MMP3, Mad City Laboratories Inc.) and was aided by a magnified camera. During scanning, only the pipette is manipulated, while the sample remains stationary. A Chem-Clamp potentiostat (Dagan) with a 1 V/nA current amplifier head stage was used for the electrochemical measurements. An FPGA (sbRIO-9626, National Instruments) was used to collect data and control the potentiostat. The entire setup was placed in a Faraday cage and set on an air table for vibration isolation. A complete description of the AMCM scanning protocol and insurance mechanism, used to prevent pipette crashes, is detailed in Figure S4 with supplementary text. In brief, a bias (*E*_app_) is applied between the pipette and MEA during approach until electrical contact is made causing a current spike trigger, halting the pipette. An electrochemical measurement is then carried out before the pipette retracts and moves to the next electrode. This continues in a raster scan, where each pixel of the scan corresponds to a single electrode of the array. Evaporation of solvent from the pipette tip during the experimental setup caused excess analyte to build up, which was normalized following the first row of measurements of each scan. Data from electrodes in the first row, electrodes where the insurance mechanism was required, or electrodes where poor electrical contact was made were removed from the data set before further analysis.

An inner environmental chamber that contains the sample and pipette is purged with water-saturated N_2_ gas. A wet sponge also surrounded the sample. A larger chamber encloses the sample stage, pipette holder, and positioning camera in a polycarbonate box. Humidity and temperature sensors were installed directly above sample surface, ∼10 mm away from the pipette tip during measurements. All AMCM experiments were performed at ∼80% relative humidity, which was the optimal humidity for preventing excess evaporation and ensured that electrical connections to the working electrode were dry.

### Electrochemistry and Collision Experiments

To remove stray capacitance, AMCM CVs shown were background subtracted using a voltammogram where no electrical contact was made between the droplet and electrode array. To extract *E*_1/2_ and Δ*E* values from the CVs, a 40 Hz notch filter (quality factor *q* = 1) was applied to remove excess noise for each scan. Values for *E*_1/2_ were found from the global minimum in the first derivative of each CV. Values for Δ*E* were calculated as |*E*_3/4_ – *E*_1/4_|, where *E*_3/4_ and *E*_1/4_ are the normalized 3/4 and 1/4 wave potentials, respectively.^36^

Collision experiments for 500 nm diameter PS beads were recorded with beads loaded into the AMCM pipette from the start of the experiment. The filling solution for these experiments was prepared by adding 6 μL of aqueous PS bead stock (diluted 25-fold from the commercial suspension) and 0.5 mL of 2 mM FcMeOH with 0.7 mM KCl. To minimize the aggregation of the beads, the filling solution was sonicated for 2 min immediately before filling each pipette. All CA experiments were conducted with AMCM by applying potential step from −0.1 V (200 ms) to 0.4 V (10 s) vs Ag/AgCl, and current was collected at a sampling rate of 2 kHz.

Collision events were quantitated via a modified U-Net deep learning model written in PyTorch that was trained with synthesized and experimental data. Step height and *I*_lim_ were then extracted at each event. Up to 12 collision events were analyzed per electrode, with subsequent events being rejected to prevent analysis of collisions occurring from multilayers of particles. Current–time transients from *t* = 2–10 s were analyzed. Details of the U-Net model, training, and postprocessing are included in Supporting Information. Code is available on GitHub for free at https://github.com/KLDistance/unet_collision_detector.

### FEM Simulations

FEM simulations of AMCM experiments were performed using COMSOL Multiphysics v 6.1. Simulations of AMCM are detailed in Supporting Information, including determination of the meniscus solution velocity due to evaporation (v_dry_). In the figures, all simulated data are plotted as dashed lines and experimental data as solid lines.

## Results and Discussion

### Preparation and Electrochemical Characterization of Au MEA and Pt NEA

NEAs and MEAs were produced by standard microfabrication techniques (Figures S1 and S2) in a clean room environment. Wafer-level fabrication produced 16 chips for measurement containing 3.6 K electrodes each. Arrays were fabricated with a single conductive layer (Au or Pt), with an insulative layer defined by etched features in a 90 nm layer of SiN_*x*_. A recessed electrode geometry results, with Pt NEAs that contained electrode diameters of nominally 505, 330, and 100 nm (Figure S1), and Au MEAs that were 2.1 μm in diameter (Figure S2). An additional layer of patterned resist atop the SiN_*x*_ contained labels and position indicators and created a visible circle around each electrode that was 40 μm in diameter (Figure S3). This layer is necessary for visualization of the array via optical cameras, provides a hydrophobic surface for stable droplet formation during AMCM, and minimizes parasitic capacitance. Ratios of the inner pipette to electrode radii were maintained at >20 to minimize the geometry effects on mass transport.^37^

**Figure 2:**
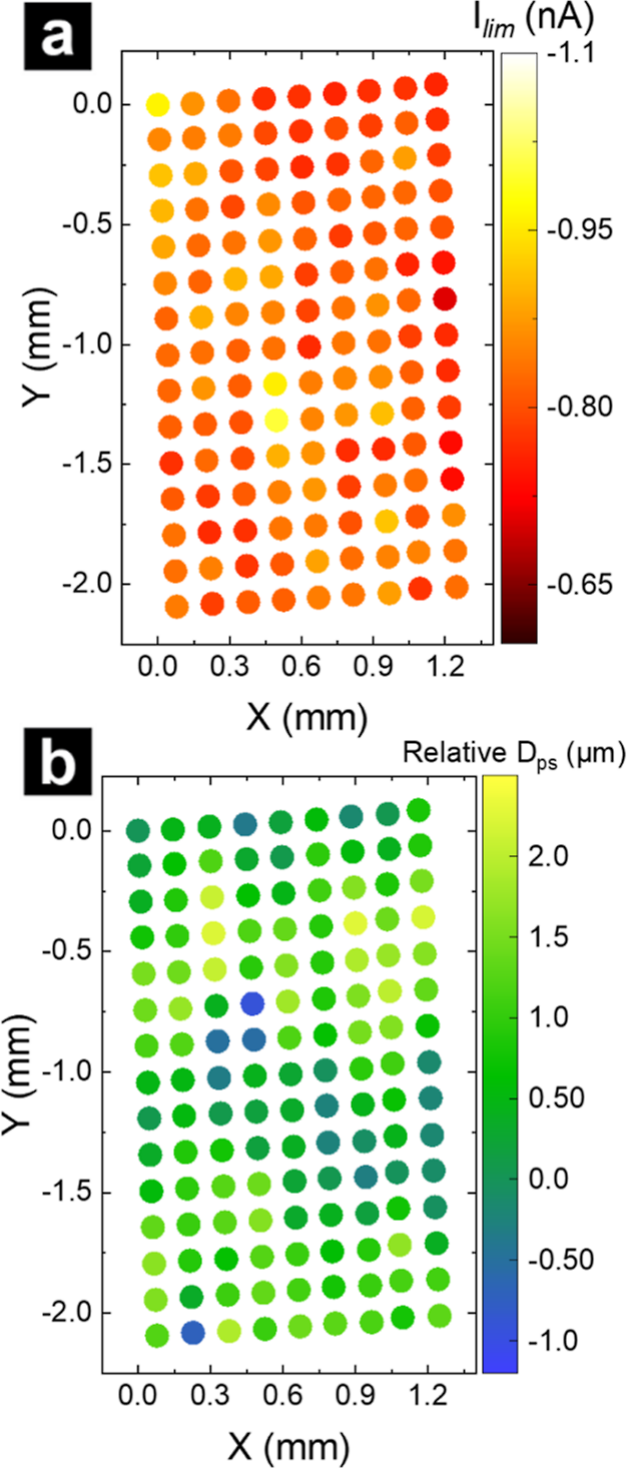
Heat maps of the *I*_lim_ current extracted at 0.6 V vs Ag/AgCl from voltammograms of FcMeOH oxidation (a) and the relative *D*_ps_ recorded by the *z*-axis of the pipette stepper-motor (b) at 2.1 μm diameter electrodes within a 15 × 9 array with 135 total electrodes measured. Pipettes (I.D. 35 μm O.D. 61 μm) were filled with 2 mM FcMeOH and 25 mM KCl, and all voltammograms were recorded at 100 mV/s.

Consistency of the electrodes within an array was assessed with electrochemical measurements. Electrode quality was inferred through kinetic measurements of the ideal redox probe FcMeOH. The AMCM setup and scanning protocol are described in Figure 1 and in Supporting Information, including Figure S4. Voltammograms at Au MEAs and Pt NEAs were collected by using micropipettes (Au MEA I.D. 35 μm O.D. 61 μm, Pt NEA I.D. 45 O.D. 75 μm) filled with 2 mM FcMeOH and 25 mM KCl. The filling solution for NEA experiments also included 4% *v/v* ethanol to improve wetting. A map of the extracted transport limited current (*I*_lim_) at 0.6 V vs Ag/AgCl and the corresponding relative probe-to-substrate distance (*D*_ps_) map are presented in Figure 2a,b, respectively. The scan began at the *x*,*y* position 0,0 and continued down the *y*-axis. The AMCM scanning protocol is configured to ensure that recessed electrodes of the array are properly aligned with the pipette tip. However, the protocol includes an “insurance mechanism”, which prevents probe crash in the event no electrical contact is made between the pipette meniscus and an electrode (see Supporting Information for description). Larger electrodes (e.g., >1 μm diameter) were found to wet easily upon contact, and scans proceeded across the 15 × 9 array (135 electrodes total) without interference, as exhibited by the homogeneous electrode response (*I*_lim_ ± standard deviation = −0.81 ± 0.04 nA) and the minimal relative *D*_ps_ variation (∼3 μm) shown in Figure 2. Using heat maps to visualize the data provides insight into the disparity in electrochemical response across the array. For example, maps revealed an e-beam lithography error that produced two electrodes (confirmed by electron microscopy) in one row of the array for 505 nm diameter Pt NEAs (Figure S6), which was observed as an increase in *I*_lim_ for FcMeOH oxidation (Figure S7). The average *I*_lim_ difference between the erroneous double feature and standard 505 nm diameter Pt electrodes at 0.6 V was 56 pA, highlighting both the uniformity in the electrode response and the ability to characterize a large number of electrodes with precision. Electrodes with such errors, or positions at which the insurance mechanism was triggered, were identified and excluded from further analysis. Properly formed electrodes show no sign of leakage of the SiN_*x*_ layer,^38^ further validating array quality.

**Figure 3:**
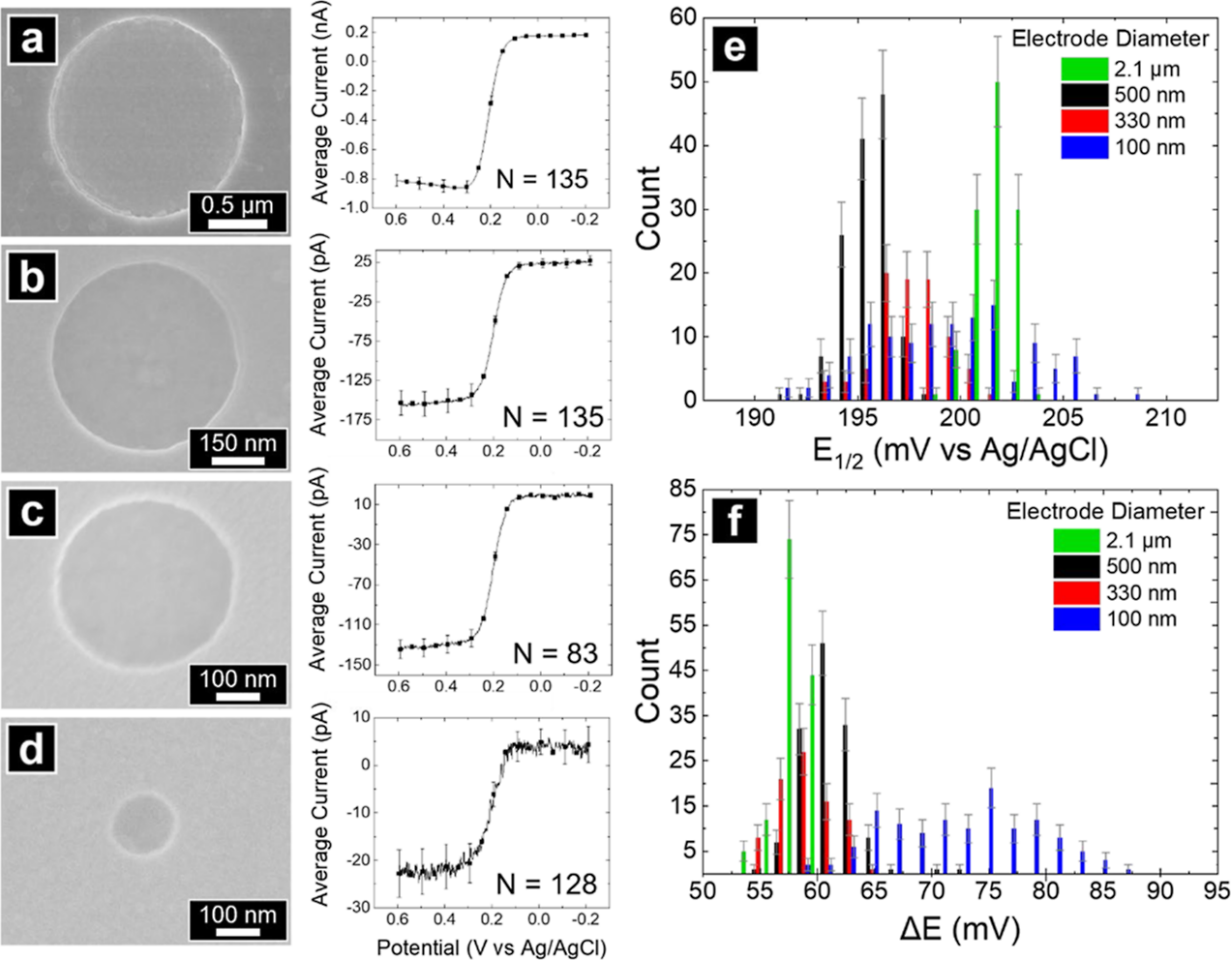
Electron micrographs and the averaged forward sweep from AMCM cyclic voltammograms of FcMeOH oxidation at nominally (a) 2.1 μm diameter Au electrodes and Pt nanoelectrode diameters of (b) 550 nm, (c) 330, and (d) 100 nm diameter Pt electrodes, respectively. Pipettes were filled with 2 mM FcMeOH and25 mM KCl (and 4% v/v EtOH for Pt NEA measurements); all voltammograms recorded at 100 mV/s and error bars represent the standard deviation. Extracted (e) *E*_1/2_ and (f) Δ*E* from voltammograms in (a–d); error bars are the square root of the number of counts in each bin.

Aggregate AMCM voltammetry results for FcMeOH oxidation on Au-MEAs and Pt-NEAs are shown in Figure 3. The averaged forward sweep from voltammograms at 2.1 μm Au-MEA, 505, 330, and 100 nm Pt-NEAs are shown with the corresponding electron micrographs in Figure 3a–d. The sigmoidal shape of voltammograms at Pt NEAs is indicative of a radial diffusion profile produced at each electrode similar to conventional ultramicroelectrodes (UME).^39^ Deviation in *I*_lim_ and shape of voltammograms, from the behavior of a disk UME^40^ in a conventional cell for 2.1 μm disk diameter Au-MEA, was investigated in detail with FEM simulations. Histograms of *E*_1/2_ and Δ*E* values extracted from AMCM voltammograms are presented in Figure 3e–f. For 2.1 μm diameter Au MEAs, the extracted *E*_1/2_ and Δ*E* values were 201 ± 3 mV vs Ag/AgCl and 57 ± 1 mV, respectively. The Δ*E* value is consistent with reversible one electron transfer using the Tomeš criterion.^41^

The known formal potential for the oxidation of FcMeOH is 0.197 V vs Ag/AgCl,^42,43^ and the measured *E*_1/2_ values for 505, 330, and 100 nm electrodes were 195 ± 1, 197 ± 1, and 199 ± 4 mV vs Ag/AgCl, respectively. For 505 and 330 nm diameter Pt electrodes, Δ*E* was 61 ± 2 and 59 ± 2 mV, respectively, while 100 nm electrodes exhibited Δ*E* of 73 ± 7 mV (Figure 3e). We postulate two possible causes for the shift in the Δ*E* at 100 nm diameter Pt NEAs. First, the ratio of recess depth and electrode radius is approximately 1.7, which is large enough to cause geometry-dependent change in mass transport at the electrode surface and lead to deviation in the calculated Δ*E*.^37^ Second, small remnants of SiN_*x*_, undetectable by standard characterization methods, such as SEM, are more likely to remain on smaller electrodes after reactive ion etching. Partially blocked electrodes could result in slower apparent kinetics.^44,45^ Smaller electrodes (<100 nm diameter) have also been reported to exhibit slower apparent kinetics in voltammetric measurements; however, this is experimentally improbable to observe at the size range explored in this study.^46^

### Environmental and Geometric Effects on the AMCM Electrochemical Response

AMCM voltammetry displays two critical differences from the expected behavior for conventional disk UMEs: first, a deviation in shape including a nonzero current at the start of the experiment (i.e., when *E*_app_ ≪ *E*_1/2_) and second, recorded values of *I*_lim_ for FcMeOH oxidation being larger than what the UME theory permits. We attribute these differences to the combined influences of the AMCM geometry and solvent drying at the pipette meniscus. These effects were investigated by comparing experimental AMCM CVs and CAs to FEM simulations.

During AMCM, the pipette–substrate contact produces a meniscus near the pipette outer radius, around 1–4 μm tall, where the electrolyte solution is exposed to air in the humidity cell. Our findings suggest that the small amount of solvent evaporation occurring at this pinned meniscus creates convection inside the pipette, which increases the transport of FcMeOH at the MEA disk. A similar phenomenon has been investigated previously in microfluidics applications to produce evaporative pumping in microchannels.^32^ Drying further increases the amount of FcMeOH available to the MEA disk due to the accumulation of FcMeOH near the meniscus as the evaporating electrolyte solution is replenished from the back of the pipette.^47−49^

**Figure 4:**
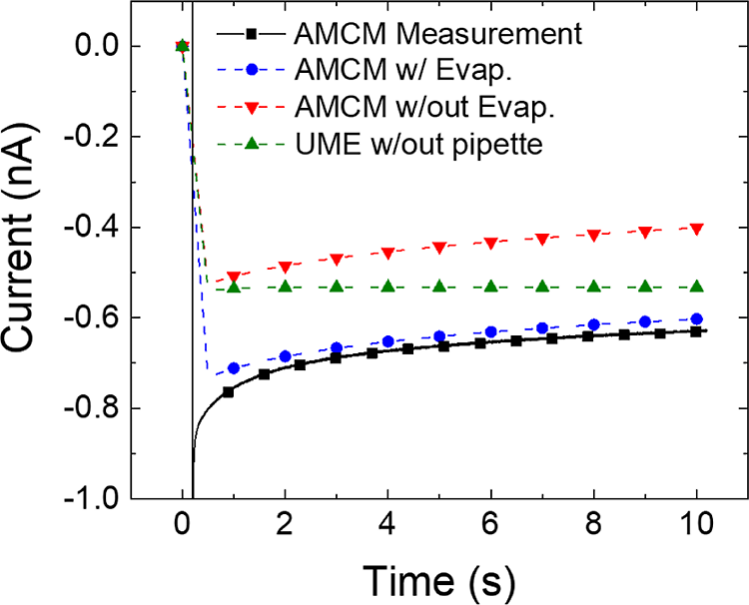
Comparison of averaged AMCM chronoamperograms (solid line, *N* = 135) at 2.1 μm Au electrodes and the simulated response (dashed lines) with (*v*_dry_ = 6 μm/s) and without (*v*_dry_ = 0 μm/s) evaporation physics. Pipette (I.D. 35 μm O.D. 61 μm) filled with 2 mM FcMeOH and 25 mM KCl.

To implement evaporation at the pipette meniscus in the FEM simulation, the meniscus was assigned an outflow velocity boundary condition in a Navier–Stokes simulation, with a coupled flux boundary condition in the Nernst–Planck simulation (Figure S8). A meniscus outflow velocity (*v*_dry_), analogous to an evaporation rate, was set to 6–8 μm/s by adjusting its value so that the simulated CA for a set of three Au-MEA disk diameters (2.1, 4.4, and 6.6 μm) matched those from the experiments shown in Figure S9a. The flux boundary condition set the net flux (diffusion and convection) of FcMeOH/FcMeOH^+^ to 0; with the coupled velocity condition, this had the effect of producing diffusive flux dependent on *v*_dry_ at the meniscus, i.e., solutes are concentrated as the solvent evaporates and convection is generated to replace the lost solvent.^47−49^ This simplified approach to modeling the solvent evaporation is realistic here because the amount of evaporation is low due to the humidity cell, and the MEA disk is relatively far from the drying meniscus. Figure 4 shows an averaged, measured AMCM CA for 2.1 μm diameter Au electrodes plotted with the results from the FEM model. The three simulated current plots are AMCM configuration with evaporation at the meniscus (blue), AMCM configuration without evaporation (red), and a UME in a large volume cell (green).

To accurately reflect the small amount of additional drying that occurs before the start of the experiment (i.e., when the pipette is moved to a new disk), simulations included a 15 s accumulation period in which drying was allowed to occur, but the MEA disk was deactivated for FcMeOH oxidation by setting *E*_app_ = −0.3 V vs *E*_f_. After the accumulation period, *E*_app_ was stepped to produce simulated data with *D*_ps_ = 2 μm and *v*_dry_ = 6 or 8 μm/s, as shown in Figures 4 and S10, respectively. Concentration profiles, solution velocities, and concentration with arrow plots showing convective and diffusive flux of FcMeOH are shown in Figures S11 and S12. Models establish that drying at the meniscus increases the transport of FcMeOH to the disk by two mechanisms: convection toward the disk from the back of the pipette by flow of the solution and diffusion, which further increases the FcMeOH concentration in the droplet due to accumulation of the solute at the drying meniscus.

Chronoamperograms in Figure 4 exhibit a decrease in the transport limited current magnitude over time for both the experimental data and AMCM models. This occurs because in the confined geometry of AMCM, FcMeOH is steadily depleted in the droplet faster than it can be replenished from the pipette. This depletion effect is further illustrated by the dependence on electrode size shown in Figure S7a, where larger electrodes exhibit a steeper decrease in the current response for both experiments and simulations. Convective flux can replenish the droplet cell, affecting the rate of FcMeOH depletion, where a higher *v*_dry_, larger *D*_ps_, or larger pipette will increase the convection of the solution toward the pipette tip, counteracting the low FcMeOH diffusive flux. Eventually, as more evaporation occurs, convection will dominate, causing an increase in oxidation current over time, as shown by the simulated responses in Figure S7b. At very high rates of evaporation (which were not observed here), transport of FcMeOH (or any other analyte) will be impacted by its solubility, setting an upper limit on evaporative effects. The effect of the AMCM geometry and evaporation is analyzed in detail in the Supporting Information for voltammogram measurements (Figure S10).

### High-Throughput Single-Entity Nanoparticle Collision Studies with AMCM

**Figure 5:**
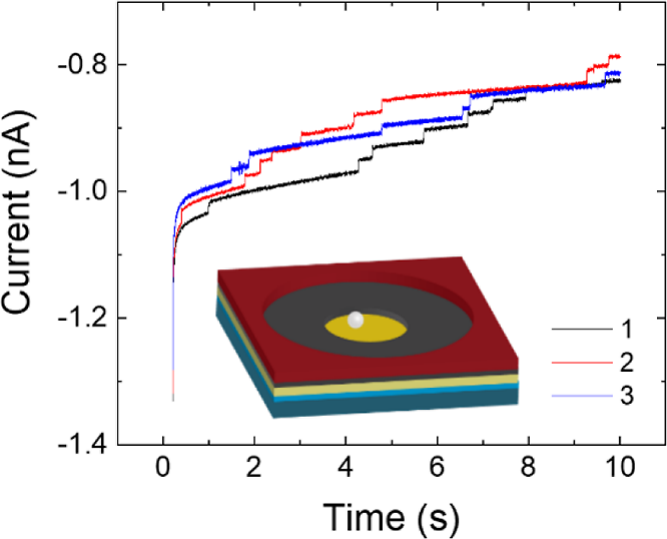
Chronoamperograms from three 2.1 μm diameter Au electrodes where *E*_app_ was held at 0.4 V vs Ag/AgCl for 10 s. Pipettes contained 500 nm PS beads in 2 mM FcMeOH and 0.7 mM KCl. Inset is a to-scale illustration of a single 500 nm bead at an electrode surface.

Automated AMCM provides an excellent opportunity to examine particle collision experiments in a high-throughput manner. AMCM particle collision experiments were performed with 2.1 μm diameter Au MEAs and 500 nm diameter PS beads (pipette I.D. 45 μm, O.D. 75 μm). In addition to beads, pipettes were filled with 2 mM FcMeOH and 0.7 mM KCl solution. At each electrode, *E*_app_ = 0.4 V vs Ag/AgCl was applied for 10 s to facilitate the migration of the negatively charged PS beads to the Au surface during FcMeOH oxidation.^5^ Representative AMCM CA traces containing steps correlating to particle adsorption are shown in Figure 5 (additional CA collision data in Figure S13), with an illustration of a single particle on an electrode to scale. Quantitation of 3270 collisions events from 671 electrodes was performed by a deep learning modified U-Net model. The U-Net model was pretrained with synthesized CA curves containing steps and was fine-tuned using experimental CAs of FcMeOH oxidation. Step magnitudes were captured by taking the difference between the average of 8 px before and after the rising edge of each event in a post-processing step. Details of data analysis via deep learning are listed in Supporting Information (Figure S5).

**Figure 6:**
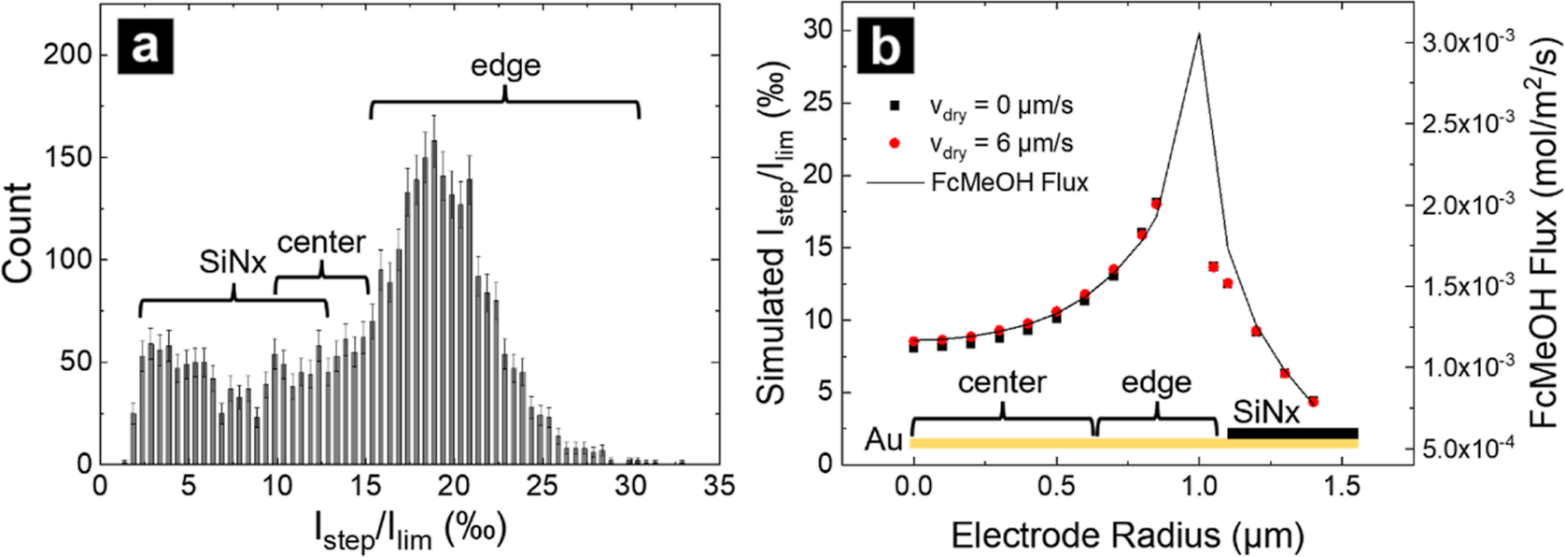
(a) Histogram of step frequency where step heights (*I*_step_) are normalized to the baseline transport limited current (*I*_lim_) just before each event. (b) Simulated normalized step height across the electrode surface without (black boxes) and with (red boxes) evaporation. Solid black line is the simulated FcMeOH flux where *V*_dry_ = 6 μm/s from the 2D axisymmetric model with the same dimensions and conditions as the collision model.

A histogram of the step heights normalized to the baseline current is shown in Figure 6a. A bimodal distribution was observed in step heights, with distributions centered at 3.35 and 18.85‰ (Figure 6a). Peaks in distributions arise because the normal flux of the redox species is not uniform over the MEA disk surface; larger steps are observed when particles land at the electrode edge than when they land at the center.^50,51^

Current steps resulting from particles adsorbed at different radial positions on the electrode surface were simulated to correlate the step height with the particle landing location. Simulated step heights and total FcMeOH flux 100 nm above the electrode surface are plotted versus the particle position in Figure 6b. Similar to experiments using conventional UMEs, simulated steps show dependence of flux as a function of particle position on the electrode.^50−52^ Electrode geometry was divided into three categories, defined by the radial position of the particle on the electrode (see Figure 6b): center (0–0.6 μm), edge (0.6–1.05 μm), and SiN_*x*_ (1.05–1.4 μm). For simulated positions with *r* ≥ 1.05 μm, the particle rests on the SiN_*x*_ ledge. From these simulations, events with a step magnitude of 2–12.5 and 10 to 15‰ were attributed to collisions at the SiN_*x*_ and center regions, respectively. Collisions at the electrode edge comprise most of the events ranging from 12.5 to 25‰. The measured peak step height at the electrode edge was 18.8‰. Simulated step height for the position nearest the electrode edge (0.85 μm) was 18.02 and 18.14‰ with (*v*_dry_ = 6 μm/s) and without (*v*_dry_ = 0 μm/s) evaporation (Figure 6b). Simulations establish that convection caused by evaporation in these experiments does not have a significant effect on the expected step heights as a function of the landing location of the particle. Flux of FcMeOH rapidly drops at the electrode edge, leading to collisions with step heights of roughly 2 to 14.5‰ with a peak at 3.35‰, as shown in Figure 6a. However, there is more variation in the geometry at the Au/SiN_*x*_ interface, making it possible that some collisions classified as “edge” may be on the SiN_*x*_ ledge. In addition, simulated step magnitudes at a radius of 1.05–1.4 μm are between 4.3 and 12.5‰ (Figure 6b), indicating that the measured collision events <4‰ are either particles beyond a radius of 1.4 μm moving on the electrode surface^51^ or colliding on top of previously adsorbed particles.

SEE particle collisions at 671 electrodes were collected by AMCM in ∼5 h of experiments, including sample preparation, where four to five collisions were recorded on average per electrode. Traditional methods employing single UMEs for each collision experiment would require multiple days to weeks, estimating a nominal experimental acquisition time at an individual electrode of ∼15 min. The bimodal distribution in Figure 6 has been reported previously for disk UMEs by Moazzenzade and co-workers;^48^ the present study provides additional experimental confirmation with statistical validation owing to the large sample size enabled by automated AMCM.

## Conclusions

We have advanced AMCM to allow for automated serial measurements of hundreds of electrodes in a single experiment. Cyclic voltammetry and CA using AMCM at electrodes 0.1–2 μm in diameter were demonstrated and analyzed. SEE measurements of nanoparticle collisions with machine learning-aided data analysis confirm AMCM as a suitable tool for high-throughput SEE experiments at the microscale. By the combination of automated instrumentation, microfabrication, and data analysis, AMCM provides a route to high-throughput, serial electrochemical experimentation.

## Supporting Information Available

Additional experimental details, methods, AMCM electrochemical data, and FEM simulations of CA/CV (PDF)
COMSOL model report for AMCM 2D CA (PDF)
COMSOL model report for AMCM 3D CA (PDF)
COMSOL model report for AMCM 2D CV (PDF)

## References

[ref1] Crooks R. M. Concluding remarks: single entity electrochemistry one step at a time. Faraday Discuss. 2016, 193, 533–547. 10.1039/C6FD00203J. 27761542

[ref2] Baker L. A. Perspective and Prospectus on Single-Entity Electrochemistry. J. Am. Chem. Soc. 2018, 140, 15549–15559. 10.1021/jacs.8b09747. 30388887 PMC8720287

[ref3] Ying Y.-L.; Wang J.; Leach A. R.; Jiang Y.; Gao R.; Xu C.; Edwards M. A.; Pendergast A. D.; Ren H.; Weatherly C. K. T.; Wang W.; Actis P.; Mao L.; White H. S.; Long Y.-T. Single-entity electrochemistry at confined sensing interfaces. Sci. China Chem. 2020, 63, 589–618. 10.1007/s11426-020-9716-2.

[ref4] Wahab O. J.; Kang M.; Unwin P. R. Scanning electrochemical cell microscopy: A natural technique for single entity electrochemistry. Curr. Opin. Electrochem. 2020, 22, 120–128. 10.1016/j.coelec.2020.04.018.

[ref5] Quinn B. M.; van’t Hof P. G.; Lemay S. G. Time-Resolved Electrochemical Detection of Discrete Adsorption Events. J. Am. Chem. Soc. 2004, 126, 8360–8361. 10.1021/ja0478577. 15237976

[ref6] Kang M.; Perry D.; Kim Y.-R.; Colburn A. W.; Lazenby R. A.; Unwin P. R. Time-Resolved Detection and Analysis of Single Nanoparticle Electrocatalytic Impacts. J. Am. Chem. Soc. 2015, 137, 10902–10905. 10.1021/jacs.5b05856. 26264494

[ref7] Alpuche Aviles M. A.; Gutierrez-Portocarrero S. Why measure particle-by-particle electrochemistry? A tutorial and perspective. J. Mex. Chem. Soc. 2023, 67, 566–580. 10.29356/jmcs.v67i4.2014.

[ref8] Boika A.; Thorgaard S. N.; Bard A. J. Monitoring the Electrophoretic Migration and Adsorption of Single Insulating Nanoparticles at Ultramicroelectrodes. J. Phys. Chem. B 2013, 117, 4371–4380. 10.1021/jp306934g. 23092206

[ref9] Boika A.; Bard A. J. Time of First Arrival in Electrochemical Collision Experiments as a Measure of Ultralow Concentrations of Analytes in Solution. Anal. Chem. 2015, 87, 4341–4346. 10.1021/acs.analchem.5b00037. 25803279

[ref10] Bonezzi J.; Boika A. Deciphering the Magnitude of Current Steps in Electrochemical Blocking Collision Experiments and Its Implications. Electrochim. Acta 2017, 236, 252–259. 10.1016/j.electacta.2017.03.090.

[ref11] Sekretareva A. Single-entity electrochemistry of collision in sensing applications. Senor. Actuator Rep. 2021, 3, 100037 10.1016/j.snr.2021.100037.

[ref12] Wang H.; Yang C.; Tang H.; Li Y. Stochastic Collision Electrochemistry from Single G-Quadruplex/Hemin: Electrochemical Amplification and MicroRNA Sensing. Anal. Chem. 2021, 93, 4593–4600. 10.1021/acs.analchem.0c05055. 33660976

[ref13] Chen C.-H.; Ravenhill E. R.; Momotenko D.; Kim Y.-R.; Lai S. C. S.; Unwin P. R. Impact of Surface Chemistry on Nanoparticle-Electrode Interactions in the Electrochemical Detection of Nanoparticle Collisions. Langmuir 2015, 31, 11932–11942. 10.1021/acs.langmuir.5b03033. 26448140

[ref14] Defnet P. A.; Anderson T. J.; Zhang B. Stochastic collision electrochemistry of single silver nanoparticles. Curr. Opin. Electrochem. 2020, 22, 129–135. 10.1016/j.coelec.2020.06.004.

[ref15] Karunathilake N.; Gutierrez-Portocarrero S.; Subedi P.; Alpuche-Aviles M. A. Reduction Kinetics and Mass Transport of ZnO Single Entities on a Hg Ultramicroelectrode. Chemelectrochem 2020, 7, 2248–2257. 10.1002/celc.202000031.

[ref16] Lu S.-M.; Chen J.-F.; Peng Y.-Y.; Ma W.; Ma H.; Wang H.-F.; Hu P.; Long Y.-T. Understanding the Dynamic Potential Distribution at the Electrode Interface by Stochastic Collision Electrochemistry. J. Am. Chem. Soc. 2021, 143, 12428–12432. 10.1021/jacs.1c02588. 34347459

[ref17] Dick J. E.; Renault C.; Bard A. J. Observation of Single-Protein and DNA Macromolecule Collisions on Ultramicroelectrodes. J. Am. Chem. Soc. 2015, 137, 8376–8379. 10.1021/jacs.5b04545. 26108405

[ref18] Peng Y.-Y.; Qian R.-C.; Hafez M. E.; Long Y.-T. Stochastic Collision Nanoelectrochemistry: A Review of Recent Developments. Chemelectrochem 2017, 4, 977–985. 10.1002/celc.201600673.

[ref19] Ma H.; Chen J.-F.; Wang H.-F.; Hu P.-J.; Ma W.; Long Y.-T. Exploring dynamic interactions of single nanoparticles at interfaces for surface-confined electrochemical behavior and size measurement. Nat. Commun. 2020, 11, 2307 10.1038/s41467-020-16149-0. 32385284 PMC7210955

[ref20] Pence M. A.; Rodríguez O.; Lukhanin N. G.; Schroeder C. M.; Rodríguez-López J. Automated Measurement of Electrogenerated Redox Species Degradation Using Multiplexed Interdigitated Electrode Arrays. ACS Meas. Sci. Au 2023, 3, 62–72. 10.1021/acsmeasuresciau.2c00054. 36817007 PMC9936799

[ref21] Gerroll B. H. R.; Kulesa K. M.; Ault C. A.; Baker L. A. Legion: An Instrument for High-Throughput Electrochemistry. ACS Meas. Sci. Au 2023, 3, 371–379. 10.1021/acsmeasuresciau.3c00022. 37868360 PMC10588931

[ref22] Rein J.; Annand J. R.; Wismer M. K.; Fu J.; Siu J. C.; Klapars A.; Strotman N. A.; Kalyani D.; Lehnherr D.; Lin S. Unlocking the Potential of High-Throughput Experimentation for Electrochemistry with a Standardized Microscale Reactor. ACS Cent. Sci. 2021, 7, 1347–1355. 10.1021/acscentsci.1c00328. 34471679 PMC8393209

[ref23] Xu X.; Valavanis D.; Ciocci P.; Confederat S.; Marcuccio F.; Lemineur J.-F.; Actis P.; Kanoufi F.; Unwin P. R. The New Era of High-Throughput Nanoelectrochemistry. Anal. Chem. 2023, 95, 319–356. 10.1021/acs.analchem.2c05105. 36625121 PMC9835065

[ref24] Alden S. E.; Siepser N. P.; Patterson J. A.; Jagdale G. S.; Choi M.; Baker L. A. Array Microcell Method (AMCM) for Serial Electroanalysis. Chemelectrochem 2020, 7, 1084–1091. 10.1002/celc.201901976. 36588586 PMC9798888

[ref25] Lohrengel M. M. Electrochemical capillary cells. Corros. Eng., Sci. Technol. 2004, 39, 53–58. 10.1179/147842204225016877.

[ref26] Williams C. G.; Edwards M. A.; Colley A. L.; Macpherson J. V.; Unwin P. R. Scanning Micropipet Contact Method for High-Resolution Imaging of Electrode Surface Redox Activity. Anal. Chem. 2009, 81, 2486–2495. 10.1021/ac802114r. 19265426

[ref27] Gateman S. M.; Georgescu N. S.; Kim M.-K.; Jung I.-H.; Mauzeroll J. Efficient Measurement of the Influence of Chemical Composition on Corrosion: Analysis of an Mg-Al Diffusion Couple Using Scanning Micropipette Contact Method. J. Electrochem. Soc. 2019, 166, C624–C630. 10.1149/2.0681916jes.

[ref28] Li Y.; Morel A.; Gallant D.; Mauzeroll J. Oil-Immersed Scanning Micropipette Contact Method Enabling Long-term Corrosion Mapping. Anal. Chem. 2020, 92, 12415–12422. 10.1021/acs.analchem.0c02177. 32786459

[ref29] Ebejer N.; Güell A. G.; Lai S. C. S.; McKelvey K.; Snowden M. E.; Unwin P. R. Scanning Electrochemical Cell Microscopy: A Versatile Technique for Nanoscale Electrochemistry and Functional Imaging. Annu. Rev. Anal. Chem. 2013, 6, 329–351. 10.1146/annurev-anchem-062012-092650. 23560932

[ref30] Ornelas I. M.; Unwin P. R.; Bentley C. L. High-Throughput Correlative Electrochemistry-Microscopy at a Transmission Electron Microscopy Grid Electrode. Anal. Chem. 2019, 91, 14854–14859. 10.1021/acs.analchem.9b04028. 31674764

[ref31] Siepser N. P.; Choi M.-H.; Alden S. E.; Baker L. A. Single-Entity Electrocatalysis at Electrode Ensembles Prepared by Template Synthesis. J. Electrochem. Soc. 2021, 168, 126526 10.1149/1945-7111/ac44b8.

[ref32] Compton R. G.; Wildgoose G. G.; Rees N. V.; Streeter I.; Baron R. Design, fabrication, characterisation and application of nanoelectrode arrays. Chem. Phys. Lett. 2008, 459, 1–17. 10.1016/j.cplett.2008.03.095.

[ref33] Jin Y.; Lai Z.; Bi P.; Yan S.; Wen L.; Wang Y.; Pan J.; Leygraf C. Combining lithography and capillary techniques for local electrochemical property measurements. Electrochem. Commun. 2018, 87, 53–57. 10.1016/j.elecom.2017.12.027.

[ref34] Lai Z.; Zou Y.; Zhao Z.; Huang F.; Liu P.; Lai T.; Jin Y. An Automated Test Platform for High-Throughput Micro-Electrochemical Characterization of Metallic Materials and Its Application on a Fe-Cr-Ni Combinatorial Materials Chip. J. Electrochem. Soc. 2021, 168, 091501 10.1149/1945-7111/ac24bc.

[ref35] Anderson K. L.; Edwards M. A. Evaluating Analytical Expressions for Scanning Electrochemical Cell Microscopy (SECCM). Anal. Chem. 2023, 95, 8258–8266. 10.1021/acs.analchem.3c00216. 37191580

[ref36] Wahab O. J.; Kang M.; Meloni G. N.; Daviddi E.; Unwin P. R. Nanoscale Visualization of Electrochemical Activity at Indium Tin Oxide Electrodes. Anal. Chem. 2022, 94, 4729–4736. 10.1021/acs.analchem.1c05168. 35255211 PMC9007413

[ref37] Guo J.; Lindner E. Cyclic voltammetry at shallow recessed microdisc electrode: Theoretical and experimental study. J. Electroanal. Chem. 2009, 629, 180–184. 10.1016/j.jelechem.2009.01.030. PMC2766860 20160948

[ref38] Bodappa N. Rapid assessment of platinum disk ultramicroelectrodes’ sealing quality by a cyclic voltammetry approach. Anal. Methods 2020, 12, 3545–3550. 10.1039/D0AY00649A. 32672251

[ref39] Sun P.; Mirkin M. V. Kinetics of Electron-Transfer Reactions at Nanoelectrodes. Anal. Chem. 2006, 78, 6526–6534. 10.1021/ac060924q. 16970330

[ref40] Bond A. M.; Luscombe D.; Oldham K. B.; Zoski C. G. A comparison of the chronoamperometric response at inlaid and recessed disc microelectrodes. J. Electroanal. Chem. 1988, 249, 1–14. 10.1016/0022-0728(88)80345-0.

[ref41] Tomeš J. Polarographic studies with the dropping mercury kathode. LXVII. Equation of the polarographic wave in the electrodeposition of hydrogen from strong and weak acids. Collect. Czech. Chem. Commun. 1937, 9, 150–167. 10.1135/cccc19370150.

[ref42] Miao W.; Ding Z.; Bard A. J. Solution Viscosity Effects on the Heterogeneous Electron Transfer Kinetics of Ferrocenemethanol in Dimethyl Sulfoxide-Water Mixtures. J. Phys. Chem. B 2002, 106, 1392–1398. 10.1021/jp013451u.

[ref43] Taherkhani F. Investigation of ion pairs in electrochemical ferrocene methanol -Ferrocenium methanol system in presence of supporting electrolyte. Electrochim. Acta 2022, 431, 141014 10.1016/j.electacta.2022.141014.

[ref44] Amatore C.; Savéant J.; Tessier D. Charge transfer at partially blocked surfaces: A model for the case of microscopic active and inactive sites. J. Electroanal. Chem. 1983, 147, 39–51. 10.1016/S0022-0728(83)80055-2.

[ref45] Davies T. J.; Banks C. E.; Compton R. G. Voltammetry at spatially heterogeneous electrodes. J. Solid State Electrochem. 2005, 9, 797–808. 10.1007/s10008-005-0699-x.

[ref46] Liu Y.; Chen S. Theory of Interfacial Electron Transfer Kinetics at Nanometer-Sized Electrodes. J. Phys. Chem. C 2012, 116, 13594–13602. 10.1021/jp300696u.

[ref47] Salmon J.-B.; Doumenc F. Buoyancy-driven dispersion in confined drying of liquid binary mixtures. Phys. Rev. E: Stat. Phys., Plasmas, Fluids, Relat. Interdiscip. Top. 2020, 5, 024201 10.1103/PhysRevFluids.5.024201.

[ref48] Bacchin P.; Leng J.; Salmon J.-B. Microfluidic Evaporation, Pervaporation, and Osmosis: From Passive Pumping to Solute Concentration. Chem. Rev. 2022, 122, 6938–6985. 10.1021/acs.chemrev.1c00459. 34882390

[ref49] Fedorchenko A. I.; Chernov A. A. Exact solution of the problem of gas segregation in the process of crystallization. Int. J. Heat Mass Transfer 2003, 46, 915–919. 10.1016/S0017-9310(02)00349-6.

[ref50] Moazzenzade T.; Walstra T.; Yang X.; Huskens J.; Lemay S. G. Ring Ultramicroelectrodes for Current-Blockade Particle-Impact Electrochemistry. Anal. Chem. 2022, 94, 10168–10174. 10.1021/acs.analchem.2c01503. 35792954 PMC9310007

[ref51] Fosdick S. E.; Anderson M. J.; Nettleton E. G.; Crooks R. M. Correlated Electrochemical and Optical Tracking of Discrete Collision Events. J. Am. Chem. Soc. 2013, 135, 5994–5997. 10.1021/ja401864k. 23590646

[ref52] Lemay S. G.; Renault C.; Dick J. E. Particle mass transport in impact electrochemistry. Curr. Opin. Electrochem. 2023, 39, 101265 10.1016/j.coelec.2023.101265.

