# Supplementary material for: High-Throughput Single-Entity Electrochemistry with Microelectrode Arrays
Source: Anal Chem. 2024 May 23;96(22):9177–84. doi: 10.1021/acs.analchem.4c01092 (PMC11154736; doi:10.1021/acs.analchem.4c01092)
Supplement: Supplementary file 1 — ac4c01092_si_001.pdf [file ac4c01092_si_001.pdf]

Supporting Information

High-throughput single-entity electrochemistry with microelectrode arrays

Sasha E. Alden[1], Lingjie Zhang[1], Yunong Wang[1], Nickolay V. Lavrik[2], Scott N. Thorgaard[3,*], and Lane A. Baker[1,*]

[1]Department of Chemistry, Texas A&M University, College Station, Texas 77843
[2]Center for Nanophase Materials Sciences, Oak Ridge National Laboratory, Oak Ridge, Tennessee 37830
[3]Department of Chemistry, Grand Valley State University, Allendale, Michigan 49401

*Corresponding Authors:
Scott N. Thorgaard - Department of Chemistry, Grand Valley State University, Allendale, Michigan 49401
Email: thorgaas@gvsu.edu

Lane A. Baker - Department of Chemistry, Texas A&M University, College Station, Texas 77843
Email: lane.baker@tamu.edu

Supplemental Methods

Microfabrication

Pt NEA (**Figure S1**). Platinum deposition was carried out after deposition of 10 nm Ti via metal evaporation, in a two-step process by first depositing ~60 nm via Pt e-beam evaporation then followed by ~20 nm of DC sputtered Pt onto a 100 mm diameter, 100 nm SiO_2 coated Si wafer (100 mm diameter). Then 7 nm of SiO_2 followed by 90 nm of silicon nitride (SiNx) deposited using plasma enhanced chemical vapor deposition. The two-step Pt deposition process was necessary to produce a smooth coherent Pt surface

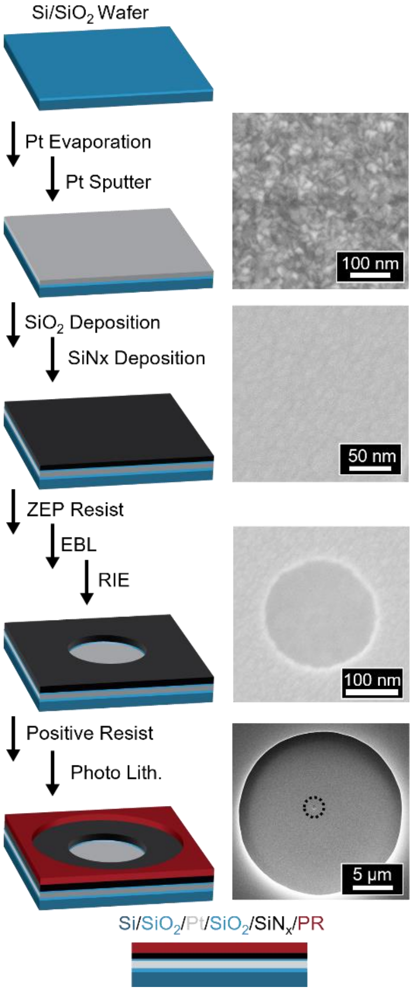

Figure S1. Pt NEA fabrication scheme with accompanying electron micrographs for at each step. Thickness of each layer is as follows: Si/SiO_2 /Pt(~70 nm)/SiO_2(7 nm)/SiN_x(~90 nm) /positive resist (1.2 μm).

at each electrode, as cracking occurred with a purely evaporated film during the deposition. SiN_x was patterned with arrays of nominally 100, 330 and 505 nm diameter nanoelectrode arrays at the wafer scale with e-beam lithography (EBL). Following development of the EBL resist (**Figure S1** ZEP resist), the SiN_x surface was subjected to anisotropic reactive ion etching (RIE). Contact pads of 1 mm x 1 mm were included for electrical contact at each chip (**Figure S3**).

Au MEA (**Figure S2**): Onto a SiO_2 coated Si wafer (100 mm diameter), 100 nm Au was deposited via e-beam physical vapor deposition. Plasma enhanced CVD then deposited 90 nm of SiN_x onto the Au electrode layer before applying a microelectrode pattern with photolithography (S1818 resist). RIE etching of SiN_x was carried out to define microelectrode arrays in SiN_x and a 2x2 mm contact pad.

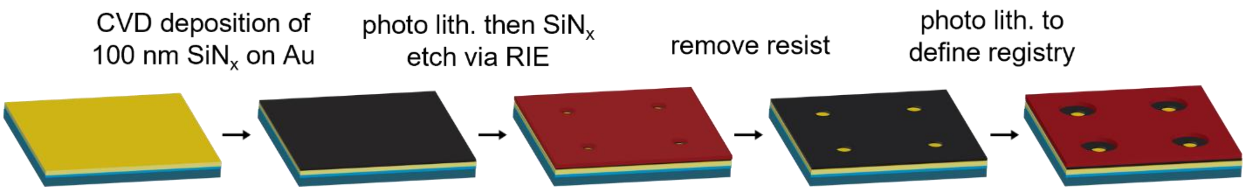

Figure S2. Fabrication scheme for Au disk microelectrode arrays, where electrodes are 2.1 μm in diameter and 90 nm recessed in SiNx.

All arrays: Following etching of SiN_x, remaining resist was removed via solvent stripping and O_2 ashing before applying a 1.2 µm thick layer of AZ1521. This final layer of resist was patterned with 40 µm diameter circles around each electrode, electrode size labels, and location markers (**Figure S1** final step for images and **Figure S3**). Wafers were finally diced into 17 x 17 mm chips each containing 3.6K electrodes of multiple sizes and a contact pad. Each chip included 16 arrays of 225 electrodes each with an electrode spacing of 150 µm, with 3 or 4 different sized electrodes depending on the design (**Figure S3**).

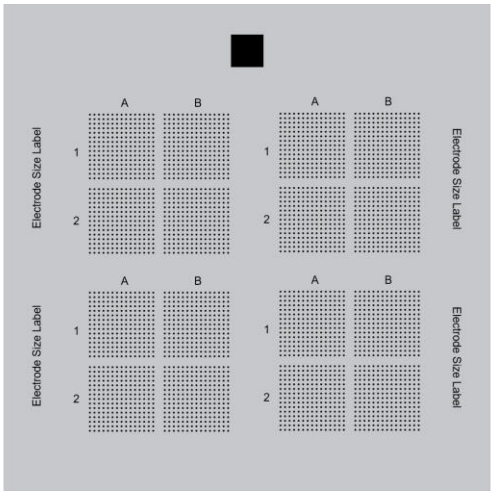

Figure S3. Illustrated chip layout for electrode arrays containing 3.6 K electrodes total.

Array Preparation for AMCM Measurements

Finished electrode array chips were prepared for electrochemistry by successive O_2 plasma cleaning (3 mins, 6.8 Watt, Harrick Plasma) and 1 hr soak in 1 M H_2SO_4 (Pt NEAs) or CV cycling in 0.1 M $HClO_4$ from 0 to 1.4 V *vs* Ag/AgCl (Au MEAs). All chips were then rinsed with RT DI water and allowed to soak in warmed (~60 °C) DI water for 10 mins before drying with filtered air. Proper sample preparation was crucial for providing

not only clean electrodes, but the surface conditions needed for the nearly instantaneous wetting required during AMCM scanning.

Pipettes and QRCE

Pipettes were fabricated as previously described[1] by first pulling a borosilicate glass capillary (0.58 mm I.D. 1 mm O.D.) with a P-97 filament puller (Sutter Instruments, Novato, CA) to a long shank with heat: 715, pull: 0, vel: 150, time: 200. Pipette tips were then scored with another pipette and broken to the desired size (~35 to 45 μm I.D.) and characterized via optical microscopy (800E Nikon, Tokyo, Japan). Silanization of the outside of the pipette by inserting the pipette tips into dichlorosilane solution with a back flow of Ar gas for 90 s was performed before all experiments to improve droplet stability. Pipettes were backfilled with an electrolyte solution using a MicroFil needle (World Precision Instruments, Sarasota, FL) until solution was observed to exit the tip. Quasi-reference counter electrodes (QRCEs) were made by soaking 0.008" Ag wire (A-M Systems, Sequim, WA) in a solution of 1 M $FeCl_3$, 0.1 M HCl overnight. The resultant Ag/AgCl QRCEs were calibrated against a 3 M KCl Ag/AgCl electrode (CHI, Austin, TX) and all potentials reported here are *vs* a 3 M KCl Ag/AgCl.

AMCM Scanning Protocol

A basic scanning method reminiscent of single-barrel pipette SECCM in hopping mode was employed, where the pipette is "hopped" between each electrode of the MEA. The pipette position was first aligned with the array aided by cameras to ensure each landing site would be centered over an electrode. Signal traces of the relative probe-substrate distance (D_{ps}) applied pipette voltage (E_{app}) and working electrode current (i_{WE}) during an approach, meniscus contact, and measurement are shown in **Figure S4**.

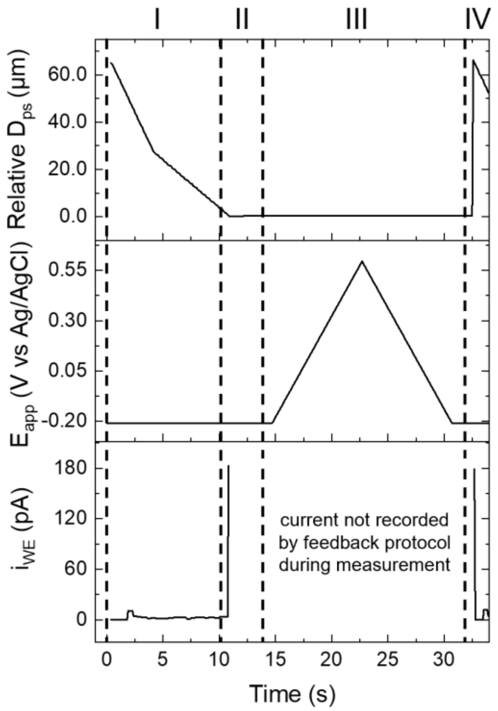

Figure S4. Scanning protocol traces of relative D_{ps} , E_{app}, and i_{WE} during approach and measurement for one electrode/pipette position. Recorded at a 2 µm diameter Au microelectrode, 2 mM FcMeOH, 25 mM KCl. During stage I (indicated at the top of (b)) pipette approaches the array at two approach speeds (first 10 µm/s, then 4 µm/s) while E_{app} = -0.1 V vs Ag/AgCl. At the moment of electrical contact between pipette meniscus and electrode, stage II (indicated by large i_{WE} increase), approach is halted and D_{ps} is established by retracting the pipette 1-2 µm from the surface. After a settling time of 2.5 s the electrochemical measurement is carried out during stage III, in this case a cyclic voltammogram. Current response is recorded and exported independently from the scanning protocol control. Finally, stage IV, the pipette is retracted 65 µm, "hopped" laterally 150 µm to the next electrode and a new approach sequence started.

During stage I of the approach, E_{app} is set to a low potential with respect to the formal potential of the expected reaction. The pipette approache-0s the sample first, at a fast 10 µm/s over a set distance, and the approach is then slowed to 4 µm/s. In stage II the meniscus electrical contact with an electrode of the MEA is made and the resultant current increase signals the approach routine to stop. The pipette is then retracted 1-2 µm to ensure no direct contact between pipette and sample surface. After a 2.5 s "settling time" to ensure droplet stability, the potential program is then applied between pipette QRCE

and working electrode in stage III. The scanning protocol and measurement recording are de-coupled within the software ensuring the current response and x, y, z data is written and exported to a CSV file after each position. If wetting of a recessed electrode does not occur instantaneously, a protocol, dubbed the “insurance mechanism”, is implemented to prevent pipette crash. Finally, in stage IV the pipette retracts 65-85 μm before moving laterally 150 μm to the next electrode position. Scanning continues in a raster formation for a set number of electrode positions.

Pipette Approach Insurance Mechanism: In the event that electrical contact fails at certain electrodes, an insurance mechanism was used as described here to avoid tip crashes that would damage the pipette. The pipette Z-height of the first electrode contact in an array is stored and set as a $D_{ps} = 0$, acting as the assumed array surface. A failsafe distance of typically 5-10 μm is set such that the pipette will not approach past the stored relative $D_{ps} = 0$ plus the failsafe distance value. If the relative D_{ps} of an electrode is lower than the assumed surface position (within a tolerance), then the $D_{ps} = 0$ Z-height will be adjusted. When the insurance mechanism is required, the pipette will retract the set failsafe distance and proceed with the measurment before moving to the next electrode. Typically, less than 6 electrodes out of every 100 require this protocol.

Deep Learning Analysis of Collision Experiments

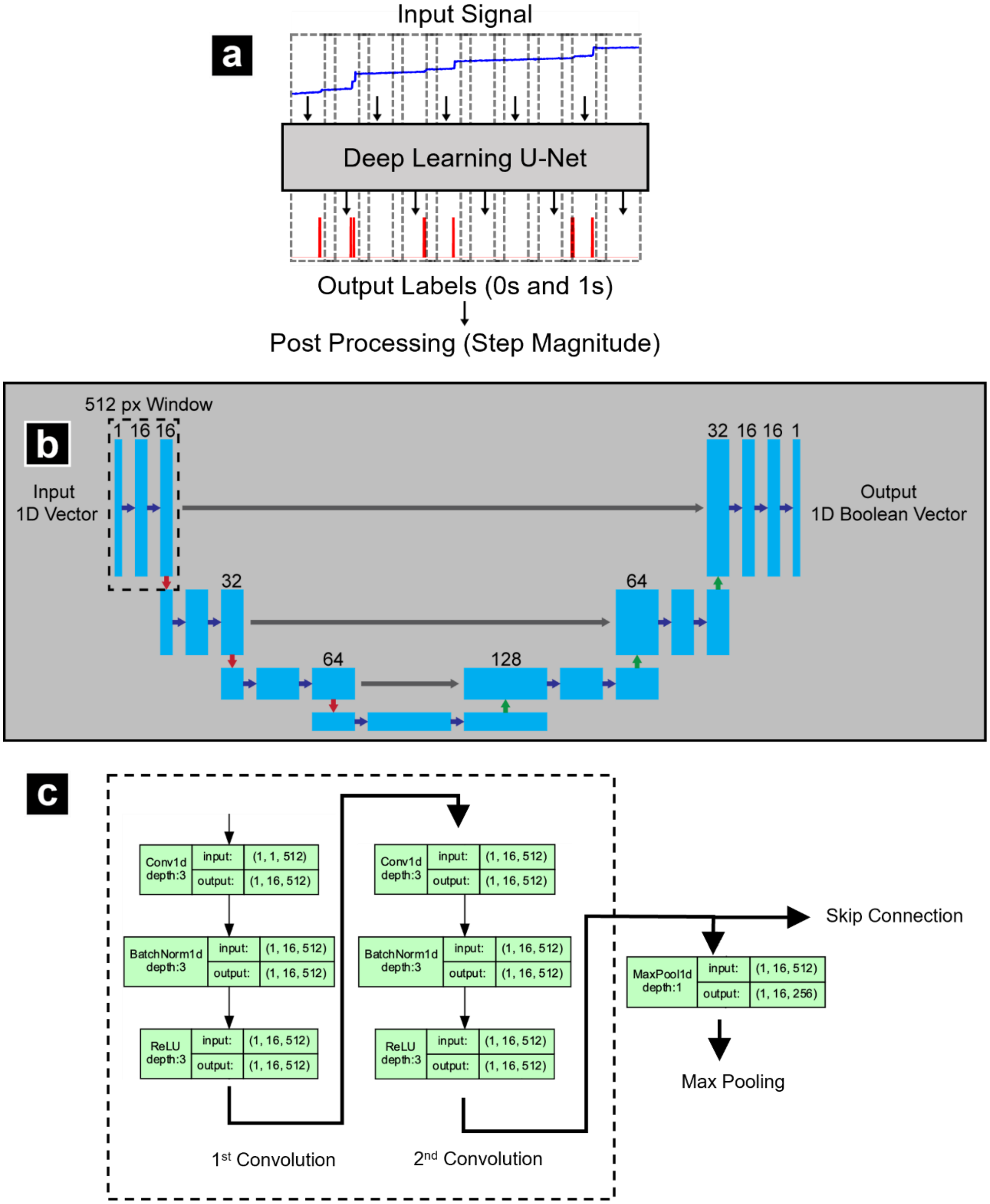

Figure S5. Illustration of deep learning and post processing analysis (a) Dashed boxes represent 512 px width sliding window used in U-Net model. (b) U-Net model architecture and (c) details of the first part of contracting path.

Time-series Collision Events Localization

In order to achieve current signal localization, we adopted and modified U-Net deep learning model created by Ronneberger, et al. by changing the model dimension, model depth and padding strategy.[2] Here, the localization means a positive (1) or negative (0) label is supposed to be assigned to each data point in current time series, to indicate if there is a signal rising stage caused by collision. The U-Net model, shown in **Figure S5b**, consists of one encoder and one decoder. In the encoder pathway, the input current signal vector with 512 data points undergoes 3 times of data compression (double convolution and 1D maxpooling), with each compression followed by a skip connection to the corresponding decoder layer (as is detailed in **Figure S5c**). The decoder decompresses the data from 128 classifiers back to one 512-sized vector while merging the skipped feature from encoder series. The model will output 512 labels, each corresponding to the input signal vector to localize the collision event at a specific time, as shown in **Figure S5a**. Sliding window strategy was used for fitting different sizes of the original signal into the model input.

Model Training: Data Augmentation

Due to the reason that the sample dataset size was limited to train the model, we achieved the data augmentation through algorithm-synthesized signals which has the similar pattern as the experimental signals. The model was pretrained with 500 synthesized signals with 100 epochs and was fine-tuned with hybrid dataset (30 synthesized signals, 30 experimental signals and 30 experimental blanks) with 10 epochs. The model achieved >99.5 % accuracy in capturing all rising stage signals for this collision experiment.

Post Processing: Step-height Extraction

After localization step height were captured by taking the difference between the average of 8 px before and after the rising edge of each event in a post processing step. Averaged pixels were 4 px away from the rising edge before and after each event to avoid the small current overshoot at the end of the step rise. The calculated step heights were further refined by a 1.5 pA threshold, which means any step events smaller than this threshold will be ignored.

All code was based on Python and PyTorch deep learning framework. Codes and models are available at https://github.com/KLDistance/unet_collision_detector. The inferencing is GPU-free.

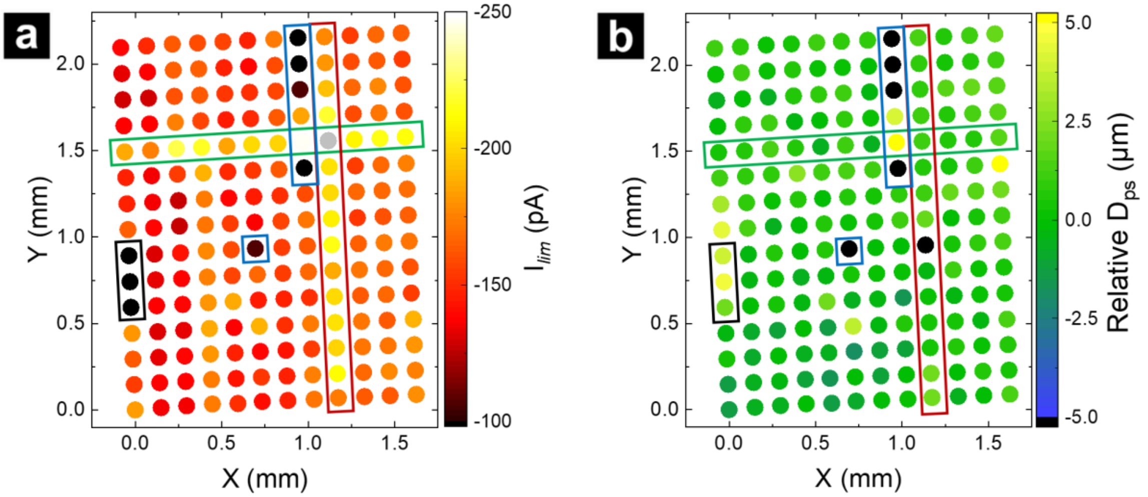

Figure S6. AMCM heat maps of the (a) I_{lim} current extracted at 0.6 V *vs* Ag/AgCl from voltammograms of FcMeOH oxidation and the (b) relative D_{ps} at 500 nm diameter electrodes within a 15 x 12 Pt-NEA. Colored boxes indicate the following and were eliminated from analysis: Black- pipette approach halted early due to noise trigger, Blue- insurance mechanism triggered caused by contaminant at pipette tip (observed in AMCM cameras in real time), Red- contaminate (dust) attached to pipette tip causing droplet stability issues, and Green- lithography error causing double feature (shown in Figure S7).

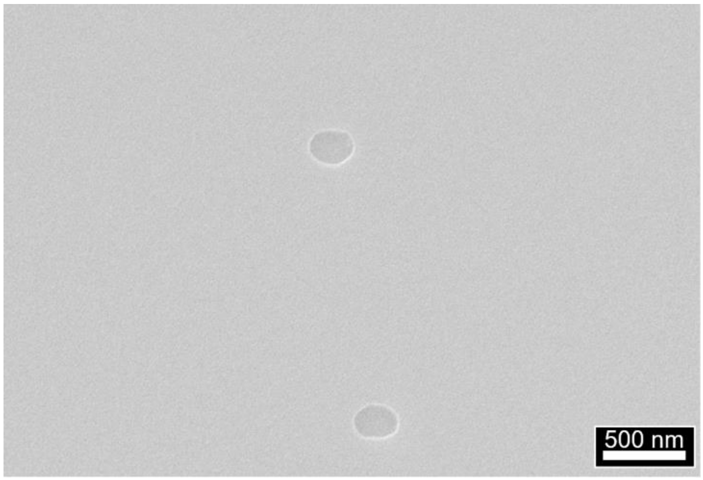

Figure S7. Electron micrograph of double feature found in 11th row from start of scan at 500 nm Pt NEA (see Figure S6).

Finite Element Method (FEM) Simulations

Models: Finite element method (FEM) simulations of AMCM experiments were built using COMSOL Multiphysics v 6.1. The models solved the Nernst-Planck and Navier-Stokes equations[3] to produce time-dependent simulations of mass transport AMCM by diffusion and convection. Two models were produced; a 2-D axisymmetric model was used to study the impact of solvent evaporation at the pipette opening on mass transport in AMCM, and a 3-D model was used to investigate step sizes for the collision experiments and the effect of pipette positioning relative to the electrode. Except where indicated, both models used identical boundary conditions. Solution parameters such as concentrations and diffusion coefficients were set equal to those used in the experiments or literature values as needed. COMSOL model reports showing additional domain and meshing information are provided with this SI.

Geometry and Physics Interfaces: The basic geometry of both models is shown in Figures S8. The model geometries match those from the experiments used for comparison in the main paper, and include the pipette, droplet, and recessions in the substrate for the SiN_X and photoresist insulation, respectively. The meniscus of the droplet

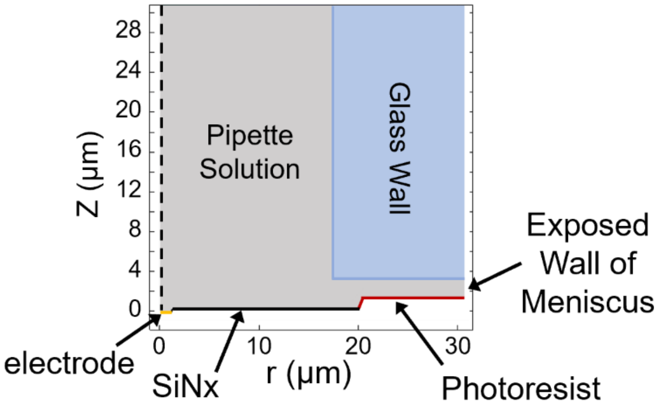

Figure S8. Schematic of labeled FEM geometry.

at the end of the pipette is modelled as a cylinder having a radius equal to the outer radius of the pipette. The probe to substrate distance (D_{ps}) was set to 2 μm for all simulations. Mass transport in simulations of chronoamperometry experiments was simulated using the "Transport of Diluted Species" (TDS) physics interface in COMSOL, while simulations of cyclic voltammetry used the "Electroanalysis" (ELAN) physics interface. Fluid flow was implemented in all models using the "Creeping Flow" (SPF) physics interface with a multiphysics flow coupling.

Boundary Conditions, Nernst-Planck (Mass Transport): The models implemented reversible 1 electron oxidation of FcMeOH at the MEA disk using a flux boundary condition (Butler-Volmer kinetics), and simulated currents for chronoamperograms and CVs were calculated by integrating the normal flux of FcMeOH at the MEA disk. A concentration boundary condition was applied at the back of the pipette to fix all concentrations at their bulk values. A flux boundary condition was applied at the droplet meniscus to model the solute accumulation that occurs during solvent evaporation (see below). All other boundaries used a "no flux" boundary condition.

Boundary Conditions, Navier-Stokes (Fluid Flow): The droplet meniscus used an outflow velocity boundary condition to simulate solvent drying. The back of the pipette used an open boundary (zero normal stress) condition. All other boundaries used a "no slip" boundary condition.

Solvent Evaporation and Selection of v_{dry}: In the AMCM experiments, a small amount of solvent evaporation at the droplet meniscus produced convection in the solution and

accumulation of solutes. Because the AMCM humidity cell kept the evaporation rate low and constant throughout the experiments, it was realistic to model this effect using an outflow boundary condition (fixed velocity) at the droplet meniscus. The flux boundary condition at the meniscus in the Nernst-Planck simulation set the net solute flux (diffusion + convection) to zero, which provided the expected accumulation of solutes resulting from evaporation.[4]

The normal velocity due to drying at the meniscus, v_{dry}, was set at 6–8 μm/s, such that the simulated chronoamperograms and cyclic voltammograms matched those from the experiments. In addition to fitting the experimental results, the values of v_{dry} also showed reasonable agreement with equation 1 below (from reference 4) when compared using analogous simulations generated for an open pipette (no substrate contact) of the same size.

$$v_{dry} = \frac{4D_w^{air}C_{sat}}{\pi\rho_w r_{capillary}}(1 - RH) \quad (1)$$

Where D_w^{air} ≈ 0.242 cm^2/s is the diffusion coefficient of water as a vapor in the gas phase and C_{sat} ≈ 0.017 kg/m^3 is the concentration of water in the gas phase at saturation, $r_{\text{capillary}}$ is the capillary radius, ρ_w = 997.8 kg/m^3 is the density of water, and RH is the relative humidity as a percent. Equation 1 is an estimate of v_{dry} for an open capillary when the concentration is low and contains a non-volatile solute. With a RH of 80%, capillary radius 17.5 μm (which is the same radius as the pipettes used for the main AMCM experiments) equation 1 estimates v_{dry} at 6 μm/s. This indicates that the values used for v_{dry} in the simulations of AMCM are within the expected range.

Accumulation period: In AMCM, a small amount of additional drying occurs between experiments while the pipette is lifted and moved between electrodes in the array. To provide realistic starting concentrations in the droplet, the simulations used a potential–time program where the electrode potential was initially set to −0.3 V vs E^0, (effectively deactivating the electrode for FcMeOH oxidation) for a period of 15 s before being stepped/swept as needed for the simulated CA or CV experiments. The period at −0.3 V vs E^0, allowed FcMeOH to accumulate in the droplet, which effectively adjusted the simulations for the extra drying that occurs between experiments in AMCM.

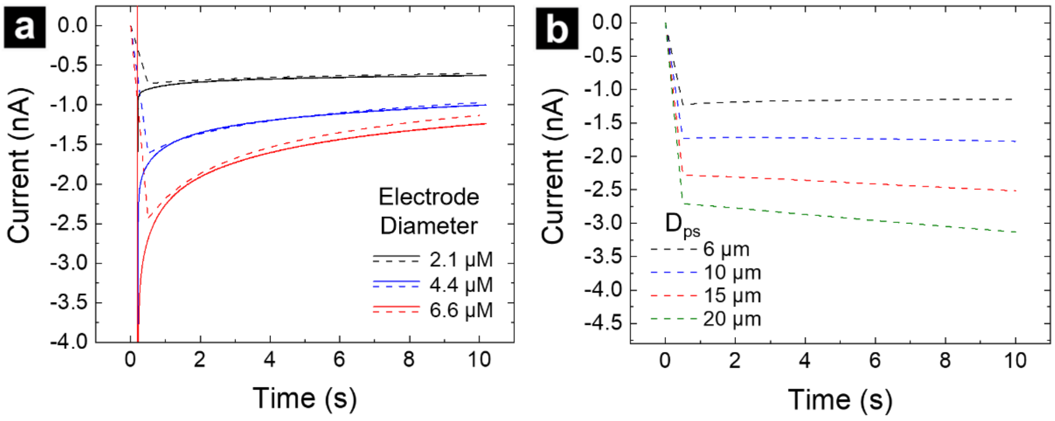

Figure S9. AMCM chronoamperometry of 2 FcMeOH (a) experimental (solid lines) and simulated (dashed lines) at different electrode diameters and (b) simulated chronoamperomogram on a 2.1 μm electrode diameter at different probe-to-substrate distance (D_{ps}) values. Same experimental and simulated parameters as Figure 4.

Expanded Discussion of CV Simulations

The non-zero (reductive) current at the start of CVs collected using automated AMCM (**Figure 3a-d and S10**) occurs because when the pipette is moved to a new disk in the MEA, some $FcMeOH^+$ remains in the pipette from the previous experiment. Even with the high pipette radius to MEA disk radius ratio, the voltammograms collected at 2.1 µm diameter electrodes show some peaking behavior instead of a pure sigmoid shape, due to the confined geometry in the pipette (**Figure S10a**). Simulations of CV in the AMCM geometry with evaporative physics were carried out with v_{dry} = 8 µm/s, and it was

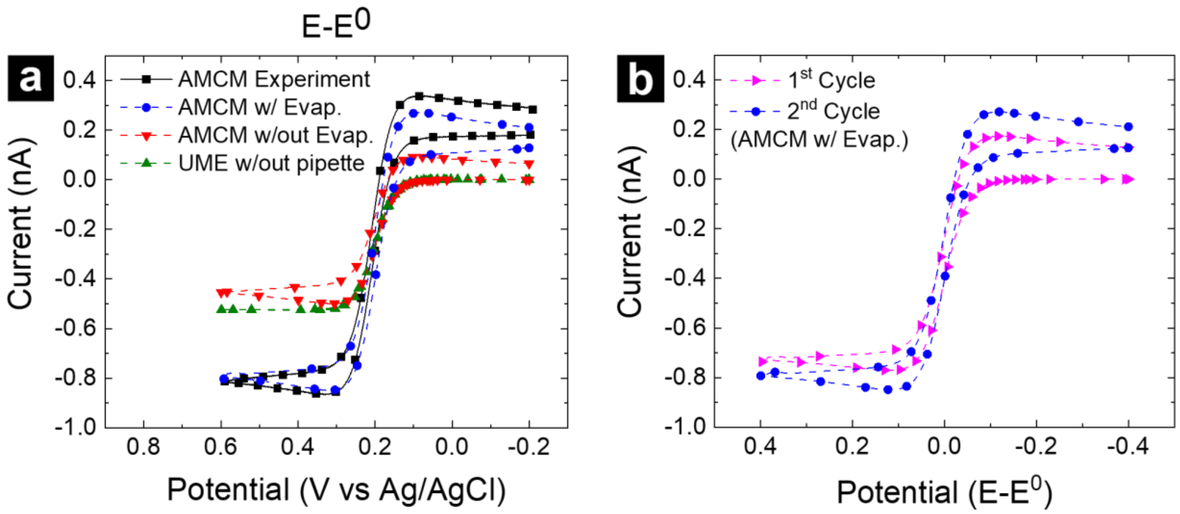

Figure S10. (a) Comparison of averaged AMCM voltammograms (solid line, N=135) at 2.1 µm Au MEA electrodes and the simulated response (dashed lines) with (v_{dry} = 8 µm/s) and without (v_{dry} = 0 µm/s) evaporation physics. (b) Includes both successive simulated cycles of AMCM w/ evaporation physics, the 2nd of which is shown in (a). Pipette (I.D. 35 µm O.D. 61 µm) filled with 2 mM FcMeOH and 25 mM KCl, all voltammograms recorded at 100 mV/s.

found that a second consecutive cycle more closely matched the experimental CV (**Figure S10b** includes both simulated cycles). The simulated 2nd cycle mimics the impact of remaining $FcMeOH^+$ in the pipette in subsequent CV measurements by AMCM, causing a non-zero (reductive) current at the start of the next CV (**Figure 3a-d and S10a**). The automated AMCM procedure is able to partly mitigate this effect, because most of

the droplet adheres to the substrate and is left behind when the pipette is moved to a new position. We postulate that this normalizes the concentration within the droplet at each electrode, leading to the low variation in the FcMeOH oxidation current across the array (-0.81 ± 0.04 nA).

Simulation of Particle Collision Step Heights

To produce simulated current step heights for collision experiments, a 3-D version of the model was used which placed a spherical blocking particle on the electrode as a new domain in the simulation. The step height was assessed by solving the model twice using the same mesh; once with the blocking sphere surface normal flux set to zero for all species, and once with the sphere domain set as part of the electrolyte solution. The simulated current step values (I_{step}) were calculated using the expression,

$$\mathrm{I}_{step} = \mathrm{I}_{block} - \mathrm{I}_{open}$$

where I_{block} is the current evaluated at t = 3 s with the sphere surface normal flux set to zero (blocking), and I_{open} is the current evaluated at t = 3 s with the sphere domain set as part of the solution.

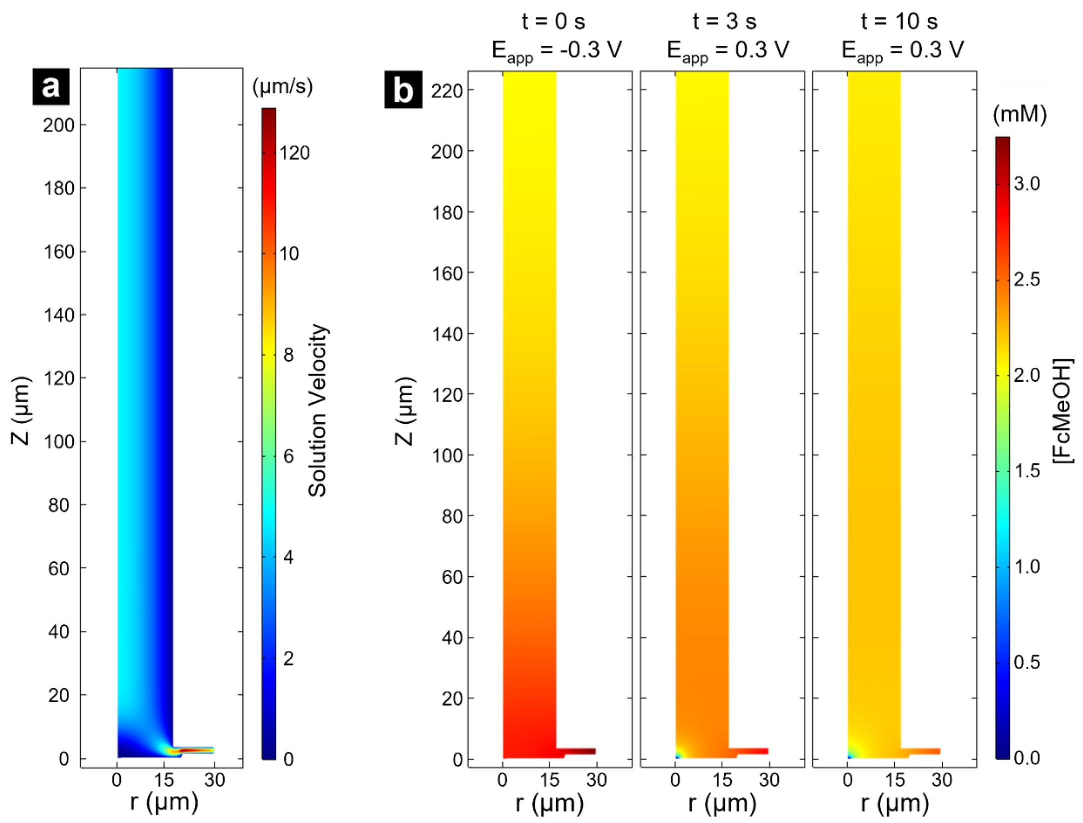

Figure S11. Simulated velocity magnitude and concentration profiles of AMCM pipette, meniscus and electrode during chronoamperometry. Profiles of (a) solution velocity and (b) FcMeOH concentration at t = 0 s (after the accumulation period but before oxidative potential is applied), t = 3 s and t = 10 s. Electrode is 2.1 µm in diameter and initial FcMeOH concentration was 2 mM. Magnified profiles with flux arrows are included in Figure S12.

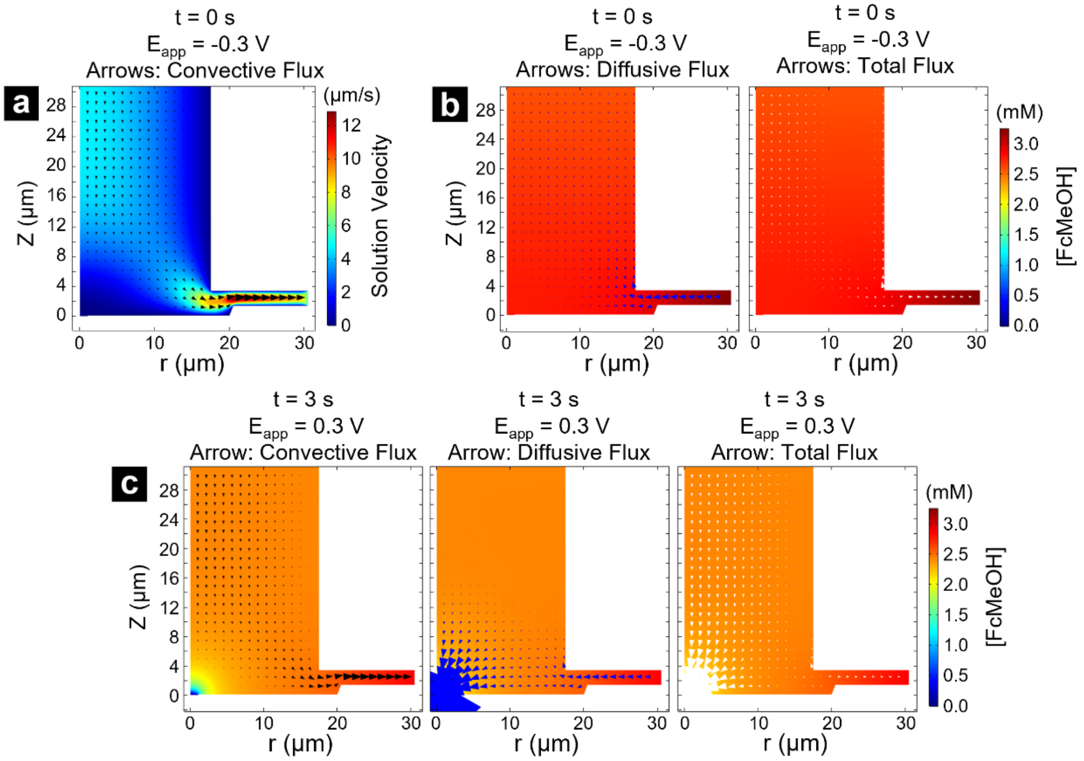

Figure S12. Simulated velocity magnitude and concentration profiles and FcMeOH flux arrows of AMCM pipette tip and meniscus during chronoamperometry of (a) solution velocity and (b) concentration at t = 0 s after the accumulation period but before an oxidative potential was applied. (c) Concentration profile at t = 3 s at E_{app} = 0.3 V vs E^0. Arrows are directly proportional to the convective (black), diffusive (blue) and total (white) FcMeOH flux. Electrode is 2.1 µm in diameter and initial FcMeOH concentration was 2 mM.

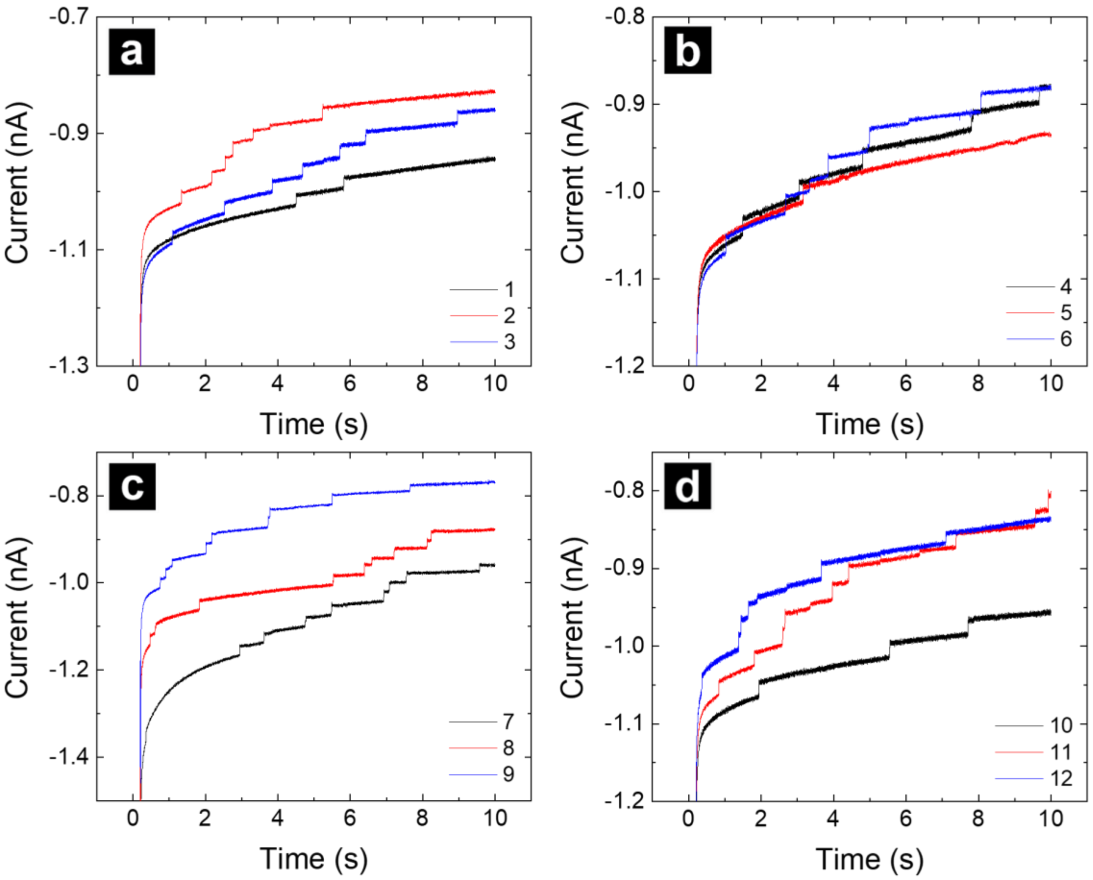

Figure S13. Chronoamperomograms from twelve 2.1 μm diameter Au electrodes where voltage was held at 0.4 V vs Ag/AgCl for 10 seconds. Pipette contained 500 nm polystyrene beads in 2 mM FcMeOH and 0.7 mM KCl.

References

1. Alden, S. E.; Siepser, N. P.; Patterson, J. A.; Jagdale, G. S.; Choi, M.; Baker, L. A., Array Microcell Method (AMCM) for Serial Electroanalysis. *ChemElectroChem* **2020,** *7*, 1084-1091, 10.1002/celc.201901976

2. Ronneberger, O.; Fischer, P.; Brox, T. In *U-Net: Convolutional Networks for Biomedical Image Segmentation*, Medical Image Computing and Computer-Assisted Intervention – MICCAI 2015, Cham, 2015//; Navab, N.; Hornegger, J.; Wells, W. M.; Frangi, A. F., Eds. Springer International Publishing: Cham, 2015; pp 234-241.

3. Bard, A. J.; Faulkner, L. R.; White, H. S., *Electrochemical Methods: Fundamentals and Applications*. 3rd ed.; John Wiley & Sons, Inc.: Hoboken, NJ, 2022; pp. 412–413.

4. Bacchin, P.; Leng, J.; Salmon, J.-B., Microfluidic Evaporation, Pervaporation, and Osmosis: From Passive Pumping to Solute Concentration. *Chem. Rev.* **2022,** *122*, 6938-6985, 10.1021/acs.chemrev.1c00459
