# Supplementary material for: High-Throughput Single-Entity Electrochemistry with Microelectrode Arrays
Source: Anal Chem. 2024 May 23;96(22):9177–84. doi: 10.1021/acs.analchem.4c01092 (PMC11154736; doi:10.1021/acs.analchem.4c01092)
Supplement: Supplementary file 2 — ac4c01092_si_002.pdf [file ac4c01092_si_002.pdf]

AMCM 2D Chronoamperometry with evaporation

Report date	Dec 20, 2023, 2:13:36 PM

Contents

1. Global Definitions 3
1.1. Parameters 3
1.2. Shared Properties 4
2. Component 1 5
2.1. Definitions 5
2.2. Geometry 1 5
2.3. Transport of Diluted Species 7
2.4. Creeping Flow 25
2.5. Events 49
2.6. Multiphysics 51
2.7. Mesh 1 52
3. Study 1 60
3.1. Time Dependent 60
3.2. Solver Configurations 60
4. Results 69
4.1. Data Sets 69
4.2. Plot Groups 76
4.3. Evaluation Groups 77

1 Global Definitions

Date	Dec 20, 2023, 1:55:59 PM

GLOBAL SETTINGS

Name	ST 2023-04-06 001 AMCM2D report.mph
Path	C:\COMSOL Projects\2022_AMCM\ST_2023-04-06_001_AMCM2D_report.mph
Version	COMSOL Multiphysics 6.1 (Build: 357)

USED PRODUCTS

COMSOL Multiphysics
Electrochemistry Module

COMPUTER INFORMATION

CPU	Intel64 Family 6 Model 60 Stepping 3, 4 cores, 31.88 GB RAM
Operating system	Windows 10

1.1 PARAMETERS

PARAMETERS 1

Name	Expression	Value	Description
re	1.05E-6 [m]	1.05E−6 m	UME radius
de	1.3E-6 [m]	1.3E−6 m	photoresist recession depth
rpipet	(35E-6 [m])/2	1.75E−5 m	inner radius of pipet
hpipet	2E-6 [m]	2E−6 m	Dps
taper	0.001 [deg]	1.7453E−5 rad	pipet taper angle
pipetshank	1E-3 [m]	0.001 m	length of pipet simulated
pipetOR	(61 [um])/2	3.05E−5 m	outer radius of pipet (w/ wall)
recOR	photo + 5E-7 [m]	2.05E−5 m	outer radius of recession
E	0.3 [V]	0.3 V	electrode potential
Ef	0 [V]	0 V	formal potential
cRbulk	0.002 [M]	2 mol/m^3	bulk conc of R
F	9.64853E4 [C/mol]	96485 C/mol	Farday constant
f	38.92 [1/V]	38.92 1/V	F/RT
DR	7.4E-6 [(cm^2)/s]	7.4E−10 m^2/s	diffusion coefficient of R
DO	DR	7.4E−10 m^2/s	diff co of O
a	0.5	0.5	alpha
k0	10 [cm/s]	0.1 m/s	e-t rate constant
photo	20 [um]	2E−5 m	photoresist hole radius

Name	Expression	Value	Description
SiNx	0.09 [um]	9E−8 m	SiNx hole depth
vdry	6E-6 [m/s]	6E−6 m/s	drying outlet velocity
t_acc	15 [s]	15 s	accumulation time
t_step	10 [s]	10 s	i-t curve length

1.2 SHARED PROPERTIES

1.2.1 Default Model Inputs

Tag	cminpt

2 Component 1

SETTINGS

Description	Value
Unit system	Same as global system (SI)
Avoid inverted elements by curving interior domain elements	Off

2.1 DEFINITIONS

2.1.1 Coordinate Systems

Boundary System 1

Coordinate system type	Boundary system
Tag	sys1

COORDINATE NAMES

First	Second	Third
t1	to	n

2.2 GEOMETRY 1

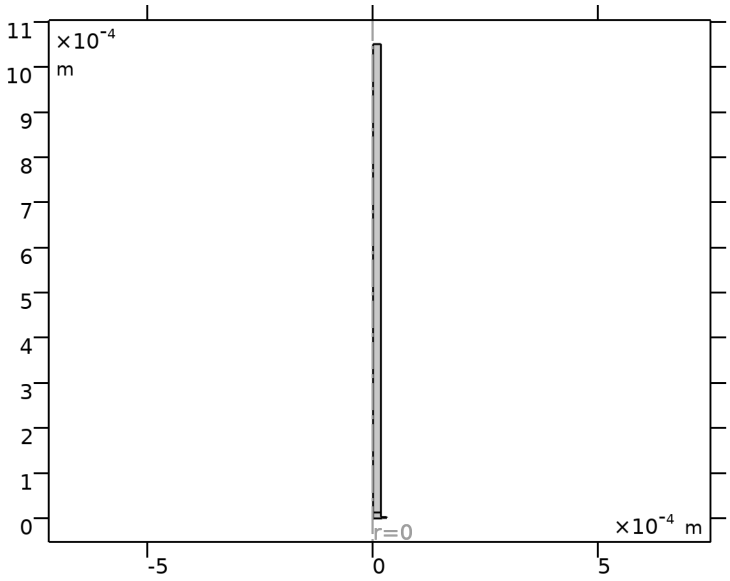

Geometry 1

UNITS

Length unit	m
Angular unit	deg

GEOMETRY STATISTICS

Description	Value
Space dimension	2
Number of domains	4
Number of boundaries	18
Number of vertices	15

2.2.1 Polygon 1 (pol1)

OBJECT TYPE

Description	Value
Type	Solid

COORDINATES

Description	Value
Data source	Table

COORDINATES

r (m)	z (m)
0	0
re	0
re	SiNx
photo	SiNx
recOR	SiNx+de
pipetOR	SiNx+de
pipetOR	SiNx+de+hpipet
rpipet	SiNx+de+hpipet
rpipet	de+hpipet+pipetshank+(50E-6[m]-hpipet)+SiNx
0	de+hpipet+pipetshank+(50E-6[m]-hpipet)+SiNx

2.2.2 Circle 1 (c1)

POSITION

Description	Value
Position	{0, 0}

SIZE AND SHAPE

Description	Value
Radius	re*1.5
Sector angle	90

2.2.3 Line Segment 1 (ls1)

SETTINGS

Description	Value
Specify	Coordinates
Coordinates	{0, SiNx + de+hpipet + 10[um]}
Specify	Coordinates
Coordinates	{rpipet, SiNx + de+hpipet + 10[um]}

2.3 TRANSPORT OF DILUTED SPECIES

USED PRODUCTS

COMSOL Multiphysics
Electrochemistry Module

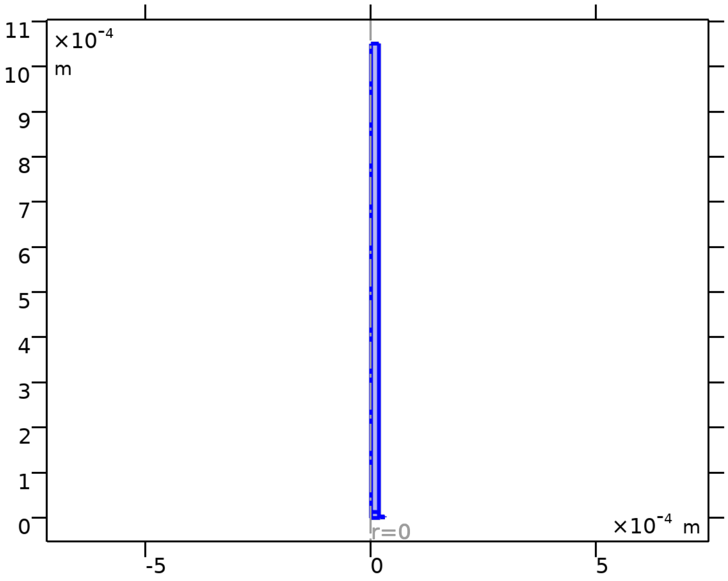

Transport of Diluted Species

SELECTION

Geometric entity level	Domain
Selection	Geometry geom1: Dimension 2: Domains 1–3

EQUATIONS

$$\frac{\partial c_i}{\partial t} + \nabla \cdot \mathbf{J}_i + \mathbf{u} \cdot \nabla c_i = R_i$$

$$\mathbf{J}_i = -D_i \nabla c_i$$

2.3.1 Interface Settings

Discretization

SETTINGS

Description	Value
Concentration	Linear

SETTINGS

Description	Value
Equation form	Study controlled

Species Activity

SETTINGS

Description	Value
Species activity	Ideal

Transport Mechanisms

SETTINGS

Description	Value
Convection	On
Migration in electric field	Off
Mass transfer in porous media	Off

2.3.2 Variables

Name	Expression	Unit	Description	Selection	Details
tds.d	1	1	Out-of-plane geometry extension	Global	
tds.f_cR	1	1	Activity coefficient	Domains 1–3	
tds.f_cO	1	1	Activity coefficient	Domains 1–3	
tds.nr	nr	1	Normal vector, r-component	Boundaries 5, 17	
tds.nphi	0	1	Normal vector, phi-component	Boundaries 5, 17	
tds.nz	nz	1	Normal vector, z-component	Boundaries 5, 17	
tds.nr	dnr	1	Normal vector, r-component	Boundaries 1–4, 6–7, 9–16	
tds.nphi	0	1	Normal vector, phi-component	Boundaries 1–4, 6–7, 9–16	

Name	Expression	Unit	Description	Selection	Details
tds.nz	dnz	1	Normal vector, z-component	Boundaries 1–4, 6–7, 9–16	
tds.nrmesh	nrmesh	1	Normal vector (mesh), r-component	Boundaries 5, 17	
tds.nphimesh	0	1	Normal vector (mesh), phi-component	Boundaries 5, 17	
tds.nzmesh	nzmesh	1	Normal vector (mesh), z-component	Boundaries 5, 17	
tds.nrmesh	dnrmesh	1	Normal vector (mesh), r-component	Boundaries 1–4, 6–7, 9–16	
tds.nphimesh	0	1	Normal vector (mesh), phi-component	Boundaries 1–4, 6–7, 9–16	
tds.nzmesh	dnzmesh	1	Normal vector (mesh), z-component	Boundaries 1–4, 6–7, 9–16	
tds.nrc	root.nrc/tds.ncLen	1	Normal vector, r-component	Boundaries 1–7, 9–17	
tds.nphic	0	1	Normal vector, phi-component	Boundaries 1–7, 9–17	
tds.nzc	root.nzc/tds.ncLen	1	Normal vector, z-component	Boundaries 1–7, 9–17	
tds.ncLen	sqrt(root.nrc^2+root.nzc^2+eps)	1	Help variable	Boundaries 1–7, 9–17	
tds.cbf_cR	0	mol/(m²·s)	Convective boundary flux	Boundaries 1–7, 9–17	
tds.u	0	m/s	Velocity field, r-component	Domains 1–3	
tds.v	0	m/s	Velocity field, phi-component	Domains 1–3	
tds.w	0	m/s	Velocity field, z-component	Domains 1–3	
tds.cbf_cO	0	mol/(m²·s)	Convective boundary flux	Boundaries 1–7, 9–17	
tds.R_cR	0	mol/(m³·s)	Total rate expression	Domains 1–3	+ operation
tds.cP_cR	0	mol/kg	Concentration species adsorbed	Domains 1–3	+ operation

Name	Expression	Unit	Description	Selection	Details
			to the solid		
tds.cP_cR	0	mol/kg	Concentration species adsorbed to the solid	Boundaries 1–7, 9–17	+ operation
tds.KP_cR	0	m^3/kg	Adsorption isotherm, first concentration derivative	Domains 1–3	+ operation
tds.KP_cR	0	m^3/kg	Adsorption isotherm, first concentration derivative	Boundaries 1–7, 9–17	+ operation
tds.Rads_cR	0	mol/(m^3·s)	Total adsorption rate	Domains 1–3	+ operation
tds.DiT_cR	0	m^2/s	Turbulent diffusivity	Domains 1–3	
tds.cVar_cR	cR	mol/m^3	Species	Boundaries 1–7, 9–17	
tds.cVar_cR	cR	mol/m^3	Species	Points 1–7, 9–15	
tds.R_cO	0	mol/(m^3·s)	Total rate expression	Domains 1–3	+ operation
tds.cP_cO	0	mol/kg	Concentration species adsorbed to the solid	Domains 1–3	+ operation
tds.cP_cO	0	mol/kg	Concentration species adsorbed to the solid	Boundaries 1–7, 9–17	+ operation
tds.KP_cO	0	m^3/kg	Adsorption isotherm, first concentration derivative	Domains 1–3	+ operation
tds.KP_cO	0	m^3/kg	Adsorption isotherm, first concentration derivative	Boundaries 1–7, 9–17	+ operation
tds.Rads_cO	0	mol/(m^3·s)	Total adsorption rate	Domains 1–3	+ operation
tds.DiT_cO	0	m^2/s	Turbulent diffusivity	Domains 1–3	
tds.cVar_cO	cO	mol/m^3	Species	Boundaries 1–7, 9–17	

Name	Expression	Unit	Description	Selection	Details
tds.cVar_cO	cO	mol/m³	Species	Points 1–7, 9–15	
tds.poro	1	1	Porosity	Domains 1–3	
tds.theta_g	0	1	Gas volume fraction	Domains 1–3	
tds.theta_l	1	1	Liquid volume fraction	Domains 1–3	
tds.theta	tds.poro	1	Mobile fluid volume fraction	Domains 1–3	

2.3.3 Transport Properties 1

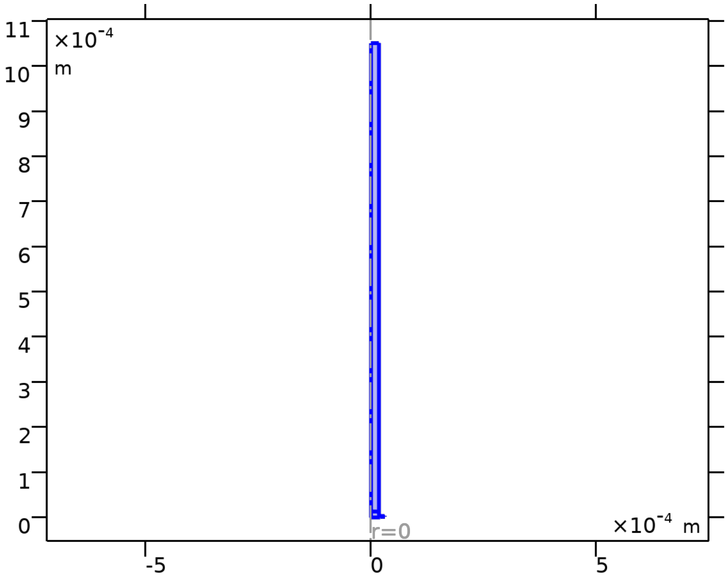

Transport Properties 1

SELECTION

Geometric entity level	Domain
Selection	Geometry geom1: Dimension 2: All domains

EQUATIONS

$$\nabla \cdot \mathbf{J}_i + \mathbf{u} \cdot \nabla c_i = R_i$$

$$\mathbf{J}_i = -D_i \nabla c_i$$

Diffusion

SETTINGS

Description	Value	Unit
Source	Material	

Description	Value	Unit
Material	None	
Diffusion coefficient	User defined	
Diffusion coefficient	DR	m²/s
Diffusion coefficient	User defined	
Diffusion coefficient	DO	m²/s

Coordinate System Selection

SETTINGS

Description	Value
Coordinate system	Global coordinate system

Model Input

SETTINGS

Description	Value
Temperature	Common model input

Variables

Name	Expression	Unit	Description	Selection	Details
domflux.cRr	2*tds.dflux_cRr*pi*r*tds.d	mol/(m·s)	Domain flux, r-component	Domains 1–3	
domflux.cRz	2*tds.dflux_cRz*pi*r*tds.d	mol/(m·s)	Domain flux, z-component	Domains 1–3	
domflux.cOr	2*tds.dflux_cOr*pi*r*tds.d	mol/(m·s)	Domain flux, r-component	Domains 1–3	
domflux.cOz	2*tds.dflux_cOz*pi*r*tds.d	mol/(m·s)	Domain flux, z-component	Domains 1–3	
tds.ndflux_cR	tds.bndFlux_cR	mol/(m²·s)	Normal diffusive flux	Boundaries 2, 5–7, 9–17	
tds.ncflux_cR	tds.cflux_cRr*tds.nrc+tds.cflux_cRphi*tds.nphic+tds.cflux_cRz*tds.nzc	mol/(m²·s)	Normal convective flux	Boundaries 2, 5–7, 9–17	
tds.ntflux_cR	tds.bndFlux_cR+tds.cflux_cRr*tds.nrc+tds.cflux_cRphi*tds.nphic+tds.cflux_cRz*tds.nzc	mol/(m²·s)	Normal total flux	Boundaries 2, 5–7, 9–17	
tds.ndflux_cO	tds.bndFlux_cO	mol/(m²·s)	Normal diffusive flux	Boundaries 2, 5–7, 9–17	
tds.ncflux_cO	tds.cflux_cOr*tds.nr	mol/(m²·s)	Normal	Boundaries 2,	

Name	Expression	Unit	Description	Selection	Details
	c+tds.cflux_cOphi*tds.nphic+tds.cflux_cOz*tds.nzc		convective flux	5–7, 9–17	
tds.ntflux_cO	tds.bndFlux_cO+tds.cflux_cOr*tds.nrc+tds.cflux_cOphi*tds.nphic+tds.cflux_cOz*tds.nzc	mol/(m²·s)	Normal total flux	Boundaries 2, 5–7, 9–17	
tds.u	model.input.u1	m/s	Velocity field, r-component	Domains 1–3	Meta
tds.v	model.input.u2	m/s	Velocity field, phi-component	Domains 1–3	Meta
tds.w	model.input.u3	m/s	Velocity field, z-component	Domains 1–3	Meta
tds.bndFlux_cR	0.25*(uflux_spatial(cR)-dflux_spatial(cR))/(pi*r*tds.d)	mol/(m²·s)	Boundary flux	Boundaries 5, 17	Meta
tds.bndFlux_cR	-dflux_spatial(cR)/tds.d	mol/(m²·s)	Boundary flux	Boundaries 1, 3–4	
tds.bndFlux_cR	-0.5*dflux_spatial(cR)/(pi*r*tds.d)	mol/(m²·s)	Boundary flux	Boundaries 2, 6–7, 9–16	Meta
tds.bndFlux_cO	0.25*(uflux_spatial(cO)-dflux_spatial(cO))/(pi*r*tds.d)	mol/(m²·s)	Boundary flux	Boundaries 5, 17	Meta
tds.bndFlux_cO	-dflux_spatial(cO)/tds.d	mol/(m²·s)	Boundary flux	Boundaries 1, 3–4	
tds.bndFlux_cO	-0.5*dflux_spatial(cO)/(pi*r*tds.d)	mol/(m²·s)	Boundary flux	Boundaries 2, 6–7, 9–16	Meta
tds.DF_cRrr	DR	m²/s	Fluid diffusion coefficient, rr-component	Domains 1–3	
tds.DF_cRphir	0	m²/s	Fluid diffusion coefficient, phir-component	Domains 1–3	
tds.DF_cRzr	0	m²/s	Fluid diffusion coefficient, zr-component	Domains 1–3	

Name	Expression	Unit	Description	Selection	Details
tds.DF_cRrphi	0	m^2/s	Fluid diffusion coefficient, rphi-component	Domains 1–3	
tds.DF_cRphiphi	DR	m^2/s	Fluid diffusion coefficient, phiphi-component	Domains 1–3	
tds.DF_cRzphi	0	m^2/s	Fluid diffusion coefficient, zphi-component	Domains 1–3	
tds.DF_cRrz	0	m^2/s	Fluid diffusion coefficient, rz-component	Domains 1–3	
tds.DF_cRphiz	0	m^2/s	Fluid diffusion coefficient, phiz-component	Domains 1–3	
tds.DF_cRzz	DR	m^2/s	Fluid diffusion coefficient, zz-component	Domains 1–3	
tds.D_cRrr	tds.DF_cRrr+tds.DiT_cR	m^2/s	Diffusion coefficient, rr-component	Domains 1–3	+ operation
tds.D_cRphir	tds.DF_cRphir	m^2/s	Diffusion coefficient, phir-component	Domains 1–3	+ operation
tds.D_cRzr	tds.DF_cRzr	m^2/s	Diffusion coefficient, zr-component	Domains 1–3	+ operation
tds.D_cRrphi	tds.DF_cRrphi	m^2/s	Diffusion coefficient, rphi-component	Domains 1–3	+ operation
tds.D_cRphiphi	tds.DF_cRphiphi+tds.DiT_cR	m^2/s	Diffusion coefficient, phiphi-component	Domains 1–3	+ operation
tds.D_cRzphi	tds.DF_cRzphi	m^2/s	Diffusion coefficient, zphi-component	Domains 1–3	+ operation
tds.D_cRrz	tds.DF_cRrz	m^2/s	Diffusion coefficient, rz-component	Domains 1–3	+ operation
tds.D_cRphiz	tds.DF_cRphiz	m^2/s	Diffusion coefficient, phiz-	Domains 1–3	+ operation

Name	Expression	Unit	Description	Selection	Details
			component		
tds.D_cRzz	tds.DF_cRzz+tds.DiT_cR	m^2/s	Diffusion coefficient, zz-component	Domains 1–3	+ operation
tds.DF_cOrr	DO	m^2/s	Fluid diffusion coefficient, rr-component	Domains 1–3	
tds.DF_cOphir	0	m^2/s	Fluid diffusion coefficient, phir-component	Domains 1–3	
tds.DF_cOzr	0	m^2/s	Fluid diffusion coefficient, zr-component	Domains 1–3	
tds.DF_cOrphi	0	m^2/s	Fluid diffusion coefficient, rphi-component	Domains 1–3	
tds.DF_cOphiphi	DO	m^2/s	Fluid diffusion coefficient, phiphi-component	Domains 1–3	
tds.DF_cOzphi	0	m^2/s	Fluid diffusion coefficient, zphi-component	Domains 1–3	
tds.DF_cOrz	0	m^2/s	Fluid diffusion coefficient, rz-component	Domains 1–3	
tds.DF_cOphiz	0	m^2/s	Fluid diffusion coefficient, phiz-component	Domains 1–3	
tds.DF_cOzz	DO	m^2/s	Fluid diffusion coefficient, zz-component	Domains 1–3	
tds.D_cOrr	tds.DF_cOrr+tds.DiT_cO	m^2/s	Diffusion coefficient, rr-component	Domains 1–3	+ operation
tds.D_cOphir	tds.DF_cOphir	m^2/s	Diffusion coefficient, phir-component	Domains 1–3	+ operation
tds.D_cOzr	tds.DF_cOzr	m^2/s	Diffusion coefficient, zr-component	Domains 1–3	+ operation
tds.D_cOrphi	tds.DF_cOrphi	m^2/s	Diffusion coefficient, rphi-	Domains 1–3	+ operation

Name	Expression	Unit	Description	Selection	Details
			component		
tds.D_cOphiphi	tds.DF_cOphiphi+tds.DiT_cO	m^2/s	Diffusion coefficient, phiphi-component	Domains 1–3	+ operation
tds.D_cOzphi	tds.DF_cOzphi	m^2/s	Diffusion coefficient, zphi-component	Domains 1–3	+ operation
tds.D_cOrz	tds.DF_cOrz	m^2/s	Diffusion coefficient, rz-component	Domains 1–3	+ operation
tds.D_cOphiz	tds.DF_cOphiz	m^2/s	Diffusion coefficient, phiz-component	Domains 1–3	+ operation
tds.D_cOzz	tds.DF_cOzz+tds.DiT_cO	m^2/s	Diffusion coefficient, zz-component	Domains 1–3	+ operation
tds.Dav_cR	0.5*(tds.D_cRrr+tds.D_cRzz)	m^2/s	Average diffusion coefficient	Domains 1–3	
tds.Dav_cO	0.5*(tds.D_cOrr+tds.D_cOzz)	m^2/s	Average diffusion coefficient	Domains 1–3	
tds.tflux_cRr	tds.dflux_cRr+tds.cflux_cRr	$mol/(m^2\cdot s)$	Total flux, r-component	Domains 1–3	+ operation
tds.tflux_cRphi	tds.dflux_cRphi+tds.cflux_cRphi	$mol/(m^2\cdot s)$	Total flux, phi-component	Domains 1–3	+ operation
tds.tflux_cRz	tds.dflux_cRz+tds.cflux_cRz	$mol/(m^2\cdot s)$	Total flux, z-component	Domains 1–3	+ operation
tds.dfluxMag_cR	sqrt(tds.dflux_cRr^2+tds.dflux_cRphi^2+tds.dflux_cRz^2)	$mol/(m^2\cdot s)$	Diffusive flux magnitude	Domains 1–3	
tds.tfluxMag_cR	sqrt(tds.tflux_cRr^2+tds.tflux_cRphi^2+tds.tflux_cRz^2)	$mol/(m^2\cdot s)$	Total flux magnitude	Domains 1–3	
tds.dpflux_cRr	0	$mol/(m^2\cdot s)$	Dispersive flux, r-component	Domains 1–3	
tds.dpflux_cRphi	0	$mol/(m^2\cdot s)$	Dispersive flux, phi-component	Domains 1–3	
tds.dpflux_cRz	0	$mol/(m^2\cdot s)$	Dispersive flux, z-component	Domains 1–3	
tds.tflux_cOr	tds.dflux_cOr+tds.cflux_cOr	$mol/(m^2\cdot s)$	Total flux, r-component	Domains 1–3	+ operation
tds.tflux_cOphi	tds.dflux_cOphi+tds	$mol/(m^2\cdot s)$	Total flux, phi-	Domains 1–3	+ operation

Name	Expression	Unit	Description	Selection	Details
	.cflux_cOphi		component		
tds.tflux_cOz	tds.dflux_cOz+tds.cflux_cOz	mol/(m²·s)	Total flux, z-component	Domains 1–3	+ operation
tds.dfluxMag_cO	sqrt(tds.dflux_cOr^2+tds.dflux_cOphi^2+tds.dflux_cOz^2)	mol/(m²·s)	Diffusive flux magnitude	Domains 1–3	
tds.tfluxMag_cO	sqrt(tds.tflux_cOr^2+tds.tflux_cOphi^2+tds.tflux_cOz^2)	mol/(m²·s)	Total flux magnitude	Domains 1–3	
tds.dpflux_cOr	0	mol/(m²·s)	Dispersive flux, r-component	Domains 1–3	
tds.dpflux_cOphi	0	mol/(m²·s)	Dispersive flux, phi-component	Domains 1–3	
tds.dpflux_cOz	0	mol/(m²·s)	Dispersive flux, z-component	Domains 1–3	
tds.dflux_cRr	-tds.D_cRrr*cRr-tds.D_cRrz*cRz	mol/(m²·s)	Diffusive flux, r-component	Domains 1–3	+ operation
tds.dflux_cRphi	-tds.D_cRphir*cRr-tds.D_cRphiz*cRz	mol/(m²·s)	Diffusive flux, phi-component	Domains 1–3	+ operation
tds.dflux_cRz	-tds.D_cRzr*cRr-tds.D_cRzz*cRz	mol/(m²·s)	Diffusive flux, z-component	Domains 1–3	+ operation
tds.grad_cRr	cRr	mol/m⁴	Concentration gradient, r-component	Domains 1–3	
tds.grad_cRphi	0	mol/m⁴	Concentration gradient, phi-component	Domains 1–3	
tds.grad_cRz	cRz	mol/m⁴	Concentration gradient, z-component	Domains 1–3	
tds.dflux_cOr	-tds.D_cOrr*cOr-tds.D_cOrz*cOz	mol/(m²·s)	Diffusive flux, r-component	Domains 1–3	+ operation
tds.dflux_cOphi	-tds.D_cOphir*cOr-tds.D_cOphiz*cOz	mol/(m²·s)	Diffusive flux, phi-component	Domains 1–3	+ operation
tds.dflux_cOz	-tds.D_cOzr*cOr-tds.D_cOzz*cOz	mol/(m²·s)	Diffusive flux, z-component	Domains 1–3	+ operation
tds.grad_cOr	cOr	mol/m⁴	Concentration gradient, r-component	Domains 1–3	
tds.grad_cOphi	0	mol/m⁴	Concentration gradient, phi-component	Domains 1–3	

Name	Expression	Unit	Description	Selection	Details
tds.grad_cOz	cOz	mol/m⁴	Concentration gradient, z-component	Domains 1–3	
tds.cflux_cRr	cR*tds.u	mol/(m²·s)	Convective flux, r-component	Domains 1–3	
tds.cflux_cRphi	cR*tds.v	mol/(m²·s)	Convective flux, phi-component	Domains 1–3	
tds.cflux_cRz	cR*tds.w	mol/(m²·s)	Convective flux, z-component	Domains 1–3	
tds.cfluxMag_cR	sqrt(tds.cflux_cRr^2 +tds.cflux_cRphi^2 +tds.cflux_cRz^2)	mol/(m²·s)	Convective flux magnitude	Domains 1–3	
tds.cflux_cOr	cO*tds.u	mol/(m²·s)	Convective flux, r-component	Domains 1–3	
tds.cflux_cOphi	cO*tds.v	mol/(m²·s)	Convective flux, phi-component	Domains 1–3	
tds.cflux_cOz	cO*tds.w	mol/(m²·s)	Convective flux, z-component	Domains 1–3	
tds.cfluxMag_cO	sqrt(tds.cflux_cOr^2 +tds.cflux_cOphi^2 +tds.cflux_cOz^2)	mol/(m²·s)	Convective flux magnitude	Domains 1–3	
tds.Rlin_cR	0	1/s	Linear source term coefficient	Domains 1–3	+ operation
tds.Res_cR	tds.u*cRr+tds.w*cRz -cR*tds.Rlin_cR-tds.R_cR	mol/(m³·s)	Equation residual	Domains 1–3	
tds.Rlin_cO	0	1/s	Linear source term coefficient	Domains 1–3	+ operation
tds.Res_cO	tds.u*cOr+tds.w*cOz-cO*tds.Rlin_cO-tds.R_cO	mol/(m³·s)	Equation residual	Domains 1–3	

Shape functions

Name	Shape function	Unit	Description	Shape frame	Selection
cR	Lagrange (Linear)	mol/m³	Concentration	Spatial	Domains 1–3
cO	Lagrange (Linear)	mol/m³	Concentration	Spatial	Domains 1–3

Weak Expressions

Weak expression	Integration order	Integration frame	Selection
2*(tds.dflux_cRr*test(cRr)+tds.dflux_cRz*test(cRz))*tds.d*pi*r	2	Spatial	Domains 1–3

Weak expression	Integration order	Integration frame	Selection
2*(tds.dflux_cOr*test(cOr)+tds.dflux_cOz*test(cOz))*tds.d*pi*r	2	Spatial	Domains 1–3
-2*(tds.u*cRr+tds.w*cRz)*test(cR)*(isScalingSystemDomain==0)*tds.d*pi*r	2	Spatial	Domains 1–3
2*tds.cbf_cR*test(cR)*tds.d*pi*r	2	Spatial	Boundaries 1–7, 9–17
-2*(tds.u*cOr+tds.w*cOz)*test(cO)*(isScalingSystemDomain==0)*tds.d*pi*r	2	Spatial	Domains 1–3
2*tds.cbf_cO*test(cO)*tds.d*pi*r	2	Spatial	Boundaries 1–7, 9–17
2*tds.streamline*(isScalingSystemDomain==0)*tds.d*pi*r	2	Spatial	Domains 1–3
2*tds.crosswind*(isScalingSystemDomain==0)*tds.d*pi*r	4	Spatial	Domains 1–3

2.3.4 Axial Symmetry 1

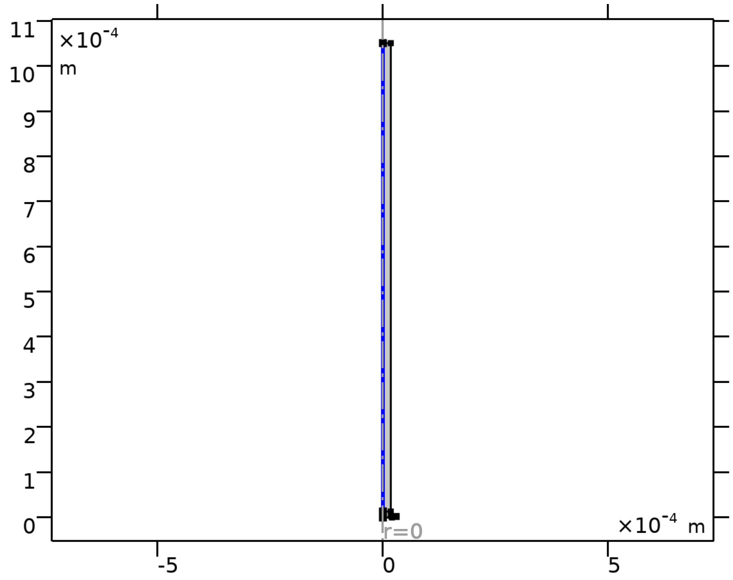

Axial Symmetry 1

SELECTION

Geometric entity level	Boundary
Selection	Geometry geom1: Dimension 1: All boundaries

2.3.5 No Flux 1

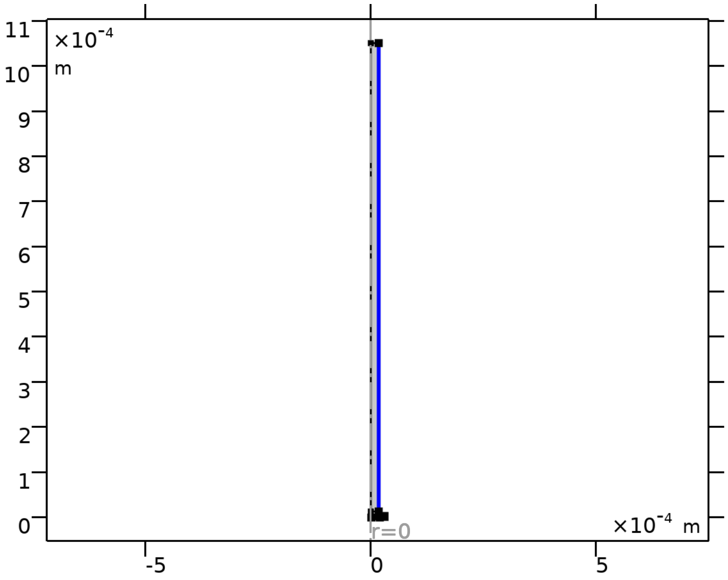

No Flux 1

SELECTION

Geometric entity level	Boundary
Selection	Geometry geom1: Dimension 1: All boundaries

EQUATIONS

$$-\mathbf{n}\cdot\mathbf{J}_i=0$$

Convection

SETTINGS

Description	Value
Include	Off

2.3.6 Initial Values 1

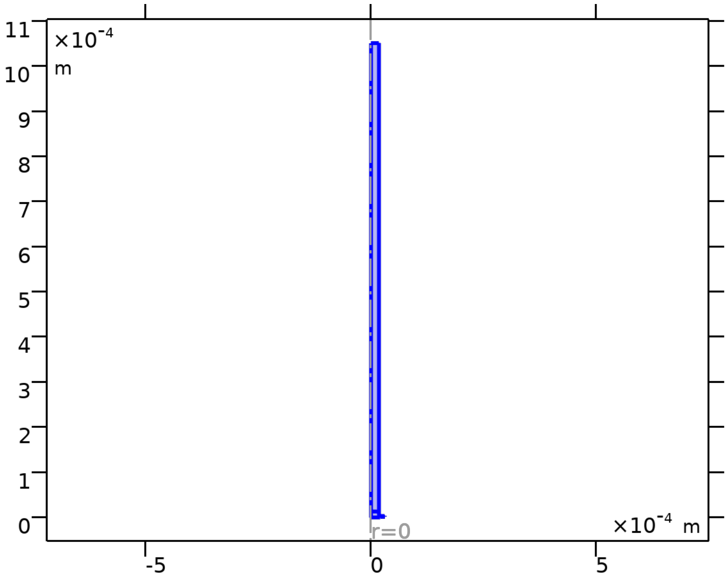

Initial Values 1

SELECTION

Geometric entity level	Domain
Selection	Geometry geom1: Dimension 2: All domains

Initial Values

SETTINGS

**Description**	**Value**	**Unit**
Concentration	{cRbulk, 0}	mol/m³

Variables

**Name**	**Expression**	**Unit**	**Description**	**Selection**	**Details**
tds.c0_cR	cRbulk	mol/m³	Concentration	Domains 1–3	+ operation
tds.c0_cO	0	mol/m³	Concentration	Domains 1–3	+ operation

2.3.7 Electrode (Flux)

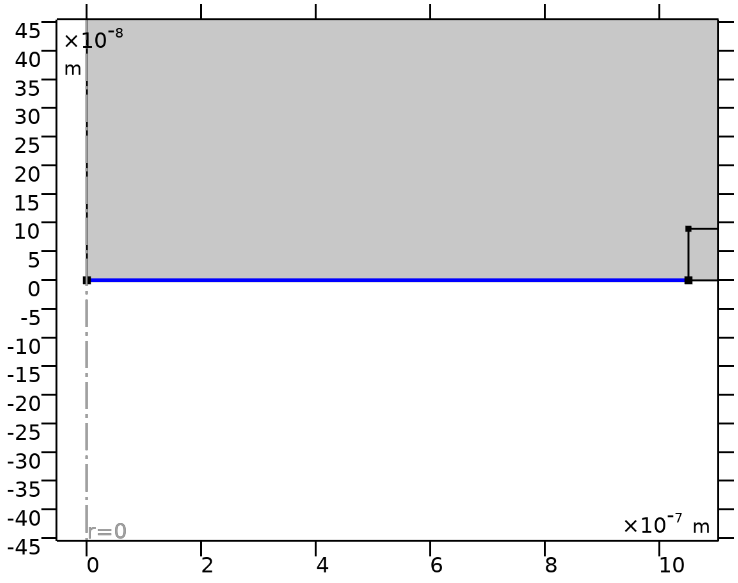

Electrode (Flux)

SELECTION

Geometric entity level	Boundary
Selection	Geometry geom1: Dimension 1: Boundary 2

EQUATIONS

$$-\mathbf{n}\cdot\mathbf{J}_i = J_{0,i}$$

Convection

SETTINGS

Description	Value
Include	Off

Inward Flux

SETTINGS

Description	Value	Unit
Flux type	General inward flux	
Species cR	On	
Species cO	On	
	{k0*(exp(-a*f*(cellE-Ef))*cO - exp((1 - a)*f*(cellE-Ef))*cR), k0*(-exp(-a*f*(cellE-Ef))*cO + exp((1 - a)*f*(cellE-Ef))*cR)}	mol/(m²·s)

Variables

Name	Expression	Unit	Description	Selection

Name	Expression	Unit	Description	Selection
tds.fl1.nmflow_cR	tds.fl1.int(2*tds.ntflux_cR*pi*r)*tds.d	mol/s	Normal molar flow rate	Global
tds.fl1.nmflow_cO	tds.fl1.int(2*tds.ntflux_cO*pi*r)*tds.d	mol/s	Normal molar flow rate	Global

Weak Expressions

Weak expression	Integration order	Integration frame	Selection
2*k0*(exp(-a*f*(cellE-Ef))*cO-exp((1-a)*f*(cellE-Ef))*cR)*test(cR)*tds.d*pi*r	2	Spatial	Boundary 2
2*k0*(-exp(-a*f*(cellE-Ef))*cO+exp((1-a)*f*(cellE-Ef))*cR)*test(cO)*tds.d*pi*r	2	Spatial	Boundary 2

2.3.8 Pipet Back (Conc)

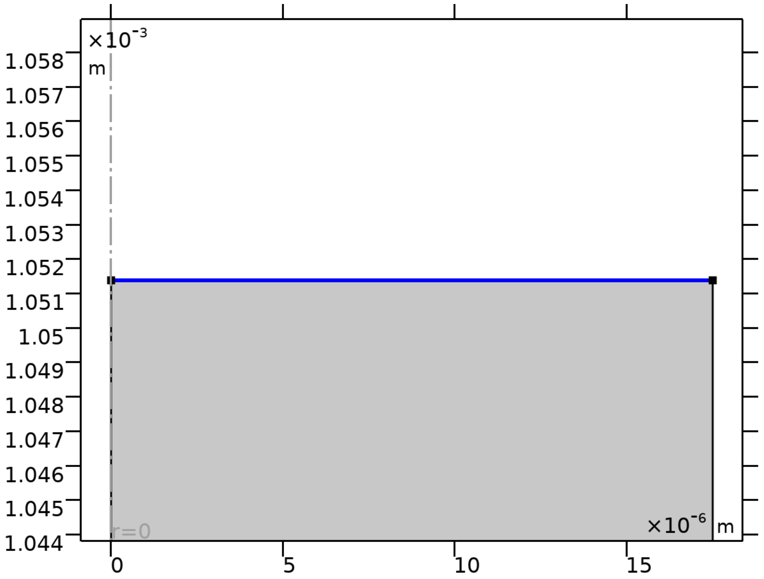

Pipet Back (Conc)

SELECTION

Geometric entity level	Boundary
Selection	Geometry geom1: Dimension 1: Boundary 6

EQUATIONS

$$c_i = c_{0,j}$$

Concentration

SETTINGS

Description	Value	Unit

Description	Value	Unit
Species cR	On	
Species cO	On	
Concentration	{cRbulk, 0}	mol/m^3

Variables

Name	Expression	Unit	Description	Selection	Details
tds.c0_cR	cRbulk	mol/m^3	Concentration	Boundary 6	+ operation
tds.c0_cO	0	mol/m^3	Concentration	Boundary 6	+ operation
tds.conc1.nmflow_cR	tds.conc1.int(2*tds.ntflux_cR*pi*r)*tds.d	mol/s	Normal molar flow rate	Global	
tds.conc1.nmflow_cO	tds.conc1.int(2*tds.ntflux_cO*pi*r)*tds.d	mol/s	Normal molar flow rate	Global	

Constraints

Constraint	Constraint force	Shape function	Selection	Details
-tds.cVar_cR+tds.c0_cR	test(-tds.cVar_cR+tds.c0_cR)	Lagrange (Linear)	Boundary 6	Elemental
-tds.cVar_cO+tds.c0_cO	test(-tds.cVar_cO+tds.c0_cO)	Lagrange (Linear)	Boundary 6	Elemental

2.3.9 Meniscus (Flux)

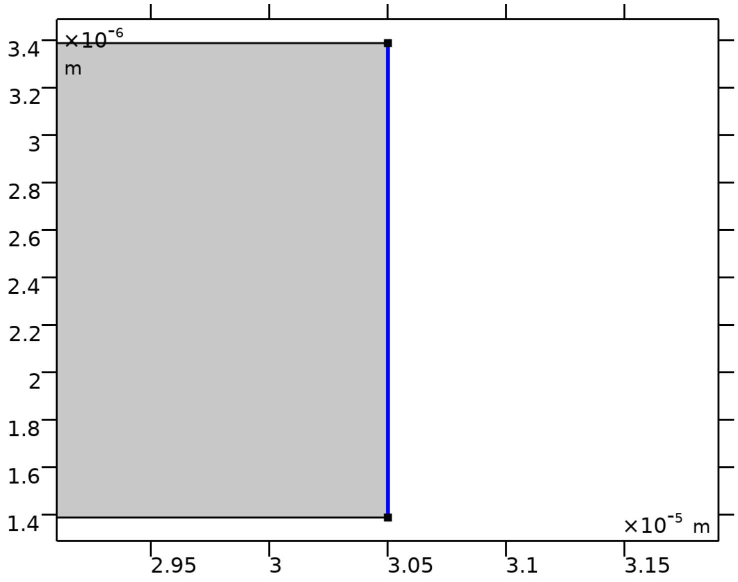

Meniscus (Flux)

SELECTION

Geometric entity level	Boundary
Selection	Geometry geom1: Dimension 1: Boundary 16

EQUATIONS

$$-\mathbf{n}\cdot(\mathbf{J}_i+\mathbf{u}c_i)=J_{0,i}$$

Convection

SETTINGS

Description	Value
Include	On

Inward Flux

SETTINGS

Description	Value	Unit
Flux type	General inward flux	
Species cR	On	
Species cO	On	
	{0, 0}	mol/(m²·s)

Variables

Name	Expression	Unit	Description	Selection
tds.cbf_cR	cR*(tds.u*tds.nrmesh+tds.v*tds.nphimesh+tds.w*tds.nzmesh)	mol/(m²·s)	Convective boundary flux	Boundary 16
tds.cbf_cO	cO*(tds.u*tds.nrmesh+tds.v*tds.nphimesh+tds.w*tds.nzmesh)	mol/(m²·s)	Convective boundary flux	Boundary 16
tds.fl2.nmflow_cR	tds.fl2.int(2*tds.ntflux_cR*pi*r)*tds.d	mol/s	Normal molar flow rate	Global
tds.fl2.nmflow_cO	tds.fl2.int(2*tds.ntflux_cO*pi*r)*tds.d	mol/s	Normal molar flow rate	Global

Weak Expressions

Weak expression	Integration order	Integration frame	Selection
0	2	Spatial	Boundary 16
0	2	Spatial	Boundary 16

2.4 CREEPING FLOW

USED PRODUCTS

COMSOL Multiphysics
Electrochemistry Module

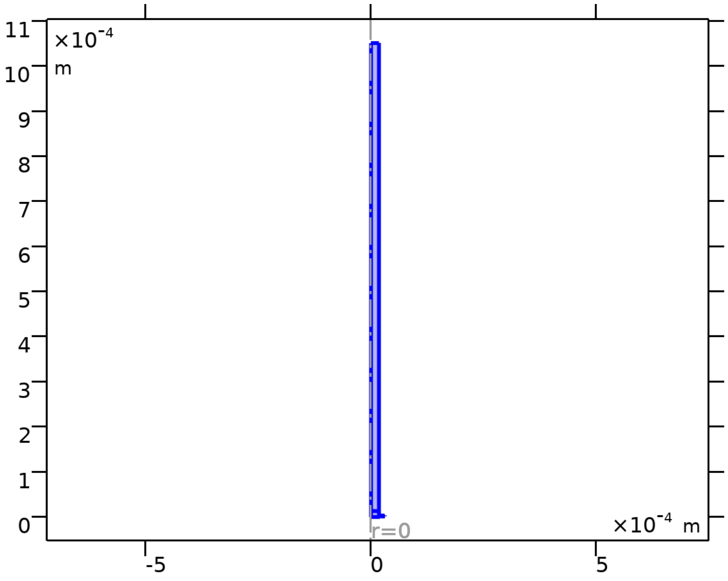

Creeping Flow

SELECTION

Geometric entity level	Domain
Selection	Geometry geom1: Dimension 2: Domains 1–3

EQUATIONS

$$\rho\frac{\partial \mathbf{u}}{\partial t} = \nabla\cdot[-p\mathbf{I} + \mathbf{K}] + \mathbf{F}$$
$$\rho\nabla\cdot\mathbf{u} = 0$$

2.4.1 Interface Settings

Discretization

SETTINGS

Description	Value
Discretization of fluids	P1 + P1

SETTINGS

Description	Value
Equation form	Study controlled

Physical Model

SETTINGS

Description	Value	Unit
Neglect inertial term (Stokes flow)	On	
Compressibility	Incompressible flow	
Enable porous media domains	Off	
Include gravity	Off	
Reference temperature	User defined	
Reference temperature	293.15	K
Reference pressure level	1.0133E5	Pa

Turbulence

SETTINGS

Description	Value
Turbulence model type	None

2.4.2 Variables

Name	Expression	Unit	Description	Selection	Details
spf.Tref	model.input.Tref	K	Reference temperature	Global	Meta
spf.dz	1	m	Thickness	Domains 1–3	
spf.pref	1[atm]	Pa	Reference pressure level	Domains 1–3	
spf.pA	p+spf.pref	Pa	Absolute pressure	Domains 1–3	
spf.hasWF	0		Help variable	Boundaries 1–4, 6–7, 9–16	
spf.hasWF_u	0		Help variable	Boundaries 5, 17	
spf.hasWF_d	0		Help variable	Boundaries 5, 17	
spf.dt_CFL	1/max(spf.maxop(sqrt(emetric_spatial(u-d(r,TIME),w-d(z,TIME)))),eps)	s	Time step, CFL=1	Global	
spf.CFL_number	timestep/spf.dt_CFL	1	CFL number	Global	
spf.Qvd_tot	spf.intop(2*spf.Qvd*pi*r)	W	Total viscous dissipation	Global	
spf.K_stressr	spf.K_stress_tensorrr*spf.nrmesh+spf.K_stress_tensorrphi*spf.nphimesh+spf.K_stress_ten	N/m^2	Viscous force, exterior boundaries, r-component	Boundaries 1–4, 6–7, 9–16	

Name	Expression	Unit	Description	Selection	Details
	sorrz*spf.nzmesh				
spf.K_stressphi	spf.K_stress_tensorphir*spf.nrmesh+spf.K_stress_tensorphiphi*spf.nphimesh+spf.K_stress_tensorphiz*spf.nzmesh	N/m²	Viscous force, exterior boundaries, phi-component	Boundaries 1–4, 6–7, 9–16	
spf.K_stressz	spf.K_stress_tensorzr*spf.nrmesh+spf.K_stress_tensorzphi*spf.nphimesh+spf.K_stress_tensorzz*spf.nzmesh	N/m²	Viscous force, exterior boundaries, z-component	Boundaries 1–4, 6–7, 9–16	
spf.T_stressr	spf.T_stress_tensorrr*spf.nrmesh+spf.T_stress_tensorrphi*spf.nphimesh+spf.T_stress_tensorrz*spf.nzmesh	N/m²	Total traction, exterior boundaries, r-component	Boundaries 1–4, 6–7, 9–16	
spf.T_stressphi	spf.T_stress_tensorphir*spf.nrmesh+spf.T_stress_tensorphiphi*spf.nphimesh+spf.T_stress_tensorphiz*spf.nzmesh	N/m²	Total traction, exterior boundaries, phi-component	Boundaries 1–4, 6–7, 9–16	
spf.T_stressz	spf.T_stress_tensorzr*spf.nrmesh+spf.T_stress_tensorzphi*spf.nphimesh+spf.T_stress_tensorzz*spf.nzmesh	N/m²	Total traction, exterior boundaries, z-component	Boundaries 1–4, 6–7, 9–16	
spf.K_stress_dr	down(spf.K_stress_tensorrr)*spf.nrmesh+down(spf.K_stress_tensorrphi)*spf.nphimesh+down(spf.K_stress_tensorrz)*spf.nzmesh	N/m²	Viscous force, interior boundaries, downside, r-component	Boundaries 5, 17	
spf.K_stress_dphi	down(spf.K_stress_tensorphir)*spf.nrmesh+down(spf.K_stress_tensorphiphi)*spf.nphimesh+down(spf.K_stress_tensorphiz)*spf.nzmesh	N/m²	Viscous force, interior boundaries, downside, phi-component	Boundaries 5, 17	
spf.K_stress_dz	down(spf.K_stress_tensorzr)*spf.nrmesh+down(spf.K_stress_tensorzphi)*spf.nphimesh+down(spf.K_stress_tens	N/m²	Viscous force, interior boundaries, downside, z-component	Boundaries 5, 17	

Name	Expression	Unit	Description	Selection	Details
	orzz)*spf.nzmesh				
spf.K_stress_dr	down(spf.K_stress_tensorrr)*spf.dnrmesh+down(spf.K_stress_tensorrphi)*spf.dnphimesh+down(spf.K_stress_tensorrz)*spf.dnzmesh	N/m^2	Viscous force, interior boundaries, downside, r-component	Boundaries 1–4, 6–7, 9–16	
spf.K_stress_dphi	down(spf.K_stress_tensorphir)*spf.dnrmesh+down(spf.K_stress_tensorphiphi)*spf.dnphimesh+down(spf.K_stress_tensorphiz)*spf.dnzmesh	N/m^2	Viscous force, interior boundaries, downside, phi-component	Boundaries 1–4, 6–7, 9–16	
spf.K_stress_dz	down(spf.K_stress_tensorzr)*spf.dnrmesh+down(spf.K_stress_tensorzphi)*spf.dnphimesh+down(spf.K_stress_tensorzz)*spf.dnzmesh	N/m^2	Viscous force, interior boundaries, downside, z-component	Boundaries 1–4, 6–7, 9–16	
spf.K_stress_ur	-up(spf.K_stress_tensorrr)*spf.nrmesh-up(spf.K_stress_tensorrphi)*spf.nphimesh-up(spf.K_stress_tensorrz)*spf.nzmesh	N/m^2	Viscous force, interior boundaries, upside, r-component	Boundaries 5, 17	
spf.K_stress_uphi	-up(spf.K_stress_tensorphir)*spf.nrmesh-up(spf.K_stress_tensorphiphi)*spf.nphimesh-up(spf.K_stress_tensorphiz)*spf.nzmesh	N/m^2	Viscous force, interior boundaries, upside, phi-component	Boundaries 5, 17	
spf.K_stress_uz	-up(spf.K_stress_tensorzr)*spf.nrmesh-up(spf.K_stress_tensorzphi)*spf.nphimesh-up(spf.K_stress_tensorzz)*spf.nzmesh	N/m^2	Viscous force, interior boundaries, upside, z-component	Boundaries 5, 17	
spf.T_stress_dr	down(spf.T_stress_tensorrr)*spf.nrmesh+down(spf.T_stress_tensorrphi)*spf.nphimesh+d	N/m^2	Total traction, interior boundaries, downside, r-	Boundaries 5, 17	

Name	Expression	Unit	Description	Selection	Details
	own(spf.T_stress_tensorrz)*spf.nzmesh		component		
spf.T_stress_dphi	down(spf.T_stress_tensorphir)*spf.nrmesh+down(spf.T_stress_tensorphiphi)*spf.nphimesh+down(spf.T_stress_tensorphiz)*spf.nzmesh	N/m²	Total traction, interior boundaries, downside, phi-component	Boundaries 5, 17	
spf.T_stress_dz	down(spf.T_stress_tensorzr)*spf.nrmesh+down(spf.T_stress_tensorzphi)*spf.nphimesh+down(spf.T_stress_tensorzz)*spf.nzmesh	N/m²	Total traction, interior boundaries, downside, z-component	Boundaries 5, 17	
spf.T_stress_dr	down(spf.T_stress_tensorrr)*spf.dnrmesh+down(spf.T_stress_tensorrphi)*spf.dnphimesh+down(spf.T_stress_tensorrz)*spf.dnzmesh	N/m²	Total traction, interior boundaries, downside, r-component	Boundaries 1–4, 6–7, 9–16	
spf.T_stress_dphi	down(spf.T_stress_tensorphir)*spf.dnrmesh+down(spf.T_stress_tensorphiphi)*spf.dnphimesh+down(spf.T_stress_tensorphiz)*spf.dnzmesh	N/m²	Total traction, interior boundaries, downside, phi-component	Boundaries 1–4, 6–7, 9–16	
spf.T_stress_dz	down(spf.T_stress_tensorzr)*spf.dnrmesh+down(spf.T_stress_tensorzphi)*spf.dnphimesh+down(spf.T_stress_tensorzz)*spf.dnzmesh	N/m²	Total traction, interior boundaries, downside, z-component	Boundaries 1–4, 6–7, 9–16	
spf.T_stress_ur	-up(spf.T_stress_tensorrr)*spf.nrmesh-up(spf.T_stress_tensorrphi)*spf.nphimesh-up(spf.T_stress_tensorrz)*spf.nzmesh	N/m²	Total traction, interior boundaries, upside, r-component	Boundaries 5, 17	
spf.T_stress_uphi	-up(spf.T_stress_tensorphir)*spf.nrmesh-up(spf.T_stress_tensorphiphi)*spf.nphimesh-	N/m²	Total traction, interior boundaries, upside, phi-component	Boundaries 5, 17	

Name	Expression	Unit	Description	Selection	Details
	up(spf.T_stress_tensor phiz)*spf.nzmesh				
spf.T_stress_uz	- up(spf.T_stress_tensor zr)*spf.nrmesh- up(spf.T_stress_tensor zphi)*spf.nphimesh- up(spf.T_stress_tensor zz)*spf.nzmesh	N/m²	Total traction, interior boundaries, upside, z-component	Boundaries 5, 17	
spf.usePseudoTimeS tepping	isrunningpseudotimes tepping	1	Help variable	Global	
spf.localCFLvalue	1.3^min(niterCMP,9)+ if(niterCMP>=25,9*1.3 ^min(- 25+niterCMP,9),0)+if(niterCMP>=45,90*1.3 ^min(- 45+niterCMP,9),0)		Local CFL number	Domains 1–3	
spf.locCFL	max(CFLCMP,sqrt(eps))	1	Local CFL number	Global	
spf.geometryLength Scale	7.625E-6	m	Geometry length scale	Domains 1–3	
spf.time_step_inv	max(sqrt(emetric_spati al(u,w)*2^gmg_level^ 2),spf.nu/spf.geometry LengthScale^2)	Hz	Inverse time step	Domains 1–3	
spf.tsti	nojac(spf.time_step_in v/spf.locCFL)	1/s	Help variable	Domains 1–3	
spf.nr	nr	1	Normal vector, r-component	Boundaries 5, 17	
spf.nphi	0	1	Normal vector, phi-component	Boundaries 5, 17	
spf.nz	nz	1	Normal vector, z-component	Boundaries 5, 17	
spf.nr	dnr	1	Normal vector, r-component	Boundaries 1–4, 6–7, 9–16	
spf.nphi	0	1	Normal vector, phi-component	Boundaries 1–4, 6–7, 9–16	
spf.nz	dnz	1	Normal vector, z-component	Boundaries 1–4, 6–7, 9–16	
spf.nrmesh	nrmesh	1	Normal vector,	Boundaries 5,	

Name	Expression	Unit	Description	Selection	Details
			r-component	17	
spf.nphimesh	0	1	Normal vector, phi-component	Boundaries 5, 17	
spf.nzmesh	nzmesh	1	Normal vector, z-component	Boundaries 5, 17	
spf.nrmesh	dnrmesh	1	Normal vector, r-component	Boundaries 1–4, 6–7, 9–16	
spf.nphimesh	0	1	Normal vector, phi-component	Boundaries 1–4, 6–7, 9–16	
spf.nzmesh	dnzmesh	1	Normal vector, z-component	Boundaries 1–4, 6–7, 9–16	

2.4.3 Fluid Properties 1

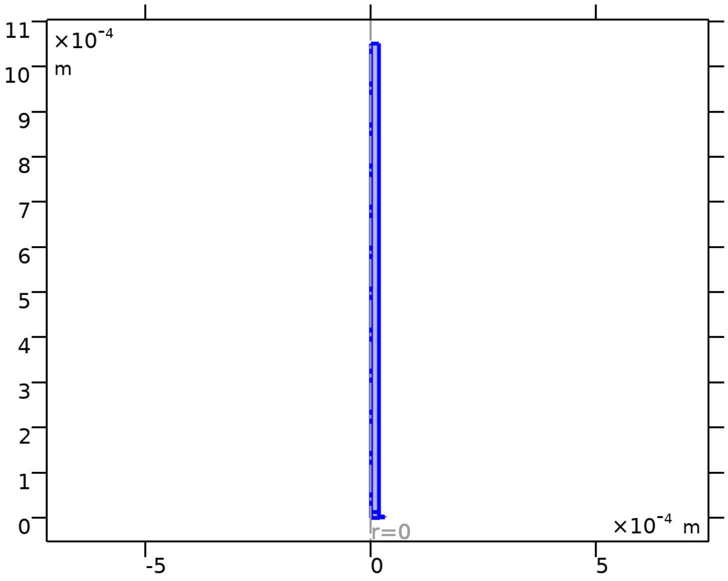

Fluid Properties 1

SELECTION

Geometric entity level	Domain
Selection	Geometry geom1: Dimension 2: All domains

EQUATIONS

$$\rho\frac{\partial \mathbf{u}}{\partial t} = \nabla\cdot[-p\mathbf{I} + \mathbf{K}] + \mathbf{F}$$

$$\rho\nabla\cdot\mathbf{u} = 0$$

$$\mathbf{K} = \mu\left(\nabla\mathbf{u} + (\nabla\mathbf{u})^T\right)$$

Fluid Properties

SETTINGS

Description	Value	Unit
Density	User defined	
Density	997.8	kg/m^3
	Specify dynamic viscosity	
Dynamic viscosity	User defined	
Dynamic viscosity	9.544E-4	Pa·s

Variables

Name	Expression	Unit	Description	Selection	Details
spf.mu	material.mu	Pa·s	Dynamic viscosity	Domains 1–3	Meta
spf.rho	material.rho	kg/m^3	Density	Domains 1–3	Meta
spf.Trho	spf.fp1.minput_temperature	K	Temperature for density evaluation	Domains 1–3	
spf.prho	spf.fp1.minput_pressure	Pa	Pressure for the evaluation of density	Domains 1–3	
spf.rhoref	subst(material.rho,minput.T,spf.Tref,minput.pA,spf.pref)	kg/m^3	Reference density	Domains 1–3	Meta
spf.mumat	material.mu	Pa·s	Dynamic viscosity	Domains 1–3	Meta
spf.srijrr	ur	1/s	Strain rate tensor, rr-component	Domains 1–3	
spf.srijphir	0	1/s	Strain rate tensor, phir-component	Domains 1–3	
spf.srijzr	0.5*(wr+uz)	1/s	Strain rate tensor, zr-component	Domains 1–3	
spf.srijrphi	0	1/s	Strain rate tensor, rphi-component	Domains 1–3	
spf.srijphiphi	if(abs(r)<0.001*h_spatial,ur,u/r)	1/s	Strain rate tensor, phiphi-component	Domains 1–3	

Name	Expression	Unit	Description	Selection	Details
spf.srijzphi	0	1/s	Strain rate tensor, zphi-component	Domains 1–3	
spf.srijrz	0.5*(uz+wr)	1/s	Strain rate tensor, rz-component	Domains 1–3	
spf.srijphiz	0	1/s	Strain rate tensor, phiz-component	Domains 1–3	
spf.srijzz	wz	1/s	Strain rate tensor, zz-component	Domains 1–3	
spf.rrijrr	0	1/s	Rotation rate tensor, rr-component	Domains 1–3	
spf.rrijphir	0	1/s	Rotation rate tensor, phir-component	Domains 1–3	
spf.rrijzr	0.5*(wr-uz)	1/s	Rotation rate tensor, zr-component	Domains 1–3	
spf.rrijrphi	0	1/s	Rotation rate tensor, rphi-component	Domains 1–3	
spf.rrijphiphi	0	1/s	Rotation rate tensor, phiphi-component	Domains 1–3	
spf.rrijzphi	0	1/s	Rotation rate tensor, zphi-component	Domains 1–3	
spf.rrijrz	0.5*(uz-wr)	1/s	Rotation rate tensor, rz-component	Domains 1–3	
spf.rrijphiz	0	1/s	Rotation rate tensor, phiz-component	Domains 1–3	
spf.rrijzz	0	1/s	Rotation rate tensor, zz-component	Domains 1–3	
spf.sr	sqrt(2*spf.srijrr^2+2*spf.srijrphi^2+2*spf.srijrz^2+2*spf.srijphir^2+2*	1/s	Shear rate	Domains 1–3	

Name	Expression	Unit	Description	Selection	Details
	spf.srijphiphi^2+2*spf.srijphiz^2+2*spf.srijzr^2+2*spf.srijzphi^2+2*spf.srijzz^2+eps)				
spf.rr	sqrt(2*spf.rrijrr^2+2*spf.rrijrphi^2+2*spf.rrijrz^2+2*spf.rrijphir^2+2*spf.rrijphiphi^2+2*spf.rrijphiz^2+2*spf.rrijzr^2+2*spf.rrijzphi^2+2*spf.rrijzz^2+eps)	1/s	Rotation rate	Domains 1–3	
spf.divu	ur+if(abs(r)<0.001*h_spatial,ur,u/r)+wz	1/s	Divergence of velocity field	Domains 1–3	
spf.Fr	0	N/m³	Volume force, r-component	Domains 1–3	+ operation
spf.Fphi	0	N/m³	Volume force, phi-component	Domains 1–3	+ operation
spf.Fz	0	N/m³	Volume force, z-component	Domains 1–3	+ operation
spf.U	sqrt(u^2+w^2)	m/s	Velocity magnitude	Domains 1–3	
spf.vorticityr	0	1/s	Vorticity field, r-component	Domains 1–3	
spf.vorticityphi	-wr+uz	1/s	Vorticity field, phi-component	Domains 1–3	
spf.vorticityz	0	1/s	Vorticity field, z-component	Domains 1–3	
spf.vort_magn	sqrt(spf.vorticityr^2+spf.vorticityphi^2+spf.vorticityz^2)	1/s	Vorticity magnitude	Domains 1–3	
spf.cellRe	0.25*spf.rho*sqrt(emetric_spatial(u-d(r,TIME),w-d(z,TIME))/emetric2_spatial)/spf.mu	1	Cell Reynolds number	Domains 1–3	
spf.nu	spf.mu/spf.rho	m²/s	Kinematic viscosity	Domains 1–3	

Name	Expression	Unit	Description	Selection	Details
spf.betaT	0	1/Pa	Isothermal compressibility coefficient	Domains 1–3	
spf.Qm	0	kg/(m^3·s)	Source term	Domains 1–3	+ operation
spf.Fgtotr	0	N/m^3	Gravity force, r-component	Domains 1–3	+ operation
spf.Fgtotphi	0	N/m^3	Gravity force, phi-component	Domains 1–3	+ operation
spf.Fgtotz	0	N/m^3	Gravity force, z-component	Domains 1–3	+ operation
spf.Qm_aco	0	kg/(m^3·s)	Acoustic mass source	Domains 1–3	
spf.F_acor	0	N/m^3	Acoustic volume force, r-component	Domains 1–3	
spf.F_acophi	0	N/m^3	Acoustic volume force, phi-component	Domains 1–3	
spf.F_acoz	0	N/m^3	Acoustic volume force, z-component	Domains 1–3	
spf.gamma_sr	sqrt(2*spf.srijrr^2+2*spf.srijrphi^2+2*spf.srijrz^2+2*spf.srijphir^2+2*spf.srijphiphi^2+2*spf.srijphiz^2+2*spf.srijzr^2+2*spf.srijzphi^2+2*spf.srijzz^2+eps)	1/s	Shear rate	Domains 1–3	
spf.mu_eff	spf.mu+spf.muT	Pa·s	Effective dynamic viscosity	Domains 1–3	
spf.muT	0	Pa·s	Turbulent dynamic viscosity	Domains 1–3	+ operation
spf.T_stress_tensorrr	spf.K_stress_tensorrr-p	N/m^2	Total stress tensor, rr-component	Domains 1–3	+ operation
spf.T_stress_tensorphir	spf.K_stress_tensorphir	N/m^2	Total stress tensor, phir-component	Domains 1–3	+ operation

Name	Expression	Unit	Description	Selection	Details
spf.T_stress_tensorzr	spf.K_stress_tensorzr	N/m²	Total stress tensor, zr-component	Domains 1–3	+ operation
spf.T_stress_tensorrphi	spf.K_stress_tensorrphi	N/m²	Total stress tensor, rphi-component	Domains 1–3	+ operation
spf.T_stress_tensorphiphi	spf.K_stress_tensorphiphi-p	N/m²	Total stress tensor, phiphi-component	Domains 1–3	+ operation
spf.T_stress_tensorzphi	spf.K_stress_tensorzphi	N/m²	Total stress tensor, zphi-component	Domains 1–3	+ operation
spf.T_stress_tensorrz	spf.K_stress_tensorrz	N/m²	Total stress tensor, rz-component	Domains 1–3	+ operation
spf.T_stress_tensorphiz	spf.K_stress_tensorphiz	N/m²	Total stress tensor, phiz-component	Domains 1–3	+ operation
spf.T_stress_tensorzz	spf.K_stress_tensorzz-p	N/m²	Total stress tensor, zz-component	Domains 1–3	+ operation
spf.K_stress_tensorrr	2*spf.mu_eff*ur	N/m²	Viscous stress tensor, rr-component	Domains 1–3	+ operation
spf.K_stress_tensorphir	0	N/m²	Viscous stress tensor, phir-component	Domains 1–3	+ operation
spf.K_stress_tensorzr	spf.mu_eff*(wr+uz)	N/m²	Viscous stress tensor, zr-component	Domains 1–3	+ operation
spf.K_stress_tensorrphi	0	N/m²	Viscous stress tensor, rphi-component	Domains 1–3	+ operation
spf.K_stress_tensorphiphi	2*spf.mu_eff*if(abs(r)<0.001*h_spatial,ur,u/r)	N/m²	Viscous stress tensor, phiphi-component	Domains 1–3	+ operation
spf.K_stress_tensorzphi	0	N/m²	Viscous stress tensor, zphi-component	Domains 1–3	+ operation
spf.K_stress_tensorrz	spf.mu_eff*(uz+wr)	N/m²	Viscous stress tensor, rz-component	Domains 1–3	+ operation

Name	Expression	Unit	Description	Selection	Details
spf.K_stress_tensorphiz	0	N/m²	Viscous stress tensor, phiz-component	Domains 1–3	+ operation
spf.K_stress_tensorzz	2*spf.mu_eff*wz	N/m²	Viscous stress tensor, zz-component	Domains 1–3	+ operation
spf.K_stress_tensor_testrr	2*spf.mu_eff*test(ur)	N/m²	Viscous stress tensor test, rr-component	Domains 1–3	+ operation
spf.K_stress_tensor_testphir	0	N/m²	Viscous stress tensor test, phir-component	Domains 1–3	+ operation
spf.K_stress_tensor_testzr	spf.mu_eff*(test(wr)+test(uz))	N/m²	Viscous stress tensor test, zr-component	Domains 1–3	+ operation
spf.K_stress_tensor_testrphi	0	N/m²	Viscous stress tensor test, rphi-component	Domains 1–3	+ operation
spf.K_stress_tensor_testphiphi	2*spf.mu_eff*if(abs(r)<0.001*h_spatial,test(ur),test(u)/r)	N/m²	Viscous stress tensor test, phiphi-component	Domains 1–3	+ operation
spf.K_stress_tensor_testzphi	0	N/m²	Viscous stress tensor test, zphi-component	Domains 1–3	+ operation
spf.K_stress_tensor_testrz	spf.mu_eff*(test(uz)+test(wr))	N/m²	Viscous stress tensor test, rz-component	Domains 1–3	+ operation
spf.K_stress_tensor_testphiz	0	N/m²	Viscous stress tensor test, phiz-component	Domains 1–3	+ operation
spf.K_stress_tensor_testzz	2*spf.mu_eff*test(wz)	N/m²	Viscous stress tensor test, zz-component	Domains 1–3	+ operation
spf.upwind_helpr	-d(r,TIME)	m/s	Upwind term, r-component	Domains 1–3	+ operation
spf.upwind_helpphi	0	m/s	Upwind term, phi-component	Domains 1–3	+ operation
spf.upwind_helpz	-d(z,TIME)	m/s	Upwind term, z-component	Domains 1–3	+ operation
spf.continuityEquation	spf.rho*spf.divu	kg/(m³·s)	Continuity equation	Domains 1–3	

Name	Expression	Unit	Description	Selection	Details
spf.contCoeff	spf.rho	kg/m³	Help variable	Domains 1–3	
spf.tau_vdrr	2*spf.mu*spf.srijrr	Pa	Viscous stress tensor, rr-component	Domains 1–3	+ operation
spf.tau_vdphir	2*spf.mu*spf.srijphir	Pa	Viscous stress tensor, phir-component	Domains 1–3	+ operation
spf.tau_vdzr	2*spf.mu*spf.srijzr	Pa	Viscous stress tensor, zr-component	Domains 1–3	+ operation
spf.tau_vdrphi	2*spf.mu*spf.srijrphi	Pa	Viscous stress tensor, rphi-component	Domains 1–3	+ operation
spf.tau_vdphiphi	2*spf.mu*spf.srijphiphi	Pa	Viscous stress tensor, phiphi-component	Domains 1–3	+ operation
spf.tau_vdzphi	2*spf.mu*spf.srijzphi	Pa	Viscous stress tensor, zphi-component	Domains 1–3	+ operation
spf.tau_vdrz	2*spf.mu*spf.srijrz	Pa	Viscous stress tensor, rz-component	Domains 1–3	+ operation
spf.tau_vdphiz	2*spf.mu*spf.srijphiz	Pa	Viscous stress tensor, phiz-component	Domains 1–3	+ operation
spf.tau_vdzz	2*spf.mu*spf.srijzz	Pa	Viscous stress tensor, zz-component	Domains 1–3	+ operation
spf.Qvd	spf.tau_vdrr*ur+spf.tau_vdrz*uz+spf.tau_vdphiphi*if(abs(r)<0.001*h_spatial,ur,u/r)+spf.tau_vdzr*wr+spf.tau_vdzz*wz	W/m³	Viscous dissipation	Domains 1–3	+ operation
spf.epsilon_p	1	1	Porosity	Domains 1–3	
spf.epsilon_p_pos	1	1	Positive porosity	Domains 1–3	
spf.Fst_tensorrr	0	N/m²	Surface tension force, rr-component	Domains 1–3	+ operation
spf.Fst_tensorphir	0	N/m²	Surface tension force, phir-	Domains 1–3	+ operation

Name	Expression	Unit	Description	Selection	Details
			component		
spf.Fst_tensorzr	0	N/m²	Surface tension force, zr-component	Domains 1–3	+ operation
spf.Fst_tensorrphi	0	N/m²	Surface tension force, rphi-component	Domains 1–3	+ operation
spf.Fst_tensorphiphi	0	N/m²	Surface tension force, phiphi-component	Domains 1–3	+ operation
spf.Fst_tensorzphi	0	N/m²	Surface tension force, zphi-component	Domains 1–3	+ operation
spf.Fst_tensorrz	0	N/m²	Surface tension force, rz-component	Domains 1–3	+ operation
spf.Fst_tensorphiz	0	N/m²	Surface tension force, phiz-component	Domains 1–3	+ operation
spf.Fst_tensorzz	0	N/m²	Surface tension force, zz-component	Domains 1–3	+ operation
spf.res_u	spf.rho*ut+pr-(d(2*ur,r)+if(abs(r)<0.001*h_spatial,d(2*ur,r),2*ur/r)+d(uz+wr,z)-2*if(abs(r)<0.001*h_spatial,ur,u/r)/r)*spf.mu-spf.Fr	N/m³	Equation residual	Domains 1–3	
spf.res_v	-spf.Fphi	N/m³	Equation residual	Domains 1–3	
spf.res_w	spf.rho*wt+pz-(d(wr+uz,r)+if(abs(r)<0.001*h_spatial,d(wr+uz,r),(wr+uz)/r)+d(2*wz,z))*spf.mu-spf.Fz	N/m³	Equation residual	Domains 1–3	
spf.res_p	spf.rho*spf.divu	kg/(m³·s)	Pressure equation residual	Domains 1–3	

Shape functions

Name	Shape function	Unit	Description	Shape frame	Selection
u	Lagrange (Linear)	m/s	Velocity field, r-component	Spatial	Domains 1–3
w	Lagrange (Linear)	m/s	Velocity field, z-component	Spatial	Domains 1–3
u	Lagrange (Linear)	m/s	Velocity field, r-component	Spatial	Domains 1–3
w	Lagrange (Linear)	m/s	Velocity field, z-component	Spatial	Domains 1–3
p	Lagrange (Linear)	Pa	Pressure	Spatial	Domains 1–3

Weak Expressions

Weak expression	Integration order	Integration frame	Selection
2*spf.rho*(-ut*test(u)-wt*test(w))*pi*r	2	Spatial	Domains 1–3
2*((p-spf.K_stress_tensorrr)*test(ur)-spf.K_stress_tensorrz*test(uz)+(p-spf.K_stress_tensorphiphi)*if(abs(r)<0.001*h_spatial,test(ur),test(u)/r)-spf.K_stress_tensorzr*test(wr)+(p-spf.K_stress_tensorzz)*test(wz))*pi*r	2	Spatial	Domains 1–3
2*(spf.Fr*test(u)+spf.Fz*test(w))*pi*r	2	Spatial	Domains 1–3
-2*spf.continuityEquation*test(p)*pi*r	2	Spatial	Domains 1–3
2*spf.streamlinens*pi*r	2	Spatial	Domains 1–3

2.4.4 Initial Values 1

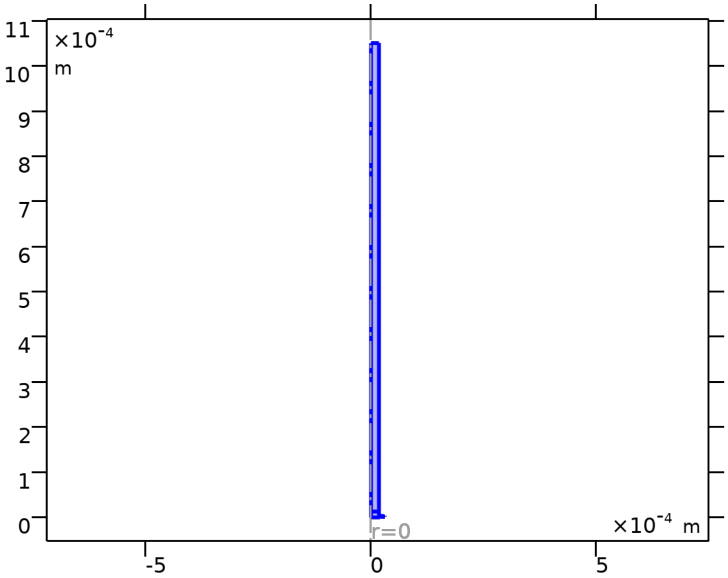

Initial Values 1

SELECTION

Geometric entity level	Domain
Selection	Geometry geom1: Dimension 2: All domains

Initial Values

SETTINGS

**Description**	**Value**	**Unit**
Velocity field, r-component	0	m/s
Velocity field, phi-component	0	m/s
Velocity field, z-component	0	m/s
Pressure	0	Pa

Coordinate System Selection

SETTINGS

**Description**	**Value**
Coordinate system	Global coordinate system

Variables

**Name**	**Expression**	**Unit**	**Description**	**Selection**
spf.u_initr	0	m/s	Velocity field, r-component	Domains 1–3
spf.u_initphi	0	m/s	Velocity field, phi-component	Domains 1–3
spf.u_initz	0	m/s	Velocity field, z-component	Domains 1–3

Name	Expression	Unit	Description	Selection
spf.p_init	0	Pa	Pressure	Domains 1–3

2.4.5 Axial Symmetry 1

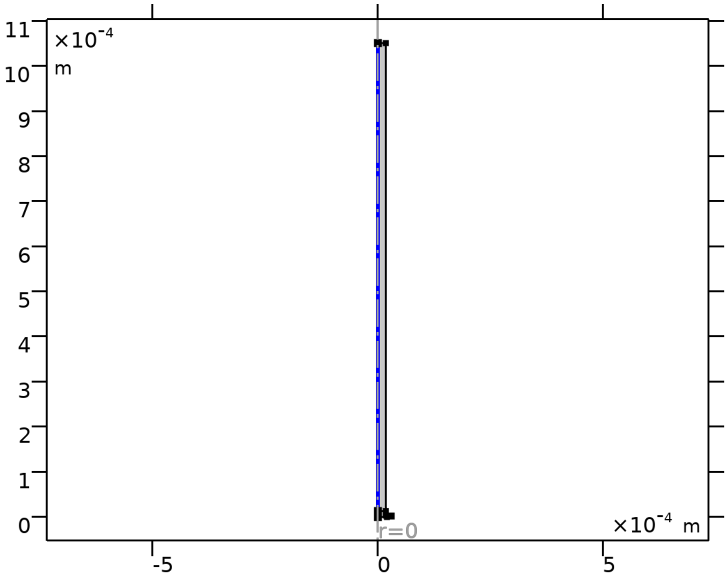

Axial Symmetry 1

SELECTION

Geometric entity level	Boundary
Selection	Geometry geom1: Dimension 1: All boundaries

Constraints

Constraint	Constraint force	Shape function	Selection	Details
-u	test(-u)	Lagrange (Linear)	Boundaries 1, 3–4	Elemental

2.4.6 Wall 1

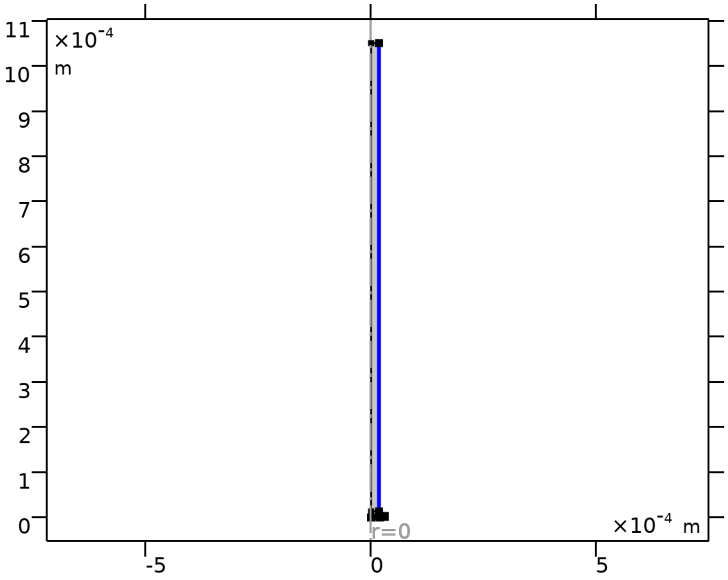

Wall 1

SELECTION

Geometric entity level	Boundary
Selection	Geometry geom1: Dimension 1: All boundaries

EQUATIONS

$$\mathbf{u} = \mathbf{0}$$

Boundary Condition

SETTINGS

**Description**	**Value**
Wall condition	No slip

Wall Movement

SETTINGS

**Description**	**Value**
Translational velocity	Automatic from frame
Sliding wall	Off

Variables

**Name**	**Expression**	**Unit**	**Description**	**Selection**	**Details**
spf.ubndr	spf.utrr+spf.usr	m/s	Velocity at boundary, r-component	Boundaries 2, 7, 9–15	

Name	Expression	Unit	Description	Selection	Details
spf.ubndphi	spf.utrphi+spf.usphi	m/s	Velocity at boundary, phi-component	Boundaries 2, 7, 9–15	
spf.ubndz	spf.utrz+spf.usz	m/s	Velocity at boundary, z-component	Boundaries 2, 7, 9–15	
spf.usr	0	m/s	Velocity of sliding wall, r-component	Boundaries 2, 7, 9–15	
spf.usphi	0	m/s	Velocity of sliding wall, phi-component	Boundaries 2, 7, 9–15	
spf.usz	0	m/s	Velocity of sliding wall, z-component	Boundaries 2, 7, 9–15	
spf.utrr	0	m/s	Velocity of moving wall, r-component	Boundaries 2, 7, 9–15	
spf.utrphi	0	m/s	Velocity of moving wall, phi-component	Boundaries 2, 7, 9–15	
spf.utrz	0	m/s	Velocity of moving wall, z-component	Boundaries 2, 7, 9–15	
spf.uLeakager	0	m/s	Leakage velocity, r-component	Boundaries 2, 7, 9–15	+ operation
spf.uLeakagephi	0	m/s	Leakage velocity, phi-component	Boundaries 2, 7, 9–15	+ operation
spf.uLeakagez	0	m/s	Leakage velocity, z-component	Boundaries 2, 7, 9–15	+ operation
spf.noSlipWall	1	1	Help variable	Boundaries 2, 7, 9–15	

Constraints

Constraint	Constraint force	Shape function	Selection	Details
-u+spf.ubndr+spf.uLeakager	test(-u)	Lagrange (Linear)	Boundaries 2, 7, 9–15	Elemental

Constraint	Constraint force	Shape function	Selection	Details
spf.ubndphi+spf.uLeakagephi	0		Boundaries 2, 7, 9–15	Elemental
-w+spf.ubndz+spf.uLeakagez	test(-w)	Lagrange (Linear)	Boundaries 2, 7, 9–15	Elemental

2.4.7 Open Boundary 1

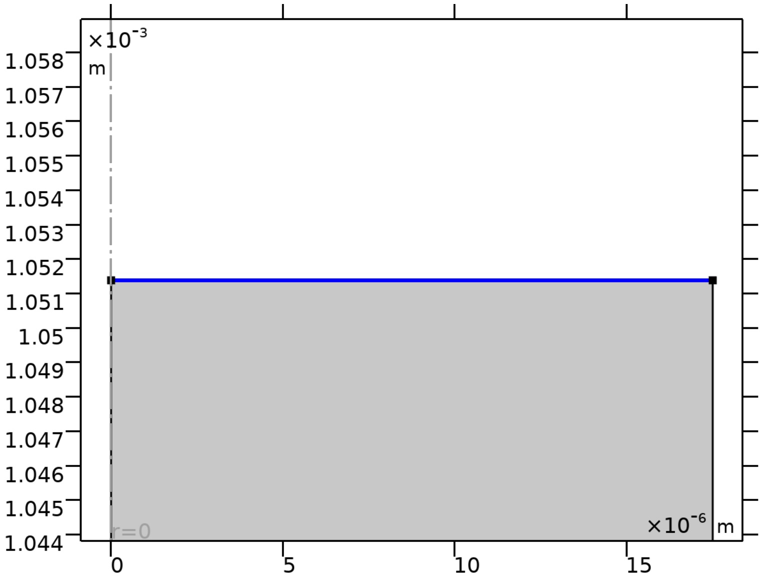

Open Boundary 1

SELECTION

Geometric entity level	Boundary
Selection	Geometry geom1: Dimension 1: Boundary 6

EQUATIONS

$$[-p\mathbf{I} + \mathbf{K}]\mathbf{n} = -f_0\mathbf{n}$$

Boundary Condition

SETTINGS

Description	Value	Unit
Boundary condition	Normal stress	
Normal stress	0	N/m²

Variables

Name	Expression	Unit	Description	Selection
spf.f0	0	N/m²	Normal stress	Boundary 6

Name	Expression	Unit	Description	Selection
spf.open1.volumeFlowRate	spf.open1.intop(2*(u*spf.nrmesh+w*spf.nzmesh)*pi*r)	m^3/s	Outward volume flow rate across feature selection	Global
spf.open1.massFlowRate	spf.open1.intop(2*spf.rho*(u*spf.nrmesh+w*spf.nzmesh)*pi*r)	kg/s	Outward mass flow rate across feature selection	Global
spf.open1.pAverage	spf.open1.intop(2*p*pi*r)/max(spf.open1.intop(2*pi*r),1000*eps)	Pa	Pressure average over feature selection	Global

Weak Expressions

Weak expression	Integration order	Integration frame	Selection
-2*spf.f0*(test(u)*spf.nrmesh+test(w)*spf.nzmesh)*pi*r	2	Spatial	Boundary 6

2.4.8 Outlet 1

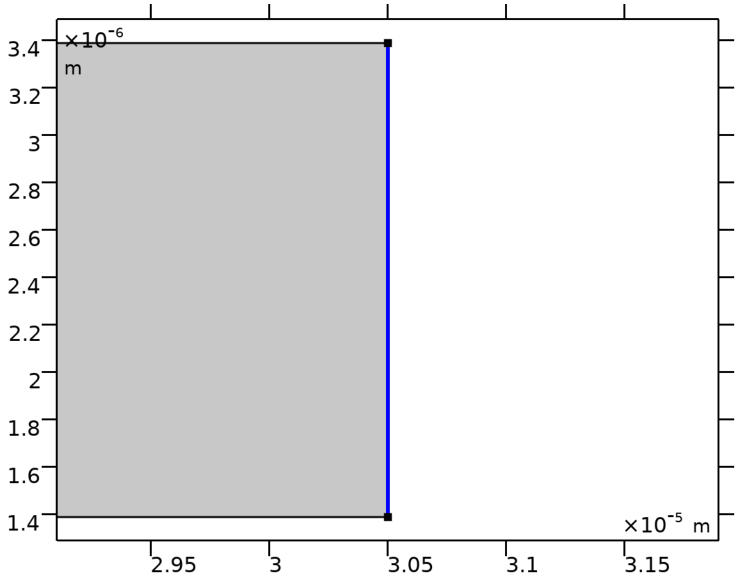

Outlet 1

SELECTION

Geometric entity level	Boundary
Selection	Geometry geom1: Dimension 1: Boundary 16

EQUATIONS

$$\mathbf{u} = U_0\mathbf{n}$$

Boundary Condition

SETTINGS

Description	Value
Boundary condition	Velocity

Velocity

SETTINGS

Description	Value	Unit
Velocity field componentwise	Normal outflow velocity	
Normal outflow velocity	vdry	m/s

Constraint Settings

SETTINGS

Description	Value
Apply reaction terms on	All physics (symmetric)
Use weak constraints	Off
Constraint method	Elemental

Variables

Name	Expression	Unit	Description	Selection
spf.ubndr	spf.nr*spf.U0out	m/s	Velocity at boundary, r-component	Boundary 16
spf.ubndphi	spf.nphi*spf.U0out	m/s	Velocity at boundary, phi-component	Boundary 16
spf.ubndz	spf.nz*spf.U0out	m/s	Velocity at boundary, z-component	Boundary 16
spf.U0out	vdry	m/s	Normal outflow velocity	Boundary 16
spf.out1.Uav	0	m/s	Average velocity	Global
spf.out1.Uavfdf	0	m/s	Average velocity	Global
spf.out1.dz	spf.dz	m	Channel	Boundary 16

Name	Expression	Unit	Description	Selection
			thickness	
spf.out1.Mflow	spf.out1.massFlowRate	kg/s	Mass flow	Global
spf.out1.volumeFlowRate	spf.out1.intop(2*(u*spf.nrmesh+w*spf.nzmesh)*pi*r)	m^3/s	Outward volume flow rate across feature selection	Global
spf.out1.massFlowRate	spf.out1.intop(2*spf.rho*(u*spf.nrmesh+w*spf.nzmesh)*pi*r)	kg/s	Outward mass flow rate across feature selection	Global
spf.out1.pAverage	spf.out1.intop(2*p*pi*r)/max(spf.out1.intop(2*pi*r),1000*eps)	Pa	Pressure average over feature selection	Global

Constraints

Constraint	Constraint force	Shape function	Selection	Details
-u+spf.ubndr	test(-u+spf.ubndr)	Lagrange (Linear)	Boundary 16	Elemental
spf.ubndphi	test(spf.ubndphi)		Boundary 16	Elemental
-w+spf.ubndz	test(-w+spf.ubndz)	Lagrange (Linear)	Boundary 16	Elemental

2.5 EVENTS

USED PRODUCTS

COMSOL Multiphysics

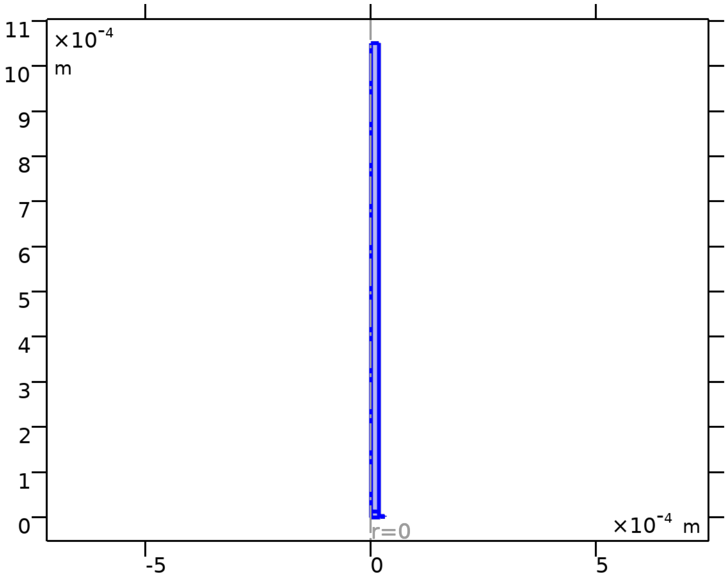

Events

SELECTION

Geometric entity level	Domain
Selection	Geometry geom1: Dimension 2: Domains 1–3

2.5.1 Interface Settings

Discretization

SETTINGS

Description	Value
Element order	Quadratic

SETTINGS

Description	Value
Equation form	Study controlled

2.5.2 Discrete States 1

SELECTION

Geometric entity level	Entire model

Discrete States

Name	Initial value (u0)	Description
cellE	-0.3	

Shape functions

Name	Shape function	Unit	Description	Shape frame	Selection
cellE	ODE		Discrete state, -component		Global

2.5.3 Explicit Event 1

SELECTION

Geometric entity level	Entire model

Event Timings

SETTINGS

Description	Value	Unit
Start of event	t_acc	s
Period of event	Inf	s
Use consistent initialization	On	

Reinitialization

Variable	Expression
cellE	0.3

2.6 MULTIPHYSICS

2.6.1 Flow Coupling 1

USED PRODUCTS

COMSOL Multiphysics

Coupled Interfaces

SETTINGS

Description	Value
Source	Creeping Flow (spf)
Destination	Transport of Diluted Species (tds)

Variables

Name	Expression	Unit	Description	Selection
fc1.uR	spatial.invF11*u+spatial.invF31*w	m/s	Velocity field, R-component	Global
fc1.uPHI	0	m/s	Velocity field, PHI-component	Global

Name	Expression	Unit	Description	Selection
fc1.uZ	spatial.invF13*u+spatial.invF33*w	m/s	Velocity field, Z-component	Global
fc1.p	p	Pa	Pressure	Global
fc1.pA	spf.pA	Pa	Absolute pressure	Global

2.7 MESH 1

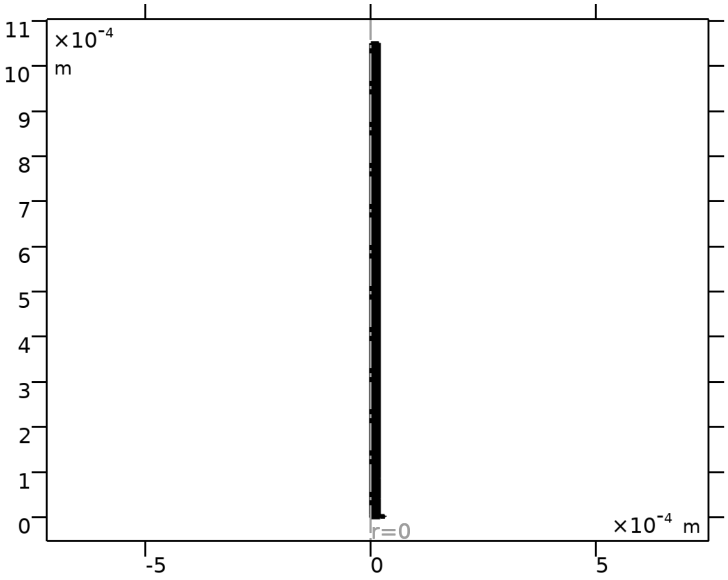

Mesh 1

2.7.1 Size (size)

SETTINGS

Description	Value
Maximum element size	2E-6
Minimum element size	1E-9
Curvature factor	0.25
Maximum element growth rate	1.11
Predefined size	Extra fine
Custom element size	Custom

2.7.2 Size 1 (size1)

SELECTION

Geometric entity level	Point
Selection	Geometry geom1: Dimension 0: Points 5–6

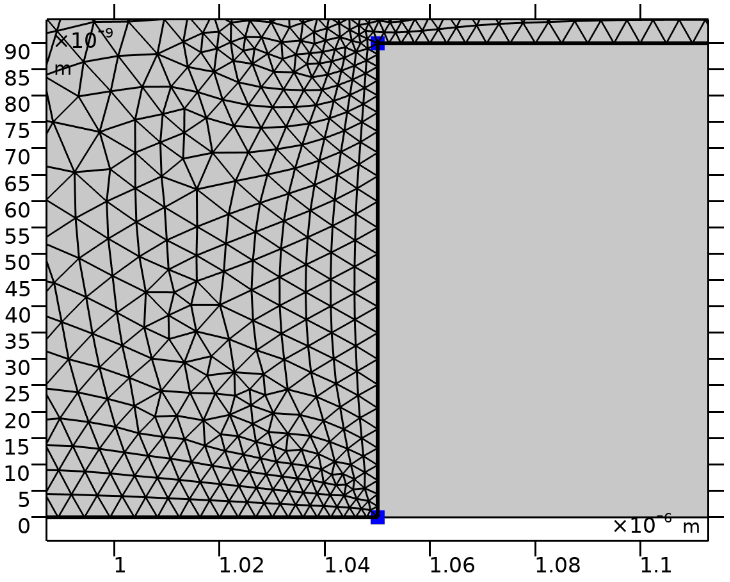

Size 1

SETTINGS

Description	Value
Maximum element size	2E-9
Minimum element size	3.06E-7
Minimum element size	Off
Curvature factor	0.3
Curvature factor	Off
Resolution of narrow regions	Off
Maximum element growth rate	1.3
Maximum element growth rate	Off
Custom element size	Custom

2.7.3 Size 2 (size2)

SELECTION

Geometric entity level	Boundary
Selection	Geometry geom1: Dimension 1: Boundaries 2, 7, 9

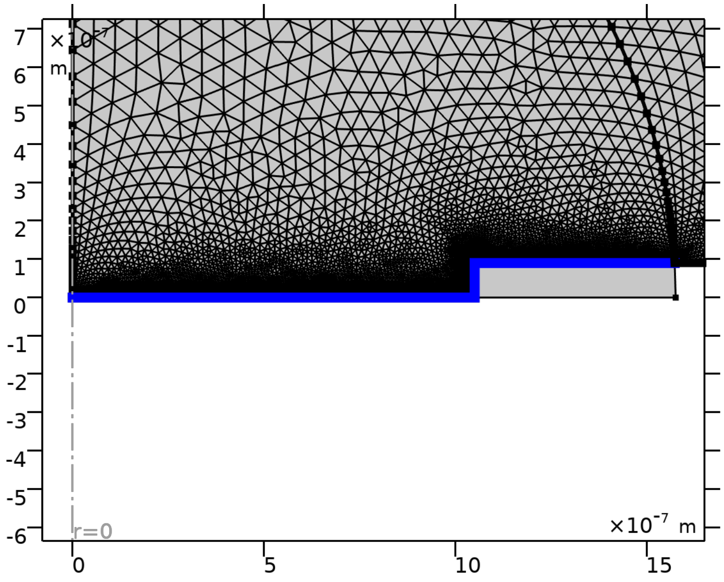

Size 2

SETTINGS

Description	Value
Maximum element size	5E-9
Minimum element size	3.06E-7
Minimum element size	Off
Curvature factor	0.3
Curvature factor	Off
Resolution of narrow regions	Off
Maximum element growth rate	1.3
Maximum element growth rate	Off
Custom element size	Custom

2.7.4 Size 3 (size3)

SELECTION

Geometric entity level	Domain
Selection	Geometry geom1: Dimension 2: Domain 1

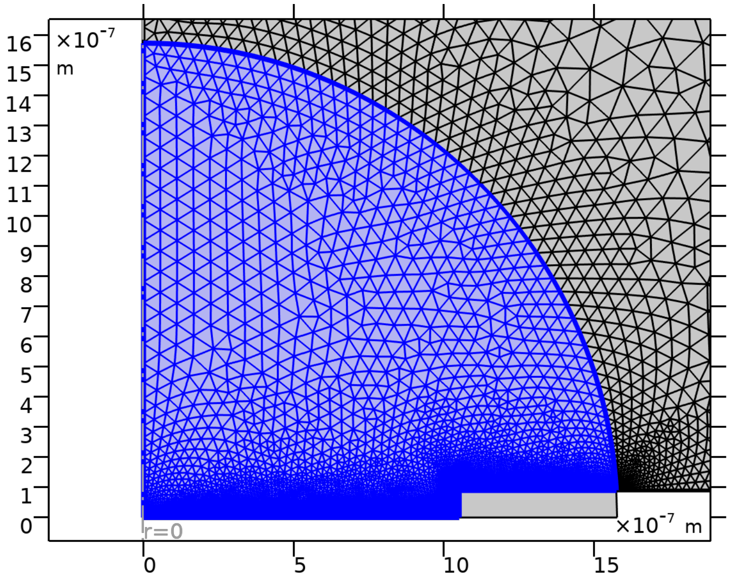

Size 3

SETTINGS

Description	Value
Maximum element size	7E-8
Minimum element size	1E-9
Curvature factor	0.25
Maximum element growth rate	1.12
Predefined size	Extra fine
Custom element size	Custom

2.7.5 Size 7 (size7)

SELECTION

Geometric entity level	Boundary
Selection	Geometry geom1: Dimension 1: Boundaries 16–17

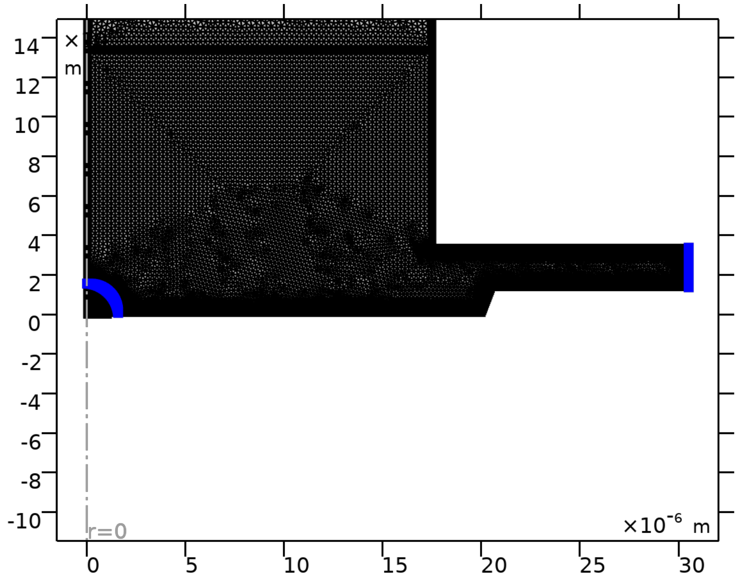

Size 7

SETTINGS

Description	Value
Maximum element size	5E-8
Minimum element size	3.15E-7
Minimum element size	Off
Curvature factor	0.3
Curvature factor	Off
Resolution of narrow regions	Off
Maximum element growth rate	1.3
Maximum element growth rate	Off
Custom element size	Custom

2.7.6 Size 4 (size4)

SELECTION

Geometric entity level	Boundary
Selection	Geometry geom1: Dimension 1: Boundaries 10, 12, 14–15

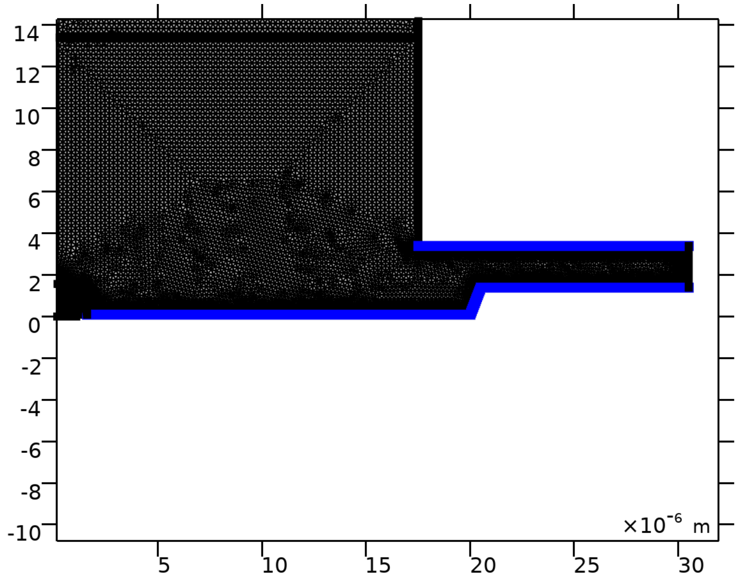

Size 4

SETTINGS

Description	Value
Maximum element size	1E-7
Minimum element size	3.06E-7
Minimum element size	Off
Curvature factor	0.3
Curvature factor	Off
Resolution of narrow regions	Off
Maximum element growth rate	1.3
Maximum element growth rate	Off
Custom element size	Custom

2.7.7 Size 5 (size5)

SELECTION

Geometric entity level	Domain
Selection	Geometry geom1: Dimension 2: Domain 2

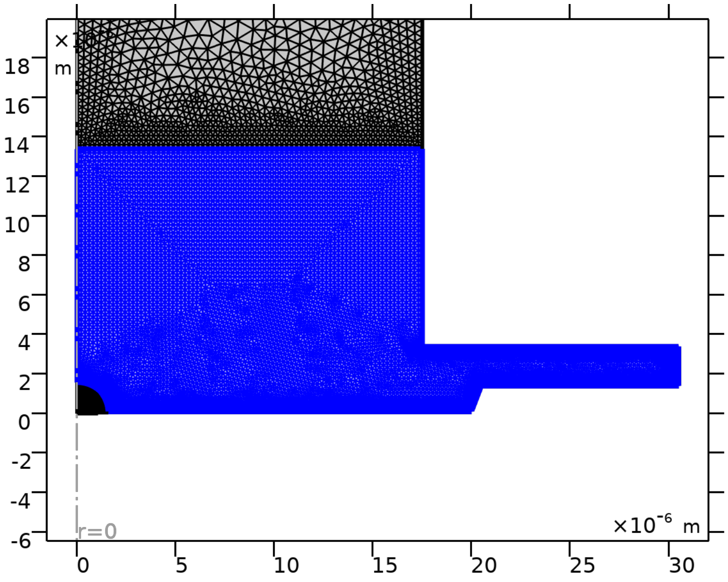

Size 5

SETTINGS

Description	Value
Maximum element size	2.5E-7
Minimum element size	3.06E-7
Minimum element size	Off
Curvature factor	0.3
Curvature factor	Off
Resolution of narrow regions	Off
Maximum element growth rate	1.15
Custom element size	Custom

2.7.8 Free Triangular 1 (ftri1)

SELECTION

Geometric entity level	Domain
Selection	Geometry geom1: Dimension 2: Domains 1–3

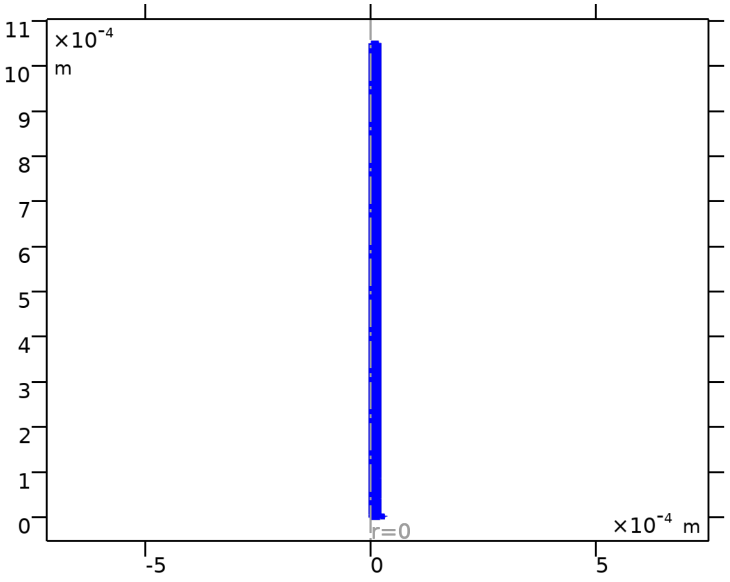

Free Triangular 1

SETTINGS

Description	Value
Last build time	1
Built with	COMSOL 6.1.0.357 (win64)|2023 - 12 - 20T13:59:36.884379300

3 Study 1

COMPUTATION INFORMATION

Computation time	3 min 8 s

3.1 TIME DEPENDENT

Times	Unit
range(0,0.5,t_acc+t_step)	s

STUDY SETTINGS

Description	Value
Include geometric nonlinearity	Off

STUDY SETTINGS

Description	Value
Output times	{0, 0.5, 1, 1.5, 2, 2.5, 3, 3.5, 4, 4.5, 5, 5.5, 6, 6.5, 7, 7.5, 8, 8.5, 9, 9.5, 10, 10.5, 11, 11.5, 12, 12.5, 13, 13.5, 14, 14.5, 15, 15.5, 16, 16.5, 17, 17.5, 18, 18.5, 19, 19.5, 20, 20.5, 21, 21.5, 22, 22.5, 23, 23.5, 24, 24.5, 25}

PHYSICS AND VARIABLES SELECTION

Physics interface	Solve for	Equation form
Transport of Diluted Species (tds)	On	Automatic (Time dependent)
Creeping Flow (spf)	On	Automatic (Time dependent)
Events (ev)	On	Automatic (Time dependent)

PHYSICS AND VARIABLES SELECTION

Multiphysics couplings	Solve for	Equation form
Flow Coupling 1 (fc1)	On	Automatic (Time dependent)

MESH SELECTION

Component	Mesh
Component 1	Mesh 1

3.2 SOLVER CONFIGURATIONS

3.2.1 Solution 1

Compile Equations: Time Dependent (st1)

STUDY AND STEP

Description	Value
Use study	Study 1

Description	Value
Use study step	Time Dependent

Dependent Variables 1 (v1)

GENERAL

Description	Value
Defined by study step	Time Dependent

RESIDUAL SCALING

Description	Value
Method	Manual

INITIAL VALUE CALCULATION CONSTANTS

Constant name	Initial value source
t	range(0,0.5,t_acc+t_step)
timestep	0.025[s]

Concentration (comp1.cO) (comp1_cO)

GENERAL

Description	Value
Field components	comp1.cO
Internal variables	{comp1.uflux.cO, comp1.dflux.cO, comp1.tds.dt2Inv_cO}

Concentration (comp1.cR) (comp1_cR)

GENERAL

Description	Value
Field components	comp1.cR
Internal variables	{comp1.uflux.cR, comp1.dflux.cR, comp1.tds.dt2Inv_cR}

Pressure (comp1.p) (comp1_p)

GENERAL

Description	Value
Field components	comp1.p

Velocity field (comp1.u) (comp1_u)

GENERAL

Description	Value
Field components	{comp1.u, comp1.w}
Internal variables	{comp1.spf.dt2Inv_u, comp1.spf.isFluidHasBeenSolved}

Discrete state (comp1.ev.ds1.dim) (comp1_ev_ds1_dim)

GENERAL

Description	Value
State components	comp1.cellE

Time-Dependent Solver 1 (t1)

GENERAL

Description	Value
Defined by study step	Time Dependent
Output times	{0, 0.5, 1, 1.5, 2, 2.5, 3, 3.5, 4, 4.5, 5, 5.5, 6, 6.5, 7, 7.5, 8, 8.5, 9, 9.5, 10, 10.5, 11, 11.5, 12, 12.5, 13, 13.5, 14, 14.5, 15, 15.5, 16, 16.5, 17, 17.5, 18, 18.5, 19, 19.5, 20, 20.5, 21, 21.5, 22, 22.5, 23, 23.5, 24, 24.5, 25}
Relative tolerance	0.005

ABSOLUTE TOLERANCE

Description	Value
Tolerance factor	0.05

ABSOLUTE TOLERANCE

Field	Method	Tolerance method	Tolerance factor	Derivative tolerance method	Tolerance for time derivatives	Tolerance	Tolerance for time derivatives
Concentration (comp1.cO)	Use global	Factor	0.1	Automatic	1	0.001	0.001
Concentration (comp1.cR)	Use global	Factor	0.1	Automatic	1	0.001	0.001
Pressure (comp1.p)	Scaled	Factor	1	Automatic	1	0.001	0.001
Velocity field (comp1.u)	Use global	Factor	0.1	Automatic	1	0.001	0.001
Discrete state (comp1.ev.ds1.dim)	Use global	Factor	0.1	Automatic	1	0.001	0.001

TIME STEPPING

Description	Value

Description	Value
Maximum BDF order	2
Nonlinear controller	On
Fraction of initial step for Backward Euler	0.01
Error estimation	Exclude algebraic

Advanced (aDef)

ASSEMBLY SETTINGS

Description	Value
Reuse sparsity pattern	On

Fully Coupled 1 (fc1)

GENERAL

Description	Value
Linear solver	Direct (merged)

METHOD AND TERMINATION

Description	Value
Damping factor	0.9
Jacobian update	Once per time step
Maximum number of iterations	8
Tolerance factor	0.5
Stabilization and acceleration	Anderson acceleration
Dimension of iteration space	5
Mixing parameter	0.9

3.2.2 Parametric Solutions 1

rdrop=1E-5 (su1)

GENERAL

Description	Value
Solution	rdrop=1E-5

rdrop=2E-5 (su2)

GENERAL

Description	Value
Solution	rdrop=2E-5

rdrop=1E-4 (su3)

GENERAL

Description	Value
Solution	rdrop=1E-4

rdrop=3E-4 (su4)

GENERAL

Description	Value
Solution	rdrop=3E-4

3.2.3 Parametric Solutions 2

de=0.1 (su1)

GENERAL

Description	Value
Solution	de=0.1

de=0.2 (su2)

GENERAL

Description	Value
Solution	de=0.2

de=0.4 (su3)

GENERAL

Description	Value
Solution	de=0.4

de=0.6 (su4)

GENERAL

Description	Value
Solution	de=0.6

de=0.8 (su5)

GENERAL

Description	Value
Solution	de=0.8

de=1 (su6)

GENERAL

Description	Value
Solution	de=1

de=1.2 (su7)

GENERAL

Description	Value
Solution	de=1.2

de=1.4 (su8)

GENERAL

Description	Value
Solution	de=1.4

de=1.6 (su9)

GENERAL

Description	Value
Solution	de=1.6

3.2.4 Parametric Solutions 3

hpipet=2E-6 (su1)

GENERAL

Description	Value
Solution	hpipet=2E-6

hpipet=4E-6 (su2)

GENERAL

Description	Value
Solution	hpipet=4E-6

hpipet=7E-6 (su3)

GENERAL

Description	Value
Solution	hpipet=7E-6

hpipet=1.4E-5 (su4)

GENERAL

Description	Value
Solution	hpipet=1.4E-5

hpipet=2E-5 (su5)

GENERAL

Description	Value
Solution	hpipet=2E-5

hpipet=3E-5 (su6)

GENERAL

Description	Value
Solution	hpipet=3E-5

3.2.5 Parametric Solutions 4

hpipet=2E-6 (2) (su1)

GENERAL

Description	Value
Solution	hpipet=2E-6 (2)

hpipet=4E-6 (2) (su2)

GENERAL

Description	Value
Solution	hpipet=4E-6 (2)

hpipet=7E-6 (2) (su3)

GENERAL

Description	Value
Solution	hpipet=7E-6 (2)

hpipet=1E-5 (su4)

GENERAL

Description	Value
Solution	hpipet=1E-5

hpipet=1.5E-5 (su5)

GENERAL

Description	Value
Solution	hpipet=1.5E-5

hpipet=2E-5 (2) (su6)

GENERAL

Description	Value
Solution	hpipet=2E-5 (2)

hpipet=3E-5 (2) (su7)

GENERAL

Description	Value
Solution	hpipet=3E-5 (2)

3.2.6 Parametric Solutions 5

re=1.4E-6 (su1)

GENERAL

Description	Value
Solution	re=1.4E-6

re=2.75E-6 (su2)

GENERAL

Description	Value
Solution	re=2.75E-6

re=4E-6 (su3)

GENERAL

Description	Value
Solution	re=4E-6

3.2.7 Parametric Solutions 6

re=1.4E-6 (2) (su1)

GENERAL

Description	Value
Solution	re=1.4E-6 (2)

re=2.75E-6 (2) (su2)

GENERAL

Description	Value
Solution	re=2.75E-6 (2)

re=4E-6 (2) (su3)

GENERAL

Description	Value
Solution	re=4E-6 (2)

3.2.8 Parametric Solutions 7

re=1.05E-6 (su1)

GENERAL

Description	Value
Solution	re=1.05E-6

re=2.2E-6 (su2)

GENERAL

Description	Value
Solution	re=2.2E-6

re=3.3E-6 (su3)

GENERAL

Description	Value
Solution	re=3.3E-6

4 Results

4.1 DATA SETS

4.1.1 Study 1/Solution 1 (1)

SOLUTION

Description	Value
Solution	Solution 1
Component	Component 1 (comp1)
Frame	Material (R, PHI, Z)

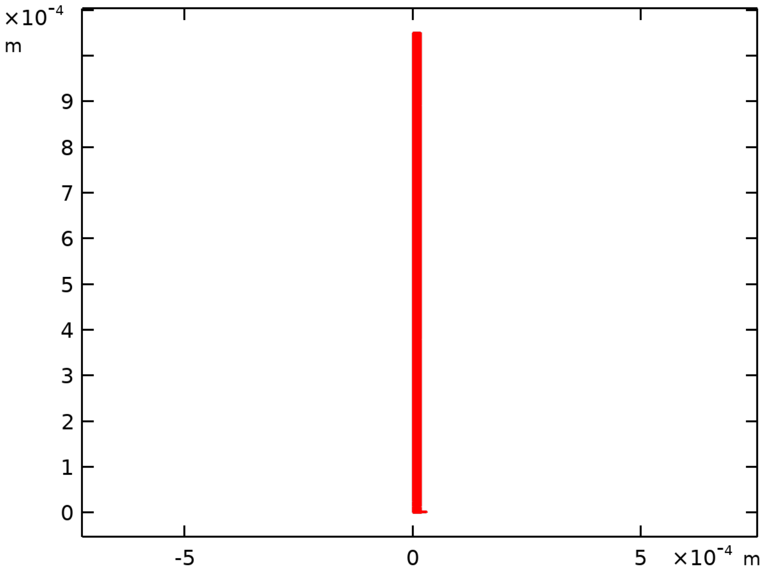

Dataset: Study 1/Solution 1 (1)

4.1.2 Revolution 2D

DATA

Description	Value
Dataset	Study 1/Solution 1 (1)

AXIS DATA

Description	Value
Axis entry method	Two points
Points	{{0, 0}, {0, 1}}

REVOLUTION LAYERS

Description	Value

Description	Value
Start angle	-90
Revolution angle	225

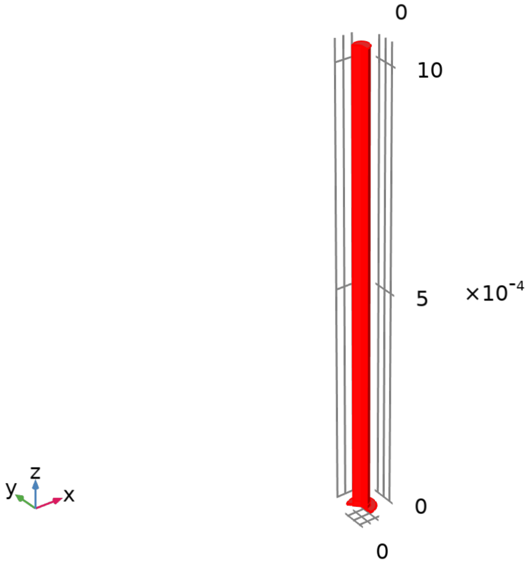

Dataset: Revolution 2D

4.1.3 Study 1/Parametric Solutions 1

SOLUTION

Description	Value
Solution	Parametric Solutions 1
Component	Component 1 (comp1)
Frame	Material (R, PHI, Z)

4.1.4 Revolution 2D 1

DATA

Description	Value
Dataset	Study 1/Parametric Solutions 1

AXIS DATA

Description	Value
Axis entry method	Two points
Points	{{0, 0}, {0, 1}}

REVOLUTION LAYERS

Description	Value

Description	Value
Start angle	-90
Revolution angle	225

4.1.5 Mesh 1

MESH

Description	Value
Mesh	Mesh 1

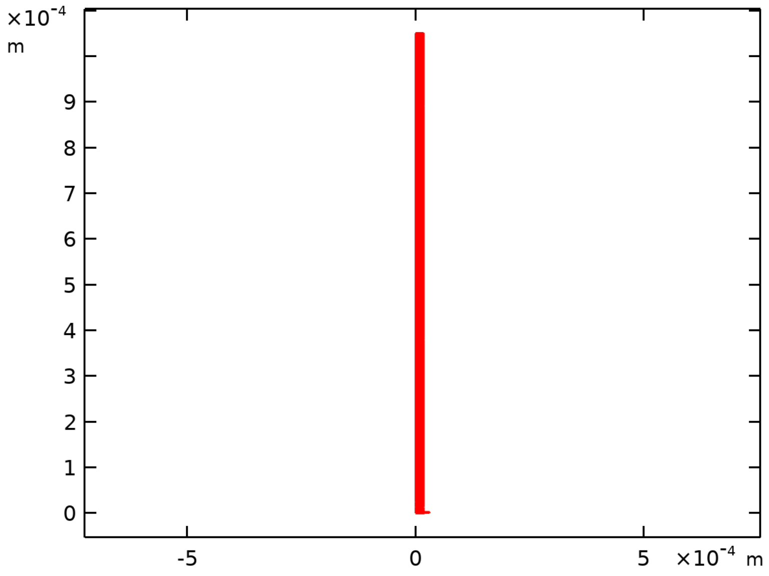

Dataset: Mesh 1

4.1.6 Study 1/Parametric Solutions 2

SOLUTION

Description	Value
Solution	Parametric Solutions 2
Component	Component 1 (comp1)
Frame	Material (R, PHI, Z)

4.1.7 Revolution 2D 2

DATA

Description	Value
Dataset	Study 1/Parametric Solutions 2

AXIS DATA

Description	Value

Description	Value
Axis entry method	Two points
Points	{{0, 0}, {0, 1}}

REVOLUTION LAYERS

Description	Value
Start angle	-90
Revolution angle	225

4.1.8 Study 1/Parametric Solutions 3

SOLUTION

Description	Value
Solution	Parametric Solutions 3
Component	Component 1 (comp1)
Frame	Material (R, PHI, Z)

4.1.9 Revolution 2D 3

DATA

Description	Value
Dataset	Study 1/Parametric Solutions 3

AXIS DATA

Description	Value
Axis entry method	Two points
Points	{{0, 0}, {0, 1}}

REVOLUTION LAYERS

Description	Value
Start angle	-90
Revolution angle	225

4.1.10 Study 1/Parametric Solutions 4

SOLUTION

Description	Value
Solution	Parametric Solutions 4
Component	Component 1 (comp1)
Frame	Material (R, PHI, Z)

4.1.11 Revolution 2D 4

DATA

Description	Value
Dataset	Study 1/Parametric Solutions 4

AXIS DATA

Description	Value
Axis entry method	Two points
Points	{{0, 0}, {0, 1}}

REVOLUTION LAYERS

Description	Value
Start angle	-90
Revolution angle	225

4.1.12 Study 1/Parametric Solutions 5

SOLUTION

Description	Value
Solution	Parametric Solutions 5
Component	Component 1 (comp1)

4.1.13 Revolution 2D 5

DATA

Description	Value
Dataset	Study 1/Parametric Solutions 5

AXIS DATA

Description	Value
Axis entry method	Two points
Points	{{0, 0}, {0, 1}}

REVOLUTION LAYERS

Description	Value
Start angle	-90
Revolution angle	225

4.1.14 Study 1/Parametric Solutions 6

SOLUTION

Description	Value
Solution	Parametric Solutions 6
Component	Component 1 (comp1)

4.1.15 Revolution 2D 6

DATA

Description	Value
Dataset	Study 1/Parametric Solutions 6

AXIS DATA

Description	Value
Axis entry method	Two points
Points	{{0, 0}, {0, 1}}

REVOLUTION LAYERS

Description	Value
Start angle	-90
Revolution angle	225

4.1.16 Study 1/Parametric Solutions 7

SOLUTION

Description	Value
Solution	Parametric Solutions 7
Component	Component 1 (comp1)

4.1.17 Revolution 2D 7

DATA

Description	Value
Dataset	Study 1/Parametric Solutions 7

AXIS DATA

Description	Value
Axis entry method	Two points
Points	{{0, 0}, {0, 1}}

REVOLUTION LAYERS

Description	Value
Start angle	-90

Description	Value
Revolution angle	225

4.1.18 Study 1/Solution 1 (10)

SOLUTION

Description	Value
Solution	Solution 1
Component	Component 1 (comp1)

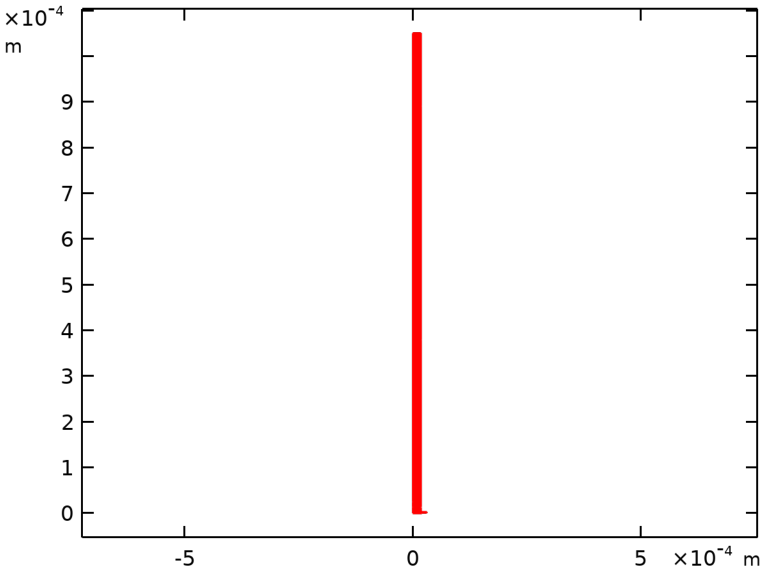

Dataset: Study 1/Solution 1 (10)

4.1.19 Revolution 2D 8

DATA

Description	Value
Dataset	Study 1/Solution 1 (1)

AXIS DATA

Description	Value
Axis entry method	Two points
Points	{{0, 0}, {0, 1}}

REVOLUTION LAYERS

Description	Value
Start angle	-90
Revolution angle	225

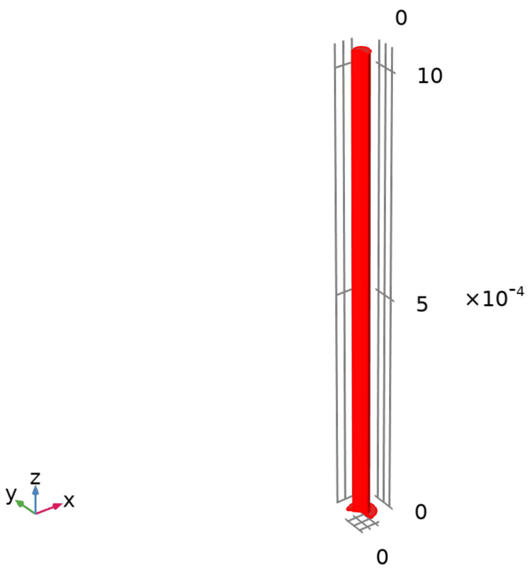

Dataset: Revolution 2D 8

4.2 PLOT GROUPS

4.2.1 1D Plot Group 3

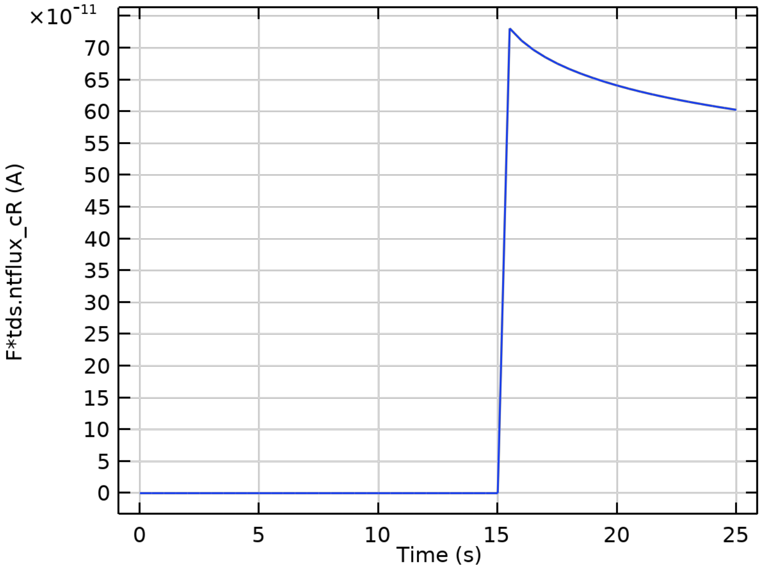

4.2.2 Concentration, R (tds)

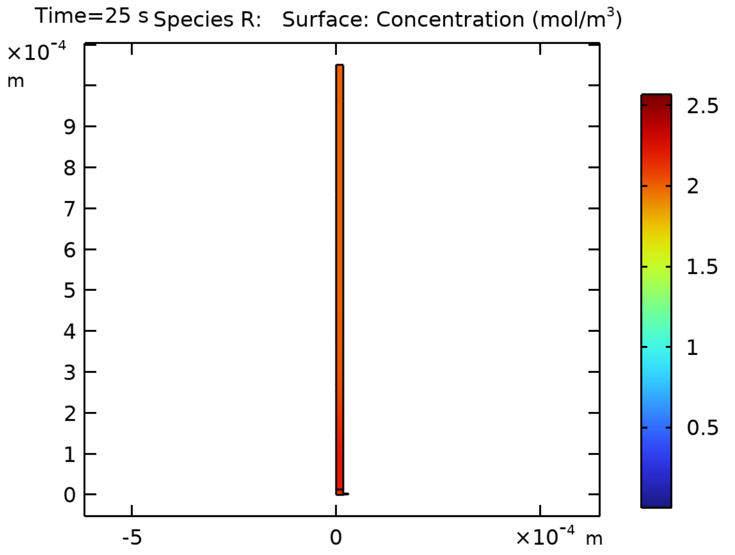

Species R: Surface: Concentration (mol/m³)

4.2.3 Velocity (spf)

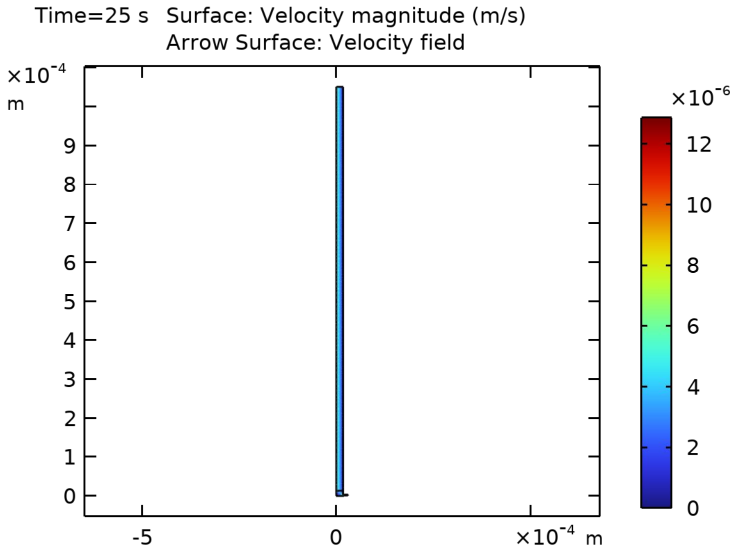

Surface: Velocity magnitude (m/s) Arrow Surface: Velocity field

4.3 EVALUATION GROUPS

4.3.1 Evaluation Group 1

DATA

Description	Value

Description	Value
Dataset	Study 1/Solution 1 (1)

FEATURES

Feature	Column
Line Integration 1	Time (s)

RESULTS

Time (s)	F*tds.ntflux_cR (A)
0	7.8666E-15
0.5	4.5898E-15
1	4.5704E-15
1.5	4.5665E-15
2	4.5452E-15
2.5	4.5343E-15
3	4.5227E-15
3.5	4.5077E-15
4	4.4965E-15
4.5	4.4853E-15
5	4.4748E-15
5.5	4.4649E-15
6	4.456E-15
6.5	4.4479E-15
7	4.4383E-15
7.5	4.4302E-15
8	4.4225E-15
8.5	4.4153E-15
9	4.4086E-15
9.5	4.4009E-15
10	4.3943E-15
10.5	4.3881E-15
11	4.3822E-15
11.5	4.3766E-15
12	4.3701E-15
12.5	4.3644E-15
13	4.359E-15
13.5	4.3538E-15

Time (s)	F*tds.ntflux_cR (A)
14	4.3488E-15
14.5	4.3436E-15
15	4.3388E-15
15.5	7.3081E-10
16	7.1148E-10
16.5	6.9697E-10
17	6.853E-10
17.5	6.7539E-10
18	6.6688E-10
18.5	6.5941E-10
19	6.5273E-10
19.5	6.4662E-10
20	6.4105E-10
20.5	6.3592E-10
21	6.3121E-10
21.5	6.2685E-10
22	6.2277E-10
22.5	6.1898E-10
23	6.1539E-10
23.5	6.1202E-10
24	6.0877E-10
24.5	6.0568E-10
25	6.0276E-10

Line Integration 1

EXPRESSIONS

Expression	Unit	Description
F*tds.ntflux_cR	A	

INTEGRATION SETTINGS

Description	Value
Integration order	4
Compute surface integral	On
