# Supplementary material for: High-Throughput Single-Entity Electrochemistry with Microelectrode Arrays
Source: Anal Chem. 2024 May 23;96(22):9177–84. doi: 10.1021/acs.analchem.4c01092 (PMC11154736; doi:10.1021/acs.analchem.4c01092)
Supplement: Supplementary file 3 — ac4c01092_si_003.pdf [file ac4c01092_si_003.pdf]

AMCM 3D Chronoamperometry with particle

Report date	Jan 27, 2024, 1:19:56 PM

Contents

1. Global Definitions 3
1.1. Parameters 3
1.2. Shared Properties 4
2. Component 2 5
2.1. Definitions 5
2.2. Geometry 2 5
2.3. Transport of Diluted Species 9
2.4. Creeping Flow 30
2.5. Events 58
2.6. Multiphysics 59
2.7. Mesh 2 60
3. Study 1 67
3.1. Time Dependent 67
3.2. Solver Configurations 68
4. Results 75
4.1. Data Sets 75
4.2. Plot Groups 76
4.3. Evaluation Groups 77

1 Global Definitions

Date	Jan 27, 2024, 9:34:02 AM

GLOBAL SETTINGS

Name	ST 2024-01-027 001 AMCM3D withblocker edit report.mph
Path	C:\COMSOL Projects\2022_AMCM\ST_2024-01-027_001_AMCM3D_withblocker_edit_report.mph
Version	COMSOL Multiphysics 6.1 (Build: 357)

USED PRODUCTS

COMSOL Multiphysics
Electrochemistry Module

COMPUTER INFORMATION

CPU	Intel64 Family 6 Model 60 Stepping 3, 4 cores, 31.88 GB RAM
Operating system	Windows 10

1.1 PARAMETERS

PARAMETERS 1

**Name**	**Expression**	**Value**	**Description**
pipetshank	1E-3 [m]	0.001 m	length of pipet simulated
rpipet	24E-6 [m]	2.4E−5 m	radius of droplet
pipetOR	41 [um]	4.1E−5 m	pipetOR
de	0.09 [um]	9E−8 m	disk recession
blockerlift	1E-9 [m]	1E−9 m	blocker lift for easier meshing
rblocker	0.25 [um]	2.5E−7 m	blocker radius
re	1.05E-6 [m]	1.05E−6 m	disk radius
theta	0 [deg]	0 rad	recession angle
hpipet	2 [um]	2E−6 m	Dps
Ecell	0.3	0.3	electrode potential
Ef	0 [V]	0 V	formal potential
cRbulk	0.002 [M]	2 mol/m^3	bulk conc of R
F	9.64853E4 [C/mol]	96485 C/mol	Faraday constant
f	38.92 [1/V]	38.92 1/V	F/RT
DR	7.4E-6 [(cm^2)/s]	7.4E−10 m^2/s	diffusion coefficient of R
DO	DR	7.4E−10 m^2/s	diff co of O
a	0.5	0.5	alpha
k0	10 [cm/s]	0.1 m/s	e-t rate constant

Name	Expression	Value	Description
offset	0 [um]	0 m	MEA center offset
v_dry	6 [um/s]	6E−6 m/s	drying velocity at meniscus
photorad	20 [um]	2E−5 m	radius of photoresist hole
photodepth	1.3 [um]	1.3E−6 m	depth of photoresist hole
photoOR	photorad + 1[um]	2.1E−5 m	upper radius of photoresist hole
blockloc	0 [um]	0 m	blocker center location
block_z	0	0	use to place sphere onto SiNx insulation

1.2 SHARED PROPERTIES

1.2.1 Default Model Inputs

Tag	cminpt

2 Component 2

SETTINGS

Description	Value
Unit system	Same as global system (SI)
Avoid inverted elements by curving interior domain elements	Off

2.1 DEFINITIONS

2.1.1 Coordinate Systems

Boundary System 2

Coordinate system type	Boundary system
Tag	sys1

COORDINATE NAMES

First	Second	Third
t1	t2	n

2.2 GEOMETRY 2

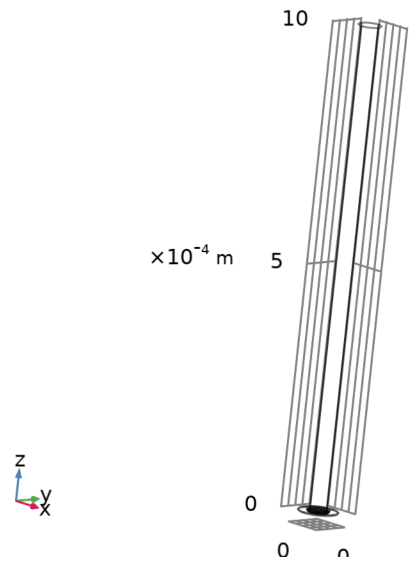

Geometry 2

UNITS

Length unit	m
Angular unit	deg

GEOMETRY STATISTICS

Description	Value
Space dimension	3
Number of domains	7
Number of boundaries	63
Number of edges	112
Number of vertices	59

2.2.1 Cylinder 1 (cyl1)

POSITION

Description	Value
Position	{0, 0, de+photodepth}

AXIS

Description	Value
Axis type	z - axis

SIZE AND SHAPE

Description	Value
Radius	pipetOR
Height	hpipet

2.2.2 Cone 2 (cone2)

POSITION

Description	Value
Position	{0, 0, de+hpipet + photodepth}

AXIS

Description	Value
Axis type	z - axis

SIZE AND SHAPE

Description	Value
Bottom radius	rpipet
Height	pipetshank
Specify top size using	Radius
Top radius	rpipet

2.2.3 Cylinder 3 (cyl3)

POSITION

Description	Value
Position	{0, 0, de+photodepth}

AXIS

Description	Value
Axis type	z - axis

SIZE AND SHAPE

Description	Value
Radius	photorad + 2E-6
Height	hpipet

2.2.4 Cylinder 4 (cyl4)

POSITION

Description	Value
Position	{0, 0, hpipet + de+photodepth}

AXIS

Description	Value
Axis type	z - axis

SIZE AND SHAPE

Description	Value
Radius	rpipet
Height	3E-6

2.2.5 Work Plane 1 (wp1)

PLANE DEFINITION

Description	Value
Plane	zx - plane

UNITE OBJECTS

Description	Value
Unite objects	On

Plane Geometry (sequence2D)

Polygon 1 (pol1)

OBJECT TYPE

Description	Value

Description	Value
Type	Solid

COORDINATES

Description	Value
Data source	Table

COORDINATES

xw (m)	yw (m)
0	0
0	re
de	re
de	re+1[um]
de+photodepth	re+1[um]
de+photodepth	0
0	0

Polygon 2 (pol2)

OBJECT TYPE

Description	Value
Type	Solid

COORDINATES

Description	Value
Data source	Table

COORDINATES

xw (m)	yw (m)
de	re+1[um]
de	photorad
de+photodepth	photoOR
de+photodepth	re+1[um]
de	re+1[um]

2.2.6 Revolve 1 (rev1)

SETTINGS

Description	Value
Work plane	Work Plane 1

REVOLUTION ANGLES

Description	Value
Angles	{0, 2*pi}
Type of specification	Full revolution
Keep original faces	Off

REVOLUTION AXIS

Description	Value
Point on the revolution axis	{0, 0}
Direction of revolution axis	{1, 0}
Revolution axis	{{0, 1}, {0, 0}}

2.2.7 Sphere 1 (sph1)

POSITION

Description	Value
Position	{0, blockloc + offset, rblocker + blockerlift + block_z}

AXIS

Description	Value
Axis type	z - axis

SIZE

Description	Value
Radius	rblocker

2.3 TRANSPORT OF DILUTED SPECIES

USED PRODUCTS

COMSOL Multiphysics
Electrochemistry Module

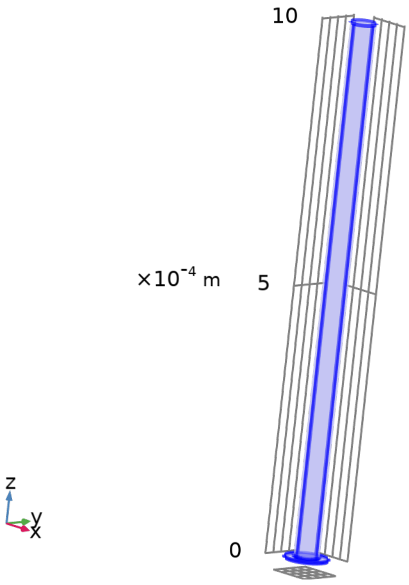

Transport of Diluted Species

SELECTION

Geometric entity level	Domain
Selection	Geometry geom2: Dimension 3: Domains 1–6

EQUATIONS

$$\frac{\partial c_i}{\partial t} + \nabla \cdot \mathbf{J}_i + \mathbf{u} \cdot \nabla c_i = R_i$$
$$\mathbf{J}_i = -D_i \nabla c_i$$

2.3.1 Interface Settings

Discretization

SETTINGS

Description	Value
Concentration	Linear

SETTINGS

Description	Value
Equation form	Study controlled

Species Activity

SETTINGS

Description	Value
Species activity	Ideal

Transport Mechanisms

SETTINGS

Description	Value
Convection	On
Migration in electric field	Off
Mass transfer in porous media	Off

2.3.2 Variables

Name	Expression	Unit	Description	Selection	Details
tds2.d	1	1	Out-of-plane geometry extension	Global	
tds2.f_cR	1	1	Activity coefficient	Domains 1–6	
tds2.f_cO	1	1	Activity coefficient	Domains 1–6	
tds2.nx	nx	1	Normal vector, x-component	Boundaries 7, 10, 12–13, 15, 18–19, 22–23, 26–27, 39, 41, 43, 45, 53, 56, 58, 60	
tds2.ny	ny	1	Normal vector, y-component	Boundaries 7, 10, 12–13, 15, 18–19, 22–23, 26–27, 39, 41, 43, 45, 53, 56, 58, 60	
tds2.nz	nz	1	Normal vector, z-component	Boundaries 7, 10, 12–13, 15, 18–19, 22–23, 26–27, 39, 41, 43, 45, 53, 56, 58, 60	
tds2.nx	unx	1	Normal vector, x-component	Boundaries 32–35, 48–50, 52	
tds2.ny	uny	1	Normal vector, y-component	Boundaries 32–35, 48–50, 52	
tds2.nz	unz	1	Normal vector, z-component	Boundaries 32–35, 48–50, 52	
tds2.nx	dnx	1	Normal vector, x-	Boundaries 1–6, 8–9, 11, 14,	

Name	Expression	Unit	Description	Selection	Details
			component	16–17, 20–21, 24–25, 28–31, 36–38, 40, 42, 44, 46–47, 51, 54–55, 57, 59, 61–63	
tds2.ny	dny	1	Normal vector, y-component	Boundaries 1–6, 8–9, 11, 14, 16–17, 20–21, 24–25, 28–31, 36–38, 40, 42, 44, 46–47, 51, 54–55, 57, 59, 61–63	
tds2.nz	dnz	1	Normal vector, z-component	Boundaries 1–6, 8–9, 11, 14, 16–17, 20–21, 24–25, 28–31, 36–38, 40, 42, 44, 46–47, 51, 54–55, 57, 59, 61–63	
tds2.nxmesh	nxmesh	1	Normal vector (mesh), x-component	Boundaries 7, 10, 12–13, 15, 18–19, 22–23, 26–27, 39, 41, 43, 45, 53, 56, 58, 60	
tds2.nymesh	nymesh	1	Normal vector (mesh), y-component	Boundaries 7, 10, 12–13, 15, 18–19, 22–23, 26–27, 39, 41, 43, 45, 53, 56, 58, 60	
tds2.nzmesh	nzmesh	1	Normal vector (mesh), z-component	Boundaries 7, 10, 12–13, 15, 18–19, 22–23, 26–27, 39, 41, 43, 45, 53, 56, 58, 60	
tds2.nxmesh	unxmesh	1	Normal vector (mesh), x-component	Boundaries 32–35, 48–50, 52	
tds2.nymesh	unymesh	1	Normal vector (mesh), y-	Boundaries 32–35, 48–50,	

Name	Expression	Unit	Description	Selection	Details
			component	52	
tds2.nzmesh	unzmesh	1	Normal vector (mesh), z-component	Boundaries 32–35, 48–50, 52	
tds2.nxmesh	dnxmesh	1	Normal vector (mesh), x-component	Boundaries 1–6, 8–9, 11, 14, 16–17, 20–21, 24–25, 28–31, 36–38, 40, 42, 44, 46–47, 51, 54–55, 57, 59, 61–63	
tds2.nymesh	dnymesh	1	Normal vector (mesh), y-component	Boundaries 1–6, 8–9, 11, 14, 16–17, 20–21, 24–25, 28–31, 36–38, 40, 42, 44, 46–47, 51, 54–55, 57, 59, 61–63	
tds2.nzmesh	dnzmesh	1	Normal vector (mesh), z-component	Boundaries 1–6, 8–9, 11, 14, 16–17, 20–21, 24–25, 28–31, 36–38, 40, 42, 44, 46–47, 51, 54–55, 57, 59, 61–63	
tds2.nxc	-root.nxc/tds2.ncLen	1	Normal vector, x-component	Boundaries 32–35, 48–50, 52	
tds2.nyc	-root.nyc/tds2.ncLen	1	Normal vector, y-component	Boundaries 32–35, 48–50, 52	
tds2.nzc	-root.nzc/tds2.ncLen	1	Normal vector, z-component	Boundaries 32–35, 48–50, 52	
tds2.nxc	root.nxc/tds2.ncLen	1	Normal vector, x-component	Boundaries 1–31, 36–47, 51, 53–63	
tds2.nyc	root.nyc/tds2.ncLen	1	Normal vector, y-component	Boundaries 1–31, 36–47, 51, 53–63	
tds2.nzc	root.nzc/tds2.ncLen	1	Normal vector, z-	Boundaries 1–	

Name	Expression	Unit	Description	Selection	Details
			component	31, 36–47, 51, 53–63	
tds2.ncLen	sqrt(root.nxc^2+root.nyc^2+root.nzc^2+eps)	1	Help variable	Boundaries 1–63	
tds2.cbf_cR	0	mol/(m²·s)	Convective boundary flux	Boundaries 1–63	
tds2.u	0	m/s	Velocity field, x-component	Domains 1–6	
tds2.v	0	m/s	Velocity field, y-component	Domains 1–6	
tds2.w	0	m/s	Velocity field, z-component	Domains 1–6	
tds2.cbf_cO	0	mol/(m²·s)	Convective boundary flux	Boundaries 1–63	
tds2.R_cR	0	mol/(m³·s)	Total rate expression	Domains 1–6	+ operation
tds2.cP_cR	0	mol/kg	Concentration species adsorbed to the solid	Domains 1–6	+ operation
tds2.cP_cR	0	mol/kg	Concentration species adsorbed to the solid	Boundaries 1–63	+ operation
tds2.KP_cR	0	m³/kg	Adsorption isotherm, first concentration derivative	Domains 1–6	+ operation
tds2.KP_cR	0	m³/kg	Adsorption isotherm, first concentration derivative	Boundaries 1–63	+ operation
tds2.Rads_cR	0	mol/(m³·s)	Total adsorption rate	Domains 1–6	+ operation
tds2.DiT_cR	0	m²/s	Turbulent diffusivity	Domains 1–6	
tds2.cVar_cR	cR	mol/m³	Species	Boundaries 1–63	
tds2.cVar_cR	cR	mol/m³	Species	Edges 1–112	
tds2.cVar_cR	cR	mol/m³	Species	Points 1–59	
tds2.R_cO	0	mol/(m³·s)	Total rate expression	Domains 1–6	+ operation

Name	Expression	Unit	Description	Selection	Details
tds2.cP_cO	0	mol/kg	Concentration species adsorbed to the solid	Domains 1–6	+ operation
tds2.cP_cO	0	mol/kg	Concentration species adsorbed to the solid	Boundaries 1–63	+ operation
tds2.KP_cO	0	m^3/kg	Adsorption isotherm, first concentration derivative	Domains 1–6	+ operation
tds2.KP_cO	0	m^3/kg	Adsorption isotherm, first concentration derivative	Boundaries 1–63	+ operation
tds2.Rads_cO	0	mol/(m^3·s)	Total adsorption rate	Domains 1–6	+ operation
tds2.DiT_cO	0	m^2/s	Turbulent diffusivity	Domains 1–6	
tds2.cVar_cO	cO	mol/m^3	Species	Boundaries 1–63	
tds2.cVar_cO	cO	mol/m^3	Species	Edges 1–112	
tds2.cVar_cO	cO	mol/m^3	Species	Points 1–59	
tds2.poro	1	1	Porosity	Domains 1–6	
tds2.theta_g	0	1	Gas volume fraction	Domains 1–6	
tds2.theta_l	1	1	Liquid volume fraction	Domains 1–6	
tds2.theta	tds2.poro	1	Mobile fluid volume fraction	Domains 1–6	

2.3.3 Transport Properties 1

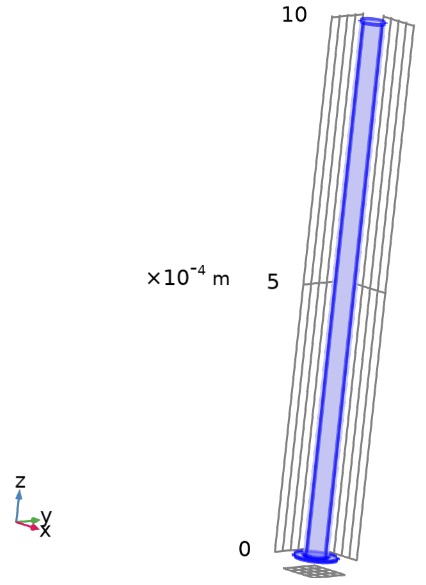

Transport Properties 1

SELECTION

Geometric entity level	Domain
Selection	Geometry geom2: Dimension 3: All domains

EQUATIONS

$$\frac{\partial c_i}{\partial t} + \nabla \cdot \mathbf{J}_i + \mathbf{u} \cdot \nabla c_i = R_i$$

$$\mathbf{J}_i = -D_i \nabla c_i$$

Diffusion

SETTINGS

**Description**	**Value**	**Unit**
Source	Material	
Material	None	
Diffusion coefficient	User defined	
Diffusion coefficient	DR	m^2/s
Diffusion coefficient	User defined	
Diffusion coefficient	DO	m^2/s

Coordinate System Selection

SETTINGS

**Description**	**Value**

Description	Value
Coordinate system	Global coordinate system

Model Input

SETTINGS

Description	Value
Temperature	Common model input

Variables

Name	Expression	Unit	Description	Selection	Details
domflux.cRx	tds2.dflux_cRx*tds2.d	mol/(m²·s)	Domain flux, x-component	Domains 1–6	
domflux.cRy	tds2.dflux_cRy*tds2.d	mol/(m²·s)	Domain flux, y-component	Domains 1–6	
domflux.cRz	tds2.dflux_cRz*tds2.d	mol/(m²·s)	Domain flux, z-component	Domains 1–6	
domflux.cOx	tds2.dflux_cOx*tds2.d	mol/(m²·s)	Domain flux, x-component	Domains 1–6	
domflux.cOy	tds2.dflux_cOy*tds2.d	mol/(m²·s)	Domain flux, y-component	Domains 1–6	
domflux.cOz	tds2.dflux_cOz*tds2.d	mol/(m²·s)	Domain flux, z-component	Domains 1–6	
tds2.ndflux_cR	tds2.bndFlux_cR	mol/(m²·s)	Normal diffusive flux	Boundaries 1–63	
tds2.ncflux_cR	tds2.cflux_cRx*tds2.nxc+tds2.cflux_cRy*tds2.nyc+tds2.cflux_cRz*tds2.nzc	mol/(m²·s)	Normal convective flux	Boundaries 1–63	
tds2.ntflux_cR	tds2.bndFlux_cR+tds2.cflux_cRx*tds2.nxc+tds2.cflux_cRy*tds2.nyc+tds2.cflux_cRz*tds2.nzc	mol/(m²·s)	Normal total flux	Boundaries 1–63	
tds2.ndflux_cO	tds2.bndFlux_cO	mol/(m²·s)	Normal diffusive flux	Boundaries 1–63	
tds2.ncflux_cO	tds2.cflux_cOx*tds2.nxc+tds2.cflux_cOy*tds2.nyc+tds2.cflux_cOz*tds2.nzc	mol/(m²·s)	Normal convective flux	Boundaries 1–63	
tds2.ntflux_cO	tds2.bndFlux_cO+tds2.cflux_cOx*tds2.nxc+tds2.cflux_cOy	mol/(m²·s)	Normal total flux	Boundaries 1–63	

Name	Expression	Unit	Description	Selection	Details
	*tds2.nyc+tds2.cflux_cOz*tds2.nzc				
tds2.u	model.input.u1	m/s	Velocity field, x-component	Domains 1–6	Meta
tds2.v	model.input.u2	m/s	Velocity field, y-component	Domains 1–6	Meta
tds2.w	model.input.u3	m/s	Velocity field, z-component	Domains 1–6	Meta
tds2.bndFlux_cR	0.5*(uflux_spatial(cR)-dflux_spatial(cR))/tds2.d	mol/(m²·s)	Boundary flux	Boundaries 7, 10, 12–13, 15, 18–19, 22–23, 26–27, 39, 41, 43, 45, 53, 56, 58, 60	Meta
tds2.bndFlux_cR	-uflux_spatial(cR)/tds2.d	mol/(m²·s)	Boundary flux	Boundaries 32–35, 48–50, 52	Meta
tds2.bndFlux_cR	-dflux_spatial(cR)/tds2.d	mol/(m²·s)	Boundary flux	Boundaries 1–6, 8–9, 11, 14, 16–17, 20–21, 24–25, 28–31, 36–38, 40, 42, 44, 46–47, 51, 54–55, 57, 59, 61–63	Meta
tds2.bndFlux_cO	0.5*(uflux_spatial(cO)-dflux_spatial(cO))/tds2.d	mol/(m²·s)	Boundary flux	Boundaries 7, 10, 12–13, 15, 18–19, 22–23, 26–27, 39, 41, 43, 45, 53, 56, 58, 60	Meta
tds2.bndFlux_cO	-uflux_spatial(cO)/tds2.d	mol/(m²·s)	Boundary flux	Boundaries 32–35, 48–50, 52	Meta
tds2.bndFlux_cO	-dflux_spatial(cO)/tds2.d	mol/(m²·s)	Boundary flux	Boundaries 1–6, 8–9, 11, 14, 16–17, 20–21, 24–25, 28–31, 36–38, 40, 42, 44, 46–47, 51, 54–55, 57, 59, 61–63	Meta
tds2.DF_cRxx	DR	m²/s	Fluid diffusion coefficient, xx-	Domains 1–6	

Name	Expression	Unit	Description	Selection	Details
			component		
tds2.DF_cRyx	0	m²/s	Fluid diffusion coefficient, yx-component	Domains 1–6	
tds2.DF_cRzx	0	m²/s	Fluid diffusion coefficient, zx-component	Domains 1–6	
tds2.DF_cRxy	0	m²/s	Fluid diffusion coefficient, xy-component	Domains 1–6	
tds2.DF_cRyy	DR	m²/s	Fluid diffusion coefficient, yy-component	Domains 1–6	
tds2.DF_cRzy	0	m²/s	Fluid diffusion coefficient, zy-component	Domains 1–6	
tds2.DF_cRxz	0	m²/s	Fluid diffusion coefficient, xz-component	Domains 1–6	
tds2.DF_cRyz	0	m²/s	Fluid diffusion coefficient, yz-component	Domains 1–6	
tds2.DF_cRzz	DR	m²/s	Fluid diffusion coefficient, zz-component	Domains 1–6	
tds2.D_cRxx	tds2.DF_cRxx+tds2.DiT_cR	m²/s	Diffusion coefficient, xx-component	Domains 1–6	+ operation
tds2.D_cRyx	tds2.DF_cRyx	m²/s	Diffusion coefficient, yx-component	Domains 1–6	+ operation
tds2.D_cRzx	tds2.DF_cRzx	m²/s	Diffusion coefficient, zx-component	Domains 1–6	+ operation
tds2.D_cRxy	tds2.DF_cRxy	m²/s	Diffusion coefficient, xy-component	Domains 1–6	+ operation
tds2.D_cRyy	tds2.DF_cRyy+tds2.DiT_cR	m²/s	Diffusion coefficient, yy-component	Domains 1–6	+ operation
tds2.D_cRzy	tds2.DF_cRzy	m²/s	Diffusion coefficient, zy-component	Domains 1–6	+ operation

Name	Expression	Unit	Description	Selection	Details
tds2.D_cRxz	tds2.DF_cRxz	m²/s	Diffusion coefficient, xz-component	Domains 1–6	+ operation
tds2.D_cRyz	tds2.DF_cRyz	m²/s	Diffusion coefficient, yz-component	Domains 1–6	+ operation
tds2.D_cRzz	tds2.DF_cRzz+tds2.DiT_cR	m²/s	Diffusion coefficient, zz-component	Domains 1–6	+ operation
tds2.DF_cOxx	DO	m²/s	Fluid diffusion coefficient, xx-component	Domains 1–6	
tds2.DF_cOyx	0	m²/s	Fluid diffusion coefficient, yx-component	Domains 1–6	
tds2.DF_cOzx	0	m²/s	Fluid diffusion coefficient, zx-component	Domains 1–6	
tds2.DF_cOxy	0	m²/s	Fluid diffusion coefficient, xy-component	Domains 1–6	
tds2.DF_cOyy	DO	m²/s	Fluid diffusion coefficient, yy-component	Domains 1–6	
tds2.DF_cOzy	0	m²/s	Fluid diffusion coefficient, zy-component	Domains 1–6	
tds2.DF_cOxz	0	m²/s	Fluid diffusion coefficient, xz-component	Domains 1–6	
tds2.DF_cOyz	0	m²/s	Fluid diffusion coefficient, yz-component	Domains 1–6	
tds2.DF_cOzz	DO	m²/s	Fluid diffusion coefficient, zz-component	Domains 1–6	
tds2.D_cOxx	tds2.DF_cOxx+tds2.DiT_cO	m²/s	Diffusion coefficient, xx-component	Domains 1–6	+ operation
tds2.D_cOyx	tds2.DF_cOyx	m²/s	Diffusion coefficient, yx-component	Domains 1–6	+ operation

Name	Expression	Unit	Description	Selection	Details
tds2.D_cOzx	tds2.DF_cOzx	m²/s	Diffusion coefficient, zx-component	Domains 1–6	+ operation
tds2.D_cOxy	tds2.DF_cOxy	m²/s	Diffusion coefficient, xy-component	Domains 1–6	+ operation
tds2.D_cOyy	tds2.DF_cOyy+tds2.DiT_cO	m²/s	Diffusion coefficient, yy-component	Domains 1–6	+ operation
tds2.D_cOzy	tds2.DF_cOzy	m²/s	Diffusion coefficient, zy-component	Domains 1–6	+ operation
tds2.D_cOxz	tds2.DF_cOxz	m²/s	Diffusion coefficient, xz-component	Domains 1–6	+ operation
tds2.D_cOyz	tds2.DF_cOyz	m²/s	Diffusion coefficient, yz-component	Domains 1–6	+ operation
tds2.D_cOzz	tds2.DF_cOzz+tds2.DiT_cO	m²/s	Diffusion coefficient, zz-component	Domains 1–6	+ operation
tds2.Dav_cR	(tds2.D_cRxx+tds2.D_cRyy+tds2.D_cRzz)/3	m²/s	Average diffusion coefficient	Domains 1–6	
tds2.Dav_cO	(tds2.D_cOxx+tds2.D_cOyy+tds2.D_cOzz)/3	m²/s	Average diffusion coefficient	Domains 1–6	
tds2.tflux_cRx	tds2.dflux_cRx+tds2.cflux_cRx	mol/(m²·s)	Total flux, x-component	Domains 1–6	+ operation
tds2.tflux_cRy	tds2.dflux_cRy+tds2.cflux_cRy	mol/(m²·s)	Total flux, y-component	Domains 1–6	+ operation
tds2.tflux_cRz	tds2.dflux_cRz+tds2.cflux_cRz	mol/(m²·s)	Total flux, z-component	Domains 1–6	+ operation
tds2.dfluxMag_cR	sqrt(tds2.dflux_cRx^2+tds2.dflux_cRy^2+tds2.dflux_cRz^2)	mol/(m²·s)	Diffusive flux magnitude	Domains 1–6	
tds2.tfluxMag_cR	sqrt(tds2.tflux_cRx^2+tds2.tflux_cRy^2+tds2.tflux_cRz^2)	mol/(m²·s)	Total flux magnitude	Domains 1–6	
tds2.dpflux_cRx	0	mol/(m²·s)	Dispersive flux, x-	Domains 1–6	

Name	Expression	Unit	Description	Selection	Details
			component		
tds2.dpflux_cRy	0	mol/(m²·s)	Dispersive flux, y-component	Domains 1–6	
tds2.dpflux_cRz	0	mol/(m²·s)	Dispersive flux, z-component	Domains 1–6	
tds2.tflux_cOx	tds2.dflux_cOx+tds2.cflux_cOx	mol/(m²·s)	Total flux, x-component	Domains 1–6	+ operation
tds2.tflux_cOy	tds2.dflux_cOy+tds2.cflux_cOy	mol/(m²·s)	Total flux, y-component	Domains 1–6	+ operation
tds2.tflux_cOz	tds2.dflux_cOz+tds2.cflux_cOz	mol/(m²·s)	Total flux, z-component	Domains 1–6	+ operation
tds2.dfluxMag_cO	sqrt(tds2.dflux_cOx^2+tds2.dflux_cOy^2+tds2.dflux_cOz^2)	mol/(m²·s)	Diffusive flux magnitude	Domains 1–6	
tds2.tfluxMag_cO	sqrt(tds2.tflux_cOx^2+tds2.tflux_cOy^2+tds2.tflux_cOz^2)	mol/(m²·s)	Total flux magnitude	Domains 1–6	
tds2.dpflux_cOx	0	mol/(m²·s)	Dispersive flux, x-component	Domains 1–6	
tds2.dpflux_cOy	0	mol/(m²·s)	Dispersive flux, y-component	Domains 1–6	
tds2.dpflux_cOz	0	mol/(m²·s)	Dispersive flux, z-component	Domains 1–6	
tds2.dflux_cRx	-tds2.D_cRxx*cRx-tds2.D_cRxy*cRy-tds2.D_cRxz*cRz	mol/(m²·s)	Diffusive flux, x-component	Domains 1–6	+ operation
tds2.dflux_cRy	-tds2.D_cRyx*cRx-tds2.D_cRyy*cRy-tds2.D_cRyz*cRz	mol/(m²·s)	Diffusive flux, y-component	Domains 1–6	+ operation
tds2.dflux_cRz	-tds2.D_cRzx*cRx-tds2.D_cRzy*cRy-tds2.D_cRzz*cRz	mol/(m²·s)	Diffusive flux, z-component	Domains 1–6	+ operation
tds2.grad_cRx	cRx	mol/m⁴	Concentration gradient, x-component	Domains 1–6	
tds2.grad_cRy	cRy	mol/m⁴	Concentration gradient, y-component	Domains 1–6	
tds2.grad_cRz	cRz	mol/m⁴	Concentration gradient, z-	Domains 1–6	

Name	Expression	Unit	Description	Selection	Details
			component		
tds2.dflux_cOx	-tds2.D_cOxx*cOx-tds2.D_cOxy*cOy-tds2.D_cOxz*cOz	mol/(m²·s)	Diffusive flux, x-component	Domains 1–6	+ operation
tds2.dflux_cOy	-tds2.D_cOyx*cOx-tds2.D_cOyy*cOy-tds2.D_cOyz*cOz	mol/(m²·s)	Diffusive flux, y-component	Domains 1–6	+ operation
tds2.dflux_cOz	-tds2.D_cOzx*cOx-tds2.D_cOzy*cOy-tds2.D_cOzz*cOz	mol/(m²·s)	Diffusive flux, z-component	Domains 1–6	+ operation
tds2.grad_cOx	cOx	mol/m⁴	Concentration gradient, x-component	Domains 1–6	
tds2.grad_cOy	cOy	mol/m⁴	Concentration gradient, y-component	Domains 1–6	
tds2.grad_cOz	cOz	mol/m⁴	Concentration gradient, z-component	Domains 1–6	
tds2.cflux_cRx	cR*tds2.u	mol/(m²·s)	Convective flux, x-component	Domains 1–6	
tds2.cflux_cRy	cR*tds2.v	mol/(m²·s)	Convective flux, y-component	Domains 1–6	
tds2.cflux_cRz	cR*tds2.w	mol/(m²·s)	Convective flux, z-component	Domains 1–6	
tds2.cfluxMag_cR	sqrt(tds2.cflux_cRx^2+tds2.cflux_cRy^2+tds2.cflux_cRz^2)	mol/(m²·s)	Convective flux magnitude	Domains 1–6	
tds2.cflux_cOx	cO*tds2.u	mol/(m²·s)	Convective flux, x-component	Domains 1–6	
tds2.cflux_cOy	cO*tds2.v	mol/(m²·s)	Convective flux, y-component	Domains 1–6	
tds2.cflux_cOz	cO*tds2.w	mol/(m²·s)	Convective flux, z-component	Domains 1–6	
tds2.cfluxMag_cO	sqrt(tds2.cflux_cOx^2+tds2.cflux_cOy^2+tds2.cflux_cOz^2)	mol/(m²·s)	Convective flux magnitude	Domains 1–6	
tds2.Rlin_cR	0	1/s	Linear source term coefficient	Domains 1–6	+ operation
tds2.Res_cR	d(cR,t)+tds2.u*cRx	mol/(m³·s)	Equation residual	Domains 1–6	

Name	Expression	Unit	Description	Selection	Details
	+tds2.v*cRy+tds2.w*cRz-cR*tds2.Rlin_cR-tds2.R_cR				
tds2.Rlin_cO	0	1/s	Linear source term coefficient	Domains 1–6	+ operation
tds2.Res_cO	d(cO,t)+tds2.u*cOx+tds2.v*cOy+tds2.w*cOz-cO*tds2.Rlin_cO-tds2.R_cO	mol/(m³·s)	Equation residual	Domains 1–6	

Shape functions

Name	Shape function	Unit	Description	Shape frame	Selection
cR	Lagrange (Linear)	mol/m³	Concentration	Spatial	Domains 1–6
cO	Lagrange (Linear)	mol/m³	Concentration	Spatial	Domains 1–6

Weak Expressions

Weak expression	Integration order	Integration frame	Selection
(-cRt*test(cR)+tds2.dflux_cRx*test(cRx)+tds2.dflux_cRy*test(cRy)+tds2.dflux_cRz*test(cRz))*tds2.d	2	Spatial	Domains 1–6
(-cOt*test(cO)+tds2.dflux_cOx*test(cOx)+tds2.dflux_cOy*test(cOy)+tds2.dflux_cOz*test(cOz))*tds2.d	2	Spatial	Domains 1–6
-(tds2.u*cRx+tds2.v*cRy+tds2.w*cRz)*test(cR)*(isScalingSystemDomain==0)*tds2.d	2	Spatial	Domains 1–6
tds2.cbf_cR*test(cR)*tds2.d	2	Spatial	Boundaries 1–63
-(tds2.u*cOx+tds2.v*cOy+tds2.w*cOz)*test(cO)*(isScalingSystemDomain==0)*tds2.d	2	Spatial	Domains 1–6
tds2.cbf_cO*test(cO)*tds2.d	2	Spatial	Boundaries 1–63
tds2.streamline*(isScalingSystemDomain==0)*tds2.d	2	Spatial	Domains 1–6
tds2.crosswind*(isScalingSyste	4	Spatial	Domains 1–6

Weak expression	Integration order	Integration frame	Selection
mDomain==0)*tds2.d			

2.3.4 No Flux 1

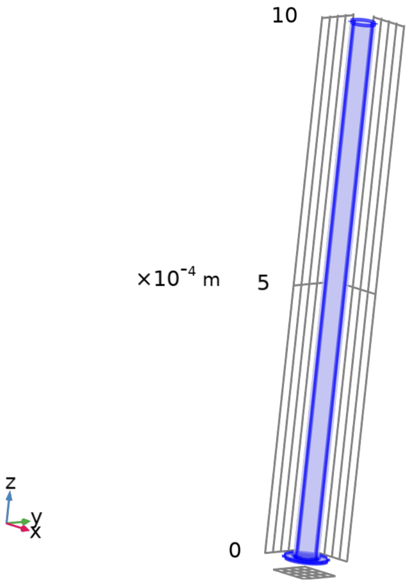

No Flux 1

SELECTION

Geometric entity level	Boundary
Selection	Geometry geom2: Dimension 2: All boundaries

EQUATIONS

$$-\mathbf{n}\cdot\mathbf{J}_i=0$$

Convection

SETTINGS

Description	Value
Include	Off

2.3.5 Initial Values 1

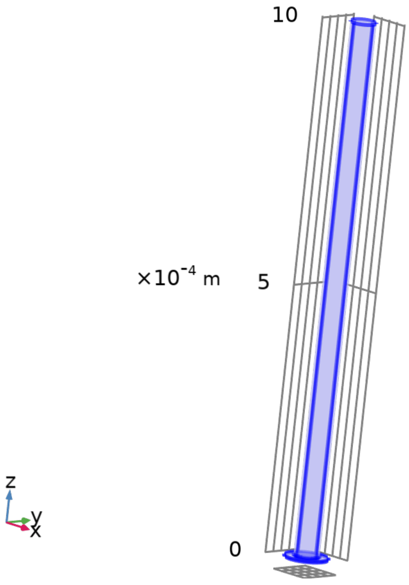

Initial Values 1

SELECTION

Geometric entity level	Domain
Selection	Geometry geom2: Dimension 3: All domains

Initial Values

SETTINGS

**Description**	**Value**	**Unit**
Concentration	{cRbulk, 0}	mol/m^3

Variables

**Name**	**Expression**	**Unit**	**Description**	**Selection**	**Details**
tds2.c0_cR	cRbulk	mol/m^3	Concentration	Domains 1–6	+ operation
tds2.c0_cO	0	mol/m^3	Concentration	Domains 1–6	+ operation

2.3.6 MEA Disk (Flux)

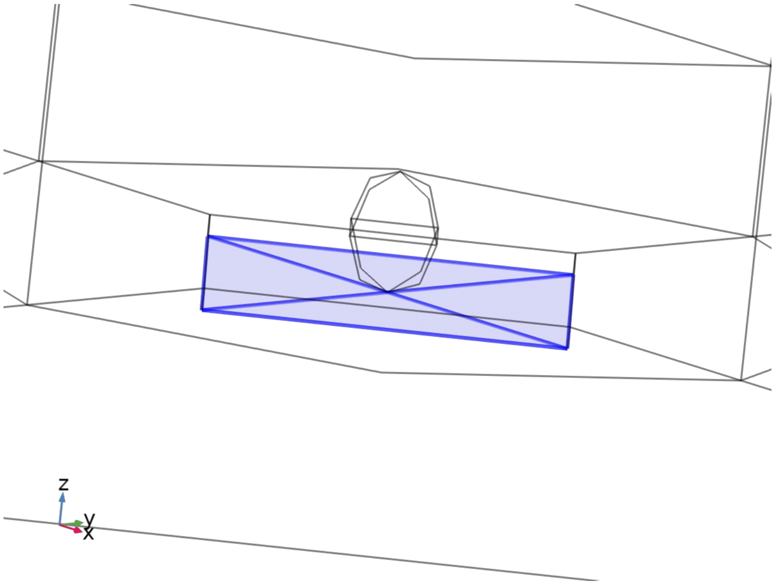

MEA Disk (Flux)

SELECTION

Geometric entity level	Boundary
Selection	Geometry geom2: Dimension 2: Boundaries 30–31, 47, 51

EQUATIONS

$$-\mathbf{n}\cdot\mathbf{J}_i = J_{0,i}$$

Convection

SETTINGS

Description	Value
Include	Off

Inward Flux

SETTINGS

Description	Value	Unit
Flux type	General inward flux	
Species cR	On	
Species cO	On	
	{k0*(exp(-a*f*(E-Ef))*cO - exp((1 - a)*f*(E-Ef))*cR), k0*(-exp(-a*f*(E-Ef))*cO + exp((1 - a)*f*(E-Ef))*cR)}	mol/(m²·s)

Variables

Name	Expression	Unit	Description	Selection

Name	Expression	Unit	Description	Selection
tds2.fl1.nmflow_cR	tds2.fl1.int(tds2.ntflux_cR)*tds2.d	mol/s	Normal molar flow rate	Global
tds2.fl1.nmflow_cO	tds2.fl1.int(tds2.ntflux_cO)*tds2.d	mol/s	Normal molar flow rate	Global

Weak Expressions

Weak expression	Integration order	Integration frame	Selection
k0*(exp(-a*f*(E-Ef))*cO-exp((1-a)*f*(E-Ef))*cR)*test(cR)*tds2.d	2	Spatial	Boundaries 30–31, 47, 51
k0*(-exp(-a*f*(E-Ef))*cO+exp((1-a)*f*(E-Ef))*cR)*test(cO)*tds2.d	2	Spatial	Boundaries 30–31, 47, 51

2.3.7 Pipette Back (Concentration)

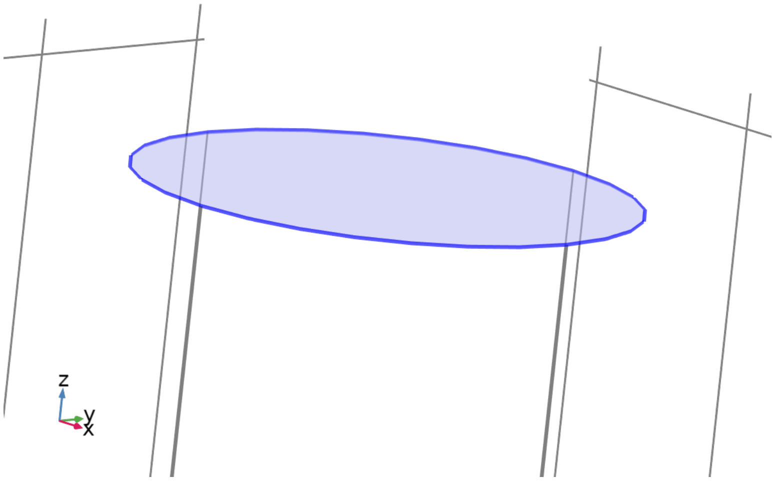

Pipette Back (Concentration)

SELECTION

Geometric entity level	Boundary
Selection	Geometry geom2: Dimension 2: Boundary 11

EQUATIONS

$$c_i = c_{0,i}$$

Concentration

SETTINGS

Description	Value	Unit

Description	Value	Unit
Species cR	On	
Species cO	On	
Concentration	{cRbulk, 0}	mol/m³

Variables

Name	Expression	Unit	Description	Selection	Details
tds2.c0_cR	cRbulk	mol/m³	Concentration	Boundary 11	+ operation
tds2.c0_cO	0	mol/m³	Concentration	Boundary 11	+ operation
tds2.conc1.nmflow_cR	tds2.conc1.int(tds2.ntflux_cR)*tds2.d	mol/s	Normal molar flow rate	Global	
tds2.conc1.nmflow_cO	tds2.conc1.int(tds2.ntflux_cO)*tds2.d	mol/s	Normal molar flow rate	Global	

Constraints

Constraint	Constraint force	Shape function	Selection	Details
-tds2.cVar_cR+tds2.c0_cR	test(-tds2.cVar_cR+tds2.c0_cR)	Lagrange (Linear)	Boundary 11	Elemental
-tds2.cVar_cO+tds2.c0_cO	test(-tds2.cVar_cO+tds2.c0_cO)	Lagrange (Linear)	Boundary 11	Elemental

2.3.8 Meniscus (Flux)

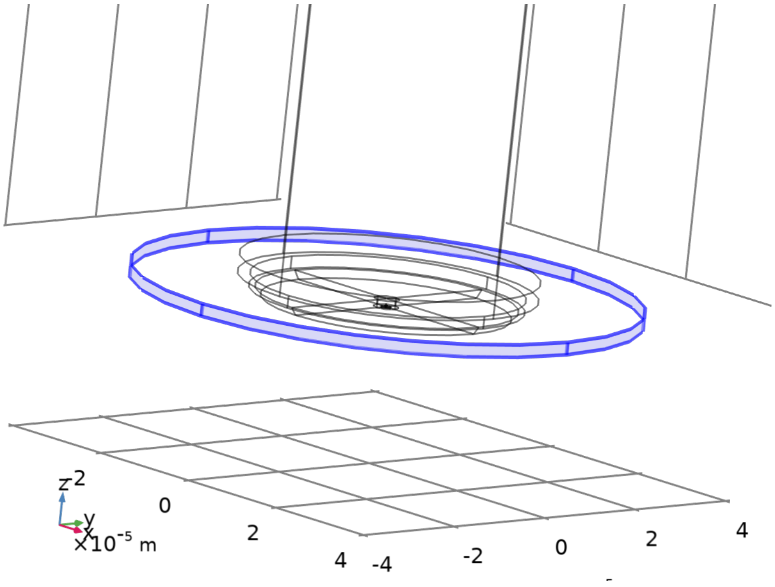

Meniscus (Flux)

SELECTION

Geometric entity level	Boundary
Selection	Geometry geom2: Dimension 2: Boundaries 1–2, 36, 63

EQUATIONS

$$-\mathbf{n}\cdot(\mathbf{J}_i+\mathbf{u}c_i)=J_{0,i}$$

Convection

SETTINGS

Description	Value
Include	On

Inward Flux

SETTINGS

Description	Value	Unit
Flux type	General inward flux	
Species cR	On	
Species cO	On	
	{0, 0}	mol/(m²·s)

Variables

Name	Expression	Unit	Description	Selection
tds2.cbf_cR	cR*(tds2.u*tds2.nxmesh+tds2.v*tds2.nymesh+tds2.w*tds2.nzmesh)	mol/(m²·s)	Convective boundary flux	Boundaries 1–2, 36, 63
tds2.cbf_cO	cO*(tds2.u*tds2.nxmesh+tds2.v*tds2.nymesh+tds2.w*tds2.nzmesh)	mol/(m²·s)	Convective boundary flux	Boundaries 1–2, 36, 63
tds2.fl2.nmflow_cR	tds2.fl2.int(tds2.ntflux_cR)*tds2.d	mol/s	Normal molar flow rate	Global
tds2.fl2.nmflow_cO	tds2.fl2.int(tds2.ntflux_cO)*tds2.d	mol/s	Normal molar flow rate	Global

Weak Expressions

Weak expression	Integration order	Integration frame	Selection
0	2	Spatial	Boundaries 1–2, 36, 63
0	2	Spatial	Boundaries 1–2, 36, 63

2.4 CREEPING FLOW

USED PRODUCTS

COMSOL Multiphysics
Electrochemistry Module

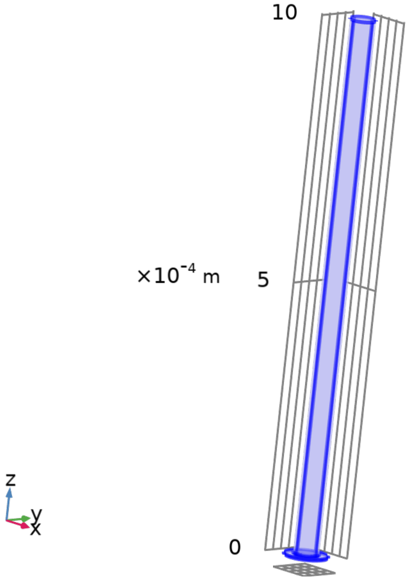

Creeping Flow

SELECTION

Geometric entity level	Domain
Selection	Geometry geom2: Dimension 3: Domains 1–6

EQUATIONS

$$\rho\frac{\partial \mathbf{u}}{\partial t} = \nabla\cdot[-p\mathbf{I} + \mathbf{K}] + \mathbf{F}$$
$$\rho\nabla\cdot\mathbf{u} = 0$$

2.4.1 Interface Settings

Discretization

SETTINGS

Description	Value
Discretization of fluids	P1 + P1

SETTINGS

Description	Value
Equation form	Study controlled

Physical Model

SETTINGS

Description	Value	Unit
Neglect inertial term (Stokes flow)	On	
Compressibility	Incompressible flow	
Enable porous media domains	Off	
Include gravity	Off	
Reference temperature	User defined	
Reference temperature	293.15	K
Reference pressure level	1.0133E5	Pa

Turbulence

SETTINGS

Description	Value
Turbulence model type	None

2.4.2 Variables

Name	Expression	Unit	Description	Selection	Details
spf.Tref	model.input.Tref	K	Reference temperature	Global	Meta
spf.dz	1	m	Thickness	Domains 1–6	
spf.pref	1[atm]	Pa	Reference pressure level	Domains 1–6	
spf.pA	p+spf.pref	Pa	Absolute pressure	Domains 1–6	
spf.hasWF	0		Help variable	Boundaries 1–6, 8–9, 11, 14, 16–17, 20–21, 24–25, 28–38, 40, 42, 44, 46–52, 54–55, 57, 59, 61–63	
spf.hasWF_u	0		Help variable	Boundaries 7, 10, 12–13, 15, 18–19, 22–23, 26–27, 39, 41, 43, 45, 53, 56, 58, 60	
spf.hasWF_d	0		Help variable	Boundaries 7, 10, 12–13, 15, 18–19, 22–23, 26–27, 39, 41, 43, 45, 53, 56, 58, 60	

Name	Expression	Unit	Description	Selection	Details
spf.dt_CFL	1/max(spf.maxop(sqrt(emetric_spatial(u-d(x,TIME),v-d(y,TIME),w-d(z,TIME)))),eps)	s	Time step, CFL=1	Global	
spf.CFL_number	timestep/spf.dt_CFL	1	CFL number	Global	
spf.Qvd_tot	spf.intop(spf.Qvd)	W	Total viscous dissipation	Global	
spf.K_stressx	spf.K_stress_tensorxx*spf.nxmesh+spf.K_stress_tensorxy*spf.nymesh+spf.K_stress_tensorxz*spf.nzmesh	N/m^2	Viscous force, exterior boundaries, x-component	Boundaries 1–6, 8–9, 11, 14, 16–17, 20–21, 24–25, 28–38, 40, 42, 44, 46–52, 54–55, 57, 59, 61–63	
spf.K_stressy	spf.K_stress_tensoryx*spf.nxmesh+spf.K_stress_tensoryy*spf.nymesh+spf.K_stress_tensoryz*spf.nzmesh	N/m^2	Viscous force, exterior boundaries, y-component	Boundaries 1–6, 8–9, 11, 14, 16–17, 20–21, 24–25, 28–38, 40, 42, 44, 46–52, 54–55, 57, 59, 61–63	
spf.K_stressz	spf.K_stress_tensorzx*spf.nxmesh+spf.K_stress_tensorzy*spf.nymesh+spf.K_stress_tensorzz*spf.nzmesh	N/m^2	Viscous force, exterior boundaries, z-component	Boundaries 1–6, 8–9, 11, 14, 16–17, 20–21, 24–25, 28–38, 40, 42, 44, 46–52, 54–55, 57, 59, 61–63	
spf.T_stressx	spf.T_stress_tensorxx*spf.nxmesh+spf.T_stress_tensorxy*spf.nymesh+spf.T_stress_tensorxz*spf.nzmesh	N/m^2	Total traction, exterior boundaries, x-component	Boundaries 1–6, 8–9, 11, 14, 16–17, 20–21, 24–25, 28–38, 40, 42, 44, 46–52, 54–55, 57, 59, 61–63	
spf.T_stressy	spf.T_stress_tensoryx*spf.nxmesh+spf.T_stress_tensoryy*spf.nymesh+spf.T_stress_tensoryz*spf.nzmesh	N/m^2	Total traction, exterior boundaries, y-component	Boundaries 1–6, 8–9, 11, 14, 16–17, 20–21, 24–25, 28–38, 40, 42, 44, 46–52, 54–55, 57, 59, 61–63	
spf.T_stressz	spf.T_stress_tensorzx*spf.nxmesh+spf.T_stres	N/m^2	Total traction, exterior	Boundaries 1–6, 8–9, 11, 14,	

Name	Expression	Unit	Description	Selection	Details
	s_tensorzy*spf.nymesh+spf.T_stress_tensorzz*spf.nzmesh		boundaries, z-component	16–17, 20–21, 24–25, 28–38, 40, 42, 44, 46–52, 54–55, 57, 59, 61–63	
spf.K_stress_dx	down(spf.K_stress_tensorxx)*spf.nxmesh+down(spf.K_stress_tensorxy)*spf.nymesh+down(spf.K_stress_tensorxz)*spf.nzmesh	N/m²	Viscous force, interior boundaries, downside, x-component	Boundaries 7, 10, 12–13, 15, 18–19, 22–23, 26–27, 39, 41, 43, 45, 53, 56, 58, 60	
spf.K_stress_dy	down(spf.K_stress_tensoryx)*spf.nxmesh+down(spf.K_stress_tensoryy)*spf.nymesh+down(spf.K_stress_tensoryz)*spf.nzmesh	N/m²	Viscous force, interior boundaries, downside, y-component	Boundaries 7, 10, 12–13, 15, 18–19, 22–23, 26–27, 39, 41, 43, 45, 53, 56, 58, 60	
spf.K_stress_dz	down(spf.K_stress_tensorzx)*spf.nxmesh+down(spf.K_stress_tensorzy)*spf.nymesh+down(spf.K_stress_tensorzz)*spf.nzmesh	N/m²	Viscous force, interior boundaries, downside, z-component	Boundaries 7, 10, 12–13, 15, 18–19, 22–23, 26–27, 39, 41, 43, 45, 53, 56, 58, 60	
spf.K_stress_dx	down(spf.K_stress_tensorxx)*spf.dnxmesh+down(spf.K_stress_tensorxy)*spf.dnymesh+down(spf.K_stress_tensorxz)*spf.dnzmesh	N/m²	Viscous force, interior boundaries, downside, x-component	Boundaries 1–6, 8–9, 11, 14, 16–17, 20–21, 24–25, 28–31, 36–38, 40, 42, 44, 46–47, 51, 54–55, 57, 59, 61–63	
spf.K_stress_dy	down(spf.K_stress_tensoryx)*spf.dnxmesh+down(spf.K_stress_tensoryy)*spf.dnymesh+down(spf.K_stress_tensoryz)*spf.dnzmesh	N/m²	Viscous force, interior boundaries, downside, y-component	Boundaries 1–6, 8–9, 11, 14, 16–17, 20–21, 24–25, 28–31, 36–38, 40, 42, 44, 46–47, 51, 54–55, 57, 59, 61–63	
spf.K_stress_dz	down(spf.K_stress_tensorzx)*spf.dnxmesh+down(spf.K_stress_tensorzy)*spf.dnymesh+down(spf.K_stress_tensorzz)*spf.dnzmesh	N/m²	Viscous force, interior boundaries, downside, z-component	Boundaries 1–6, 8–9, 11, 14, 16–17, 20–21, 24–25, 28–31, 36–38, 40, 42, 44, 46–47, 51,	

Name	Expression	Unit	Description	Selection	Details
				54–55, 57, 59, 61–63	
spf.K_stress_ux	-up(spf.K_stress_tensorxx)*spf.nxmesh-up(spf.K_stress_tensorxy)*spf.nymesh-up(spf.K_stress_tensorxz)*spf.nzmesh	N/m²	Viscous force, interior boundaries, upside, x-component	Boundaries 7, 10, 12–13, 15, 18–19, 22–23, 26–27, 39, 41, 43, 45, 53, 56, 58, 60	
spf.K_stress_uy	-up(spf.K_stress_tensoryx)*spf.nxmesh-up(spf.K_stress_tensoryy)*spf.nymesh-up(spf.K_stress_tensoryz)*spf.nzmesh	N/m²	Viscous force, interior boundaries, upside, y-component	Boundaries 7, 10, 12–13, 15, 18–19, 22–23, 26–27, 39, 41, 43, 45, 53, 56, 58, 60	
spf.K_stress_uz	-up(spf.K_stress_tensorzx)*spf.nxmesh-up(spf.K_stress_tensorzy)*spf.nymesh-up(spf.K_stress_tensorzz)*spf.nzmesh	N/m²	Viscous force, interior boundaries, upside, z-component	Boundaries 7, 10, 12–13, 15, 18–19, 22–23, 26–27, 39, 41, 43, 45, 53, 56, 58, 60	
spf.K_stress_ux	up(spf.K_stress_tensorxx)*spf.unxmesh+up(spf.K_stress_tensorxy)*spf.unymesh+up(spf.K_stress_tensorxz)*spf.unzmesh	N/m²	Viscous force, interior boundaries, upside, x-component	Boundaries 32–35, 48–50, 52	
spf.K_stress_uy	up(spf.K_stress_tensoryx)*spf.unxmesh+up(spf.K_stress_tensoryy)*spf.unymesh+up(spf.K_stress_tensoryz)*spf.unzmesh	N/m²	Viscous force, interior boundaries, upside, y-component	Boundaries 32–35, 48–50, 52	
spf.K_stress_uz	up(spf.K_stress_tensorzx)*spf.unxmesh+up(spf.K_stress_tensorzy)*spf.unymesh+up(spf.K_stress_tensorzz)*spf.unzmesh	N/m²	Viscous force, interior boundaries, upside, z-component	Boundaries 32–35, 48–50, 52	
spf.T_stress_dx	down(spf.T_stress_tensorxx)*spf.nxmesh+down(spf.T_stress_tensorxy)*spf.nymesh+down	N/m²	Total traction, interior boundaries, downside, x-	Boundaries 7, 10, 12–13, 15, 18–19, 22–23, 26–27, 39, 41,	

Name	Expression	Unit	Description	Selection	Details
	(spf.T_stress_tensorxz)*spf.nzmesh		component	43, 45, 53, 56, 58, 60	
spf.T_stress_dy	down(spf.T_stress_tensoryx)*spf.nxmesh+down(spf.T_stress_tensoryy)*spf.nymesh+down(spf.T_stress_tensoryz)*spf.nzmesh	N/m²	Total traction, interior boundaries, downside, y-component	Boundaries 7, 10, 12–13, 15, 18–19, 22–23, 26–27, 39, 41, 43, 45, 53, 56, 58, 60	
spf.T_stress_dz	down(spf.T_stress_tensorzx)*spf.nxmesh+down(spf.T_stress_tensorzy)*spf.nymesh+down(spf.T_stress_tensorzz)*spf.nzmesh	N/m²	Total traction, interior boundaries, downside, z-component	Boundaries 7, 10, 12–13, 15, 18–19, 22–23, 26–27, 39, 41, 43, 45, 53, 56, 58, 60	
spf.T_stress_dx	down(spf.T_stress_tensorxx)*spf.dnxmesh+down(spf.T_stress_tensorxy)*spf.dnymesh+down(spf.T_stress_tensorxz)*spf.dnzmesh	N/m²	Total traction, interior boundaries, downside, x-component	Boundaries 1–6, 8–9, 11, 14, 16–17, 20–21, 24–25, 28–31, 36–38, 40, 42, 44, 46–47, 51, 54–55, 57, 59, 61–63	
spf.T_stress_dy	down(spf.T_stress_tensoryx)*spf.dnxmesh+down(spf.T_stress_tensoryy)*spf.dnymesh+down(spf.T_stress_tensoryz)*spf.dnzmesh	N/m²	Total traction, interior boundaries, downside, y-component	Boundaries 1–6, 8–9, 11, 14, 16–17, 20–21, 24–25, 28–31, 36–38, 40, 42, 44, 46–47, 51, 54–55, 57, 59, 61–63	
spf.T_stress_dz	down(spf.T_stress_tensorzx)*spf.dnxmesh+down(spf.T_stress_tensorzy)*spf.dnymesh+down(spf.T_stress_tensorzz)*spf.dnzmesh	N/m²	Total traction, interior boundaries, downside, z-component	Boundaries 1–6, 8–9, 11, 14, 16–17, 20–21, 24–25, 28–31, 36–38, 40, 42, 44, 46–47, 51, 54–55, 57, 59, 61–63	
spf.T_stress_ux	-up(spf.T_stress_tensorxx)*spf.nxmesh-up(spf.T_stress_tensorxy)*spf.nymesh-up(spf.T_stress_tensorxz)*spf.nzmesh	N/m²	Total traction, interior boundaries, upside, x-component	Boundaries 7, 10, 12–13, 15, 18–19, 22–23, 26–27, 39, 41, 43, 45, 53, 56, 58, 60	

Name	Expression	Unit	Description	Selection	Details
spf.T_stress_uy	-up(spf.T_stress_tensoryx)*spf.nxmesh-up(spf.T_stress_tensoryy)*spf.nymesh-up(spf.T_stress_tensoryz)*spf.nzmesh	N/m²	Total traction, interior boundaries, upside, y-component	Boundaries 7, 10, 12–13, 15, 18–19, 22–23, 26–27, 39, 41, 43, 45, 53, 56, 58, 60	
spf.T_stress_uz	-up(spf.T_stress_tensorzx)*spf.nxmesh-up(spf.T_stress_tensorzy)*spf.nymesh-up(spf.T_stress_tensorzz)*spf.nzmesh	N/m²	Total traction, interior boundaries, upside, z-component	Boundaries 7, 10, 12–13, 15, 18–19, 22–23, 26–27, 39, 41, 43, 45, 53, 56, 58, 60	
spf.T_stress_ux	up(spf.T_stress_tensorxx)*spf.unxmesh+up(spf.T_stress_tensorxy)*spf.unymesh+up(spf.T_stress_tensorxz)*spf.unzmesh	N/m²	Total traction, interior boundaries, upside, x-component	Boundaries 32–35, 48–50, 52	
spf.T_stress_uy	up(spf.T_stress_tensoryx)*spf.unxmesh+up(spf.T_stress_tensoryy)*spf.unymesh+up(spf.T_stress_tensoryz)*spf.unzmesh	N/m²	Total traction, interior boundaries, upside, y-component	Boundaries 32–35, 48–50, 52	
spf.T_stress_uz	up(spf.T_stress_tensorzx)*spf.unxmesh+up(spf.T_stress_tensorzy)*spf.unymesh+up(spf.T_stress_tensorzz)*spf.unzmesh	N/m²	Total traction, interior boundaries, upside, z-component	Boundaries 32–35, 48–50, 52	
spf.usePseudoTimeStepping	isrunningpseudotimestepping	1	Help variable	Global	
spf.localCFLvalue	1.3^min(niterCMP,9)+if(niterCMP>=25,9*1.3^min(-25+niterCMP,9),0)+if(niterCMP>=45,90*1.3^min(-45+niterCMP,9),0)		Local CFL number	Domains 1–6	
spf.locCFL	max(CFLCMP,sqrt(eps))	1	Local CFL number	Global	
spf.geometryLengthScale	2.0499999999999997E-5	m	Geometry length scale	Domains 1–6	

Name	Expression	Unit	Description	Selection	Details
spf.time_step_inv	max(sqrt(emetric_spatial(u,v,w)*2^gmg_level^2),spf.nu/spf.geometryLengthScale^2)	Hz	Inverse time step	Domains 1–6	
spf.tsti	nojac(spf.time_step_inv/spf.locCFL)	1/s	Help variable	Domains 1–6	
spf.nx	nx	1	Normal vector, x-component	Boundaries 7, 10, 12–13, 15, 18–19, 22–23, 26–27, 39, 41, 43, 45, 53, 56, 58, 60	
spf.ny	ny	1	Normal vector, y-component	Boundaries 7, 10, 12–13, 15, 18–19, 22–23, 26–27, 39, 41, 43, 45, 53, 56, 58, 60	
spf.nz	nz	1	Normal vector, z-component	Boundaries 7, 10, 12–13, 15, 18–19, 22–23, 26–27, 39, 41, 43, 45, 53, 56, 58, 60	
spf.nx	unx	1	Normal vector, x-component	Boundaries 32–35, 48–50, 52	
spf.ny	uny	1	Normal vector, y-component	Boundaries 32–35, 48–50, 52	
spf.nz	unz	1	Normal vector, z-component	Boundaries 32–35, 48–50, 52	
spf.nx	dnx	1	Normal vector, x-component	Boundaries 1–6, 8–9, 11, 14, 16–17, 20–21, 24–25, 28–31, 36–38, 40, 42, 44, 46–47, 51, 54–55, 57, 59, 61–63	
spf.ny	dny	1	Normal vector, y-component	Boundaries 1–6, 8–9, 11, 14, 16–17, 20–21,	

Name	Expression	Unit	Description	Selection	Details
				24–25, 28–31, 36–38, 40, 42, 44, 46–47, 51, 54–55, 57, 59, 61–63	
spf.nz	dnz	1	Normal vector, z-component	Boundaries 1–6, 8–9, 11, 14, 16–17, 20–21, 24–25, 28–31, 36–38, 40, 42, 44, 46–47, 51, 54–55, 57, 59, 61–63	
spf.nxmesh	nxmesh	1	Normal vector, x-component	Boundaries 7, 10, 12–13, 15, 18–19, 22–23, 26–27, 39, 41, 43, 45, 53, 56, 58, 60	
spf.nymesh	nymesh	1	Normal vector, y-component	Boundaries 7, 10, 12–13, 15, 18–19, 22–23, 26–27, 39, 41, 43, 45, 53, 56, 58, 60	
spf.nzmesh	nzmesh	1	Normal vector, z-component	Boundaries 7, 10, 12–13, 15, 18–19, 22–23, 26–27, 39, 41, 43, 45, 53, 56, 58, 60	
spf.nxmesh	unxmesh	1	Normal vector, x-component	Boundaries 32–35, 48–50, 52	
spf.nymesh	unymesh	1	Normal vector, y-component	Boundaries 32–35, 48–50, 52	
spf.nzmesh	unzmesh	1	Normal vector, z-component	Boundaries 32–35, 48–50, 52	
spf.nxmesh	dnxmesh	1	Normal vector, x-component	Boundaries 1–6, 8–9, 11, 14, 16–17, 20–21, 24–25, 28–31, 36–38, 40, 42,	

Name	Expression	Unit	Description	Selection	Details
				44, 46–47, 51, 54–55, 57, 59, 61–63	
spf.nymesh	dnymesh	1	Normal vector, y-component	Boundaries 1–6, 8–9, 11, 14, 16–17, 20–21, 24–25, 28–31, 36–38, 40, 42, 44, 46–47, 51, 54–55, 57, 59, 61–63	
spf.nzmesh	dnzmesh	1	Normal vector, z-component	Boundaries 1–6, 8–9, 11, 14, 16–17, 20–21, 24–25, 28–31, 36–38, 40, 42, 44, 46–47, 51, 54–55, 57, 59, 61–63	

2.4.3 Fluid Properties 1

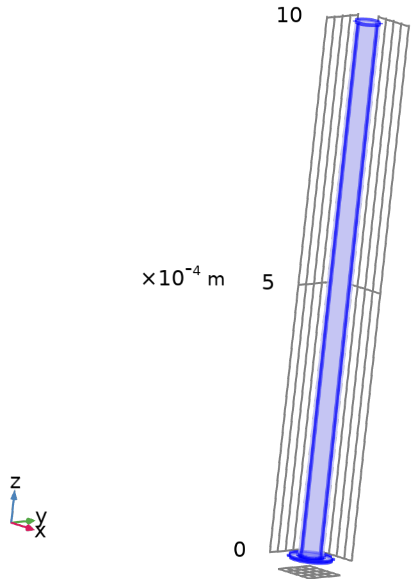

Fluid Properties 1

SELECTION

Geometric entity level	Domain
Selection	Geometry geom2: Dimension 3: All domains

EQUATIONS

$$\rho\frac{\partial \mathbf{u}}{\partial t} = \nabla \cdot [-p\mathbf{I} + \mathbf{K}] + \mathbf{F}$$

$$\rho\nabla \cdot \mathbf{u} = 0$$

$$\mathbf{K} = \mu\left(\nabla\mathbf{u} + (\nabla\mathbf{u})^T\right)$$

Fluid Properties

SETTINGS

Description	Value	Unit
Density	User defined	
Density	997	kg/m³
	Specify dynamic viscosity	
Dynamic viscosity	User defined	
Dynamic viscosity	8.9E-4	Pa·s

Variables

Name	Expression	Unit	Description	Selection	Details
spf.mu	material.mu	Pa·s	Dynamic viscosity	Domains 1–6	Meta
spf.rho	material.rho	kg/m³	Density	Domains 1–6	Meta
spf.Trho	spf.fp1.minput_temperature	K	Temperature for density evaluation	Domains 1–6	
spf.prho	spf.fp1.minput_pressure	Pa	Pressure for the evaluation of density	Domains 1–6	
spf.rhoref	subst(material.rho,minput.T,spf.Tref,minput.pA,spf.pref)	kg/m³	Reference density	Domains 1–6	Meta
spf.mumat	material.mu	Pa·s	Dynamic viscosity	Domains 1–6	Meta
spf.srijxx	ux	1/s	Strain rate tensor, xx-component	Domains 1–6	
spf.srijyx	0.5*(vx+uy)	1/s	Strain rate tensor, yx-component	Domains 1–6	
spf.srijzx	0.5*(wx+uz)	1/s	Strain rate tensor, zx-component	Domains 1–6	
spf.srijxy	0.5*(uy+vx)	1/s	Strain rate tensor, xy-	Domains 1–6	

Name	Expression	Unit	Description	Selection	Details
			component		
spf.srijyy	vy	1/s	Strain rate tensor, yy-component	Domains 1–6	
spf.srijzy	0.5*(wy+vz)	1/s	Strain rate tensor, zy-component	Domains 1–6	
spf.srijxz	0.5*(uz+wx)	1/s	Strain rate tensor, xz-component	Domains 1–6	
spf.srijyz	0.5*(vz+wy)	1/s	Strain rate tensor, yz-component	Domains 1–6	
spf.srijzz	wz	1/s	Strain rate tensor, zz-component	Domains 1–6	
spf.rrijxx	0	1/s	Rotation rate tensor, xx-component	Domains 1–6	
spf.rrijyx	0.5*(vx-uy)	1/s	Rotation rate tensor, yx-component	Domains 1–6	
spf.rrijzx	0.5*(wx-uz)	1/s	Rotation rate tensor, zx-component	Domains 1–6	
spf.rrijxy	0.5*(uy-vx)	1/s	Rotation rate tensor, xy-component	Domains 1–6	
spf.rrijyy	0	1/s	Rotation rate tensor, yy-component	Domains 1–6	
spf.rrijzy	0.5*(wy-vz)	1/s	Rotation rate tensor, zy-component	Domains 1–6	
spf.rrijxz	0.5*(uz-wx)	1/s	Rotation rate tensor, xz-component	Domains 1–6	
spf.rrijyz	0.5*(vz-wy)	1/s	Rotation rate tensor, yz-component	Domains 1–6	
spf.rrijzz	0	1/s	Rotation rate tensor, zz-component	Domains 1–6	

Name	Expression	Unit	Description	Selection	Details
spf.sr	sqrt(2*spf.srijxx^2+2*spf.srijxy^2+2*spf.srijxz^2+2*spf.srijyx^2+2*spf.srijyy^2+2*spf.srijyz^2+2*spf.srijzx^2+2*spf.srijzy^2+2*spf.srijzz^2+eps)	1/s	Shear rate	Domains 1–6	
spf.rr	sqrt(2*spf.rrijxx^2+2*spf.rrijxy^2+2*spf.rrijxz^2+2*spf.rrijyx^2+2*spf.rrijyy^2+2*spf.rrijyz^2+2*spf.rrijzx^2+2*spf.rrijzy^2+2*spf.rrijzz^2+eps)	1/s	Rotation rate	Domains 1–6	
spf.divu	ux+vy+wz	1/s	Divergence of velocity field	Domains 1–6	
spf.Fx	0	N/m^3	Volume force, x-component	Domains 1–6	+ operation
spf.Fy	0	N/m^3	Volume force, y-component	Domains 1–6	+ operation
spf.Fz	0	N/m^3	Volume force, z-component	Domains 1–6	+ operation
spf.U	sqrt(u^2+v^2+w^2)	m/s	Velocity magnitude	Domains 1–6	
spf.vorticityx	wy-vz	1/s	Vorticity field, x-component	Domains 1–6	
spf.vorticityy	-wx+uz	1/s	Vorticity field, y-component	Domains 1–6	
spf.vorticityz	vx-uy	1/s	Vorticity field, z-component	Domains 1–6	
spf.vort_magn	sqrt(spf.vorticityx^2+spf.vorticityy^2+spf.vorticityz^2)	1/s	Vorticity magnitude	Domains 1–6	
spf.cellRe	0.25*spf.rho*sqrt(emetric_spatial(u-d(x,TIME),v-d(y,TIME),w-d(z,TIME))/emetric2_spatial)/spf.m	1	Cell Reynolds number	Domains 1–6	

Name	Expression	Unit	Description	Selection	Details
	u				
spf.nu	spf.mu/spf.rho	m^2/s	Kinematic viscosity	Domains 1–6	
spf.betaT	0	1/Pa	Isothermal compressibility coefficient	Domains 1–6	
spf.Qm	0	$kg/(m^3 \cdot s)$	Source term	Domains 1–6	+ operation
spf.Fgtotx	0	N/m^3	Gravity force, x-component	Domains 1–6	+ operation
spf.Fgtoty	0	N/m^3	Gravity force, y-component	Domains 1–6	+ operation
spf.Fgtotz	0	N/m^3	Gravity force, z-component	Domains 1–6	+ operation
spf.Qm_aco	0	$kg/(m^3 \cdot s)$	Acoustic mass source	Domains 1–6	
spf.F_acox	0	N/m^3	Acoustic volume force, x-component	Domains 1–6	
spf.F_acoy	0	N/m^3	Acoustic volume force, y-component	Domains 1–6	
spf.F_acoz	0	N/m^3	Acoustic volume force, z-component	Domains 1–6	
spf.gamma_sr	sqrt(2*spf.srijxx^2+2*spf.srijxy^2+2*spf.srijxz^2+2*spf.srijyx^2+2*spf.srijyy^2+2*spf.srijyz^2+2*spf.srijzx^2+2*spf.srijzy^2+2*spf.srijzz^2+eps)	1/s	Shear rate	Domains 1–6	
spf.mu_eff	spf.mu+spf.muT	Pa·s	Effective dynamic viscosity	Domains 1–6	
spf.muT	0	Pa·s	Turbulent dynamic viscosity	Domains 1–6	+ operation
spf.T_stress_tensorxx	spf.K_stress_tensorxx-p	N/m^2	Total stress tensor, xx-component	Domains 1–6	+ operation

Name	Expression	Unit	Description	Selection	Details
spf.T_stress_tensoryx	spf.K_stress_tensoryx	N/m²	Total stress tensor, yx-component	Domains 1–6	+ operation
spf.T_stress_tensorzx	spf.K_stress_tensorzx	N/m²	Total stress tensor, zx-component	Domains 1–6	+ operation
spf.T_stress_tensorxy	spf.K_stress_tensorxy	N/m²	Total stress tensor, xy-component	Domains 1–6	+ operation
spf.T_stress_tensoryy	spf.K_stress_tensoryy-p	N/m²	Total stress tensor, yy-component	Domains 1–6	+ operation
spf.T_stress_tensorzy	spf.K_stress_tensorzy	N/m²	Total stress tensor, zy-component	Domains 1–6	+ operation
spf.T_stress_tensorxz	spf.K_stress_tensorxz	N/m²	Total stress tensor, xz-component	Domains 1–6	+ operation
spf.T_stress_tensoryz	spf.K_stress_tensoryz	N/m²	Total stress tensor, yz-component	Domains 1–6	+ operation
spf.T_stress_tensorzz	spf.K_stress_tensorzz-p	N/m²	Total stress tensor, zz-component	Domains 1–6	+ operation
spf.K_stress_tensorxx	2*spf.mu_eff*ux	N/m²	Viscous stress tensor, xx-component	Domains 1–6	+ operation
spf.K_stress_tensoryx	spf.mu_eff*(vx+uy)	N/m²	Viscous stress tensor, yx-component	Domains 1–6	+ operation
spf.K_stress_tensorzx	spf.mu_eff*(wx+uz)	N/m²	Viscous stress tensor, zx-component	Domains 1–6	+ operation
spf.K_stress_tensorxy	spf.mu_eff*(uy+vx)	N/m²	Viscous stress tensor, xy-component	Domains 1–6	+ operation
spf.K_stress_tensoryy	2*spf.mu_eff*vy	N/m²	Viscous stress tensor, yy-component	Domains 1–6	+ operation
spf.K_stress_tensorzy	spf.mu_eff*(wy+vz)	N/m²	Viscous stress tensor, zy-component	Domains 1–6	+ operation

Name	Expression	Unit	Description	Selection	Details
spf.K_stress_tensorxz	spf.mu_eff*(uz+wx)	N/m²	Viscous stress tensor, xz-component	Domains 1–6	+ operation
spf.K_stress_tensoryz	spf.mu_eff*(vz+wy)	N/m²	Viscous stress tensor, yz-component	Domains 1–6	+ operation
spf.K_stress_tensorzz	2*spf.mu_eff*wz	N/m²	Viscous stress tensor, zz-component	Domains 1–6	+ operation
spf.K_stress_tensor_testxx	2*spf.mu_eff*test(ux)	N/m²	Viscous stress tensor test, xx-component	Domains 1–6	+ operation
spf.K_stress_tensor_testyx	spf.mu_eff*(test(vx)+test(uy))	N/m²	Viscous stress tensor test, yx-component	Domains 1–6	+ operation
spf.K_stress_tensor_testzx	spf.mu_eff*(test(wx)+test(uz))	N/m²	Viscous stress tensor test, zx-component	Domains 1–6	+ operation
spf.K_stress_tensor_testxy	spf.mu_eff*(test(uy)+test(vx))	N/m²	Viscous stress tensor test, xy-component	Domains 1–6	+ operation
spf.K_stress_tensor_testyy	2*spf.mu_eff*test(vy)	N/m²	Viscous stress tensor test, yy-component	Domains 1–6	+ operation
spf.K_stress_tensor_testzy	spf.mu_eff*(test(wy)+test(vz))	N/m²	Viscous stress tensor test, zy-component	Domains 1–6	+ operation
spf.K_stress_tensor_testxz	spf.mu_eff*(test(uz)+test(wx))	N/m²	Viscous stress tensor test, xz-component	Domains 1–6	+ operation
spf.K_stress_tensor_testyz	spf.mu_eff*(test(vz)+test(wy))	N/m²	Viscous stress tensor test, yz-component	Domains 1–6	+ operation
spf.K_stress_tensor_testzz	2*spf.mu_eff*test(wz)	N/m²	Viscous stress tensor test, zz-component	Domains 1–6	+ operation
spf.upwind_helpx	-d(x,TIME)	m/s	Upwind term, x-component	Domains 1–6	+ operation
spf.upwind_helpy	-d(y,TIME)	m/s	Upwind term, y-component	Domains 1–6	+ operation
spf.upwind_helpz	-d(z,TIME)	m/s	Upwind term, z-component	Domains 1–6	+ operation

Name	Expression	Unit	Description	Selection	Details
spf.continuityEquation	spf.rho*spf.divu	kg/(m³·s)	Continuity equation	Domains 1–6	
spf.contCoeff	spf.rho	kg/m³	Help variable	Domains 1–6	
spf.tau_vdxx	2*spf.mu*spf.srijxx	Pa	Viscous stress tensor, xx-component	Domains 1–6	+ operation
spf.tau_vdyx	2*spf.mu*spf.srijyx	Pa	Viscous stress tensor, yx-component	Domains 1–6	+ operation
spf.tau_vdzx	2*spf.mu*spf.srijzx	Pa	Viscous stress tensor, zx-component	Domains 1–6	+ operation
spf.tau_vdxy	2*spf.mu*spf.srijxy	Pa	Viscous stress tensor, xy-component	Domains 1–6	+ operation
spf.tau_vdyy	2*spf.mu*spf.srijyy	Pa	Viscous stress tensor, yy-component	Domains 1–6	+ operation
spf.tau_vdzy	2*spf.mu*spf.srijzy	Pa	Viscous stress tensor, zy-component	Domains 1–6	+ operation
spf.tau_vdxz	2*spf.mu*spf.srijxz	Pa	Viscous stress tensor, xz-component	Domains 1–6	+ operation
spf.tau_vdyz	2*spf.mu*spf.srijyz	Pa	Viscous stress tensor, yz-component	Domains 1–6	+ operation
spf.tau_vdzz	2*spf.mu*spf.srijzz	Pa	Viscous stress tensor, zz-component	Domains 1–6	+ operation
spf.Qvd	spf.tau_vdxx*ux+spf.tau_vdxy*uy+spf.tau_vdxz*uz+spf.tau_vdyx*vx+spf.tau_vdyy*vy+spf.tau_vdyz*vz+spf.tau_vdzx*wx+spf.tau_vdzy*wy+spf.tau_vdzz*wz	W/m³	Viscous dissipation	Domains 1–6	+ operation
spf.epsilon_p	1	1	Porosity	Domains 1–6	
spf.epsilon_p_pos	1	1	Positive porosity	Domains 1–6	
spf.Fst_tensorxx	0	N/m²	Surface tension force, xx-	Domains 1–6	+ operation

Name	Expression	Unit	Description	Selection	Details
			component		
spf.Fst_tensoryx	0	N/m²	Surface tension force, yx-component	Domains 1–6	+ operation
spf.Fst_tensorzx	0	N/m²	Surface tension force, zx-component	Domains 1–6	+ operation
spf.Fst_tensorxy	0	N/m²	Surface tension force, xy-component	Domains 1–6	+ operation
spf.Fst_tensoryy	0	N/m²	Surface tension force, yy-component	Domains 1–6	+ operation
spf.Fst_tensorzy	0	N/m²	Surface tension force, zy-component	Domains 1–6	+ operation
spf.Fst_tensorxz	0	N/m²	Surface tension force, xz-component	Domains 1–6	+ operation
spf.Fst_tensoryz	0	N/m²	Surface tension force, yz-component	Domains 1–6	+ operation
spf.Fst_tensorzz	0	N/m²	Surface tension force, zz-component	Domains 1–6	+ operation
spf.res_u	spf.rho*ut+px-(d(2*ux,x)+d(uy+vx,y)+d(uz+wx,z))*spf.mu-spf.Fx	N/m³	Equation residual	Domains 1–6	
spf.res_v	spf.rho*vt+py-(d(vx+uy,x)+d(2*vy,y)+d(vz+wy,z))*spf.mu-spf.Fy	N/m³	Equation residual	Domains 1–6	
spf.res_w	spf.rho*wt+pz-(d(wx+uz,x)+d(wy+vz,y)+d(2*wz,z))*spf.mu-spf.Fz	N/m³	Equation residual	Domains 1–6	
spf.res_p	spf.rho*spf.divu	kg/(m³·s)	Pressure equation residual	Domains 1–6	

Shape functions

Name	Shape function	Unit	Description	Shape frame	Selection

Name	Shape function	Unit	Description	Shape frame	Selection
u	Lagrange (Linear)	m/s	Velocity field, x-component	Spatial	Domains 1–6
v	Lagrange (Linear)	m/s	Velocity field, y-component	Spatial	Domains 1–6
w	Lagrange (Linear)	m/s	Velocity field, z-component	Spatial	Domains 1–6
u	Lagrange (Linear)	m/s	Velocity field, x-component	Spatial	Domains 1–6
v	Lagrange (Linear)	m/s	Velocity field, y-component	Spatial	Domains 1–6
w	Lagrange (Linear)	m/s	Velocity field, z-component	Spatial	Domains 1–6
p	Lagrange (Linear)	Pa	Pressure	Spatial	Domains 1–6

Weak Expressions

Weak expression	Integration order	Integration frame	Selection
spf.rho*(-ut*test(u)-vt*test(v)-wt*test(w))	2	Spatial	Domains 1–6
(p-spf.K_stress_tensorxx)*test(ux)-spf.K_stress_tensorxy*test(uy)-spf.K_stress_tensorxz*test(uz)-spf.K_stress_tensoryx*test(vx)+(p-spf.K_stress_tensoryy)*test(vy)-spf.K_stress_tensoryz*test(vz)-spf.K_stress_tensorzx*test(wx)-spf.K_stress_tensorzy*test(wy)+(p-spf.K_stress_tensorzz)*test(wz)	2	Spatial	Domains 1–6
spf.Fx*test(u)+spf.Fy*test(v)+spf.Fz*test(w)	2	Spatial	Domains 1–6
-spf.continuityEquation*test(p)	2	Spatial	Domains 1–6
spf.streamlinens	2	Spatial	Domains 1–6

2.4.4 Initial Values 1

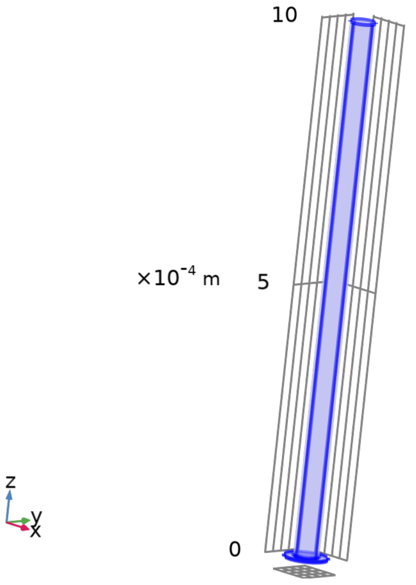

Initial Values 1

SELECTION

Geometric entity level	Domain
Selection	Geometry geom2: Dimension 3: All domains

Initial Values

SETTINGS

**Description**	**Value**	**Unit**
Velocity field, x-component	0	m/s
Velocity field, y-component	0	m/s
Velocity field, z-component	0	m/s
Pressure	0	Pa

Coordinate System Selection

SETTINGS

**Description**	**Value**
Coordinate system	Global coordinate system

Variables

**Name**	**Expression**	**Unit**	**Description**	**Selection**
spf.u_initx	0	m/s	Velocity field, x-component	Domains 1–6
spf.u_inity	0	m/s	Velocity field, y-component	Domains 1–6
spf.u_initz	0	m/s	Velocity field, z-component	Domains 1–6

Name	Expression	Unit	Description	Selection
spf.p_init	0	Pa	Pressure	Domains 1–6

2.4.5 Wall 1

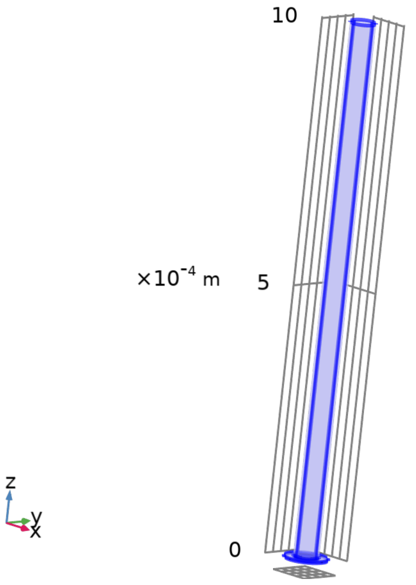

Wall 1

SELECTION

Geometric entity level	Boundary
Selection	Geometry geom2: Dimension 2: All boundaries

EQUATIONS

$$\mathbf{u} = \mathbf{0}$$

Boundary Condition

SETTINGS

Description	Value
Wall condition	No slip

Wall Movement

SETTINGS

Description	Value
Translational velocity	Automatic from frame
Sliding wall	Off

Variables

Name	Expression	Unit	Description	Selection	Details

Name	Expression	Unit	Description	Selection	Details
spf.ubndx	spf.utrx+spf.usx	m/s	Velocity at boundary, x-component	Boundaries 3–6, 8–9, 14, 16–17, 20–21, 24–25, 28–35, 37–38, 40, 42, 44, 46–52, 54–55, 57, 59, 61–62	
spf.ubndy	spf.utry+spf.usy	m/s	Velocity at boundary, y-component	Boundaries 3–6, 8–9, 14, 16–17, 20–21, 24–25, 28–35, 37–38, 40, 42, 44, 46–52, 54–55, 57, 59, 61–62	
spf.ubndz	spf.utrz+spf.usz	m/s	Velocity at boundary, z-component	Boundaries 3–6, 8–9, 14, 16–17, 20–21, 24–25, 28–35, 37–38, 40, 42, 44, 46–52, 54–55, 57, 59, 61–62	
spf.usx	0	m/s	Velocity of sliding wall, x-component	Boundaries 3–6, 8–9, 14, 16–17, 20–21, 24–25, 28–35, 37–38, 40, 42, 44, 46–52, 54–55, 57, 59, 61–62	
spf.usy	0	m/s	Velocity of sliding wall, y-component	Boundaries 3–6, 8–9, 14, 16–17, 20–21, 24–25, 28–35, 37–38, 40, 42, 44, 46–52, 54–55, 57, 59, 61–62	
spf.usz	0	m/s	Velocity of sliding wall, z-component	Boundaries 3–6, 8–9, 14, 16–17, 20–21, 24–25, 28–35, 37–38, 40, 42, 44, 46–52, 54–55, 57, 59, 61–62	
spf.utrx	0	m/s	Velocity of moving wall, x-component	Boundaries 3–6, 8–9, 14, 16–17, 20–21, 24–	

Name	Expression	Unit	Description	Selection	Details
				25, 28–35, 37–38, 40, 42, 44, 46–52, 54–55, 57, 59, 61–62	
spf.utry	0	m/s	Velocity of moving wall, y-component	Boundaries 3–6, 8–9, 14, 16–17, 20–21, 24–25, 28–35, 37–38, 40, 42, 44, 46–52, 54–55, 57, 59, 61–62	
spf.utrz	0	m/s	Velocity of moving wall, z-component	Boundaries 3–6, 8–9, 14, 16–17, 20–21, 24–25, 28–35, 37–38, 40, 42, 44, 46–52, 54–55, 57, 59, 61–62	
spf.uLeakagex	0	m/s	Leakage velocity, x-component	Boundaries 3–6, 8–9, 14, 16–17, 20–21, 24–25, 28–35, 37–38, 40, 42, 44, 46–52, 54–55, 57, 59, 61–62	+ operation
spf.uLeakagey	0	m/s	Leakage velocity, y-component	Boundaries 3–6, 8–9, 14, 16–17, 20–21, 24–25, 28–35, 37–38, 40, 42, 44, 46–52, 54–55, 57, 59, 61–62	+ operation
spf.uLeakagez	0	m/s	Leakage velocity, z-component	Boundaries 3–6, 8–9, 14, 16–17, 20–21, 24–25, 28–35, 37–38, 40, 42, 44, 46–52, 54–55, 57, 59, 61–62	+ operation
spf.noSlipWall	1	1	Help variable	Boundaries 3–6, 8–9, 14, 16–17, 20–21, 24–25, 28–35, 37–38, 40, 42, 44, 46–52, 54–55,	

Name	Expression	Unit	Description	Selection	Details
				57, 59, 61–62	

Constraints

Constraint	Constraint force	Shape function	Selection	Details
-u+spf.ubndx+spf.uLeakagex	test(-u)	Lagrange (Linear)	Boundaries 3–6, 8–9, 14, 16–17, 20–21, 24–25, 28–35, 37–38, 40, 42, 44, 46–52, 54–55, 57, 59, 61–62	Elemental
-v+spf.ubndy+spf.uLeakagey	test(-v)	Lagrange (Linear)	Boundaries 3–6, 8–9, 14, 16–17, 20–21, 24–25, 28–35, 37–38, 40, 42, 44, 46–52, 54–55, 57, 59, 61–62	Elemental
-w+spf.ubndz+spf.uLeakagez	test(-w)	Lagrange (Linear)	Boundaries 3–6, 8–9, 14, 16–17, 20–21, 24–25, 28–35, 37–38, 40, 42, 44, 46–52, 54–55, 57, 59, 61–62	Elemental

2.4.6 Pipette Back (Open Boundary)

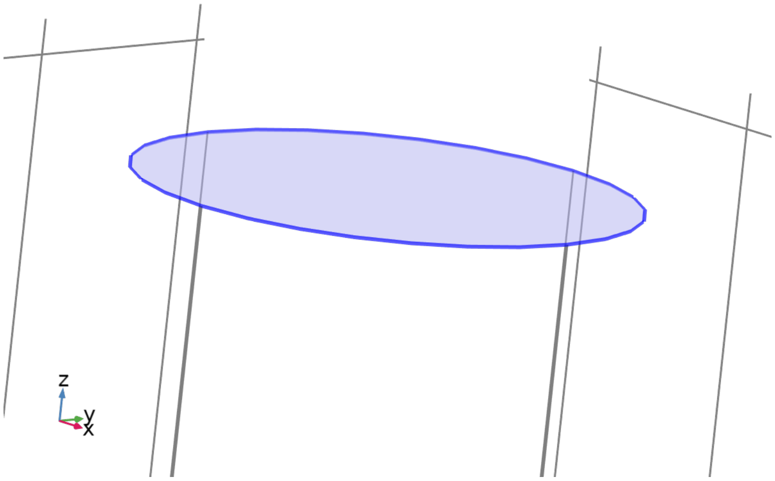

Pipette Back (Open Boundary)

SELECTION

Geometric entity level	Boundary
Selection	Geometry geom2: Dimension 2: Boundary 11

EQUATIONS

$$[-p\mathbf{I}+\mathbf{K}]\mathbf{n}=-f_0\mathbf{n}$$

Boundary Condition

SETTINGS

Description	Value	Unit
Boundary condition	Normal stress	
Normal stress	0	N/m²

Variables

Name	Expression	Unit	Description	Selection
spf.f0	0	N/m²	Normal stress	Boundary 11
spf.open1.volumeFlowRate	spf.open1.intop(u*spf.nxmesh+v*spf.nymesh+w*spf.nzmesh)	m³/s	Outward volume flow rate across feature selection	Global
spf.open1.massFlowRate	spf.open1.intop(spf.rho*(u*spf.nxmesh+v*spf.nymesh+w*spf.nzmesh))	kg/s	Outward mass flow rate across feature selection	Global
spf.open1.pAverage	spf.open1.aveop(p)	Pa	Pressure average over feature selection	Global

Weak Expressions

Weak expression	Integration order	Integration frame	Selection
-spf.f0*(test(u)*spf.nxmesh+test(v)*spf.nymesh+test(w)*spf.nzmesh)	2	Spatial	Boundary 11

2.4.7 Meniscus (Velocity)

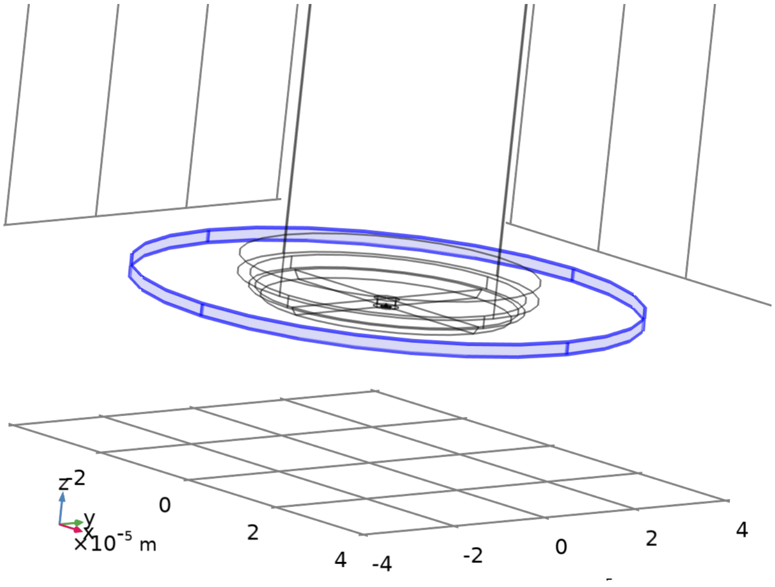

Meniscus (Velocity)

SELECTION

Geometric entity level	Boundary
Selection	Geometry geom2: Dimension 2: Boundaries 1–2, 36, 63

EQUATIONS

$$\mathbf{u} = U_0\mathbf{n}$$

Boundary Condition

SETTINGS

Description	Value
Boundary condition	Velocity

Velocity

SETTINGS

Description	Value	Unit
Velocity field componentwise	Normal outflow velocity	
Normal outflow velocity	v_dry	m/s

Constraint Settings

SETTINGS

Description	Value
Apply reaction terms on	All physics (symmetric)

Description	Value
Use weak constraints	Off
Constraint method	Elemental

Variables

Name	Expression	Unit	Description	Selection
spf.ubndx	spf.nx*spf.U0out	m/s	Velocity at boundary, x-component	Boundaries 1–2, 36, 63
spf.ubndy	spf.ny*spf.U0out	m/s	Velocity at boundary, y-component	Boundaries 1–2, 36, 63
spf.ubndz	spf.nz*spf.U0out	m/s	Velocity at boundary, z-component	Boundaries 1–2, 36, 63
spf.U0out	v_dry	m/s	Normal outflow velocity	Boundaries 1–2, 36, 63
spf.out1.Uav	0	m/s	Average velocity	Global
spf.out1.Uavfdf	0	m/s	Average velocity	Global
spf.out1.dz	spf.dz	m	Channel thickness	Boundaries 1–2, 36, 63
spf.out1.Mflow	spf.out1.massFlowRate	kg/s	Mass flow	Global
spf.out1.volumeFlowRate	spf.out1.intop(u*spf.nxmesh+v*spf.nymesh+w*spf.nzmesh)	m^3/s	Outward volume flow rate across feature selection	Global
spf.out1.massFlowRate	spf.out1.intop(spf.rho*(u*spf.nxmesh+v*spf.nymesh+w*spf.nzmesh))	kg/s	Outward mass flow rate across feature selection	Global
spf.out1.pAverage	spf.out1.aveop(p)	Pa	Pressure average over feature selection	Global

Constraints

Constraint	Constraint force	Shape function	Selection	Details
-u+spf.ubndx	test(-u+spf.ubndx)	Lagrange (Linear)	Boundaries 1–2, 36, 63	Elemental

Constraint	Constraint force	Shape function	Selection	Details
-v+spf.ubndy	test(-v+spf.ubndy)	Lagrange (Linear)	Boundaries 1–2, 36, 63	Elemental
-w+spf.ubndz	test(-w+spf.ubndz)	Lagrange (Linear)	Boundaries 1–2, 36, 63	Elemental

2.5 EVENTS

USED PRODUCTS

COMSOL Multiphysics

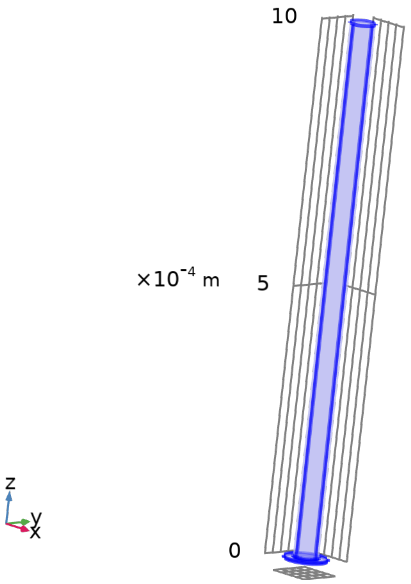

Events

SELECTION

Geometric entity level	Domain
Selection	Geometry geom2: Dimension 3: Domains 1–7

2.5.1 Interface Settings

Discretization

SETTINGS

Description	Value
Element order	Quadratic

SETTINGS

Description	Value
Equation form	Study controlled

2.5.2 Discrete States 1

SELECTION

Geometric entity level	Entire model

Discrete States

Name	Initial value (u0)	Description
E	-0.3[V]	

Shape functions

Name	Shape function	Unit	Description	Shape frame	Selection
E	ODE		Discrete state, -component		Global

2.5.3 Explicit Event 1

SELECTION

Geometric entity level	Entire model

Event Timings

SETTINGS

Description	Value	Unit
Start of event	15	s
Period of event	Inf	s
Use consistent initialization	On	

Reinitialization

Variable	Expression
E	0.3

2.6 MULTIPHYSICS

2.6.1 Flow Coupling 1

USED PRODUCTS

COMSOL Multiphysics

Coupled Interfaces

SETTINGS

Description	Value
Source	Creeping Flow (spf)
Destination	Transport of Diluted Species (tds2)

Variables

Name	Expression	Unit	Description	Selection
fc1.uX	spatial.invF11*u+spatial.invF21*v+spatial.invF31*w	m/s	Velocity field, X-component	Global
fc1.uY	spatial.invF12*u+spatial.invF22*v+spatial.invF32*w	m/s	Velocity field, Y-component	Global
fc1.uZ	spatial.invF13*u+spatial.invF23*v+spatial.invF33*w	m/s	Velocity field, Z-component	Global
fc1.p	p	Pa	Pressure	Global
fc1.pA	spf.pA	Pa	Absolute pressure	Global

2.7 MESH 2

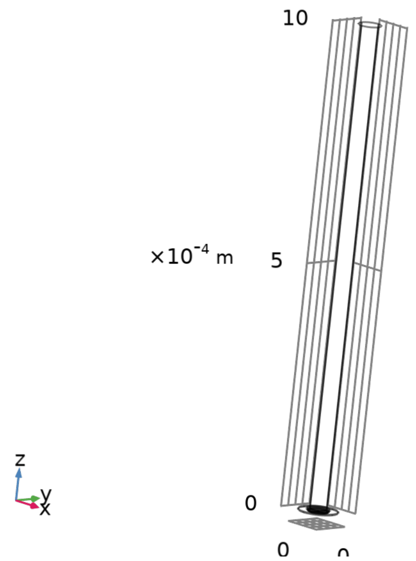

Mesh 2

2.7.1 Size (size)

SETTINGS

Description	Value
Maximum element size	4E-5
Minimum element size	1E-7
Curvature factor	0.25
Maximum element growth rate	1.2
Predefined size	Extra fine
Custom element size	Custom

2.7.2 Size- UME edge (size1)

SELECTION

Geometric entity level	Edge
Selection	Geometry geom2: Dimension 1: Edges 34, 36, 66, 81

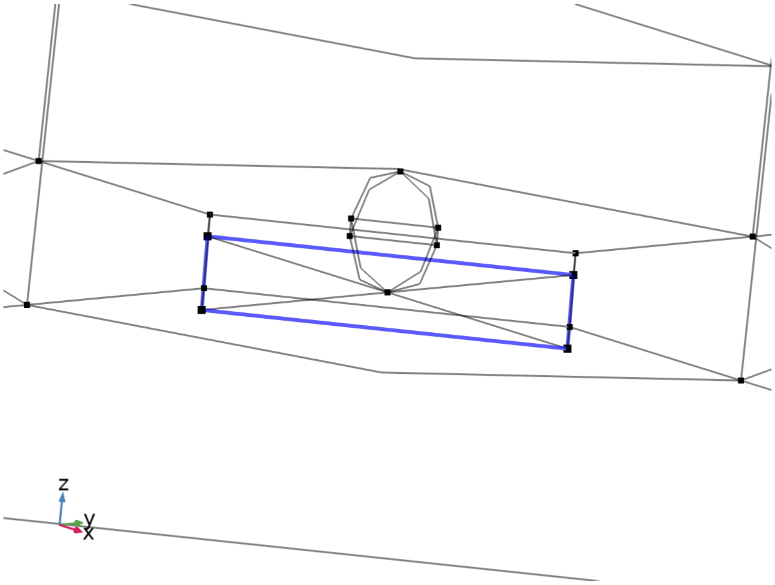

Size- UME edge

SETTINGS

Description	Value
Maximum element size	5E-9
Minimum element size	5E-10
Curvature factor	0.6
Curvature factor	Off
Resolution of narrow regions	0.5
Resolution of narrow regions	Off
Maximum element growth rate	1.5
Maximum element growth rate	Off
Custom element size	Custom

2.7.3 particle surfaces (size2)

SELECTION

Geometric entity level	Boundary
Selection	Geometry geom2: Dimension 2: Boundaries 32–35, 48–50, 52

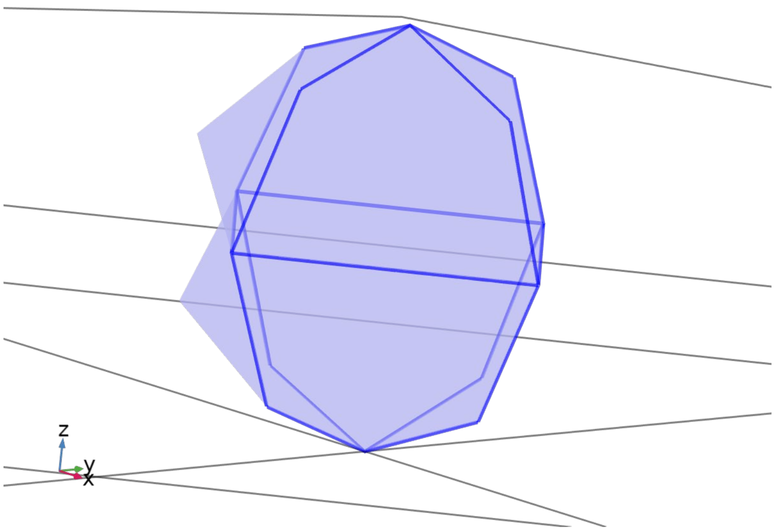

particle surfaces

SETTINGS

Description	Value
Maximum element size	2.5E-8
Minimum element size	5E-10
Curvature factor	0.6
Curvature factor	Off
Resolution of narrow regions	0.5
Resolution of narrow regions	Off
Maximum element growth rate	1.5
Maximum element growth rate	Off
Custom element size	Custom

2.7.4 Size- UME domain (size10)

SELECTION

Geometric entity level	Domain
Selection	Geometry geom2: Dimension 3: Domains 6–7

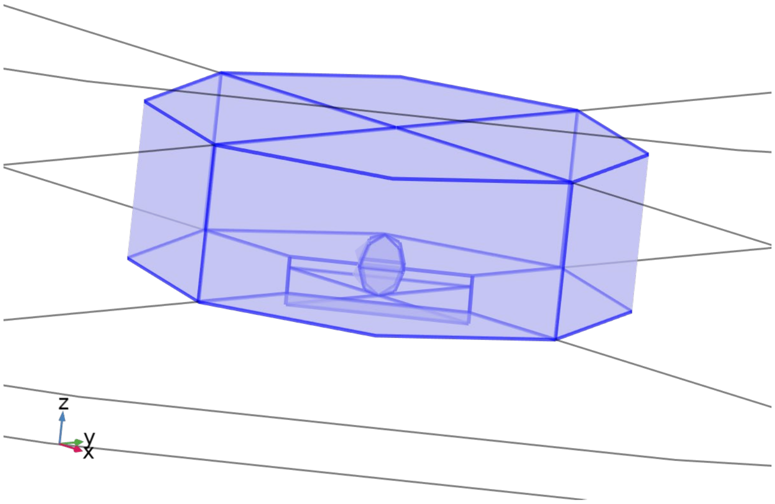

Size- UME domain

SETTINGS

Description	Value
Maximum element size	1E-7
Minimum element size	1E-9
Curvature factor	0.6
Curvature factor	Off
Resolution of narrow regions	0.5
Resolution of narrow regions	Off
Maximum element growth rate	1.2
Custom element size	Custom

2.7.5 Size- Outer domain 2 (size12)

SELECTION

Geometric entity level	Domain
Selection	Geometry geom2: Dimension 3: Domains 2, 4–5

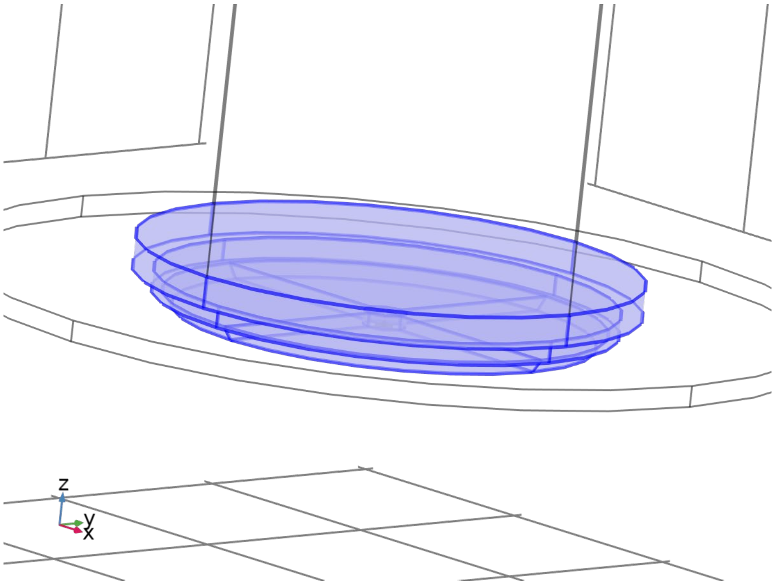

Size- Outer domain 2

SETTINGS

Description	Value
Maximum element size	7E-7
Minimum element size	2E-8
Curvature factor	0.6
Curvature factor	Off
Resolution of narrow regions	0.5
Resolution of narrow regions	Off
Maximum element growth rate	1.2
Maximum element growth rate	Off
Custom element size	Custom

2.7.6 Size- Near meniscus (size11)

SELECTION

Geometric entity level	Domain
Selection	Geometry geom2: Dimension 3: Domain 1

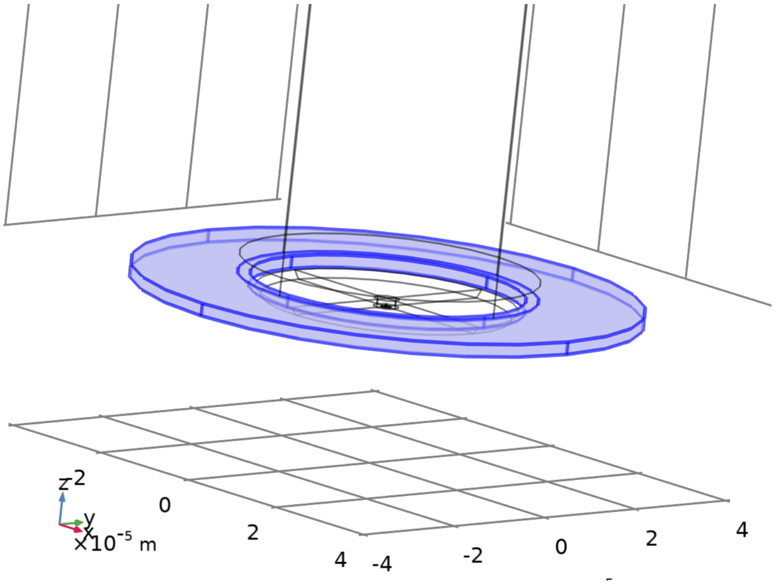

Size- Near meniscus

SETTINGS

Description	Value
Maximum element size	1.8E-6
Minimum element size	1E-8
Curvature factor	0.6
Curvature factor	Off
Resolution of narrow regions	0.5
Resolution of narrow regions	Off
Maximum element growth rate	1.25
Custom element size	Custom

2.7.7 Free Tetrahedral 1 (ftet1)

SELECTION

Geometric entity level	Domain
Selection	Geometry geom2: Dimension 3: Domains 1–7

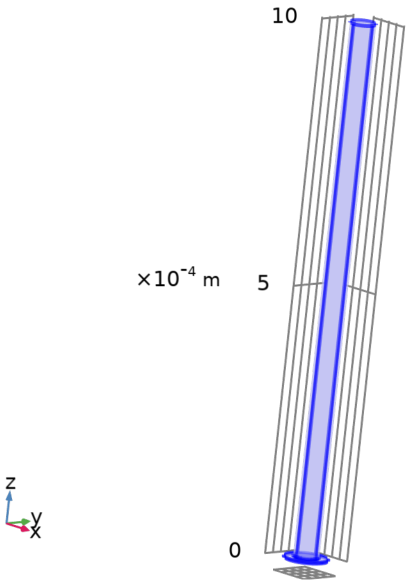

Free Tetrahedral 1

SETTINGS

Description	Value
Last build time	22
Built with	COMSOL 6.1.0.357 (win64)|2024 - 01 - 27T09:36:19.776305200

3 Study 1

COMPUTATION INFORMATION

Computation time	1 h 18 min 34 s

3.1 TIME DEPENDENT

Times	Unit
range(0,0.2,25)	s

STUDY SETTINGS

Description	Value
Include geometric nonlinearity	Off

STUDY SETTINGS

Description	Value
Output times	{0, 0.2, 0.4, 0.6, 0.8, 1, 1.2, 1.4, 1.6, 1.8, 2, 2.2, 2.4, 2.6, 2.8, 3, 3.2, 3.4, 3.6, 3.8, 4, 4.2, 4.4, 4.6, 4.8, 5, 5.2, 5.4, 5.6, 5.8, 6, 6.2, 6.4, 6.6, 6.8, 7, 7.2, 7.4, 7.6, 7.8, 8, 8.2, 8.4, 8.6, 8.8, 9, 9.2, 9.4, 9.6, 9.8, 10, 10.2, 10.4, 10.6, 10.8, 11, 11.2, 11.4, 11.6, 11.8, 12, 12.2, 12.4, 12.6, 12.8, 13, 13.2, 13.4, 13.6, 13.8, 14, 14.2, 14.4, 14.6, 14.8, 15, 15.2, 15.4, 15.6, 15.8, 16, 16.2, 16.4, 16.6, 16.8, 17, 17.2, 17.4, 17.6, 17.8, 18, 18.2, 18.4, 18.6, 18.8, 19, 19.2, 19.4, 19.6, 19.8, 20, 20.2, 20.4, 20.6, 20.8, 21, 21.2, 21.4, 21.6, 21.8, 22, 22.2, 22.4, 22.6, 22.8, 23, 23.2, 23.4, 23.6, 23.8, 24, 24.2, 24.4, 24.6, 24.8, 25}

PHYSICS AND VARIABLES SELECTION

Physics interface	Solve for	Equation form
Transport of Diluted Species (tds2)	On	Automatic (Time dependent)
Creeping Flow (spf)	On	Automatic (Time dependent)
Events (ev)	On	Automatic (Time dependent)

PHYSICS AND VARIABLES SELECTION

Multiphysics couplings	Solve for	Equation form
Flow Coupling 1 (fc1)	On	Automatic (Time dependent)

MESH SELECTION

Component	Mesh
Component 2	Mesh 2

3.2 SOLVER CONFIGURATIONS

3.2.1 Solution 1

Compile Equations: Time Dependent (st1)

STUDY AND STEP

Description	Value
Use study	Study 1
Use study step	Time Dependent

Dependent Variables 1 (v1)

GENERAL

Description	Value
Defined by study step	Time Dependent

RESIDUAL SCALING

Description	Value
Method	Manual

INITIAL VALUE CALCULATION CONSTANTS

Constant name	Initial value source
t	range(0,0.2,25)
timestep	0.025[s]

Concentration (comp2.cO) (comp2_cO)

GENERAL

Description	Value
Field components	comp2.cO
Internal variables	{comp2.uflux.cO, comp2.dflux.cO, comp2.tds2.dt2Inv_cO}

Concentration (comp2.cR) (comp2_cR)

GENERAL

Description	Value
Field components	comp2.cR
Internal variables	{comp2.uflux.cR, comp2.dflux.cR, comp2.tds2.dt2Inv_cR}

Pressure (comp2.p) (comp2_p)

GENERAL

Description	Value
Field components	comp2.p

Velocity field (comp2.u) (comp2_u)

GENERAL

Description	Value
Field components	{comp2.u, comp2.v, comp2.w}
Internal variables	{comp2.spf.dt2Inv_u, comp2.spf.isFluidHasBeenSolved}

Discrete state (comp2.ev.ds1.dim) (comp2_ev_ds1_dim)

GENERAL

Description	Value
State components	comp2.E

Time-Dependent Solver 1 (t1)

GENERAL

Description	Value
Defined by study step	Time Dependent
Output times	{0, 0.2, 0.4, 0.6, 0.8, 1, 1.2, 1.4, 1.6, 1.8, 2, 2.2, 2.4, 2.6, 2.8, 3, 3.2, 3.4, 3.6, 3.8, 4, 4.2, 4.4, 4.6, 4.8, 5, 5.2, 5.4, 5.6, 5.8, 6, 6.2, 6.4, 6.6, 6.8, 7, 7.2, 7.4, 7.6, 7.8, 8, 8.2, 8.4, 8.6, 8.8, 9, 9.2, 9.4, 9.6, 9.8, 10, 10.2, 10.4, 10.6, 10.8, 11, 11.2, 11.4, 11.6, 11.8, 12, 12.2, 12.4, 12.6, 12.8, 13, 13.2, 13.4, 13.6, 13.8, 14, 14.2, 14.4, 14.6, 14.8, 15, 15.2, 15.4, 15.6, 15.8, 16, 16.2, 16.4, 16.6, 16.8, 17, 17.2, 17.4, 17.6, 17.8, 18, 18.2, 18.4, 18.6, 18.8, 19, 19.2, 19.4, 19.6, 19.8, 20, 20.2, 20.4, 20.6, 20.8, 21, 21.2, 21.4, 21.6, 21.8, 22, 22.2, 22.4, 22.6, 22.8, 23, 23.2, 23.4, 23.6, 23.8, 24, 24.2, 24.4, 24.6, 24.8, 25}
Relative tolerance	0.005

ABSOLUTE TOLERANCE

Description	Value
Tolerance factor	0.05

ABSOLUTE TOLERANCE

Field	Method	Tolerance method	Tolerance factor	Derivative tolerance method	Tolerance for time derivatives	Tolerance	Tolerance for time derivatives
Concentration (comp2.cO)	Use global	Factor	0.1	Automatic	1	0.001	0.001
Concentration (comp2.cR)	Use global	Factor	0.1	Automatic	1	0.001	0.001

Field	Method	Tolerance method	Tolerance factor	Derivative tolerance method	Tolerance for time derivatives	Tolerance	Tolerance for time derivatives
Pressure (comp2.p)	Scaled	Factor	1	Automatic	1	0.001	0.001
Velocity field (comp2.u)	Use global	Factor	0.1	Automatic	1	0.001	0.001
Discrete state (comp2.ev.ds1.dim)	Use global	Factor	0.1	Automatic	1	0.001	0.001

TIME STEPPING

Description	Value
Maximum BDF order	2
Nonlinear controller	On
Fraction of initial step for Backward Euler	0.01
Error estimation	Exclude algebraic

Advanced (aDef)

ASSEMBLY SETTINGS

Description	Value
Reuse sparsity pattern	On

Segregated 1 (se1)

GENERAL

Description	Value
Tolerance factor	0.5
Stabilization and acceleration	Anderson acceleration
Dimension of iteration space	5
Mixing parameter	0.9

Events (ss1)

GENERAL

Description	Value
Variables	Discrete state (comp2.ev.ds1.dim)
Linear solver	Direct

Velocity u, Pressure p (ss2)

GENERAL

Description	Value
Variables	{Velocity field (comp2.u), Pressure (comp2.p)}
Linear solver	AMG, fluid flow variables (spf)

METHOD AND TERMINATION

Description	Value
Damping factor	0.8
Jacobian update	Once per time step

Concentrations (ss3)

GENERAL

Description	Value
Variables	{Concentration (comp2.cR), Concentration (comp2.cO)}
Linear solver	AMG, concentrations (tds2)

METHOD AND TERMINATION

Description	Value
Damping factor	0.8
Jacobian update	Once per time step

AMG, fluid flow variables (spf) (i1)

GENERAL

Description	Value
Maximum number of iterations	100

ERROR

Description	Value
Factor in error estimate	20

Multigrid 1 (mg1)

GENERAL

Description	Value
Solver	Smoothed aggregation AMG
Maximum number of DOFs at coarsest level	80000
Strength of connections	0.02
Construct prolongators componentwise	On
Prolongator smoothing	Off

Presmoother (pr)

SCGS 1 (sc1)

MAIN

Description	Value
Sweep type	SSOR
Number of iterations	0

Postsmoother (po)

SCGS 1 (sc1)

MAIN

Description	Value
Sweep type	SSOR
Number of iterations	1

Coarse Solver (cs)

Direct 1 (d1)

GENERAL

Description	Value
Solver	PARDISO
Pivoting perturbation	1E-13

AMG, concentrations (tds2) (i2)

GENERAL

Description	Value
Maximum number of iterations	50

Multigrid 1 (mg1)

GENERAL

Description	Value
Solver	Smoothed aggregation AMG
Maximum number of DOFs at coarsest level	50000
Construct prolongators componentwise	On
Prolongator smoothing	Off

Presmoother (pr)

SOR Line 1 (sl1)

MAIN

Description	Value
Sweep type	SSOR

Description	Value
Number of iterations	1
Relaxation factor	0.7

SECONDARY

Description	Value
Relaxation factor	0.5

Postsmoother (po)

SOR Line 1 (sl1)

MAIN

Description	Value
Sweep type	SSOR
Number of iterations	1
Relaxation factor	0.7

SECONDARY

Description	Value
Relaxation factor	0.5

Coarse Solver (cs)

Direct 1 (d1)

GENERAL

Description	Value
Solver	PARDISO
Pivoting perturbation	1E-13

3.2.2 Parametric Solutions 1

de=1.2E-6 (su1)

GENERAL

Description	Value
Solution	de=1.2E-6

de=2.4E-6 (su2)

GENERAL

Description	Value
Solution	de=2.4E-6

de=4.8E-6 (su3)

GENERAL

Description	Value
Solution	de=4.8E-6

3.2.3 Parametric Solutions 2

v_dry=0, blockloc=1.3E-6 (su1)

GENERAL

Description	Value
Solution	v_dry=0, blockloc=1.3E-6

v_dry=0, blockloc=1.4E-6 (su2)

GENERAL

Description	Value
Solution	v_dry=0, blockloc=1.4E-6

v_dry=6E-6, blockloc=1.3E-6 (su3)

GENERAL

Description	Value
Solution	v_dry=6E-6, blockloc=1.3E-6

v_dry=6E-6, blockloc=1.4E-6 (su4)

GENERAL

Description	Value
Solution	v_dry=6E-6, blockloc=1.4E-6

4 Results

4.1 DATA SETS

4.1.1 Study 1/Solution 1

SOLUTION

Description	Value
Solution	Solution 1
Component	Component 2 (comp2)
Frame	Material (X, Y, Z)

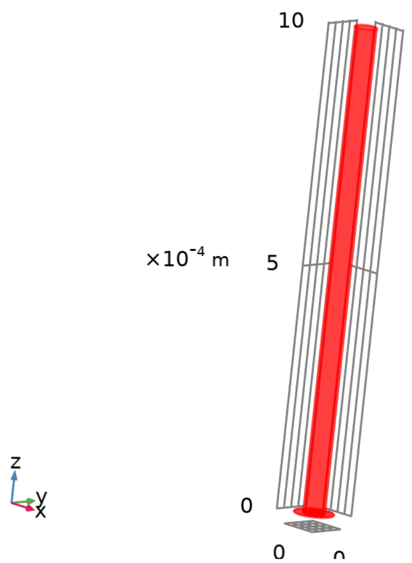

Dataset: Study 1/Solution 1

4.1.2 Study 1/Parametric Solutions 1

SOLUTION

Description	Value
Solution	Parametric Solutions 1
Component	Component 2 (comp2)
Frame	Material (X, Y, Z)

4.1.3 Study 1/Parametric Solutions 2

SOLUTION

Description	Value
Solution	Parametric Solutions 2

Description	Value
Component	Component 2 (comp2)

4.1.4 Exterior Walls

DATA

Description	Value
Dataset	Study 1/Parametric Solutions 2

PARAMETERIZATION

Description	Value
x- and y-axes	Surface parameters

4.2 PLOT GROUPS

4.2.1 Concentration (tds2)

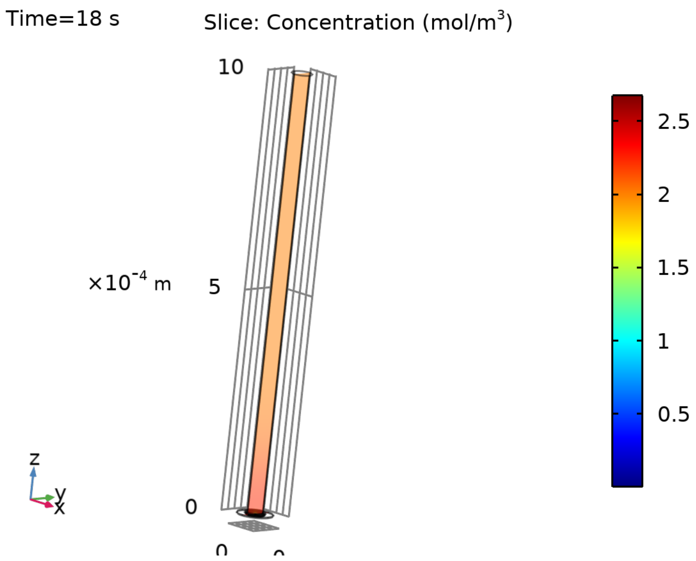

Slice: Concentration (mol/m^3)

4.2.2 Flux

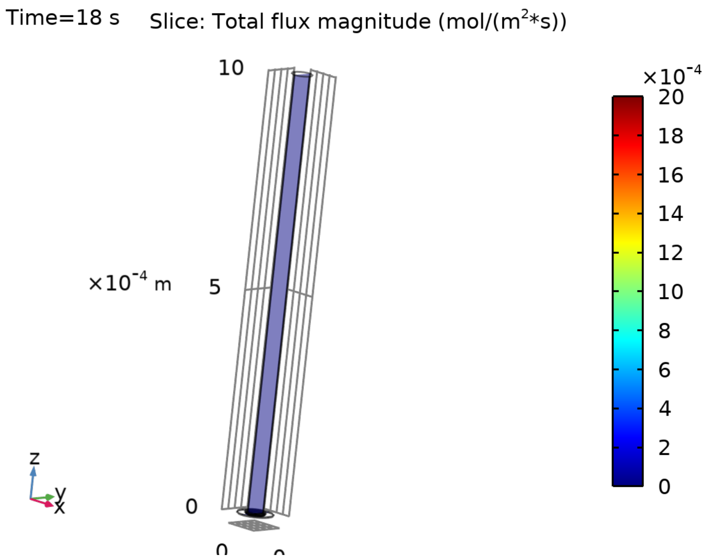

*Slice: Total flux magnitude (mol/(m²*s))*

4.2.3 Velocity (spf)

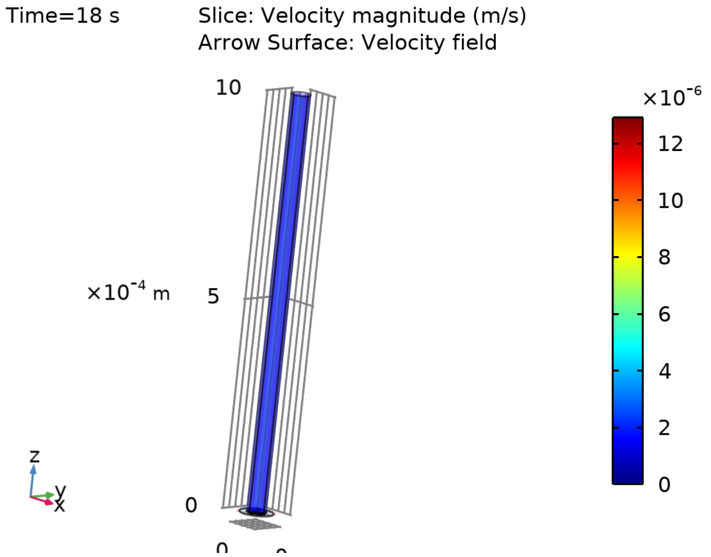

Slice: Velocity magnitude (m/s) Arrow Surface: Velocity field

4.3 EVALUATION GROUPS

4.3.1 Evaluation Group 1

DATA

Description	Value

Description	Value
Dataset	Study 1/Solution 1

FEATURES

Feature	Column
Surface Integration 1	Time (s)

RESULTS

Time (s)	tds2.ntflux_cR*F (A)
0	7.1349E-15
0.2	4.8184E-15
0.4	4.2769E-15
0.6	4.1929E-15
0.8	4.2201E-15
1	4.2531E-15
1.2	4.281E-15
1.4	4.2705E-15
1.6	4.3129E-15
1.8	4.2977E-15
2	4.2824E-15
2.2	4.2671E-15
2.4	4.2971E-15
2.6	4.2866E-15
2.8	4.2761E-15
3	4.2657E-15
3.2	4.3034E-15
3.4	4.2976E-15
3.6	4.2919E-15
3.8	4.2861E-15
4	4.2803E-15
4.2	4.2745E-15
4.4	4.2687E-15
4.6	4.2629E-15
4.8	4.286E-15
5	4.282E-15
5.2	4.2781E-15
5.4	4.2741E-15

Time (s)	tds2.ntflux_cR*F (A)
5.6	4.2702E-15
5.8	4.2662E-15
6	4.2622E-15
6.2	4.2583E-15
6.4	4.2736E-15
6.6	4.2708E-15
6.8	4.268E-15
7	4.2653E-15
7.2	4.2625E-15
7.4	4.2597E-15
7.6	4.2569E-15
7.8	4.2542E-15
8	4.2702E-15
8.2	4.2682E-15
8.4	4.2663E-15
8.6	4.2643E-15
8.8	4.2624E-15
9	4.2604E-15
9.2	4.2585E-15
9.4	4.2565E-15
9.6	4.2546E-15
9.8	4.2526E-15
10	4.2507E-15
10.2	4.2487E-15
10.4	4.2596E-15
10.6	4.2581E-15
10.8	4.2566E-15
11	4.2551E-15
11.2	4.2536E-15
11.4	4.2521E-15
11.6	4.2507E-15
11.8	4.2492E-15
12	4.2477E-15
12.2	4.2462E-15
12.4	4.2447E-15

Time (s)	tds2.ntflux_cR*F (A)
12.6	4.2432E-15
12.8	4.2418E-15
13	4.2472E-15
13.2	4.246E-15
13.4	4.2447E-15
13.6	4.2434E-15
13.8	4.2421E-15
14	4.2409E-15
14.2	4.2396E-15
14.4	4.2383E-15
14.6	4.2371E-15
14.8	4.2358E-15
15	4.2345E-15
15.2	6.0538E-10
15.4	5.9999E-10
15.6	5.9632E-10
15.8	5.9331E-10
16	5.9072E-10
16.2	5.8843E-10
16.4	5.8633E-10
16.6	5.8438E-10
16.8	5.8257E-10
17	5.8088E-10
17.2	5.7931E-10
17.4	5.7783E-10
17.6	5.7644E-10
17.8	5.7513E-10
18	5.7385E-10
18.2	5.7264E-10
18.4	5.7149E-10
18.6	5.7041E-10
18.8	5.6935E-10
19	5.6833E-10
19.2	5.6736E-10
19.4	5.6644E-10

Time (s)	tds2.ntflux_cR*F (A)
19.6	5.6556E-10
19.8	5.647E-10
20	5.6386E-10
20.2	5.6305E-10
20.4	5.6228E-10
20.6	5.6154E-10
20.8	5.608E-10
21	5.6009E-10
21.2	5.594E-10
21.4	5.5874E-10
21.6	5.5809E-10
21.8	5.5744E-10
22	5.5681E-10
22.2	5.562E-10
22.4	5.556E-10
22.6	5.5502E-10
22.8	5.5445E-10
23	5.539E-10
23.2	5.5336E-10
23.4	5.5284E-10
23.6	5.5232E-10
23.8	5.518E-10
24	5.513E-10
24.2	5.5081E-10
24.4	5.5034E-10
24.6	5.4987E-10
24.8	5.4942E-10
25	5.4897E-10

Surface Integration 1

EXPRESSIONS

Expression	Unit	Description
tds2.ntflux_cR*F	A	

INTEGRATION SETTINGS

Description	Value

Description	Value
Integration order	4
