# Supplementary material for: High-Throughput Single-Entity Electrochemistry with Microelectrode Arrays
Source: Anal Chem. 2024 May 23;96(22):9177–84. doi: 10.1021/acs.analchem.4c01092 (PMC11154736; doi:10.1021/acs.analchem.4c01092)
Supplement: Supplementary file 4 — ac4c01092_si_004.pdf [file ac4c01092_si_004.pdf]

AMCM2D convection 2.1um CV

Report date	Jan 11, 2024, 6:27:42 PM

Contents

1. Global Definitions 3

1.1. Parameters 3

1.2. Shared Properties 4

2. Component 1 5

2.1. Definitions 5

2.2. Geometry 1 5

2.3. Creeping Flow 7

2.4. Electrostatics 30

2.5. Electroanalysis 47

2.6. Multiphysics 70

2.7. Mesh 1 72

3. Study 1 80

3.1. Parametric Sweep 80

3.2. Cyclic Voltammetry 80

3.3. Time Dependent 81

3.4. Solver Configurations 81

4. Results 86

4.1. Data Sets 86

4.2. Derived Values 92

4.3. Tables 92

4.4. Plot Groups 94

1 Global Definitions

Date	Jan 11, 2024, 6:18:02 PM

GLOBAL SETTINGS

Name	SA 2024-1-11 AMCM2D convection 2.1um CV for report out.mph
Path	/home/sealden/SA_2024-1-11_AMCM2D_convection_2.1um_CV_for report out.mph
Version	COMSOL Multiphysics 6.1 (Build: 252)

USED PRODUCTS

COMSOL Multiphysics
Electrochemistry Module

COMPUTER INFORMATION

CPU	AMD EPYC 7402P 24-Core Processor, 24 cores, 124.89 GB RAM
Operating system	Linux

1.1 PARAMETERS

PARAMETERS 1

Name	Expression	Value	Description
re	1.05E-6 [m]	1.05E−6 m	UME radius
de	1.3E-6 [m]	1.3E−6 m	photoresist recession depth
rpipet	(35E-6 [m])/2	1.75E−5 m	inner radius of pipet
hpipet	2E-6 [m]	2E−6 m	pip-subs spacing (Dps)
taper	0.001 [deg]	1.7453E−5 rad	pipet taper angle
pipetshank	1E-3 [m]	0.001 m	length of pipet simulated
pipetOR	(61 [um])/2	3.05E−5 m	outer radius of pipet (w/ wall)
recOR	photo + 5E-7 [m]	2.05E−5 m	outer radius of recession
vdry	8E-6 [m/s]	8E−6 m/s	drying outlet velocity
E	0.3 [V]	0.3 V	electrode potential
Ef	0 [V]	0 V	formal potential
cRbulk	0.002 [M]	2 mol/m^3	bulk conc of R
F	9.64853E4 [C/mol]	96485 C/mol	Farday constant
f	38.92 [1/V]	38.92 1/V	F/RT
DR	7.4E-6 [(cm^2)/s]	7.4E−10 m^2/s	diffusion coefficient of R
DO	DR	7.4E−10 m^2/s	diff co of O
a	0.5	0.5	alpha
k0	10 [cm/s]	0.1 m/s	e-t rate constant

Name	Expression	Value	Description
photo	20 [um]	2E−5 m	photoresist hole radius
SiNx	0.09 [um]	9E−8 m	SiNx hole depth
t_acc	15 [s]	15 s	accumulation time
t_step	10 [s]	10 s	i-t curve length
E_start	-0.4 [V]	−0.4 V	
E_vertex	0.4 [V]	0.4 V	
v_sweep	0.1 [V/s]	0.1 V/s	scan rate

1.2 SHARED PROPERTIES

1.2.1 Default Model Inputs

Tag	cminpt

2 Component 1

SETTINGS

Description	Value
Unit system	Same as global system (SI)
Avoid inverted elements by curving interior domain elements	Off

2.1 DEFINITIONS

2.1.1 Coordinate Systems

Boundary System 1

Coordinate system type	Boundary system
Tag	sys1

COORDINATE NAMES

First	Second	Third
t1	to	n

2.2 GEOMETRY 1

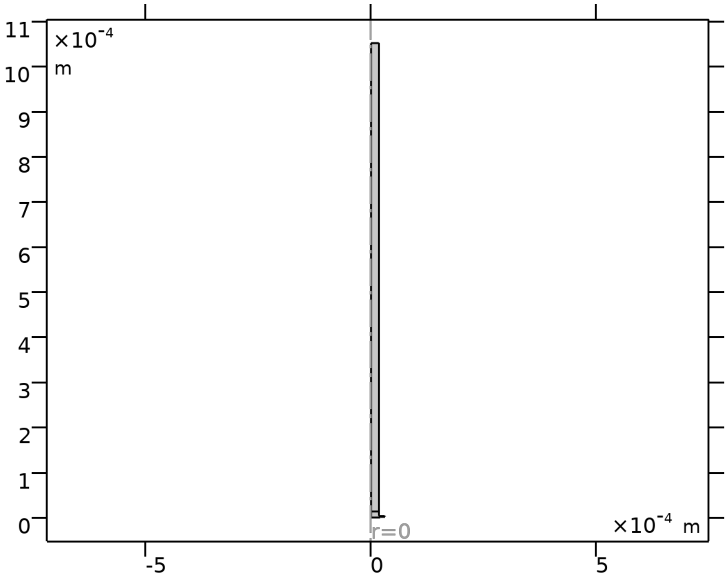

Geometry 1

UNITS

Length unit	m
Angular unit	deg

GEOMETRY STATISTICS

Description	Value
Space dimension	2
Number of domains	4
Number of boundaries	18
Number of vertices	15

2.2.1 Polygon 1 (pol1)

OBJECT TYPE

Description	Value
Type	Solid

COORDINATES

Description	Value
Data source	Table

COORDINATES

r (m)	z (m)
0	0
re	0
re	SiNx
photo	SiNx
recOR	SiNx+de
pipetOR	SiNx+de
pipetOR	SiNx+de+hpipet
rpipet	SiNx+de+hpipet
rpipet	de+hpipet+pipetshank+(50E-6[m]-hpipet)+SiNx
0	de+hpipet+pipetshank+(50E-6[m]-hpipet)+SiNx

2.2.2 Circle 1 (c1)

POSITION

Description	Value
Position	{0, 0}

SIZE AND SHAPE

Description	Value
Radius	re*1.5
Sector angle	90

2.2.3 Line Segment 1 (ls1)

SETTINGS

Description	Value
Specify	Coordinates
Coordinates	{0, SiNx + de+hpipet + 10[um]}
Specify	Coordinates
Coordinates	{rpipet, SiNx + de+hpipet + 10[um]}

2.3 CREEPING FLOW

USED PRODUCTS

COMSOL Multiphysics
Electrochemistry Module

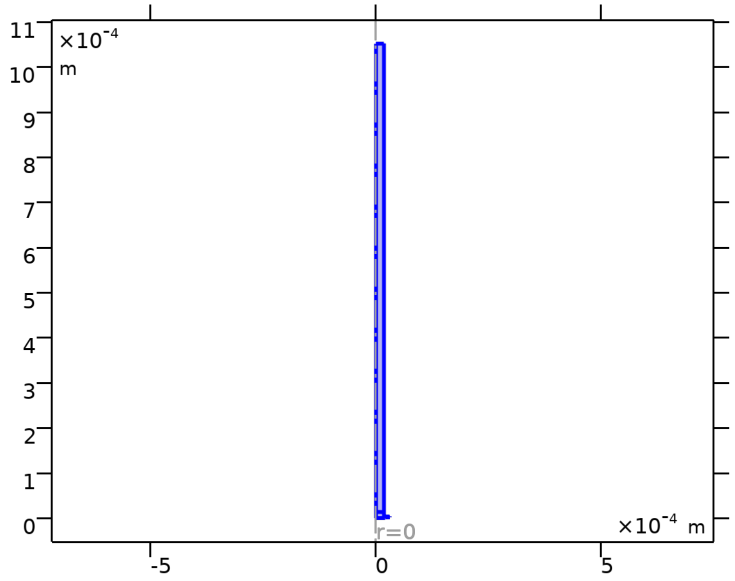

Creeping Flow

SELECTION

Geometric entity level	Domain
Selection	Geometry geom1: Dimension 2: Domains 1–3

EQUATIONS

$$\rho\frac{\partial \mathbf{u}}{\partial t} = \nabla \cdot [-p\mathbf{I} + \mathbf{K}] + \mathbf{F}$$
$$\rho\nabla \cdot \mathbf{u} = 0$$

2.3.1 Interface Settings

Discretization

SETTINGS

Description	Value
Discretization of fluids	P1 + P1

SETTINGS

Description	Value
Equation form	Study controlled

Physical Model

SETTINGS

Description	Value	Unit
Neglect inertial term (Stokes flow)	On	
Compressibility	Incompressible flow	
Enable porous media domains	Off	
Include gravity	Off	
Reference temperature	User defined	
Reference temperature	293.15	K
Reference pressure level	1.0133E5	Pa

Turbulence

SETTINGS

Description	Value
Turbulence model type	None

2.3.2 Variables

Name	Expression	Unit	Description	Selection	Details
spf.Tref	model.input.Tref	K	Reference temperature	Global	Meta
spf.dz	1	m	Thickness	Domains 1–3	
spf.pref	1[atm]	Pa	Reference pressure level	Domains 1–3	
spf.pA	p+spf.pref	Pa	Absolute pressure	Domains 1–3	
spf.hasWF	0		Help variable	Boundaries 1–4, 6–7, 9–16	
spf.hasWF_u	0		Help variable	Boundaries 5,	

Name	Expression	Unit	Description	Selection	Details
				17	
spf.hasWF_d	0		Help variable	Boundaries 5, 17	
spf.dt_CFL	1/max(spf.maxop(sqrt(emetric_spatial(u-d(r,TIME),w-d(z,TIME)))),eps)	s	Time step, CFL=1	Global	
spf.CFL_number	timestep/spf.dt_CFL	1	CFL number	Global	
spf.Qvd_tot	spf.intop(2*spf.Qvd*pi*r)	W	Total viscous dissipation	Global	
spf.K_stressr	spf.K_stress_tensorrr*spf.nrmesh+spf.K_stress_tensorrphi*spf.nphimesh+spf.K_stress_tensorrz*spf.nzmesh	N/m²	Viscous force, exterior boundaries, r-component	Boundaries 1–4, 6–7, 9–16	
spf.K_stressphi	spf.K_stress_tensorphir*spf.nrmesh+spf.K_stress_tensorphiphi*spf.nphimesh+spf.K_stress_tensorphiz*spf.nzmesh	N/m²	Viscous force, exterior boundaries, phi-component	Boundaries 1–4, 6–7, 9–16	
spf.K_stressz	spf.K_stress_tensorzr*spf.nrmesh+spf.K_stress_tensorzphi*spf.nphimesh+spf.K_stress_tensorzz*spf.nzmesh	N/m²	Viscous force, exterior boundaries, z-component	Boundaries 1–4, 6–7, 9–16	
spf.T_stressr	spf.T_stress_tensorrr*spf.nrmesh+spf.T_stress_tensorrphi*spf.nphimesh+spf.T_stress_tensorrz*spf.nzmesh	N/m²	Total traction, exterior boundaries, r-component	Boundaries 1–4, 6–7, 9–16	
spf.T_stressphi	spf.T_stress_tensorphir*spf.nrmesh+spf.T_stress_tensorphiphi*spf.nphimesh+spf.T_stress_tensorphiz*spf.nzmesh	N/m²	Total traction, exterior boundaries, phi-component	Boundaries 1–4, 6–7, 9–16	
spf.T_stressz	spf.T_stress_tensorzr*spf.nrmesh+spf.T_stress_tensorzphi*spf.nphimesh+spf.T_stress_tensorzz*spf.nzmesh	N/m²	Total traction, exterior boundaries, z-component	Boundaries 1–4, 6–7, 9–16	
spf.K_stress_dr	down(spf.K_stress_tensorrr)*spf.nrmesh+do	N/m²	Viscous force, interior	Boundaries 5, 17	

Name	Expression	Unit	Description	Selection	Details
	wn(spf.K_stress_tensorrphi)*spf.nphimesh+down(spf.K_stress_tensorrz)*spf.nzmesh		boundaries, downside, r-component		
spf.K_stress_dphi	down(spf.K_stress_tensorphir)*spf.nrmesh+down(spf.K_stress_tensorphiphi)*spf.nphimesh+down(spf.K_stress_tensorphiz)*spf.nzmesh	N/m²	Viscous force, interior boundaries, downside, phi-component	Boundaries 5, 17	
spf.K_stress_dz	down(spf.K_stress_tensorzr)*spf.nrmesh+down(spf.K_stress_tensorzphi)*spf.nphimesh+down(spf.K_stress_tensorzz)*spf.nzmesh	N/m²	Viscous force, interior boundaries, downside, z-component	Boundaries 5, 17	
spf.K_stress_dr	down(spf.K_stress_tensorrr)*spf.dnrmesh+down(spf.K_stress_tensorrphi)*spf.dnphimesh+down(spf.K_stress_tensorrz)*spf.dnzmesh	N/m²	Viscous force, interior boundaries, downside, r-component	Boundaries 1–4, 6–7, 9–16	
spf.K_stress_dphi	down(spf.K_stress_tensorphir)*spf.dnrmesh+down(spf.K_stress_tensorphiphi)*spf.dnphimesh+down(spf.K_stress_tensorphiz)*spf.dnzmesh	N/m²	Viscous force, interior boundaries, downside, phi-component	Boundaries 1–4, 6–7, 9–16	
spf.K_stress_dz	down(spf.K_stress_tensorzr)*spf.dnrmesh+down(spf.K_stress_tensorzphi)*spf.dnphimesh+down(spf.K_stress_tensorzz)*spf.dnzmesh	N/m²	Viscous force, interior boundaries, downside, z-component	Boundaries 1–4, 6–7, 9–16	
spf.K_stress_ur	-up(spf.K_stress_tensorrr)*spf.nrmesh-up(spf.K_stress_tensorrphi)*spf.nphimesh-up(spf.K_stress_tensorrz)*spf.nzmesh	N/m²	Viscous force, interior boundaries, upside, r-component	Boundaries 5, 17	
spf.K_stress_uphi	-up(spf.K_stress_tensorphir)*spf.nrmesh-	N/m²	Viscous force, interior boundaries,	Boundaries 5, 17	

Name	Expression	Unit	Description	Selection	Details
	up(spf.K_stress_tensorphiphi)*spf.nphimesh-up(spf.K_stress_tensorphiz)*spf.nzmesh		upside, phi-component		
spf.K_stress_uz	-up(spf.K_stress_tensorzr)*spf.nrmesh-up(spf.K_stress_tensorzphi)*spf.nphimesh-up(spf.K_stress_tensorzz)*spf.nzmesh	N/m^2	Viscous force, interior boundaries, upside, z-component	Boundaries 5, 17	
spf.T_stress_dr	down(spf.T_stress_tensorrr)*spf.nrmesh+down(spf.T_stress_tensorrphi)*spf.nphimesh+down(spf.T_stress_tensorrz)*spf.nzmesh	N/m^2	Total traction, interior boundaries, downside, r-component	Boundaries 5, 17	
spf.T_stress_dphi	down(spf.T_stress_tensorphir)*spf.nrmesh+down(spf.T_stress_tensorphiphi)*spf.nphimesh+down(spf.T_stress_tensorphiz)*spf.nzmesh	N/m^2	Total traction, interior boundaries, downside, phi-component	Boundaries 5, 17	
spf.T_stress_dz	down(spf.T_stress_tensorzr)*spf.nrmesh+down(spf.T_stress_tensorzphi)*spf.nphimesh+down(spf.T_stress_tensorzz)*spf.nzmesh	N/m^2	Total traction, interior boundaries, downside, z-component	Boundaries 5, 17	
spf.T_stress_dr	down(spf.T_stress_tensorrr)*spf.dnrmesh+down(spf.T_stress_tensorrphi)*spf.dnphimesh+down(spf.T_stress_tensorrz)*spf.dnzmesh	N/m^2	Total traction, interior boundaries, downside, r-component	Boundaries 1–4, 6–7, 9–16	
spf.T_stress_dphi	down(spf.T_stress_tensorphir)*spf.dnrmesh+down(spf.T_stress_tensorphiphi)*spf.dnphimesh+down(spf.T_stress_tensorphiz)*spf.dnzmesh	N/m^2	Total traction, interior boundaries, downside, phi-component	Boundaries 1–4, 6–7, 9–16	
spf.T_stress_dz	down(spf.T_stress_tensorzr)*spf.dnrmesh+down(spf.T_stress_tens	N/m^2	Total traction, interior boundaries,	Boundaries 1–4, 6–7, 9–16	

Name	Expression	Unit	Description	Selection	Details
	orzphi)*spf.dnphimesh+down(spf.T_stress_tensorzz)*spf.dnzmesh		downside, z-component		
spf.T_stress_ur	-up(spf.T_stress_tensorrr)*spf.nrmesh-up(spf.T_stress_tensorrphi)*spf.nphimesh-up(spf.T_stress_tensorrz)*spf.nzmesh	N/m²	Total traction, interior boundaries, upside, r-component	Boundaries 5, 17	
spf.T_stress_uphi	-up(spf.T_stress_tensorphir)*spf.nrmesh-up(spf.T_stress_tensorphiphi)*spf.nphimesh-up(spf.T_stress_tensorphiz)*spf.nzmesh	N/m²	Total traction, interior boundaries, upside, phi-component	Boundaries 5, 17	
spf.T_stress_uz	-up(spf.T_stress_tensorzr)*spf.nrmesh-up(spf.T_stress_tensorzphi)*spf.nphimesh-up(spf.T_stress_tensorzz)*spf.nzmesh	N/m²	Total traction, interior boundaries, upside, z-component	Boundaries 5, 17	
spf.usePseudoTimeStepping	isrunningpseudotimestepping	1	Help variable	Global	
spf.localCFLvalue	1.3^min(niterCMP,9)+if(niterCMP>=25,9*1.3^min(-25+niterCMP,9),0)+if(niterCMP>=45,90*1.3^min(-45+niterCMP,9),0)		Local CFL number	Domains 1–3	
spf.locCFL	max(CFLCMP,sqrt(eps))	1	Local CFL number	Global	
spf.geometryLengthScale	7.625E-6	m	Geometry length scale	Domains 1–3	
spf.time_step_inv	max(sqrt(emetric_spatial(u,w)*2^gmg_level^2),spf.nu/spf.geometryLengthScale^2)	Hz	Inverse time step	Domains 1–3	
spf.tsti	nojac(spf.time_step_inv/spf.locCFL)	1/s	Help variable	Domains 1–3	
spf.nr	nr	1	Normal vector,	Boundaries 5,	

Name	Expression	Unit	Description	Selection	Details
			r-component	17	
spf.nphi	0	1	Normal vector, phi-component	Boundaries 5, 17	
spf.nz	nz	1	Normal vector, z-component	Boundaries 5, 17	
spf.nr	dnr	1	Normal vector, r-component	Boundaries 1–4, 6–7, 9–16	
spf.nphi	0	1	Normal vector, phi-component	Boundaries 1–4, 6–7, 9–16	
spf.nz	dnz	1	Normal vector, z-component	Boundaries 1–4, 6–7, 9–16	
spf.nrmesh	nrmesh	1	Normal vector, r-component	Boundaries 5, 17	
spf.nphimesh	0	1	Normal vector, phi-component	Boundaries 5, 17	
spf.nzmesh	nzmesh	1	Normal vector, z-component	Boundaries 5, 17	
spf.nrmesh	dnrmesh	1	Normal vector, r-component	Boundaries 1–4, 6–7, 9–16	
spf.nphimesh	0	1	Normal vector, phi-component	Boundaries 1–4, 6–7, 9–16	
spf.nzmesh	dnzmesh	1	Normal vector, z-component	Boundaries 1–4, 6–7, 9–16	

2.3.3 Fluid Properties 1

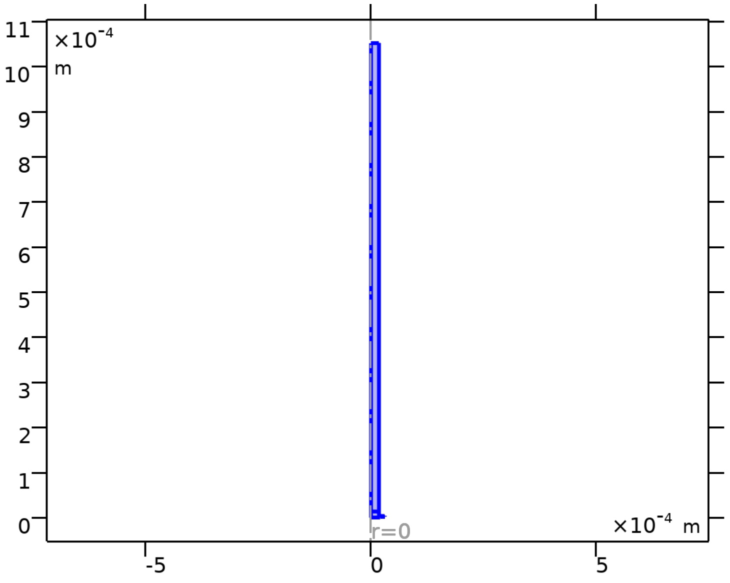

Fluid Properties 1

SELECTION

Geometric entity level	Domain
Selection	Geometry geom1: Dimension 2: All domains

EQUATIONS

$$\rho\frac{\partial \mathbf{u}}{\partial t} = \nabla\cdot[-p\mathbf{I} + \mathbf{K}] + \mathbf{F}$$

$$\rho\nabla\cdot\mathbf{u} = 0$$

$$\mathbf{K} = \mu\left(\nabla\mathbf{u} + (\nabla\mathbf{u})^T\right)$$

Fluid Properties

SETTINGS

Description	Value	Unit
Density	User defined	
Density	997.8	kg/m³
	Specify dynamic viscosity	
Dynamic viscosity	User defined	
Dynamic viscosity	9.544E-4	Pa·s

Variables

Name	Expression	Unit	Description	Selection	Details
spf.mu	material.mu	Pa·s	Dynamic	Domains 1–3	Meta

Name	Expression	Unit	Description	Selection	Details
			viscosity		
spf.rho	material.rho	kg/m^3	Density	Domains 1–3	Meta
spf.Trho	spf.fp1.minput_temperature	K	Temperature for density evaluation	Domains 1–3	
spf.prho	spf.fp1.minput_pressure	Pa	Pressure for the evaluation of density	Domains 1–3	
spf.rhoref	subst(material.rho,minput.T,spf.Tref,minput.pA,spf.pref)	kg/m^3	Reference density	Domains 1–3	Meta
spf.mumat	material.mu	Pa·s	Dynamic viscosity	Domains 1–3	Meta
spf.srijrr	ur	1/s	Strain rate tensor, rr-component	Domains 1–3	
spf.srijphir	0	1/s	Strain rate tensor, phir-component	Domains 1–3	
spf.srijzr	0.5*(wr+uz)	1/s	Strain rate tensor, zr-component	Domains 1–3	
spf.srijrphi	0	1/s	Strain rate tensor, rphi-component	Domains 1–3	
spf.srijphiphi	if(abs(r)<0.001*h_spatial,ur,u/r)	1/s	Strain rate tensor, phiphi-component	Domains 1–3	
spf.srijzphi	0	1/s	Strain rate tensor, zphi-component	Domains 1–3	
spf.srijrz	0.5*(uz+wr)	1/s	Strain rate tensor, rz-component	Domains 1–3	
spf.srijphiz	0	1/s	Strain rate tensor, phiz-component	Domains 1–3	
spf.srijzz	wz	1/s	Strain rate tensor, zz-component	Domains 1–3	
spf.rrijrr	0	1/s	Rotation rate	Domains 1–3	

Name	Expression	Unit	Description	Selection	Details
			tensor, rr-component		
spf.rrijphir	0	1/s	Rotation rate tensor, phir-component	Domains 1–3	
spf.rrijzr	0.5*(wr-uz)	1/s	Rotation rate tensor, zr-component	Domains 1–3	
spf.rrijrphi	0	1/s	Rotation rate tensor, rphi-component	Domains 1–3	
spf.rrijphiphi	0	1/s	Rotation rate tensor, phiphi-component	Domains 1–3	
spf.rrijzphi	0	1/s	Rotation rate tensor, zphi-component	Domains 1–3	
spf.rrijrz	0.5*(uz-wr)	1/s	Rotation rate tensor, rz-component	Domains 1–3	
spf.rrijphiz	0	1/s	Rotation rate tensor, phiz-component	Domains 1–3	
spf.rrijzz	0	1/s	Rotation rate tensor, zz-component	Domains 1–3	
spf.sr	sqrt(2*spf.srijrr^2+2*spf.srijrphi^2+2*spf.srijrz^2+2*spf.srijphir^2+2*spf.srijphiphi^2+2*spf.srijphiz^2+2*spf.srijzr^2+2*spf.srijzphi^2+2*spf.srijzz^2+eps)	1/s	Shear rate	Domains 1–3	
spf.rr	sqrt(2*spf.rrijrr^2+2*spf.rrijrphi^2+2*spf.rrijrz^2+2*spf.rrijphir^2+2*spf.rrijphiphi^2+2*spf.rrijphiz^2+2*spf.rrijzr^2+2*spf.rrijzphi^2+2*spf.rrijzz^2+eps)	1/s	Rotation rate	Domains 1–3	

Name	Expression	Unit	Description	Selection	Details
spf.divu	ur+if(abs(r)<0.001*h_spatial,ur,u/r)+wz	1/s	Divergence of velocity field	Domains 1–3	
spf.Fr	0	N/m^3	Volume force, r-component	Domains 1–3	+ operation
spf.Fphi	0	N/m^3	Volume force, phi-component	Domains 1–3	+ operation
spf.Fz	0	N/m^3	Volume force, z-component	Domains 1–3	+ operation
spf.U	sqrt(u^2+w^2)	m/s	Velocity magnitude	Domains 1–3	
spf.vorticityr	0	1/s	Vorticity field, r-component	Domains 1–3	
spf.vorticityphi	-wr+uz	1/s	Vorticity field, phi-component	Domains 1–3	
spf.vorticityz	0	1/s	Vorticity field, z-component	Domains 1–3	
spf.vort_magn	sqrt(spf.vorticityr^2+spf.vorticityphi^2+spf.vorticityz^2)	1/s	Vorticity magnitude	Domains 1–3	
spf.cellRe	0.25*spf.rho*sqrt(emetric_spatial(u-d(r,TIME),w-d(z,TIME))/emetric2_spatial)/spf.mu	1	Cell Reynolds number	Domains 1–3	
spf.nu	spf.mu/spf.rho	m^2/s	Kinematic viscosity	Domains 1–3	
spf.betaT	0	1/Pa	Isothermal compressibility coefficient	Domains 1–3	
spf.Qm	0	$kg/(m^3 \cdot s)$	Source term	Domains 1–3	+ operation
spf.Fgtotr	0	N/m^3	Gravity force, r-component	Domains 1–3	+ operation
spf.Fgtotphi	0	N/m^3	Gravity force, phi-component	Domains 1–3	+ operation
spf.Fgtotz	0	N/m^3	Gravity force, z-component	Domains 1–3	+ operation
spf.Qm_aco	0	$kg/(m^3 \cdot s)$	Acoustic mass source	Domains 1–3	

Name	Expression	Unit	Description	Selection	Details
spf.F_acor	0	N/m³	Acoustic volume force, r-component	Domains 1–3	
spf.F_acophi	0	N/m³	Acoustic volume force, phi-component	Domains 1–3	
spf.F_acoz	0	N/m³	Acoustic volume force, z-component	Domains 1–3	
spf.gamma_sr	sqrt(2*spf.srijrr^2+2*spf.srijrphi^2+2*spf.srijrz^2+2*spf.srijphir^2+2*spf.srijphiphi^2+2*spf.srijphiz^2+2*spf.srijzr^2+2*spf.srijzphi^2+2*spf.srijzz^2+eps)	1/s	Shear rate	Domains 1–3	
spf.mu_eff	spf.mu+spf.muT	Pa·s	Effective dynamic viscosity	Domains 1–3	
spf.muT	0	Pa·s	Turbulent dynamic viscosity	Domains 1–3	+ operation
spf.T_stress_tensorrr	spf.K_stress_tensorrr-p	N/m²	Total stress tensor, rr-component	Domains 1–3	+ operation
spf.T_stress_tensorphir	spf.K_stress_tensorphir	N/m²	Total stress tensor, phir-component	Domains 1–3	+ operation
spf.T_stress_tensorzr	spf.K_stress_tensorzr	N/m²	Total stress tensor, zr-component	Domains 1–3	+ operation
spf.T_stress_tensorrphi	spf.K_stress_tensorrphi	N/m²	Total stress tensor, rphi-component	Domains 1–3	+ operation
spf.T_stress_tensorphiphi	spf.K_stress_tensorphiphi-p	N/m²	Total stress tensor, phiphi-component	Domains 1–3	+ operation
spf.T_stress_tensorzphi	spf.K_stress_tensorzphi	N/m²	Total stress tensor, zphi-component	Domains 1–3	+ operation
spf.T_stress_tens	spf.K_stress_tenso	N/m²	Total stress	Domains 1–3	+ operation

Name	Expression	Unit	Description	Selection	Details
orrz	rrz		tensor, rz-component		
spf.T_stress_tensorphiz	spf.K_stress_tensorphiz	N/m²	Total stress tensor, phiz-component	Domains 1–3	+ operation
spf.T_stress_tensorzz	spf.K_stress_tensorzz-p	N/m²	Total stress tensor, zz-component	Domains 1–3	+ operation
spf.K_stress_tensorrr	2*spf.mu_eff*ur	N/m²	Viscous stress tensor, rr-component	Domains 1–3	+ operation
spf.K_stress_tensorphir	0	N/m²	Viscous stress tensor, phir-component	Domains 1–3	+ operation
spf.K_stress_tensorzr	spf.mu_eff*(wr+uz)	N/m²	Viscous stress tensor, zr-component	Domains 1–3	+ operation
spf.K_stress_tensorrphi	0	N/m²	Viscous stress tensor, rphi-component	Domains 1–3	+ operation
spf.K_stress_tensorphiphi	2*spf.mu_eff*if(abs(r)<0.001*h_spatial,ur,u/r)	N/m²	Viscous stress tensor, phiphi-component	Domains 1–3	+ operation
spf.K_stress_tensorzphi	0	N/m²	Viscous stress tensor, zphi-component	Domains 1–3	+ operation
spf.K_stress_tensorrz	spf.mu_eff*(uz+wr)	N/m²	Viscous stress tensor, rz-component	Domains 1–3	+ operation
spf.K_stress_tensorphiz	0	N/m²	Viscous stress tensor, phiz-component	Domains 1–3	+ operation
spf.K_stress_tensorzz	2*spf.mu_eff*wz	N/m²	Viscous stress tensor, zz-component	Domains 1–3	+ operation
spf.K_stress_tensor_testrr	2*spf.mu_eff*test(ur)	N/m²	Viscous stress tensor test, rr-component	Domains 1–3	+ operation
spf.K_stress_tensor_testphir	0	N/m²	Viscous stress tensor test, phir-component	Domains 1–3	+ operation
spf.K_stress_tensor_testzr	spf.mu_eff*(test(wr)+test(uz))	N/m²	Viscous stress tensor test, zr-	Domains 1–3	+ operation

Name	Expression	Unit	Description	Selection	Details
			component		
spf.K_stress_tensor_testrphi	0	N/m²	Viscous stress tensor test, rphi-component	Domains 1–3	+ operation
spf.K_stress_tensor_testphiphi	2*spf.mu_eff*if(abs(r)<0.001*h_spatial,test(ur),test(u)/r)	N/m²	Viscous stress tensor test, phiphi-component	Domains 1–3	+ operation
spf.K_stress_tensor_testzphi	0	N/m²	Viscous stress tensor test, zphi-component	Domains 1–3	+ operation
spf.K_stress_tensor_testrz	spf.mu_eff*(test(uz)+test(wr))	N/m²	Viscous stress tensor test, rz-component	Domains 1–3	+ operation
spf.K_stress_tensor_testphiz	0	N/m²	Viscous stress tensor test, phiz-component	Domains 1–3	+ operation
spf.K_stress_tensor_testzz	2*spf.mu_eff*test(wz)	N/m²	Viscous stress tensor test, zz-component	Domains 1–3	+ operation
spf.upwind_helpr	-d(r,TIME)	m/s	Upwind term, r-component	Domains 1–3	+ operation
spf.upwind_helpphi	0	m/s	Upwind term, phi-component	Domains 1–3	+ operation
spf.upwind_helpz	-d(z,TIME)	m/s	Upwind term, z-component	Domains 1–3	+ operation
spf.continuityEquation	spf.rho*spf.divu	kg/(m³·s)	Continuity equation	Domains 1–3	
spf.contCoeff	spf.rho	kg/m³	Help variable	Domains 1–3	
spf.tau_vdrr	2*spf.mu*spf.srijrr	Pa	Viscous stress tensor, rr-component	Domains 1–3	+ operation
spf.tau_vdphir	2*spf.mu*spf.srijphir	Pa	Viscous stress tensor, phir-component	Domains 1–3	+ operation
spf.tau_vdzr	2*spf.mu*spf.srijzr	Pa	Viscous stress tensor, zr-component	Domains 1–3	+ operation
spf.tau_vdrphi	2*spf.mu*spf.srijrphi	Pa	Viscous stress tensor, rphi-component	Domains 1–3	+ operation
spf.tau_vdphiphi	2*spf.mu*spf.srijp	Pa	Viscous stress	Domains 1–3	+ operation

Name	Expression	Unit	Description	Selection	Details
	hiphi		tensor, phiphi-component		
spf.tau_vdzphi	2*spf.mu*spf.srijzphi	Pa	Viscous stress tensor, zphi-component	Domains 1–3	+ operation
spf.tau_vdrz	2*spf.mu*spf.srijrz	Pa	Viscous stress tensor, rz-component	Domains 1–3	+ operation
spf.tau_vdphiz	2*spf.mu*spf.srijphiz	Pa	Viscous stress tensor, phiz-component	Domains 1–3	+ operation
spf.tau_vdzz	2*spf.mu*spf.srijzz	Pa	Viscous stress tensor, zz-component	Domains 1–3	+ operation
spf.Qvd	spf.tau_vdrr*ur+spf.tau_vdrz*uz+spf.tau_vdphiphi*if(abs(r)<0.001*h_spatial,ur,u/r)+spf.tau_vdzr*wr+spf.tau_vdzz*wz	W/m³	Viscous dissipation	Domains 1–3	+ operation
spf.epsilon_p	1	1	Porosity	Domains 1–3	
spf.epsilon_p_pos	1	1	Positive porosity	Domains 1–3	
spf.Fst_tensorrr	0	N/m²	Surface tension force, rr-component	Domains 1–3	+ operation
spf.Fst_tensorphir	0	N/m²	Surface tension force, phir-component	Domains 1–3	+ operation
spf.Fst_tensorzr	0	N/m²	Surface tension force, zr-component	Domains 1–3	+ operation
spf.Fst_tensorrphi	0	N/m²	Surface tension force, rphi-component	Domains 1–3	+ operation
spf.Fst_tensorphiphi	0	N/m²	Surface tension force, phiphi-component	Domains 1–3	+ operation
spf.Fst_tensorzphi	0	N/m²	Surface tension force, zphi-component	Domains 1–3	+ operation
spf.Fst_tensorrz	0	N/m²	Surface tension force, rz-	Domains 1–3	+ operation

Name	Expression	Unit	Description	Selection	Details
			component		
spf.Fst_tensorphiz	0	N/m²	Surface tension force, phiz-component	Domains 1–3	+ operation
spf.Fst_tensorzz	0	N/m²	Surface tension force, zz-component	Domains 1–3	+ operation
spf.res_u	spf.rho*ut+pr-(d(2*ur,r)+if(abs(r)<0.001*h_spatial,d(2*ur,r),2*ur/r)+d(uz+wr,z)-2*if(abs(r)<0.001*h_spatial,ur,u/r)/r)*spf.mu-spf.Fr	N/m³	Equation residual	Domains 1–3	
spf.res_v	-spf.Fphi	N/m³	Equation residual	Domains 1–3	
spf.res_w	spf.rho*wt+pz-(d(wr+uz,r)+if(abs(r)<0.001*h_spatial,d(wr+uz,r),(wr+uz)/r)+d(2*wz,z))*spf.mu-spf.Fz	N/m³	Equation residual	Domains 1–3	
spf.res_p	spf.rho*spf.divu	kg/(m³·s)	Pressure equation residual	Domains 1–3	

Shape functions

Name	Shape function	Unit	Description	Shape frame	Selection
u	Lagrange (Linear)	m/s	Velocity field, r-component	Spatial	Domains 1–3
w	Lagrange (Linear)	m/s	Velocity field, z-component	Spatial	Domains 1–3
u	Lagrange (Linear)	m/s	Velocity field, r-component	Spatial	Domains 1–3
w	Lagrange (Linear)	m/s	Velocity field, z-component	Spatial	Domains 1–3
p	Lagrange (Linear)	Pa	Pressure	Spatial	Domains 1–3

Weak Expressions

Weak expression	Integration order	Integration frame	Selection
2*spf.rho*(-ut*test(u)-	2	Spatial	Domains 1–3

Weak expression	Integration order	Integration frame	Selection
wt*test(w))*pi*r			
2*((p-spf.K_stress_tensorrr)*test(ur)-spf.K_stress_tensorrz*test(uz)+(p-spf.K_stress_tensorphiphi)*if(abs(r)<0.001*h_spatial,test(ur),test(u)/r)-spf.K_stress_tensorzr*test(wr)+(p-spf.K_stress_tensorzz)*test(wz))*pi*r	2	Spatial	Domains 1–3
2*(spf.Fr*test(u)+spf.Fz*test(w))*pi*r	2	Spatial	Domains 1–3
-2*spf.continuityEquation*test(p)*pi*r	2	Spatial	Domains 1–3
2*spf.streamlinens*pi*r	2	Spatial	Domains 1–3

2.3.4 Initial Values 1

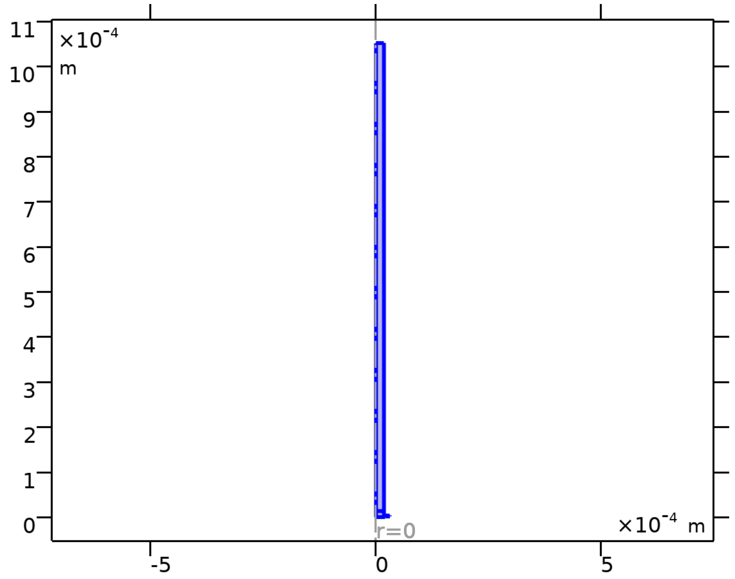

Initial Values 1

SELECTION

Geometric entity level	Domain
Selection	Geometry geom1: Dimension 2: All domains

Initial Values

SETTINGS

Description	Value	Unit
Velocity field, r-component	0	m/s
Velocity field, phi-component	0	m/s

Description	Value	Unit
Velocity field, z-component	0	m/s
Pressure	0	Pa

Coordinate System Selection

SETTINGS

Description	Value
Coordinate system	Global coordinate system

Variables

Name	Expression	Unit	Description	Selection
spf.u_initr	0	m/s	Velocity field, r-component	Domains 1–3
spf.u_initphi	0	m/s	Velocity field, phi-component	Domains 1–3
spf.u_initz	0	m/s	Velocity field, z-component	Domains 1–3
spf.p_init	0	Pa	Pressure	Domains 1–3

2.3.5 Axial Symmetry 1

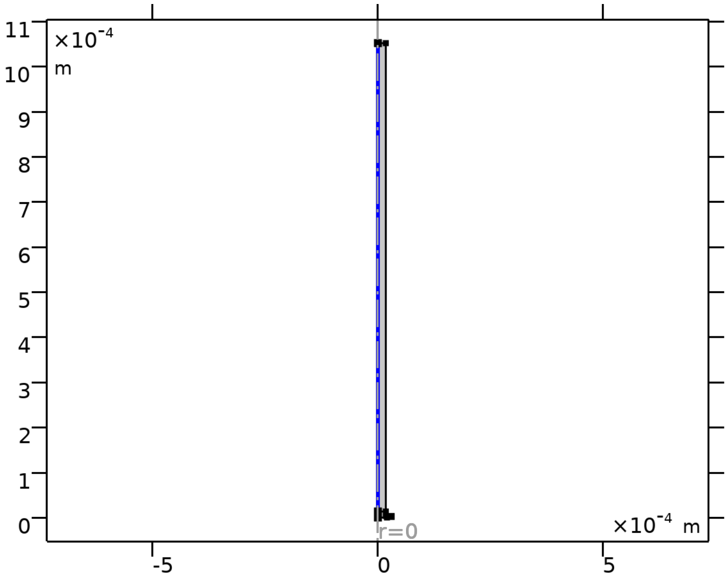

Axial Symmetry 1

SELECTION

Geometric entity level	Boundary
Selection	Geometry geom1: Dimension 1: All boundaries

Constraints

Constraint	Constraint force	Shape function	Selection	Details

Constraint	Constraint force	Shape function	Selection	Details
-u	test(-u)	Lagrange (Linear)	Boundaries 1, 3–4	Elemental

2.3.6 Wall 1

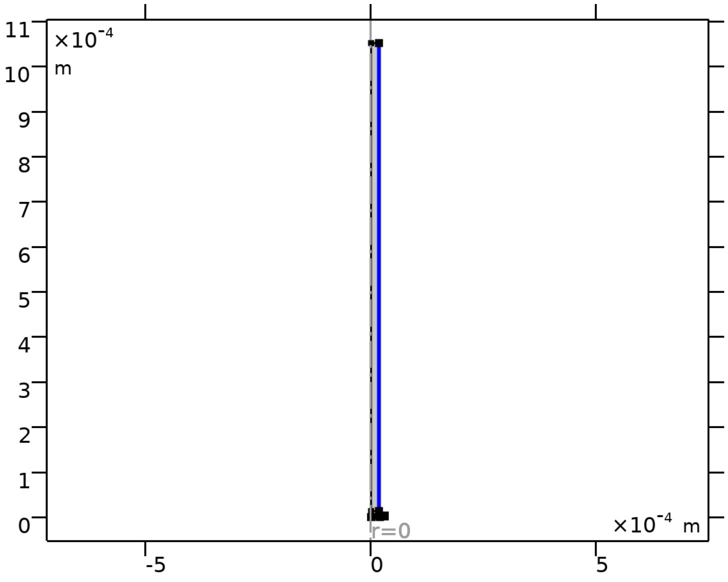

Wall 1

SELECTION

Geometric entity level	Boundary
Selection	Geometry geom1: Dimension 1: All boundaries

EQUATIONS

$$\mathbf{u} = \mathbf{0}$$

Boundary Condition

SETTINGS

Description	Value
Wall condition	No slip

Wall Movement

SETTINGS

Description	Value
Translational velocity	Automatic from frame
Sliding wall	Off

Variables

Name	Expression	Unit	Description	Selection	Details

Name	Expression	Unit	Description	Selection	Details
spf.ubndr	spf.utrr+spf.usr	m/s	Velocity at boundary, r-component	Boundaries 2, 7, 9–15	
spf.ubndphi	spf.utrphi+spf.usphi	m/s	Velocity at boundary, phi-component	Boundaries 2, 7, 9–15	
spf.ubndz	spf.utrz+spf.usz	m/s	Velocity at boundary, z-component	Boundaries 2, 7, 9–15	
spf.usr	0	m/s	Velocity of sliding wall, r-component	Boundaries 2, 7, 9–15	
spf.usphi	0	m/s	Velocity of sliding wall, phi-component	Boundaries 2, 7, 9–15	
spf.usz	0	m/s	Velocity of sliding wall, z-component	Boundaries 2, 7, 9–15	
spf.utrr	0	m/s	Velocity of moving wall, r-component	Boundaries 2, 7, 9–15	
spf.utrphi	0	m/s	Velocity of moving wall, phi-component	Boundaries 2, 7, 9–15	
spf.utrz	0	m/s	Velocity of moving wall, z-component	Boundaries 2, 7, 9–15	
spf.uLeakager	0	m/s	Leakage velocity, r-component	Boundaries 2, 7, 9–15	+ operation
spf.uLeakagephi	0	m/s	Leakage velocity, phi-component	Boundaries 2, 7, 9–15	+ operation
spf.uLeakagez	0	m/s	Leakage velocity, z-component	Boundaries 2, 7, 9–15	+ operation
spf.noSlipWall	1	1	Help variable	Boundaries 2, 7, 9–15	

Constraints

Constraint	Constraint force	Shape function	Selection	Details
-u+spf.ubndr+spf.uLeakager	test(-u)	Lagrange (Linear)	Boundaries 2, 7, 9–15	Elemental
spf.ubndphi+spf.uLeakagephi	0		Boundaries 2, 7, 9–15	Elemental
-w+spf.ubndz+spf.uLeakagez	test(-w)	Lagrange (Linear)	Boundaries 2, 7, 9–15	Elemental

2.3.7 Open Boundary 1

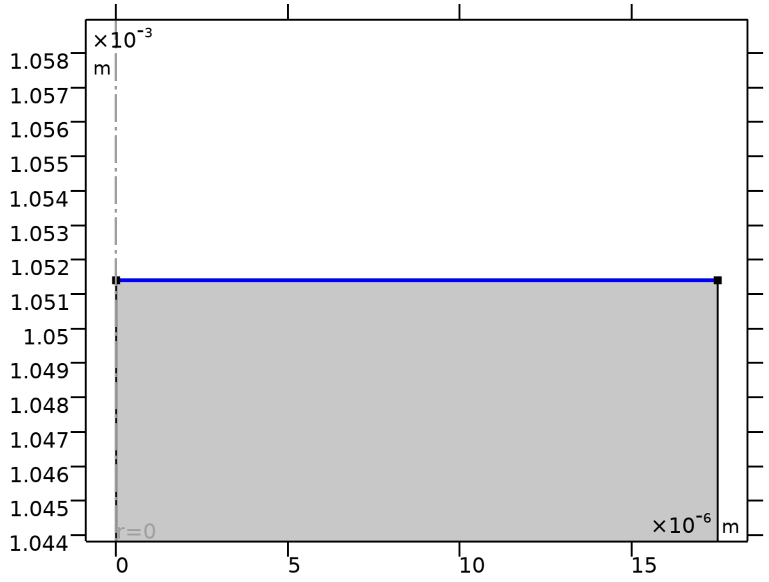

Open Boundary 1

SELECTION

Geometric entity level	Boundary
Selection	Geometry geom1: Dimension 1: Boundary 6

EQUATIONS

$$[-p\mathbf{I} + \mathbf{K}]\mathbf{n} = -f_0\mathbf{n}$$

Boundary Condition

SETTINGS

Description	Value	Unit
Boundary condition	Normal stress	
Normal stress	0	N/m²

Variables

Name	Expression	Unit	Description	Selection
spf.f0	0	N/m^2	Normal stress	Boundary 6
spf.open1.volumeFlowRate	spf.open1.intop(2*(u*spf.nrmesh+w*spf.nzmesh)*pi*r)	m^3/s	Outward volume flow rate across feature selection	Global
spf.open1.massFlowRate	spf.open1.intop(2*spf.rho*(u*spf.nrmesh+w*spf.nzmesh)*pi*r)	kg/s	Outward mass flow rate across feature selection	Global
spf.open1.pAverage	spf.open1.intop(2*p*pi*r)/max(spf.open1.intop(2*pi*r),1000*eps)	Pa	Pressure average over feature selection	Global

Weak Expressions

Weak expression	Integration order	Integration frame	Selection
-2*spf.f0*(test(u)*spf.nrmesh+test(w)*spf.nzmesh)*pi*r	2	Spatial	Boundary 6

2.3.8 Outlet 1

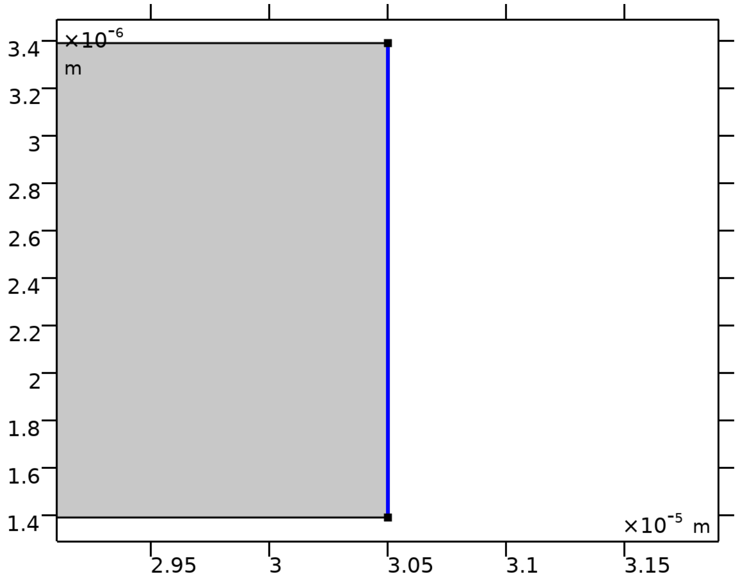

Outlet 1

SELECTION

Geometric entity level	Boundary

Selection	Geometry geom1: Dimension 1: Boundary 16

EQUATIONS

$$\mathbf{u} = U_0\mathbf{n}$$

Boundary Condition

SETTINGS

Description	Value
Boundary condition	Velocity

Velocity

SETTINGS

Description	Value	Unit
Velocity field componentwise	Normal outflow velocity	
Normal outflow velocity	vdry	m/s

Constraint Settings

SETTINGS

Description	Value
Apply reaction terms on	All physics (symmetric)
Use weak constraints	Off
Constraint method	Elemental

Variables

Name	Expression	Unit	Description	Selection
spf.ubndr	spf.nr*spf.U0out	m/s	Velocity at boundary, r-component	Boundary 16
spf.ubndphi	spf.nphi*spf.U0out	m/s	Velocity at boundary, phi-component	Boundary 16
spf.ubndz	spf.nz*spf.U0out	m/s	Velocity at boundary, z-component	Boundary 16
spf.U0out	vdry	m/s	Normal outflow velocity	Boundary 16
spf.out1.Uav	0	m/s	Average velocity	Global
spf.out1.Uavfdf	0	m/s	Average velocity	Global

Name	Expression	Unit	Description	Selection
spf.out1.dz	spf.dz	m	Channel thickness	Boundary 16
spf.out1.Mflow	spf.out1.massFlowRate	kg/s	Mass flow	Global
spf.out1.volumeFlowRat e	spf.out1.intop(2*(u*spf.nrmesh +w*spf.nzmesh)*pi*r)	m^3/s	Outward volume flow rate across feature selection	Global
spf.out1.massFlowRate	spf.out1.intop(2*spf.rho*(u*spf. nrmesh+w*spf.nzmesh)*pi*r)	kg/s	Outward mass flow rate across feature selection	Global
spf.out1.pAverage	spf.out1.intop(2*p*pi*r)/max(s pf.out1.intop(2*pi*r),1000*eps)	Pa	Pressure average over feature selection	Global

Constraints

Constraint	Constraint force	Shape function	Selection	Details
-u+spf.ubndr	test(-u+spf.ubndr)	Lagrange (Linear)	Boundary 16	Elemental
spf.ubndphi	test(spf.ubndphi)		Boundary 16	Elemental
-w+spf.ubndz	test(-w+spf.ubndz)	Lagrange (Linear)	Boundary 16	Elemental

2.4 ELECTROSTATICS

USED PRODUCTS

COMSOL Multiphysics

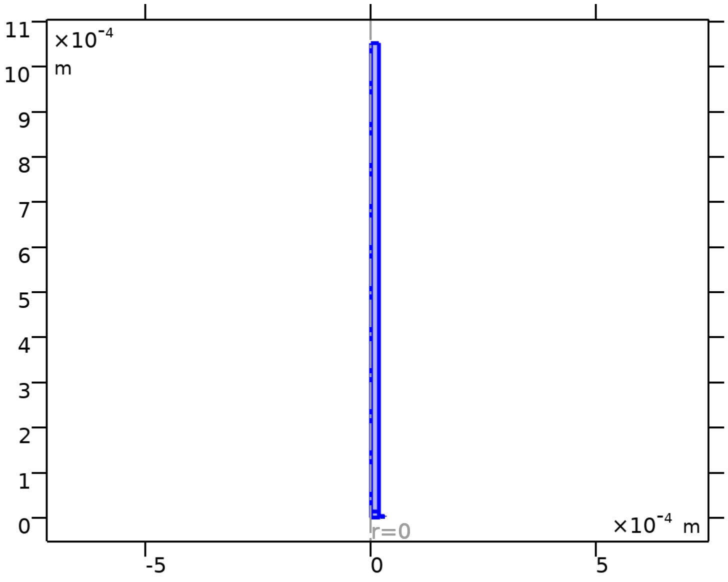

Electrostatics

SELECTION

Geometric entity level	Domain
Selection	Geometry geom1: Dimension 2: Domains 1–3

EQUATIONS

$$\nabla \cdot \mathbf{D} = \rho_v$$
$$\mathbf{E} = -\nabla V$$

2.4.1 Interface Settings

Discretization

SETTINGS

**Description**	**Value**
Electric potential	Quadratic

Manual Terminal Sweep Settings

SETTINGS

**Description**	**Value**	**Unit**
Use manual terminal sweep	Off	
Reference impedance	50	Ω

2.4.2 Variables

**Name**	**Expression**	**Unit**	**Description**	**Selection**	**Details**
es.d	1	1	Contribution	Domains 1–3	

Name	Expression	Unit	Description	Selection	Details
es.nr	nr		Normal vector, r-component	Boundaries 5, 17	
es.nphi	0		Normal vector, phi-component	Boundaries 5, 17	
es.nz	nz		Normal vector, z-component	Boundaries 5, 17	
es.nr	dnr		Normal vector, r-component	Boundaries 1–4, 6–7, 9–16	
es.nphi	0		Normal vector, phi-component	Boundaries 1–4, 6–7, 9–16	
es.nz	dnz		Normal vector, z-component	Boundaries 1–4, 6–7, 9–16	
es.nmeshr	nrmesh		Mesh normal vector, r-component	Boundaries 5, 17	
es.nmeshphi	0		Mesh normal vector, phi-component	Boundaries 5, 17	
es.nmeshz	nzmesh		Mesh normal vector, z-component	Boundaries 5, 17	
es.nmeshr	dnrmesh		Mesh normal vector, r-component	Boundaries 1–4, 6–7, 9–16	
es.nmeshphi	0		Mesh normal vector, phi-component	Boundaries 1–4, 6–7, 9–16	
es.nmeshz	dnzmesh		Mesh normal vector, z-component	Boundaries 1–4, 6–7, 9–16	
es.unmeshr	unrmesh		Mesh normal vector, upside, r-component	Boundaries 1–7, 9–17	
es.unmeshphi	0		Mesh normal vector, upside, phi-component	Boundaries 1–7, 9–17	
es.unmeshz	unzmesh		Mesh normal vector, upside, z-component	Boundaries 1–7, 9–17	
es.dnmeshr	dnrmesh		Mesh normal vector, downside, r-	Boundaries 1–7, 9–17	

Name	Expression	Unit	Description	Selection	Details
			component		
es.dnmeshphi	0		Mesh normal vector, downside, phi-component	Boundaries 1–7, 9–17	
es.dnmeshz	dnzmesh		Mesh normal vector, downside, z-component	Boundaries 1–7, 9–17	
es.I_sRR	(spatial.invF11*(spatial.invF11*es.I_srr+spatial.invF31*es.I_szr)+spatial.invF31*(spatial.invF11*es.I_srz+spatial.invF31*es.I_szz))*spatial.detF	1	Spatial identity matrix, material frame, RR-component	Domains 1–3	
es.I_sPHIR	if(Rg>0.001*h,R/r,Rr)*(spatial.invF11*es.I_sphir+spatial.invF31*es.I_sphiz)*spatial.detF	1	Spatial identity matrix, material frame, PHIR-component	Domains 1–3	
es.I_sZR	(spatial.invF11*(spatial.invF13*es.I_srr+spatial.invF33*es.I_szr)+spatial.invF31*(spatial.invF13*es.I_srz+spatial.invF33*es.I_szz))*spatial.detF	1	Spatial identity matrix, material frame, ZR-component	Domains 1–3	
es.I_sRPHI	if(Rg>0.001*h,R/r,Rr)*(spatial.invF11*es.I_srphi+spatial.invF31*es.I_szphi)*spatial.detF	1	Spatial identity matrix, material frame, RPHI-component	Domains 1–3	
es.I_sPHIPHI	if(Rg>0.001*h,R/r,Rr)^2*es.I_sphiphi*spatial.detF	1	Spatial identity matrix, material frame, PHIPHI-component	Domains 1–3	
es.I_sZPHI	if(Rg>0.001*h,R/r,Rr)*(spatial.invF13*es.I_srphi+spatial.invF33*es.I_szphi)*spatial.detF	1	Spatial identity matrix, material frame, ZPHI-component	Domains 1–3	
es.I_sRZ	(spatial.invF13*(spatial.invF11*es.I_srr+spatial.invF31*es.I_szr)+spatial.invF33*(spatial.invF11*es.I_srz+spatial.inv	1	Spatial identity matrix, material frame, RZ-component	Domains 1–3	

Name	Expression	Unit	Description	Selection	Details
	F31*es.I_szz))*spatial.detF				
es.I_sPHIZ	if(Rg>0.001*h,R/r,Rr)*(spatial.invF13*es.I_sphir+spatial.invF33*es.I_sphiz)*spatial.detF	1	Spatial identity matrix, material frame, PHIZ-component	Domains 1–3	
es.I_sZZ	(spatial.invF13*(spatial.invF13*es.I_srr+spatial.invF33*es.I_szr)+spatial.invF33*(spatial.invF13*es.I_srz+spatial.invF33*es.I_szz))*spatial.detF	1	Spatial identity matrix, material frame, ZZ-component	Domains 1–3	
es.I_srr	1	1	Spatial identity matrix, rr-component	Domains 1–3	
es.I_sphir	0	1	Spatial identity matrix, phir-component	Domains 1–3	
es.I_szr	0	1	Spatial identity matrix, zr-component	Domains 1–3	
es.I_srphi	0	1	Spatial identity matrix, rphi-component	Domains 1–3	
es.I_sphiphi	1	1	Spatial identity matrix, phiphi-component	Domains 1–3	
es.I_szphi	0	1	Spatial identity matrix, zphi-component	Domains 1–3	
es.I_srz	0	1	Spatial identity matrix, rz-component	Domains 1–3	
es.I_sphiz	0	1	Spatial identity matrix, phiz-component	Domains 1–3	
es.I_szz	1	1	Spatial identity matrix, zz-component	Domains 1–3	
es.unTr	es.unTer	Pa	Maxwell upward surface stress tensor, r-	Boundaries 1–7, 9–17	

Name	Expression	Unit	Description	Selection	Details
			component		
es.unTphi	es.unTephi	Pa	Maxwell upward surface stress tensor, phi-component	Boundaries 1–7, 9–17	
es.unTz	es.unTez	Pa	Maxwell upward surface stress tensor, z-component	Boundaries 1–7, 9–17	
es.dnTr	es.dnTer	Pa	Maxwell downward surface stress tensor, r-component	Boundaries 1–7, 9–17	
es.dnTphi	es.dnTephi	Pa	Maxwell downward surface stress tensor, phi-component	Boundaries 1–7, 9–17	
es.dnTz	es.dnTez	Pa	Maxwell downward surface stress tensor, z-component	Boundaries 1–7, 9–17	
es.unr	unr		Normal vector up direction, r-component	Boundaries 1–7, 9–17	
es.unphi	0		Normal vector up direction, phi-component	Boundaries 1–7, 9–17	
es.unz	unz		Normal vector up direction, z-component	Boundaries 1–7, 9–17	
es.dnr	dnr		Normal vector down direction, r-component	Boundaries 1–7, 9–17	
es.dnphi	0		Normal vector down direction, phi-component	Boundaries 1–7, 9–17	
es.dnz	dnz		Normal vector down direction, z-component	Boundaries 1–7, 9–17	
es.unTer	-	Pa	Maxwell upward	Boundaries 5,	

Name	Expression	Unit	Description	Selection	Details
	0.5*es.dnr*(real(up(es.Dr))*real(up(es.Er))+real(up(es.Dphi))*real(up(es.Ephi))+real(up(es.Dz))*real(up(es.Ez)))+real(up(es.Dr))*(real(up(es.Er))*es.dnr+real(up(es.Ephi))*es.dnphi+real(up(es.Ez))*es.dnz)		electric surface stress tensor, r-component	17	
es.unTephi	-0.5*es.dnphi*(real(up(es.Dr))*real(up(es.Er))+real(up(es.Dphi))*real(up(es.Ephi))+real(up(es.Dz))*real(up(es.Ez)))+real(up(es.Dphi))*(real(up(es.Er))*es.dnr+real(up(es.Ephi))*es.dnphi+real(up(es.Ez))*es.dnz)	Pa	Maxwell upward electric surface stress tensor, phi-component	Boundaries 5, 17	
es.unTez	-0.5*es.dnz*(real(up(es.Dr))*real(up(es.Er))+real(up(es.Dphi))*real(up(es.Ephi))+real(up(es.Dz))*real(up(es.Ez)))+real(up(es.Dz))*(real(up(es.Er))*es.dnr+real(up(es.Ephi))*es.dnphi+real(up(es.Ez))*es.dnz)	Pa	Maxwell upward electric surface stress tensor, z-component	Boundaries 5, 17	
es.unTer	0	Pa	Maxwell upward electric surface stress tensor, r-component	Boundaries 1–4, 6–7, 9–16	
es.unTephi	0	Pa	Maxwell upward electric surface stress tensor, phi-component	Boundaries 1–4, 6–7, 9–16	
es.unTez	0	Pa	Maxwell upward electric surface stress tensor, z-component	Boundaries 1–4, 6–7, 9–16	
es.dnTer	-0.5*es.unr*(real(down(es.Dr))*real(down(es.E	Pa	Maxwell downward electric surface	Boundaries 1–7, 9–17	

Name	Expression	Unit	Description	Selection	Details
	r))+real(down(es.Dphi))*real(down(es.Ephi))+real(down(es.Dz))*real(down(es.Ez)))+real(down(es.Dr))*(real(down(es.Er))*es.unr+real(down(es.Ephi))*es.unphi+real(down(es.Ez))*es.unz)		stress tensor, r-component		
es.dnTephi	-0.5*es.unphi*(real(down(es.Dr))*real(down(es.Er))+real(down(es.Dphi))*real(down(es.Ephi))+real(down(es.Dz))*real(down(es.Ez)))+real(down(es.Dphi))*(real(down(es.Er))*es.unr+real(down(es.Ephi))*es.unphi+real(down(es.Ez))*es.unz)	Pa	Maxwell downward electric surface stress tensor, phi-component	Boundaries 1–7, 9–17	
es.dnTez	-0.5*es.unz*(real(down(es.Dr))*real(down(es.Er))+real(down(es.Dphi))*real(down(es.Ephi))+real(down(es.Dz))*real(down(es.Ez)))+real(down(es.Dz))*(real(down(es.Er))*es.unr+real(down(es.Ephi))*es.unphi+real(down(es.Ez))*es.unz)	Pa	Maxwell downward electric surface stress tensor, z-component	Boundaries 1–7, 9–17	
es.intWe	es.int_We(es.d*es.dWe)	J	Total electric energy	Global	+ operation
es.zref	50[ohm]	Ω	Reference impedance	Global	

2.4.3 Charge Conservation 1

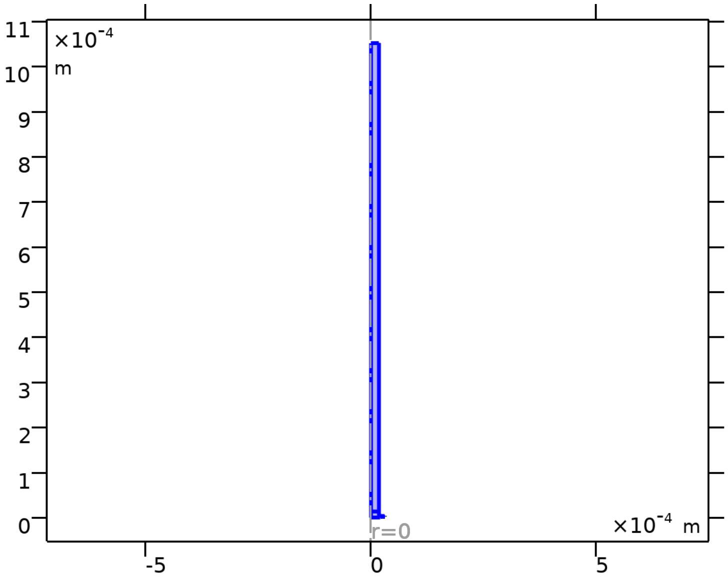

Charge Conservation 1

SELECTION

Geometric entity level	Domain
Selection	Geometry geom1: Dimension 2: All domains

EQUATIONS

$$\mathbf{E} = -\nabla V$$
$$\nabla \cdot (\epsilon_0 \epsilon_r \mathbf{E}) = \rho_v$$

Constitutive Relation D-E

SETTINGS

Description	Value
Dielectric model	Relative permittivity
Relative permittivity	User defined
Relative permittivity	80

Coordinate System Selection

SETTINGS

Description	Value
Coordinate system	Global coordinate system

Variables

Name	Expression	Unit	Description	Selection	Details

Name	Expression	Unit	Description	Selection	Details
es.nD	0	C/m²	Surface charge density	Boundaries 1–7, 9–17	+ operation
es.epsilonrrr	80	1	Relative permittivity, rr-component	Domains 1–3	
es.epsilonrphir	0	1	Relative permittivity, phir-component	Domains 1–3	
es.epsilonrzr	0	1	Relative permittivity, zr-component	Domains 1–3	
es.epsilonrrphi	0	1	Relative permittivity, rphi-component	Domains 1–3	
es.epsilonrphiphi	80	1	Relative permittivity, phiphi-component	Domains 1–3	
es.epsilonrzphi	0	1	Relative permittivity, zphi-component	Domains 1–3	
es.epsilonrrz	0	1	Relative permittivity, rz-component	Domains 1–3	
es.epsilonrphiz	0	1	Relative permittivity, phiz-component	Domains 1–3	
es.epsilonrzz	80	1	Relative permittivity, zz-component	Domains 1–3	
es.epsilonr_iso	80	1	Relative permittivity, isotropic value	Domains 1–3	
es.DrR	0	C/m²	Remanent electric displacement, R-component	Domains 1–3	
es.DrPHI	0	C/m²	Remanent electric displacement, PHI-component	Domains 1–3	
es.DrZ	0	C/m²	Remanent electric	Domains 1–3	

Name	Expression	Unit	Description	Selection	Details
			displacement, Z-component		
es.Dr	epsilon0_const*es.I_srr*es.Er+epsilon0_const*es.I_srphi*es.Ephi+epsilon0_const*es.I_srz*es.Ez+es.Pr+es.Per+es.Phr	C/m²	Electric displacement field, r-component	Domains 1–3	
es.Dphi	epsilon0_const*es.I_sphir*es.Er+epsilon0_const*es.I_sphiphi*es.Ephi+epsilon0_const*es.I_sphiz*es.Ez+es.Pphi+es.Pephi+es.Phphi	C/m²	Electric displacement field, phi-component	Domains 1–3	
es.Dz	epsilon0_const*es.I_szr*es.Er+epsilon0_const*es.I_szphi*es.Ephi+epsilon0_const*es.I_szz*es.Ez+es.Pz+es.Pez+es.Phz	C/m²	Electric displacement field, z-component	Domains 1–3	
es.Pr	epsilon0_const*(es.chirr*es.Er+es.chirphi*es.Ephi+es.chirz*es.Ez)	C/m²	Polarization, r-component	Domains 1–3	
es.Pphi	epsilon0_const*(es.chiphir*es.Er+es.chiphiphi*es.Ephi+es.chiphiz*es.Ez)	C/m²	Polarization, phi-component	Domains 1–3	
es.Pz	epsilon0_const*(es.chizr*es.Er+es.chizphi*es.Ephi+es.chizz*es.Ez)	C/m²	Polarization, z-component	Domains 1–3	
es.normD	sqrt(realdot(es.Dr,es.Dr)+realdot(es.Dphi,es.Dphi)+realdot(es.Dz,es.Dz))	C/m²	Electric displacement field norm	Domains 1–3	
es.normP	sqrt(realdot(es.Pr,es.Pr)+realdot(es.Pphi,es.Pphi)+realdot(es.Pz,es.Pz))	C/m²	Polarization norm	Domains 1–3	
es.Per	0	C/m²	Polarization contribution, r-component	Domains 1–3	+ operation

Name	Expression	Unit	Description	Selection	Details
es.Pephi	0	C/m²	Polarization contribution, phi-component	Domains 1–3	+ operation
es.Pez	0	C/m²	Polarization contribution, z-component	Domains 1–3	+ operation
es.Phr	0	C/m²	Polarization contribution, r-component	Domains 1–3	+ operation
es.Phphi	0	C/m²	Polarization contribution, phi-component	Domains 1–3	+ operation
es.Phz	0	C/m²	Polarization contribution, z-component	Domains 1–3	+ operation
es.chirr	-1+es.epsilonrrr	1	Electric susceptibility, rr-component	Domains 1–3	
es.chiphir	es.epsilonrphir	1	Electric susceptibility, phir-component	Domains 1–3	
es.chizr	es.epsilonrzr	1	Electric susceptibility, zr-component	Domains 1–3	
es.chirphi	es.epsilonrrphi	1	Electric susceptibility, rphi-component	Domains 1–3	
es.chiphiphi	-1+es.epsilonrphiphi	1	Electric susceptibility, phiphi-component	Domains 1–3	
es.chizphi	es.epsilonrzphi	1	Electric susceptibility, zphi-component	Domains 1–3	
es.chirz	es.epsilonrrz	1	Electric susceptibility, rz-component	Domains 1–3	
es.chiphiz	es.epsilonrphiz	1	Electric susceptibility, phiz-component	Domains 1–3	
es.chizz	-1+es.epsilonrzz	1	Electric susceptibility, zz-component	Domains 1–3	

Name	Expression	Unit	Description	Selection	Details
es.Er	-Vr	V/m	Electric field, r-component	Domains 1–3	
es.Ephi	0	V/m	Electric field, phi-component	Domains 1–3	
es.Ez	-Vz	V/m	Electric field, z-component	Domains 1–3	
es.tEr	-VTr	V/m	Tangential electric field, r-component	Boundaries 1–7, 9–17	
es.tEphi	0	V/m	Tangential electric field, phi-component	Boundaries 1–7, 9–17	
es.tEz	-VTz	V/m	Tangential electric field, z-component	Boundaries 1–7, 9–17	
es.normE	sqrt(realdot(es.Er,es.Er)+realdot(es.Ephi,es.Ephi)+realdot(es.Ez,es.Ez))	V/m	Electric field norm	Domains 1–3	
es.Jr	es.Jdr	A/m²	Current density, r-component	Domains 1–3	+ operation
es.Jphi	es.Jdphi	A/m²	Current density, phi-component	Domains 1–3	+ operation
es.Jz	es.Jdz	A/m²	Current density, z-component	Domains 1–3	+ operation
es.JR	(spatial.invF11*es.Jdr+spatial.invF31*es.Jdz)*spatial.detF	A/m²	Current density, R-component	Domains 1–3	+ operation
es.JPHI	if(Rg>0.001*h,R/r,Rr)*es.Jdphi*spatial.detF	A/m²	Current density, PHI-component	Domains 1–3	+ operation
es.JZ	(spatial.invF13*es.Jdr+spatial.invF33*es.Jdz)*spatial.detF	A/m²	Current density, Z-component	Domains 1–3	+ operation
es.Jdr	d(es.Dr,t)	A/m²	Displacement current density, r-component	Domains 1–3	
es.Jdphi	d(es.Dphi,t)	A/m²	Displacement current density, phi-component	Domains 1–3	
es.Jdz	d(es.Dz,t)	A/m²	Displacement current density,	Domains 1–3	

Name	Expression	Unit	Description	Selection	Details
			z-component		
es.normJ	sqrt(realdot(es.Jr,es.Jr)+realdot(es.Jphi,es.Jphi)+realdot(es.Jz,es.Jz))	A/m²	Current density norm	Domains 1–3	
es.ccn1.nJ	es.unr*down(es.Jr)+es.unphi*down(es.Jphi)+es.unz*down(es.Jz)	A/m²	Inward current density	Boundaries 1–4, 6–7, 9–16	
es.W	es.We	J/m³	Energy density	Domains 1–3	+ operation
es.dWe	2*es.We*pi*r	J/m²	Integrand for total electric energy	Domains 1–3	Meta
es.We	0.5*epsilon0_const*(((es.I_srr+es.chirr)*es.Er+(es.I_srphi+es.chirphi)*es.Ephi+(es.I_srz+es.chirz)*es.Ez)*es.Er+((es.I_sphir+es.chiphir)*es.Er+(es.I_sphiphi+es.chiphiphi)*es.Ephi+(es.I_sphiz+es.chiphiz)*es.Ez)*es.Ephi+((es.I_szr+es.chizr)*es.Er+(es.I_szphi+es.chizphi)*es.Ephi+(es.I_szz+es.chizz)*es.Ez)*es.Ez)	J/m³	Electric energy density	Domains 1–3	

Shape functions

Name	Shape function	Unit	Description	Shape frame	Selection
V	Lagrange (Quadratic)	V	Electric potential	Spatial	Domains 1–3
V	Lagrange (Quadratic)	V	Electric potential	Material	Domains 1–3
V	Lagrange (Quadratic)	V	Electric potential	Geometry	Domains 1–3
V	Lagrange (Quadratic)	V	Electric potential	Mesh	Domains 1–3

Weak Expressions

Weak expression	Integration order	Integration frame	Selection
-2*(es.Dr*test(Vr)+es.Dz*test(Vz))*es.d*pi*r	4	Spatial	Domains 1–3

2.4.4 Axial Symmetry 1

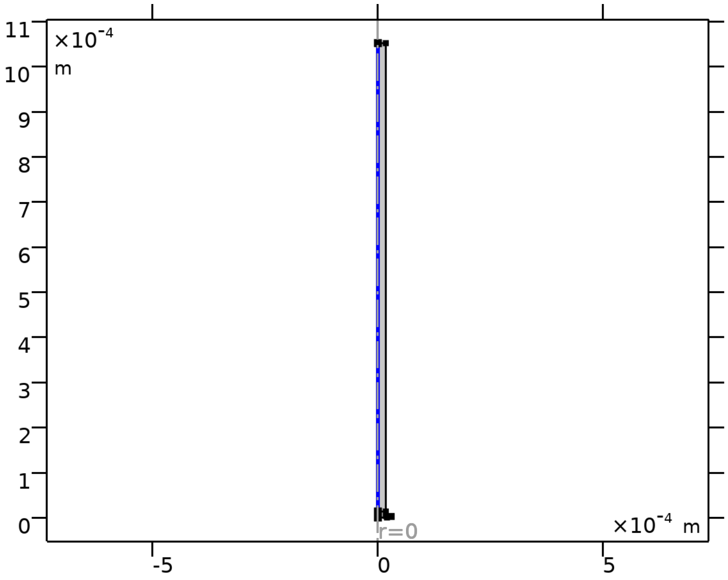

Axial Symmetry 1

SELECTION

Geometric entity level	Boundary
Selection	Geometry geom1: Dimension 1: All boundaries

2.4.5 Zero Charge 1

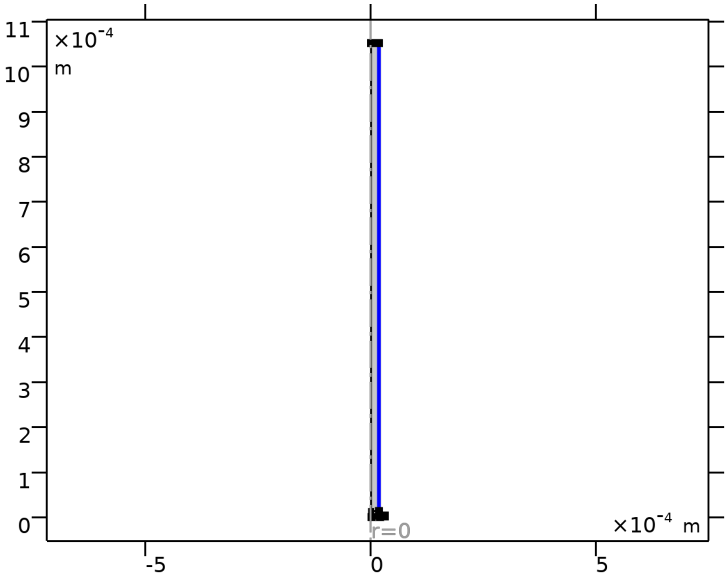

Zero Charge 1

SELECTION

Geometric entity level	Boundary

Selection	Geometry geom1: Dimension 1: All boundaries

EQUATIONS

$$\mathbf{n} \cdot \mathbf{D} = 0$$

Variables

Name	Expression	Unit	Description	Selection	Details
es.nD	0	C/m^2	Surface charge density	Boundaries 6–7, 9–16	+ operation

Shape functions

Name	Shape function	Unit	Description	Shape frame	Selection	Details
V	Lagrange (Quadratic)	V	Electric potential	Spatial	No boundaries	Slit
V	Lagrange (Quadratic)	V	Electric potential	Material	No boundaries	Slit
V	Lagrange (Quadratic)	V	Electric potential	Geometry	No boundaries	Slit
V	Lagrange (Quadratic)	V	Electric potential	Mesh	No boundaries	Slit

2.4.6 Initial Values 1

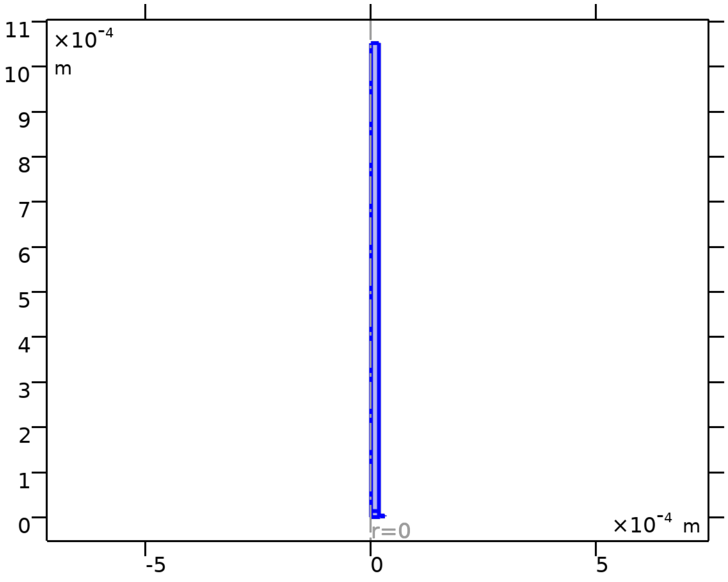

Initial Values 1

SELECTION

Geometric entity level	Domain

Selection	Geometry geom1: Dimension 2: All domains

SETTINGS

Description	Value	Unit
Electric potential	0	V

2.4.7 Electric Potential 1

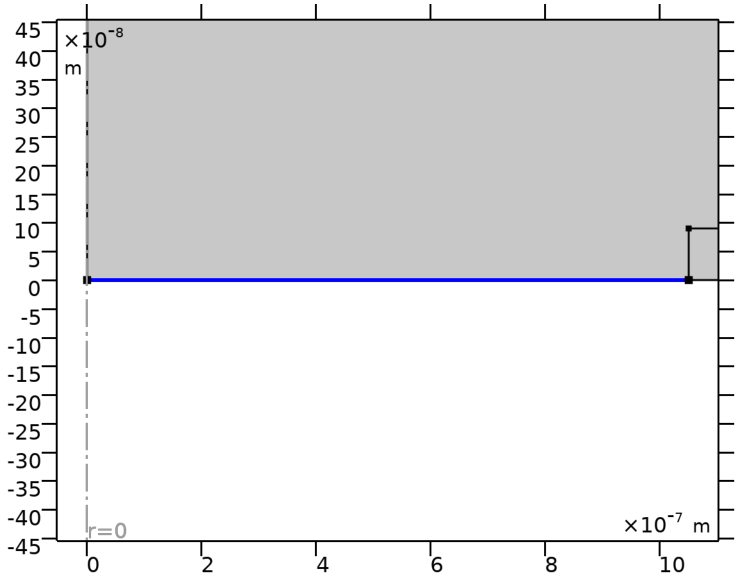

Electric Potential 1

SELECTION

Geometric entity level	Boundary
Selection	Geometry geom1: Dimension 1: Boundary 2

EQUATIONS

$$V = V_0$$

Electric Potential

SETTINGS

Description	Value	Unit
Electric potential	V	V

Variables

Name	Expression	Unit	Description	Selection	Details
es.nD	es.unr*down(es.Dr)+es.unphi*down(es.Dphi)+es.unz*down(es.Dz)	C/m^2	Surface charge density	Boundary 2	+ operation

Name	Expression	Unit	Description	Selection	Details
es.V0	V	V	Electric potential	Boundary 2	

Constraints

Constraint	Constraint force	Shape function	Selection	Details
es.V0-V	test(es.V0-V)	Lagrange (Quadratic)	Boundary 2	Elemental

2.5 ELECTROANALYSIS

USED PRODUCTS

COMSOL Multiphysics
Electrochemistry Module

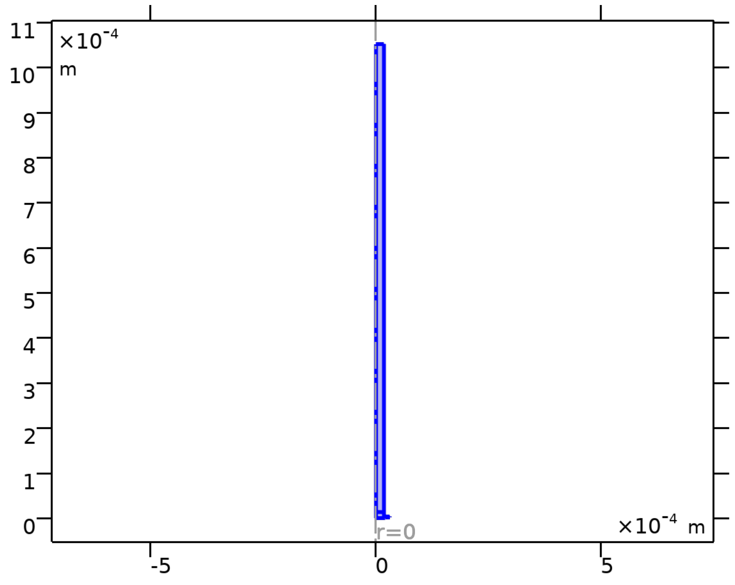

Electroanalysis

SELECTION

Geometric entity level	Domain
Selection	Geometry geom1: Dimension 2: Domains 1–3

EQUATIONS

$$\frac{\partial c_i}{\partial t} + \nabla \cdot \mathbf{J}_i + \mathbf{u} \cdot \nabla c_i = R_i$$
$$\mathbf{J}_i = -D_i \nabla c_i$$
$$\phi_l = 0$$

2.5.1 Interface Settings

Discretization

SETTINGS

Description	Value
Concentration	Linear

SETTINGS

Description	Value
Equation form	Study controlled

Transport Mechanisms

SETTINGS

Description	Value
Convection	On
Migration in electric field	Off

2.5.2 Variables

Name	Expression	Unit	Description	Selection	Details
domflux.c1r	2*elan.dflux_c1r*pi*r*elan.d	mol/(m·s)	Domain flux, r-component	Domains 1–3	
domflux.c1z	2*elan.dflux_c1z*pi*r*elan.d	mol/(m·s)	Domain flux, z-component	Domains 1–3	
domflux.c2r	2*elan.dflux_c2r*pi*r*elan.d	mol/(m·s)	Domain flux, r-component	Domains 1–3	
domflux.c2z	2*elan.dflux_c2z*pi*r*elan.d	mol/(m·s)	Domain flux, z-component	Domains 1–3	
elan.mulstopcond	1	1	Multiplicative stop condition	Global	* operation
elan.stopcond	elan.mulstopcond	1	Solver stop condition	Global	
elan.nr	nr	1	Normal vector, r-component	Boundaries 5, 17	
elan.nphi	0	1	Normal vector, phi-component	Boundaries 5, 17	
elan.nz	nz	1	Normal vector, z-component	Boundaries 5, 17	
elan.nr	dnr	1	Normal vector, r-component	Boundaries 1–4, 6–7, 9–16	
elan.nphi	0	1	Normal vector,	Boundaries 1–	

Name	Expression	Unit	Description	Selection	Details
			phi-component	4, 6–7, 9–16	
elan.nz	dnz	1	Normal vector, z-component	Boundaries 1–4, 6–7, 9–16	
elan.nrmesh	nrmesh	1	Normal vector (mesh), r-component	Boundaries 5, 17	
elan.nphimesh	0	1	Normal vector (mesh), phi-component	Boundaries 5, 17	
elan.nzmesh	nzmesh	1	Normal vector (mesh), z-component	Boundaries 5, 17	
elan.nrmesh	dnrmesh	1	Normal vector (mesh), r-component	Boundaries 1–4, 6–7, 9–16	
elan.nphimesh	0	1	Normal vector (mesh), phi-component	Boundaries 1–4, 6–7, 9–16	
elan.nzmesh	dnzmesh	1	Normal vector (mesh), z-component	Boundaries 1–4, 6–7, 9–16	
elan.nrc	root.nrc/elan.ncLen	1	Normal vector, r-component	Boundaries 1–7, 9–17	
elan.nphic	0	1	Normal vector, phi-component	Boundaries 1–7, 9–17	
elan.nzc	root.nzc/elan.ncLen	1	Normal vector, z-component	Boundaries 1–7, 9–17	
elan.ncLen	sqrt(root.nrc^2+root.nzc^2+eps)	1	Help variable	Boundaries 1–7, 9–17	
elan.ndflux_c1	elan.bndFlux_c1	mol/(m²·s)	Normal diffusive flux	Boundaries 2, 5–7, 9–17	
elan.ncflux_c1	elan.cflux_c1r*elan.nrc+elan.cflux_c1phi*elan.nphic+elan.cflux_c1z*elan.nzc	mol/(m²·s)	Normal convective flux	Boundaries 2, 5–7, 9–17	
elan.ntflux_c1	elan.bndFlux_c1+elan.cflux_c1r*elan.nrc+elan.cflux_c1phi*elan.nphic+elan.cflux_c1z*elan.nzc	mol/(m²·s)	Normal total flux	Boundaries 2, 5–7, 9–17	
elan.ndflux_c2	elan.bndFlux_c2	mol/(m²·s)	Normal diffusive flux	Boundaries 2, 5–7, 9–17	

Name	Expression	Unit	Description	Selection	Details
elan.ncflux_c2	elan.cflux_c2r*elan.nrc+elan.cflux_c2phi*elan.nphic+elan.cflux_c2z*elan.nzc	mol/(m²·s)	Normal convective flux	Boundaries 2, 5–7, 9–17	
elan.ntflux_c2	elan.bndFlux_c2+elan.cflux_c2r*elan.nrc+elan.cflux_c2phi*elan.nphic+elan.cflux_c2z*elan.nzc	mol/(m²·s)	Normal total flux	Boundaries 2, 5–7, 9–17	
elan.bndFlux_c1	0.25*(uflux_spatial(c1)-dflux_spatial(c1))/(pi*r*elan.d)	mol/(m²·s)	Boundary flux	Boundaries 5, 17	Meta
elan.bndFlux_c1	-dflux_spatial(c1)/elan.d	mol/(m²·s)	Boundary flux	Boundaries 1, 3–4	
elan.bndFlux_c1	-0.5*dflux_spatial(c1)/(pi*r*elan.d)	mol/(m²·s)	Boundary flux	Boundaries 2, 6–7, 9–16	Meta
elan.bndFlux_c2	0.25*(uflux_spatial(c2)-dflux_spatial(c2))/(pi*r*elan.d)	mol/(m²·s)	Boundary flux	Boundaries 5, 17	Meta
elan.bndFlux_c2	-dflux_spatial(c2)/elan.d	mol/(m²·s)	Boundary flux	Boundaries 1, 3–4	
elan.bndFlux_c2	-0.5*dflux_spatial(c2)/(pi*r*elan.d)	mol/(m²·s)	Boundary flux	Boundaries 2, 6–7, 9–16	Meta
elan.R_c1	0	mol/(m³·s)	Total rate expression	Domains 1–3	+ operation
elan.cP_c1	0	mol/kg	Concentration species adsorbed to the solid	Domains 1–3	+ operation
elan.cP_c1	0	mol/kg	Concentration species adsorbed to the solid	Boundaries 1–7, 9–17	+ operation
elan.KP_c1	0	m³/kg	Adsorption isotherm, first concentration derivative	Domains 1–3	+ operation
elan.KP_c1	0	m³/kg	Adsorption isotherm, first	Boundaries 1–7, 9–17	+ operation

Name	Expression	Unit	Description	Selection	Details
			concentration derivative		
elan.Rads_c1	0	mol/(m³·s)	Total adsorption rate	Domains 1–3	+ operation
elan.DiT_c1	0	m²/s	Turbulent diffusivity	Domains 1–3	
elan.cVar_c1	c1	mol/m³	Species	Boundaries 1–7, 9–17	
elan.cVar_c1	c1	mol/m³	Species	Points 1–7, 9–15	
elan.R_c2	0	mol/(m³·s)	Total rate expression	Domains 1–3	+ operation
elan.cP_c2	0	mol/kg	Concentration species adsorbed to the solid	Domains 1–3	+ operation
elan.cP_c2	0	mol/kg	Concentration species adsorbed to the solid	Boundaries 1–7, 9–17	+ operation
elan.KP_c2	0	m³/kg	Adsorption isotherm, first concentration derivative	Domains 1–3	+ operation
elan.KP_c2	0	m³/kg	Adsorption isotherm, first concentration derivative	Boundaries 1–7, 9–17	+ operation
elan.Rads_c2	0	mol/(m³·s)	Total adsorption rate	Domains 1–3	+ operation
elan.DiT_c2	0	m²/s	Turbulent diffusivity	Domains 1–3	
elan.cVar_c2	c2	mol/m³	Species	Boundaries 1–7, 9–17	
elan.cVar_c2	c2	mol/m³	Species	Points 1–7, 9–15	
elan.cbf_c1	0	mol/(m²·s)	Convective boundary flux	Boundaries 1–7, 9–17	
elan.cbf_c2	0	mol/(m²·s)	Convective boundary flux	Boundaries 1–7, 9–17	
elan.d	1	1	Out-of-plane geometry extension	Global	
elan.nil	0	A/m²	Inward electrolyte	Domains 1–3	+ operation

Name	Expression	Unit	Description	Selection	Details
			current density		

2.5.3 Transport Properties 1

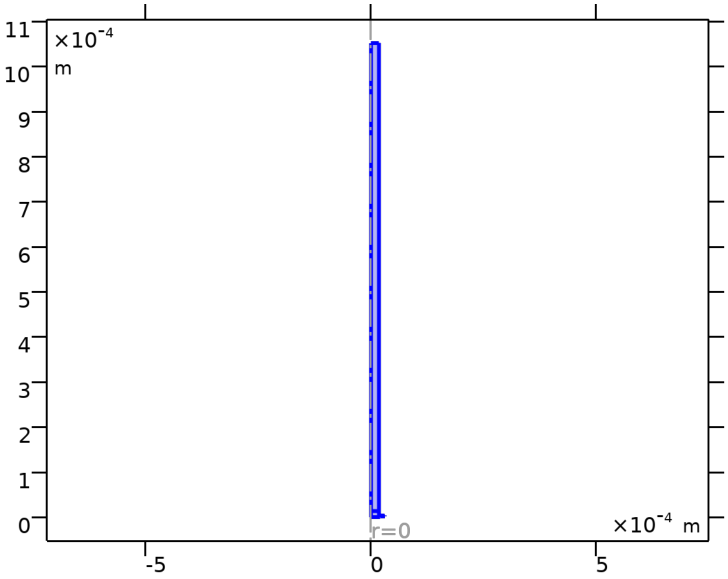

Transport Properties 1

SELECTION

Geometric entity level	Domain
Selection	Geometry geom1: Dimension 2: All domains

EQUATIONS

$$\frac{\partial c_i}{\partial t} + \nabla \cdot \mathbf{J}_i + \mathbf{u} \cdot \nabla c_i = R_i$$

$$\mathbf{J}_i = -D_i \nabla c_i$$

$$\phi_l = 0$$

Diffusion

SETTINGS

Description	Value	Unit
Material	None	
Diffusion coefficient	User defined	
Diffusion coefficient	DR	m^2/s
Diffusion coefficient	User defined	
Diffusion coefficient	DO	m^2/s

Coordinate System Selection

SETTINGS

Description	Value
Coordinate system	Global coordinate system

Model Input

SETTINGS

Description	Value	Unit
Temperature	User defined	
Temperature	293.15	K

Variables

Name	Expression	Unit	Description	Selection	Details
elan.DF_c1rr	DR	m²/s	Fluid diffusion coefficient, rr-component	Domains 1–3	
elan.DF_c1phir	0	m²/s	Fluid diffusion coefficient, phir-component	Domains 1–3	
elan.DF_c1zr	0	m²/s	Fluid diffusion coefficient, zr-component	Domains 1–3	
elan.DF_c1rphi	0	m²/s	Fluid diffusion coefficient, rphi-component	Domains 1–3	
elan.DF_c1phiphi	DR	m²/s	Fluid diffusion coefficient, phiphi-component	Domains 1–3	
elan.DF_c1zphi	0	m²/s	Fluid diffusion coefficient, zphi-component	Domains 1–3	
elan.DF_c1rz	0	m²/s	Fluid diffusion coefficient, rz-component	Domains 1–3	
elan.DF_c1phiz	0	m²/s	Fluid diffusion coefficient, phiz-component	Domains 1–3	
elan.DF_c1zz	DR	m²/s	Fluid diffusion coefficient, zz-component	Domains 1–3	
elan.D_c1rr	elan.DF_c1rr+elan.DiT_c1	m²/s	Diffusion coefficient, rr-	Domains 1–3	+ operation

Name	Expression	Unit	Description	Selection	Details
			component		
elan.D_c1phir	elan.DF_c1phir	m²/s	Diffusion coefficient, phir-component	Domains 1–3	+ operation
elan.D_c1zr	elan.DF_c1zr	m²/s	Diffusion coefficient, zr-component	Domains 1–3	+ operation
elan.D_c1rphi	elan.DF_c1rphi	m²/s	Diffusion coefficient, rphi-component	Domains 1–3	+ operation
elan.D_c1phiphi	elan.DF_c1phiphi+ elan.DiT_c1	m²/s	Diffusion coefficient, phiphi-component	Domains 1–3	+ operation
elan.D_c1zphi	elan.DF_c1zphi	m²/s	Diffusion coefficient, zphi-component	Domains 1–3	+ operation
elan.D_c1rz	elan.DF_c1rz	m²/s	Diffusion coefficient, rz-component	Domains 1–3	+ operation
elan.D_c1phiz	elan.DF_c1phiz	m²/s	Diffusion coefficient, phiz-component	Domains 1–3	+ operation
elan.D_c1zz	elan.DF_c1zz+elan. DiT_c1	m²/s	Diffusion coefficient, zz-component	Domains 1–3	+ operation
elan.DF_c2rr	DO	m²/s	Fluid diffusion coefficient, rr-component	Domains 1–3	
elan.DF_c2phir	0	m²/s	Fluid diffusion coefficient, phir-component	Domains 1–3	
elan.DF_c2zr	0	m²/s	Fluid diffusion coefficient, zr-component	Domains 1–3	
elan.DF_c2rphi	0	m²/s	Fluid diffusion coefficient, rphi-component	Domains 1–3	
elan.DF_c2phiphi	DO	m²/s	Fluid diffusion coefficient, phiphi-component	Domains 1–3	
elan.DF_c2zphi	0	m²/s	Fluid diffusion coefficient, zphi-component	Domains 1–3	

Name	Expression	Unit	Description	Selection	Details
elan.DF_c2rz	0	m^2/s	Fluid diffusion coefficient, rz-component	Domains 1–3	
elan.DF_c2phiz	0	m^2/s	Fluid diffusion coefficient, phiz-component	Domains 1–3	
elan.DF_c2zz	DO	m^2/s	Fluid diffusion coefficient, zz-component	Domains 1–3	
elan.D_c2rr	elan.DF_c2rr+elan.DiT_c2	m^2/s	Diffusion coefficient, rr-component	Domains 1–3	+ operation
elan.D_c2phir	elan.DF_c2phir	m^2/s	Diffusion coefficient, phir-component	Domains 1–3	+ operation
elan.D_c2zr	elan.DF_c2zr	m^2/s	Diffusion coefficient, zr-component	Domains 1–3	+ operation
elan.D_c2rphi	elan.DF_c2rphi	m^2/s	Diffusion coefficient, rphi-component	Domains 1–3	+ operation
elan.D_c2phiphi	elan.DF_c2phiphi+elan.DiT_c2	m^2/s	Diffusion coefficient, phiphi-component	Domains 1–3	+ operation
elan.D_c2zphi	elan.DF_c2zphi	m^2/s	Diffusion coefficient, zphi-component	Domains 1–3	+ operation
elan.D_c2rz	elan.DF_c2rz	m^2/s	Diffusion coefficient, rz-component	Domains 1–3	+ operation
elan.D_c2phiz	elan.DF_c2phiz	m^2/s	Diffusion coefficient, phiz-component	Domains 1–3	+ operation
elan.D_c2zz	elan.DF_c2zz+elan.DiT_c2	m^2/s	Diffusion coefficient, zz-component	Domains 1–3	+ operation
elan.Dav_c1	0.5*(elan.D_c1rr+elan.D_c1zz)	m^2/s	Average diffusion coefficient	Domains 1–3	
elan.Dav_c2	0.5*(elan.D_c2rr+elan.D_c2zz)	m^2/s	Average diffusion coefficient	Domains 1–3	
elan.tflux_c1r	elan.dflux_c1r+elan.cflux_c1r	$mol/(m^2 \cdot s)$	Total flux, r-component	Domains 1–3	+ operation

Name	Expression	Unit	Description	Selection	Details
elan.tflux_c1phi	elan.dflux_c1phi+elan.cflux_c1phi	mol/(m²·s)	Total flux, phi-component	Domains 1–3	+ operation
elan.tflux_c1z	elan.dflux_c1z+elan.cflux_c1z	mol/(m²·s)	Total flux, z-component	Domains 1–3	+ operation
elan.dfluxMag_c1	sqrt(elan.dflux_c1r^2+elan.dflux_c1phi^2+elan.dflux_c1z^2)	mol/(m²·s)	Diffusive flux magnitude	Domains 1–3	
elan.tfluxMag_c1	sqrt(elan.tflux_c1r^2+elan.tflux_c1phi^2+elan.tflux_c1z^2)	mol/(m²·s)	Total flux magnitude	Domains 1–3	
elan.dpflux_c1r	0	mol/(m²·s)	Dispersive flux, r-component	Domains 1–3	
elan.dpflux_c1phi	0	mol/(m²·s)	Dispersive flux, phi-component	Domains 1–3	
elan.dpflux_c1z	0	mol/(m²·s)	Dispersive flux, z-component	Domains 1–3	
elan.tflux_c2r	elan.dflux_c2r+elan.cflux_c2r	mol/(m²·s)	Total flux, r-component	Domains 1–3	+ operation
elan.tflux_c2phi	elan.dflux_c2phi+elan.cflux_c2phi	mol/(m²·s)	Total flux, phi-component	Domains 1–3	+ operation
elan.tflux_c2z	elan.dflux_c2z+elan.cflux_c2z	mol/(m²·s)	Total flux, z-component	Domains 1–3	+ operation
elan.dfluxMag_c2	sqrt(elan.dflux_c2r^2+elan.dflux_c2phi^2+elan.dflux_c2z^2)	mol/(m²·s)	Diffusive flux magnitude	Domains 1–3	
elan.tfluxMag_c2	sqrt(elan.tflux_c2r^2+elan.tflux_c2phi^2+elan.tflux_c2z^2)	mol/(m²·s)	Total flux magnitude	Domains 1–3	
elan.dpflux_c2r	0	mol/(m²·s)	Dispersive flux, r-component	Domains 1–3	
elan.dpflux_c2phi	0	mol/(m²·s)	Dispersive flux, phi-component	Domains 1–3	
elan.dpflux_c2z	0	mol/(m²·s)	Dispersive flux, z-component	Domains 1–3	
elan.dflux_c1r	-elan.D_c1rr*c1r-elan.D_c1rz*c1z	mol/(m²·s)	Diffusive flux, r-component	Domains 1–3	+ operation
elan.dflux_c1phi	-elan.D_c1phir*c1r-elan.D_c1phiz*c1z	mol/(m²·s)	Diffusive flux, phi-component	Domains 1–3	+ operation

Name	Expression	Unit	Description	Selection	Details
elan.dflux_c1z	-elan.D_c1zr*c1r-elan.D_c1zz*c1z	mol/(m²·s)	Diffusive flux, z-component	Domains 1–3	+ operation
elan.grad_c1r	c1r	mol/m⁴	Concentration gradient, r-component	Domains 1–3	
elan.grad_c1phi	0	mol/m⁴	Concentration gradient, phi-component	Domains 1–3	
elan.grad_c1z	c1z	mol/m⁴	Concentration gradient, z-component	Domains 1–3	
elan.dflux_c2r	-elan.D_c2rr*c2r-elan.D_c2rz*c2z	mol/(m²·s)	Diffusive flux, r-component	Domains 1–3	+ operation
elan.dflux_c2phi	-elan.D_c2phir*c2r-elan.D_c2phiz*c2z	mol/(m²·s)	Diffusive flux, phi-component	Domains 1–3	+ operation
elan.dflux_c2z	-elan.D_c2zr*c2r-elan.D_c2zz*c2z	mol/(m²·s)	Diffusive flux, z-component	Domains 1–3	+ operation
elan.grad_c2r	c2r	mol/m⁴	Concentration gradient, r-component	Domains 1–3	
elan.grad_c2phi	0	mol/m⁴	Concentration gradient, phi-component	Domains 1–3	
elan.grad_c2z	c2z	mol/m⁴	Concentration gradient, z-component	Domains 1–3	
elan.u	model.input.u1	m/s	Velocity field, r-component	Domains 1–3	Meta
elan.v	model.input.u2	m/s	Velocity field, phi-component	Domains 1–3	Meta
elan.w	model.input.u3	m/s	Velocity field, z-component	Domains 1–3	Meta
elan.cflux_c1r	c1*elan.u	mol/(m²·s)	Convective flux, r-component	Domains 1–3	
elan.cflux_c1phi	c1*elan.v	mol/(m²·s)	Convective flux, phi-component	Domains 1–3	
elan.cflux_c1z	c1*elan.w	mol/(m²·s)	Convective flux, z-component	Domains 1–3	
elan.cfluxMag_c1	sqrt(elan.cflux_c1r^2+elan.cflux_c1phi^2+elan.cflux_c1z^2)	mol/(m²·s)	Convective flux magnitude	Domains 1–3	

Name	Expression	Unit	Description	Selection	Details
elan.cflux_c2r	c2*elan.u	mol/(m²·s)	Convective flux, r-component	Domains 1–3	
elan.cflux_c2phi	c2*elan.v	mol/(m²·s)	Convective flux, phi-component	Domains 1–3	
elan.cflux_c2z	c2*elan.w	mol/(m²·s)	Convective flux, z-component	Domains 1–3	
elan.cfluxMag_c2	sqrt(elan.cflux_c2r^2+elan.cflux_c2phi^2+elan.cflux_c2z^2)	mol/(m²·s)	Convective flux magnitude	Domains 1–3	
elan.phil	0	V	Electrolyte potential	Domains 1–3	
elan.Rlin_c1	0	1/s	Linear source term coefficient	Domains 1–3	+ operation
elan.Res_c1	d(c1,t)+elan.u*c1r+elan.w*c1z-c1*elan.Rlin_c1-elan.R_c1	mol/(m³·s)	Equation residual	Domains 1–3	
elan.Rlin_c2	0	1/s	Linear source term coefficient	Domains 1–3	+ operation
elan.Res_c2	d(c2,t)+elan.u*c2r+elan.w*c2z-c2*elan.Rlin_c2-elan.R_c2	mol/(m³·s)	Equation residual	Domains 1–3	

Shape functions

Name	Shape function	Unit	Description	Shape frame	Selection
c1	Lagrange (Linear)	mol/m³	Concentration	Spatial	Domains 1–3
c2	Lagrange (Linear)	mol/m³	Concentration	Spatial	Domains 1–3

Weak Expressions

Weak expression	Integration order	Integration frame	Selection
2*(-c1t*test(c1)+elan.dflux_c1r*test(c1r)+elan.dflux_c1z*test(c1z))*elan.d*pi*r	2	Spatial	Domains 1–3
2*(-c2t*test(c2)+elan.dflux_c2r*test(c2r)+elan.dflux_c2z*test(c2z))*elan.d*pi*r	2	Spatial	Domains 1–3
-2*(elan.u*c1r+elan.w*c1z)*test(c1)*(isScalingSystemDomain==0)*elan.d*pi*r	2	Spatial	Domains 1–3

Weak expression	Integration order	Integration frame	Selection
2*elan.cbf_c1*test(c1)*elan.d*pi*r	2	Spatial	Boundaries 1–7, 9–17
-2*(elan.u*c2r+elan.w*c2z)*test(c2)*(isScalingSystemDomain==0)*elan.d*pi*r	2	Spatial	Domains 1–3
2*elan.cbf_c2*test(c2)*elan.d*pi*r	2	Spatial	Boundaries 1–7, 9–17
2*elan.streamline*(isScalingSystemDomain==0)*elan.d*pi*r	2	Spatial	Domains 1–3
2*elan.crosswind*(isScalingSystemDomain==0)*elan.d*pi*r	4	Spatial	Domains 1–3

2.5.4 Axial Symmetry 1

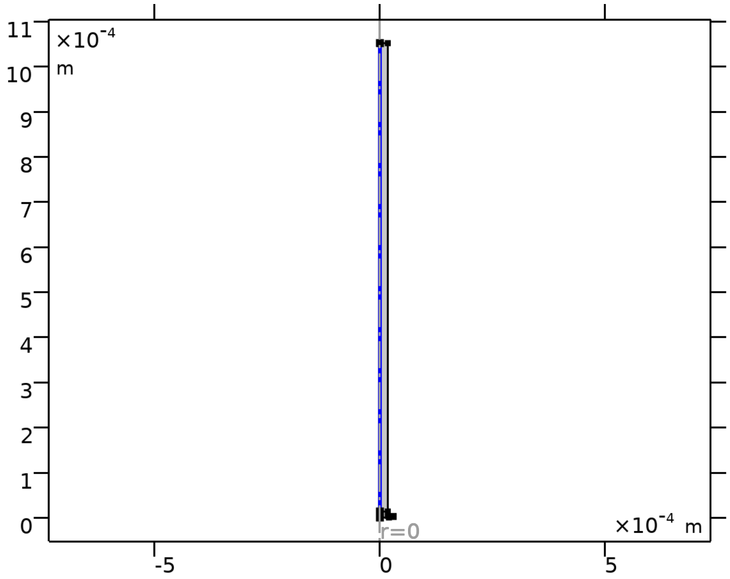

Axial Symmetry 1

SELECTION

Geometric entity level	Boundary
Selection	Geometry geom1: Dimension 1: All boundaries

2.5.5 No Flux 1

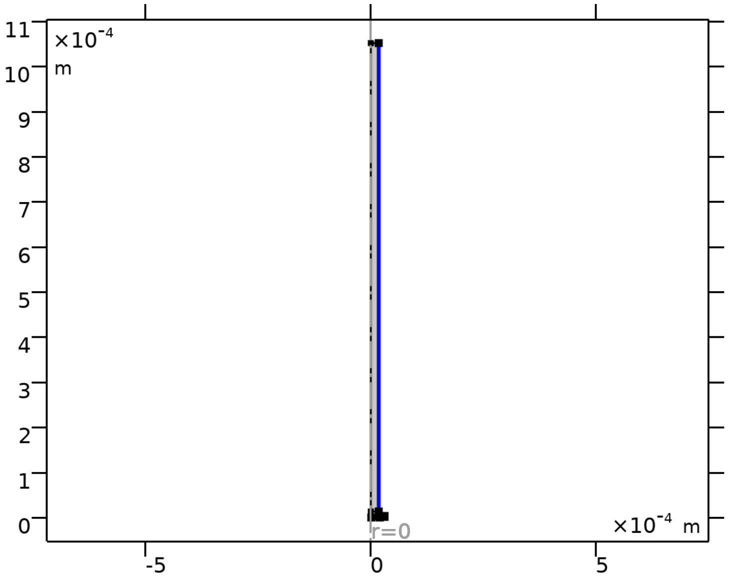

No Flux 1

SELECTION

Geometric entity level	Boundary
Selection	Geometry geom1: Dimension 1: All boundaries

EQUATIONS

$$-\mathbf{n}\cdot\left(\mathbf{J}_i+\mathbf{u}c_i\right)=0$$

Convection

SETTINGS

Description	Value
Include	On

Variables

Name	Expression	Unit	Description	Selection
elan.cbf_c1	c1*(elan.u*elan.nrmesh+elan.v*elan.nphimesh+elan.w*elan.nzmesh)	mol/(m²·s)	Convective boundary flux	Boundaries 7, 9–16
elan.cbf_c2	c2*(elan.u*elan.nrmesh+elan.v*elan.nphimesh+elan.w*elan.nzmesh)	mol/(m²·s)	Convective boundary flux	Boundaries 7, 9–16

2.5.6 Initial Values 1

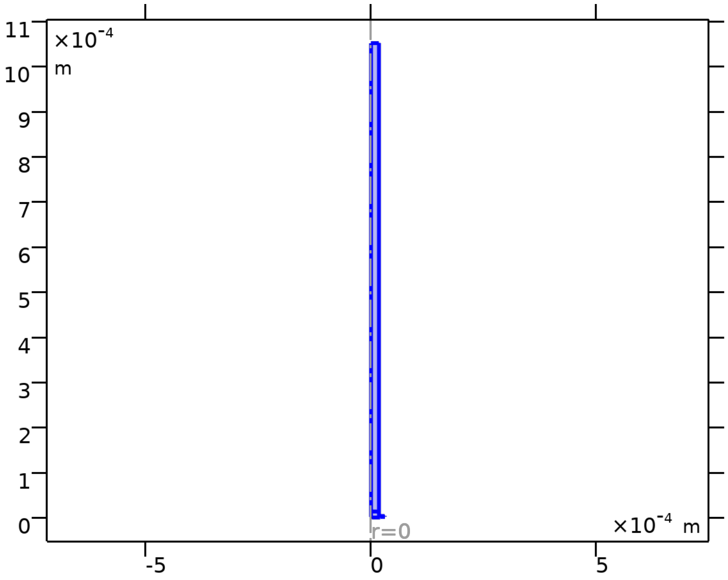

Initial Values 1

SELECTION

Geometric entity level	Domain
Selection	Geometry geom1: Dimension 2: All domains

Initial Values

SETTINGS

Description	Value	Unit
Concentration	{cRbulk, 0}	mol/m³

Variables

Name	Expression	Unit	Description	Selection	Details
elan.c0_c1	cRbulk	mol/m³	Concentration	Domains 2–3	+ operation
elan.c0_c2	0	mol/m³	Concentration	Domains 2–3	+ operation

2.5.7 Concentration 1

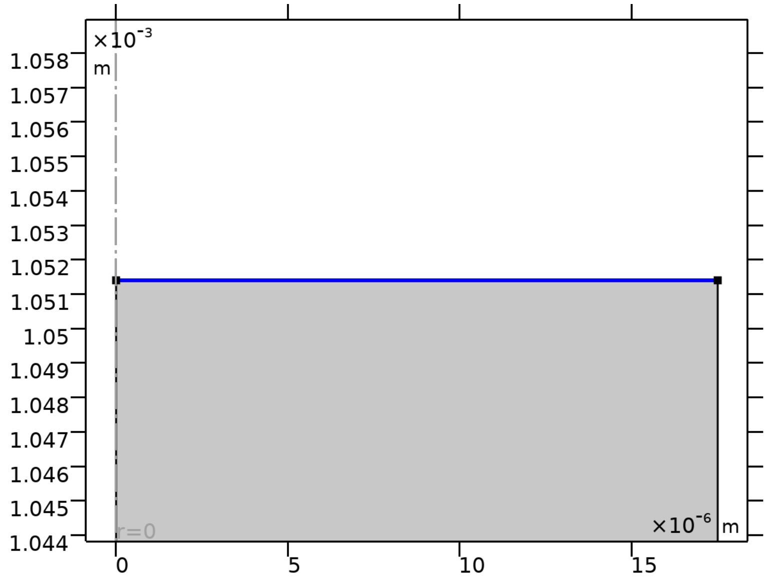

Concentration 1

SELECTION

Geometric entity level	Boundary
Selection	Geometry geom1: Dimension 1: Boundary 6

EQUATIONS

$$c_i = c_{0,i}$$

Concentration

SETTINGS

Description	Value	Unit
Species c1	On	
Species c2	On	
Concentration	{cRbulk, 0}	mol/m^3

Variables

Name	Expression	Unit	Description	Selection	Details
elan.c0_c1	cRbulk	mol/m^3	Concentration	Boundary 6	+ operation
elan.c0_c2	0	mol/m^3	Concentration	Boundary 6	+ operation
elan.conc1.nmflow_c1	elan.conc1.int(2*elan.ntflux_c1*pi*r)*elan.d	mol/s	Normal molar flow rate	Global	
elan.conc1.nmflow_c2	elan.conc1.int(2*elan.ntflux_c2*pi*r)*el	mol/s	Normal molar flow rate	Global	

Name	Expression	Unit	Description	Selection	Details
	an.d				

Constraints

Constraint	Constraint force	Shape function	Selection	Details
-elan.cVar_c1+elan.c0_c1	test(-elan.cVar_c1+elan.c0_c1)	Lagrange (Linear)	Boundary 6	Elemental
-elan.cVar_c2+elan.c0_c2	test(-elan.cVar_c2+elan.c0_c2)	Lagrange (Linear)	Boundary 6	Elemental

2.5.8 Electrode Surface 1

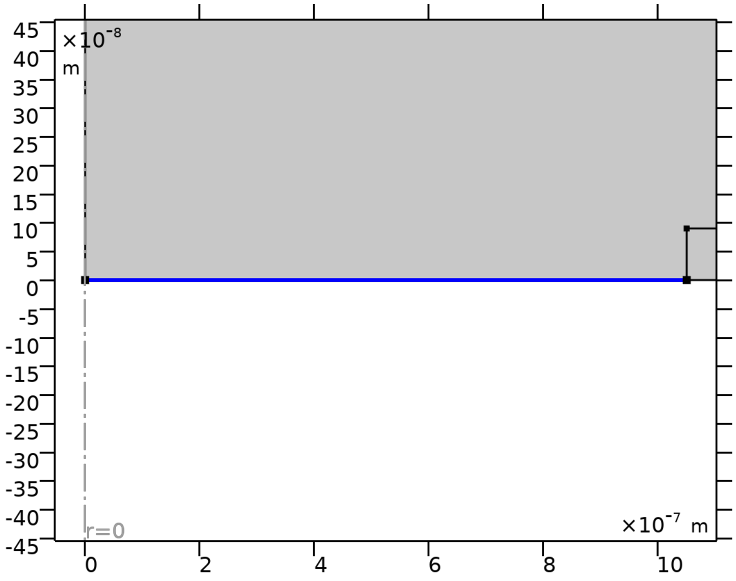

Electrode Surface 1

SELECTION

Geometric entity level	Boundary
Selection	Geometry geom1: Dimension 1: Boundary 2

EQUATIONS

$$i_{\text{total}} = \sum_m i_{\text{loc},m} + i_{\text{dl}}$$

$$-\mathbf{n} \cdot \mathbf{J}_i = R_{i,\text{tot}}, \quad R_{i,\text{tot}} = \sum_m R_{i,m}$$

Dissolving-Depositing Species

SETTINGS

Description	Value
Species	
Solve for surface concentration variables	On

Film Resistance

SETTINGS

Description	Value
Film resistance	No film resistance

Harmonic Perturbation

SETTINGS

Description	Value	Unit
Perturbation amplitude	0	V

Boundary Condition

SETTINGS

Description	Value	Unit
Boundary condition	Cyclic voltammetry	1
Linear sweep rate	v_sweep	V/s
Start potential	Off	
Number of cycles	2	1
Vertex potential 1	E_start	V
Vertex potential 2	E_vertex	V
End potential	Off	

Variables

Name	Expression	Unit	Description	Selection	Details
elan.mulstopcond	t>elan.ncycle_els1*abs(2*(E_start-E_vertex))/v_sweep	1	Multiplicative stop condition	Global	* operation
elan.nil	elan.itot	A/m^2	Inward electrolyte current density	Boundary 2	+ operation
elan.ncycle_els1	2	1	Number of cycles	Global	
elan.phis_els1	elan.els1.int(elan.phisext*elan.dvolfactor*elan.d)/elan.Area_els1	V	Electric potential	Global	
elan.phisext	elan.cv_els1(t)	V	External electric potential	Boundary 2	

Name	Expression	Unit	Description	Selection	Details
elan.dvolfactor	2*pi*r	m	Differential volume factor	Boundary 2	Meta
elan.Area_els1	elan.els1.int(elan.dvolfactor*elan.d)	m²	Area	Global	
elan.itotavg_els1	elan.els1.int(elan.itot*elan.dvolfactor*elan.d)/elan.Area_els1	A/m²	Average total interface current density	Global	
elan.Temp	elan.els1.minput_temperature	K	Temperature	Boundary 2	
elan.Evsref	elan.phisext-elan.phil	V	Electrode potential vs. adjacent reference	Boundary 2	
elan.Ect	elan.phisext-elan.phil	V	Electrode potential	Boundary 2	
elan.Ectmat	elan.Ect	V	Electrode potential	Boundary 2	
elan.Itot_els1	elan.els1.int(elan.itot*elan.dvolfactor*elan.d)	A	Total current	Global	
elan.itot	0	A/m²	Total interface current density	Boundary 2	+ operation
elan.rhos	8960	kg/m³	Density	Boundary 2	
elan.Ms	0.06355	kg/mol	Molar mass	Boundary 2	
elan.cycle_els1	max(round(0.5+min(t,(-1+elan.ncycle_els1)*abs(2*(E_start-E_vertex))/v_sweep)*v_sweep/abs(2*(E_start-E_vertex))),1)	1	Cycle number	Global	

Electrode Reaction 1

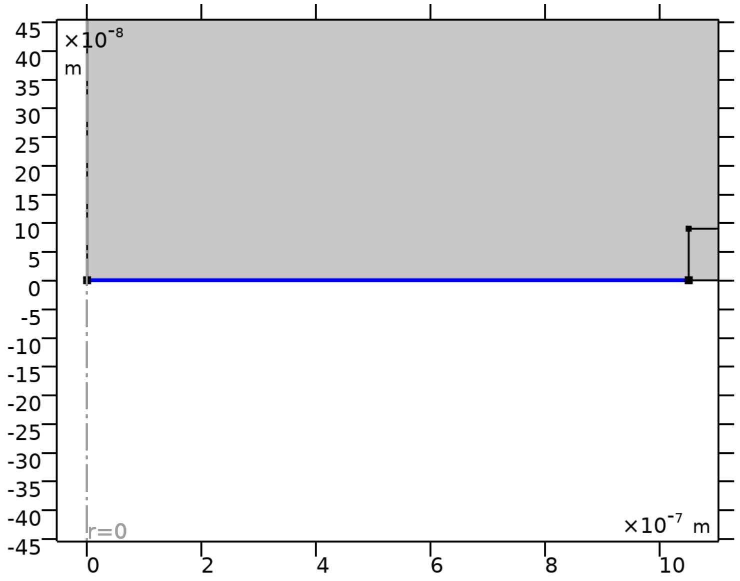

Electrode Reaction 1

SELECTION

Geometric entity level	Boundary
Selection	Geometry geom1: Dimension 1: All boundaries

EQUATIONS

$$\eta = E_{ct} - E_{eq}, \quad E_{ct} = \phi_{s,ext} - \phi_l$$

$$R_i = \frac{-\nu_i i_{loc}}{nF}$$

Equilibrium Potential

SETTINGS

Description	Value	Unit
Equilibrium potential	User defined	
Equilibrium potential	Ef	V

Electrode Kinetics

SETTINGS

Description	Value	Unit
Kinetics expression type	Electroanalytical Butler - Volmer	
Heterogeneous rate constant	k0	m/s
Cathodic transfer coefficient	a	1

Stoichiometric Coefficients

SETTINGS

Description	Value
Number of participating electrons	1
Stoichiometric coefficient	{1, -1}

Heat of Reaction

SETTINGS

Description	Value	Unit
Specify	Temperature derivative	
Temperature derivative of equilibrium potential	User defined	
Temperature derivative of equilibrium potential	0	V/K

Model Input

SETTINGS

Description	Value	Unit
Temperature	User defined	
Temperature	293.15	K

Variables

Name	Expression	Unit	Description	Selection	Details
elan.itot	elan.iloc_er1	A/m²	Total interface current density	Boundary 2	+ operation
elan.Eeq_er1	Ef	V	Equilibrium potential, Electrode Reaction 1	Boundary 2	
elan.alphac_er1	a	1	Cathodic transfer coefficient	Boundary 2	
elan.k0_er1	k0	m/s	Heterogeneous rate constant	Boundary 2	
elan.iloc_er1	F_const*elan.k0_er1*(elan.cred_er1*exp((1-elan.alphac_er1)*F_const*elan.eta_er1/(R_const*elan.els1.er1.minput_temperature))-elan.cox_er1*exp(-elan.alphac_er1*F_const*elan.eta_er1/(R_const*elan.els1.er1.minput_tempera	A/m²	Local current density	Boundary 2	

Name	Expression	Unit	Description	Selection	Details
	ture)))				
elan.els1.er1.iloc	elan.iloc_er1	A/m²	Local current density, Electrode Reaction 1	Boundary 2	
elan.dEeqdT_er1	0	V/K	Temperature derivative of equilibrium potential	Boundary 2	
elan.els1.er1.N0_c1	-elan.iloc_er1/F_const	mol/(m²·s)	Inward flux	Boundary 2	
elan.cred_er1	c1	mol/m³	Reduced species concentration	Boundary 2	+ operation
elan.cox_er1	c2	mol/m³	Oxidized species concentration	Boundary 2	+ operation
elan.els1.er1.N0_c2	elan.iloc_er1/F_const	mol/(m²·s)	Inward flux	Boundary 2	
elan.Qirrev_er1	elan.iloc_er1*elan.eta_er1	W/m²	Irreversible heat flux	Boundary 2	
elan.Qrev_er1	elan.iloc_er1*elan.els1.er1.minput_temperature*elan.dEeqdT_er1	W/m²	Reversible heat flux	Boundary 2	
elan.els1.er1.Qb	elan.Qrev_er1+elan.Qirrev_er1	W/m²	Electrochemical reaction boundary heat source	Boundary 2	
elan.Qbtot	elan.els1.er1.Qb	W/m²	Electrochemical reaction boundary heat source	Boundary 2	+ operation
elan.eta_er1	elan.Ect-elan.Eeq_er1	V	Overpotential	Boundary 2	

Weak Expressions

Weak expression	Integration order	Integration frame	Selection
2*elan.els1.er1.N0_c1*test(c1)*pi*R	2	Material	Boundary 2
2*elan.els1.er1.N0_c2*test(c2)*pi*R	2	Material	Boundary 2

Double Layer Capacitance 1

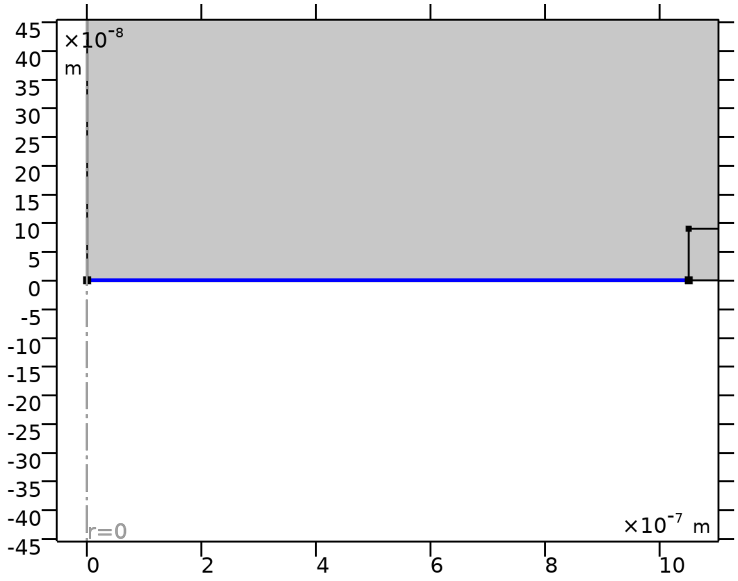

Double Layer Capacitance 1

SELECTION

Geometric entity level	Boundary
Selection	Geometry geom1: Dimension 1: All boundaries

EQUATIONS

$$i_{\mathrm{dl}} = \left(\frac{\partial(\phi_s - \phi_l)}{\partial t}\right) C_{\mathrm{dl}}$$

Double Layer Capacitance

SETTINGS

Description	Value	Unit
Electrical double layer capacitance	0.2	F/m²

Variables

Name	Expression	Unit	Description	Selection	Details
elan.itot	elan.idl	A/m²	Total interface current density	Boundary 2	+ operation
elan.Cdl	0.2[F/m^2]	F/m²	Electrical double layer capacitance	Boundary 2	
elan.idl	d(elan.Ect,t)*elan.Cdl	A/m²	Double layer current density	Boundary 2	

2.5.9 Initial Values 2

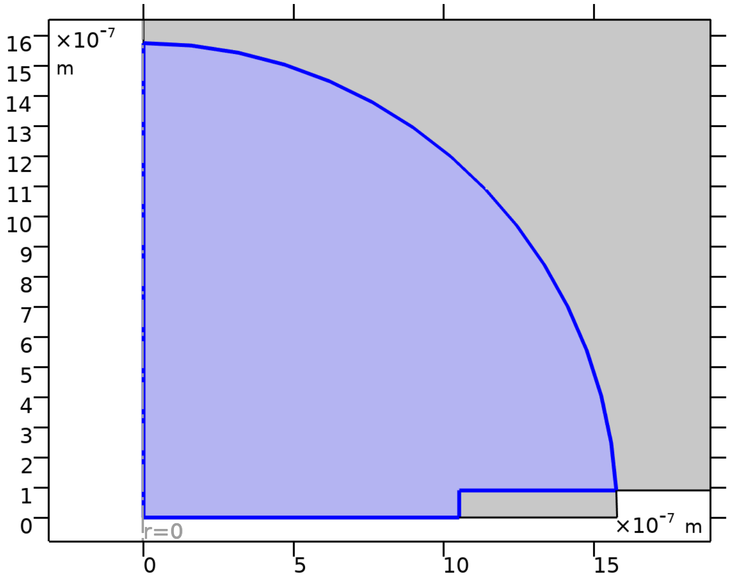

Initial Values 2

SELECTION

Geometric entity level	Domain
Selection	Geometry geom1: Dimension 2: Domain 1

Initial Values

SETTINGS

**Description**	**Value**	**Unit**
Concentration	{cRbulk, 0}	mol/m³

Variables

**Name**	**Expression**	**Unit**	**Description**	**Selection**	**Details**
elan.c0_c1	cRbulk	mol/m³	Concentration	Domain 1	+ operation
elan.c0_c2	0	mol/m³	Concentration	Domain 1	+ operation

2.6 MULTIPHYSICS

2.6.1 Flow Coupling 1

USED PRODUCTS

COMSOL Multiphysics

Coupled Interfaces

SETTINGS

Description	Value
Source	Creeping Flow (spf)
Destination	Electroanalysis (elan)

Variables

Name	Expression	Unit	Description	Selection
fc1.uR	spatial.invF11*u+spatial.invF31*w	m/s	Velocity field, R-component	Global
fc1.uPHI	0	m/s	Velocity field, PHI-component	Global
fc1.uZ	spatial.invF13*u+spatial.invF33*w	m/s	Velocity field, Z-component	Global
fc1.p	p	Pa	Pressure	Global
fc1.pA	spf.pA	Pa	Absolute pressure	Global

2.6.2 Potential Coupling 1

USED PRODUCTS

COMSOL Multiphysics

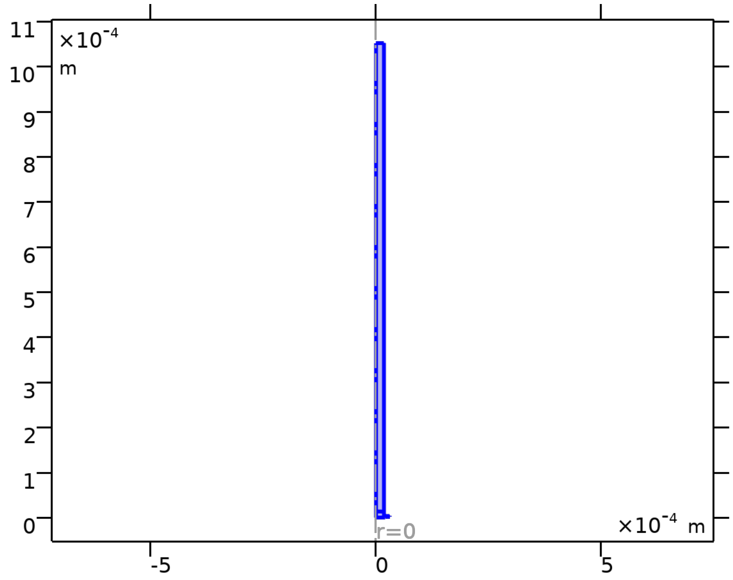

Potential Coupling 1

SELECTION

Geometric entity level	Domain
Selection	Geometry geom1: Dimension 2: Domains 1–3

Coupled Interfaces

SETTINGS

Description	Value
Source	Electrostatics (es)
Destination	Electroanalysis (elan)

Variables

Name	Expression	Unit	Description	Selection
pc1.V	V	V	Electrolyte potential	Domains 1–3

2.7 MESH 1

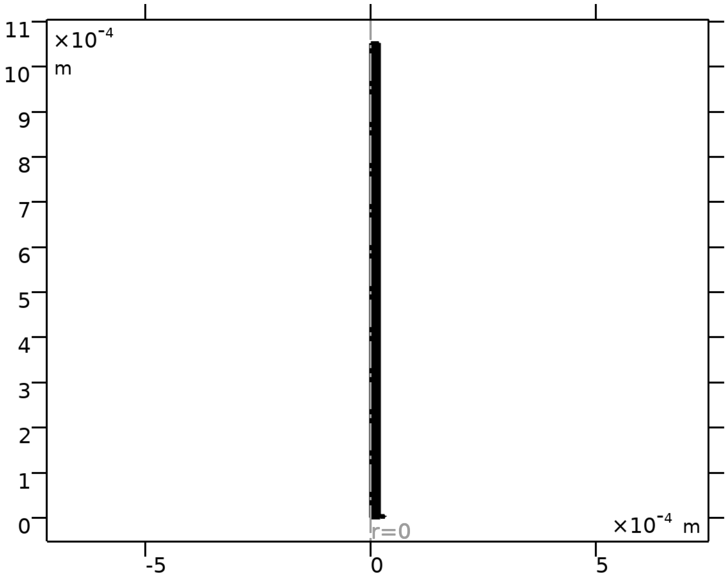

Mesh 1

2.7.1 Size (size)

SETTINGS

Description	Value
Maximum element size	2E-6
Minimum element size	1E-9
Curvature factor	0.25
Maximum element growth rate	1.11
Predefined size	Extra fine
Custom element size	Custom

2.7.2 Size 1 (size1)

SELECTION

Geometric entity level	Point
Selection	Geometry geom1: Dimension 0: Points 5–6

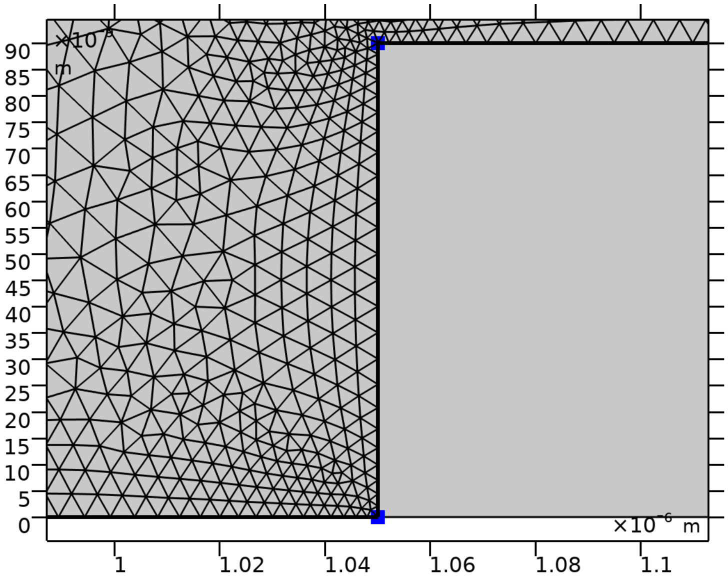

Size 1

SETTINGS

Description	Value
Maximum element size	2E-9
Minimum element size	3.06E-7
Minimum element size	Off
Curvature factor	0.3
Curvature factor	Off
Resolution of narrow regions	Off
Maximum element growth rate	1.3
Maximum element growth rate	Off
Custom element size	Custom

2.7.3 Size 2 (size2)

SELECTION

Geometric entity level	Boundary
Selection	Geometry geom1: Dimension 1: Boundaries 2, 7, 9

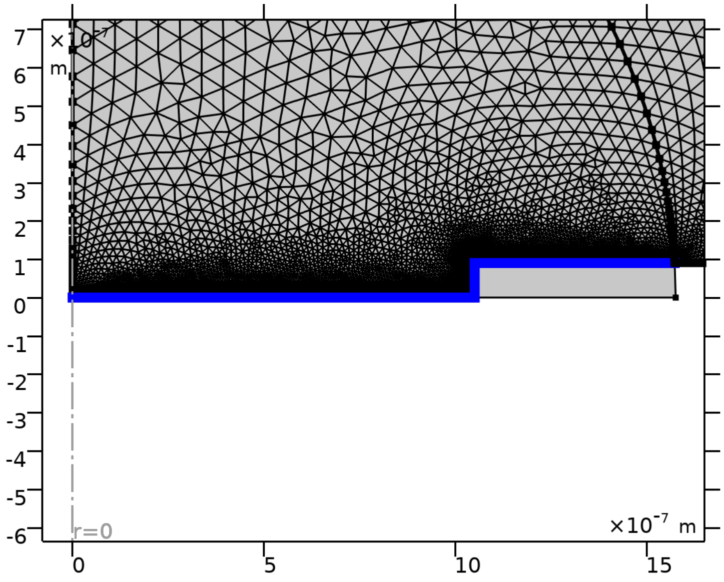

Size 2

SETTINGS

Description	Value
Maximum element size	5E-9
Minimum element size	3.06E-7
Minimum element size	Off
Curvature factor	0.3
Curvature factor	Off
Resolution of narrow regions	Off
Maximum element growth rate	1.3
Maximum element growth rate	Off
Custom element size	Custom

2.7.4 Size 3 (size3)

SELECTION

Geometric entity level	Domain
Selection	Geometry geom1: Dimension 2: Domain 1

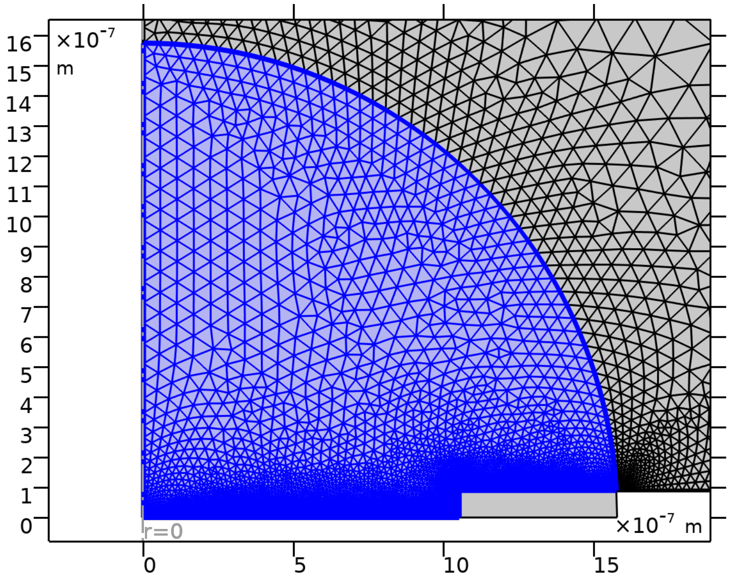

Size 3

SETTINGS

Description	Value
Maximum element size	7E-8
Minimum element size	1E-9
Curvature factor	0.25
Maximum element growth rate	1.12
Predefined size	Extra fine
Custom element size	Custom

2.7.5 Size 7 (size7)

SELECTION

Geometric entity level	Boundary
Selection	Geometry geom1: Dimension 1: Boundaries 16–17

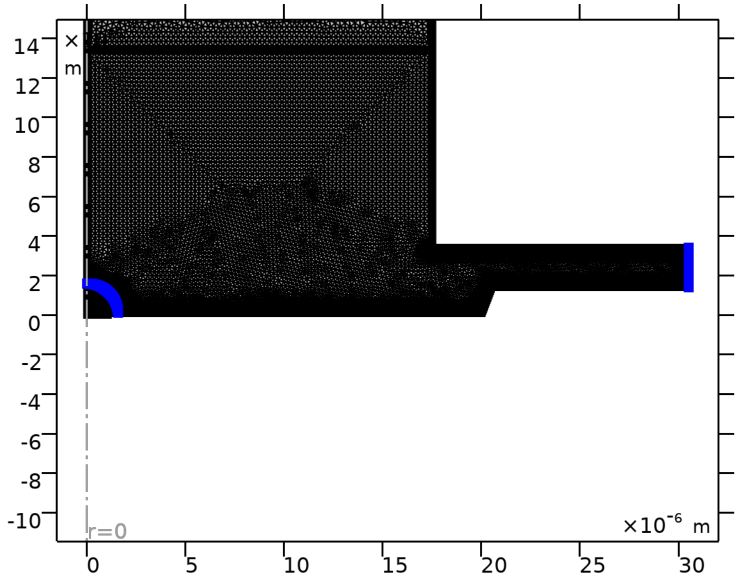

Size 7

SETTINGS

Description	Value
Maximum element size	5E-8
Minimum element size	3.15E-7
Minimum element size	Off
Curvature factor	0.3
Curvature factor	Off
Resolution of narrow regions	Off
Maximum element growth rate	1.3
Maximum element growth rate	Off
Custom element size	Custom

2.7.6 Size 4 (size4)

SELECTION

Geometric entity level	Boundary
Selection	Geometry geom1: Dimension 1: Boundaries 10, 12, 14–15

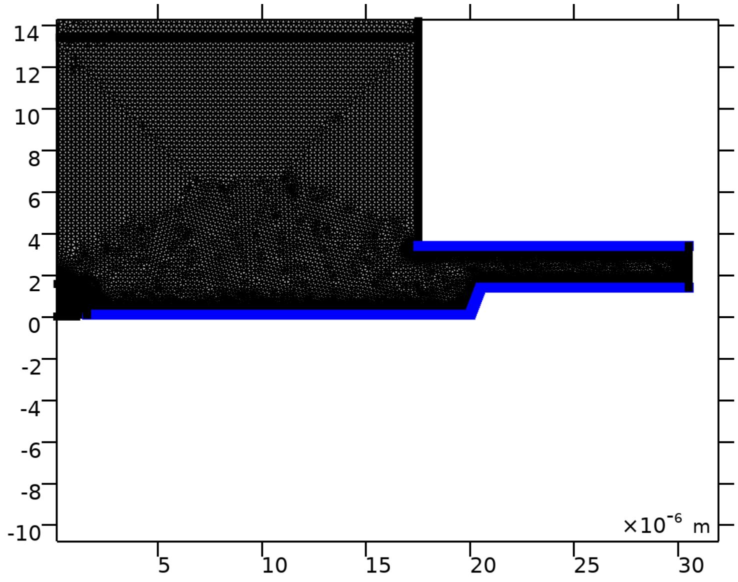

Size 4

SETTINGS

Description	Value
Maximum element size	1E-7
Minimum element size	3.06E-7
Minimum element size	Off
Curvature factor	0.3
Curvature factor	Off
Resolution of narrow regions	Off
Maximum element growth rate	1.3
Maximum element growth rate	Off
Custom element size	Custom

2.7.7 Size 5 (size5)

SELECTION

Geometric entity level	Domain
Selection	Geometry geom1: Dimension 2: Domain 2

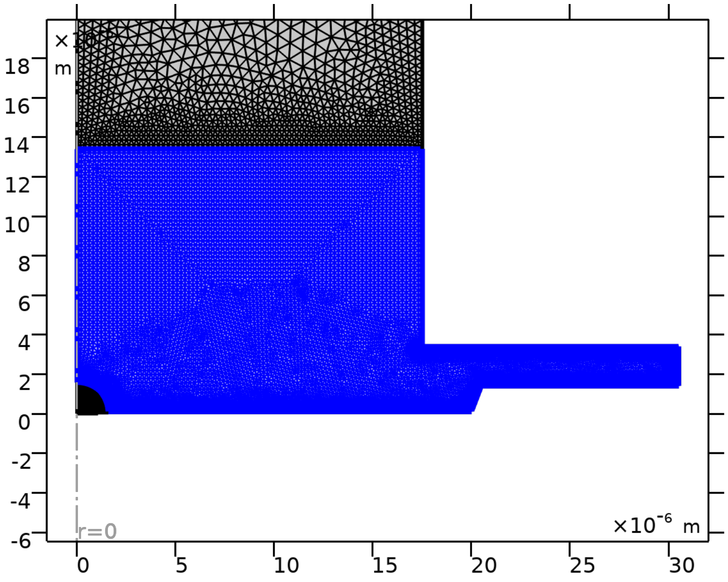

Size 5

SETTINGS

Description	Value
Maximum element size	2.5E-7
Minimum element size	3.06E-7
Minimum element size	Off
Curvature factor	0.3
Curvature factor	Off
Resolution of narrow regions	Off
Maximum element growth rate	1.15
Custom element size	Custom

2.7.8 Free Triangular 1 (ftri1)

SELECTION

Geometric entity level	Domain
Selection	Geometry geom1: Dimension 2: Domains 1–3

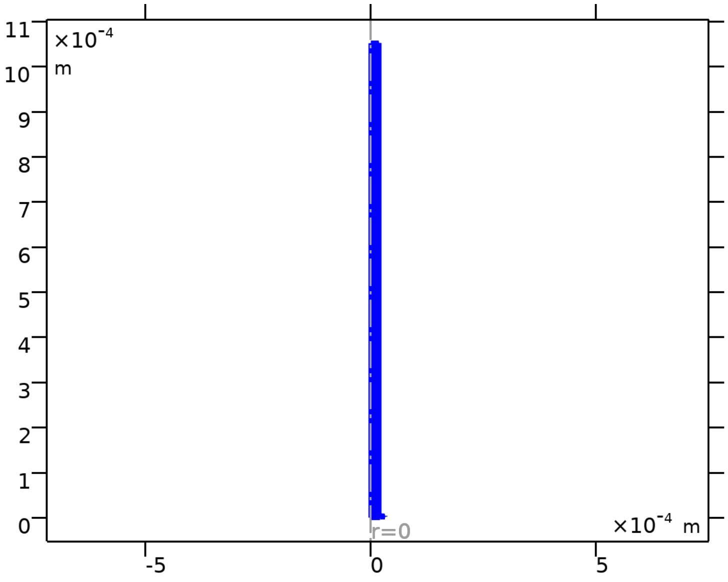

Free Triangular 1

SETTINGS

Description	Value
Last build time	0
Built with	COMSOL 6.1.0.252 (glnxa64)|2023 - 04 - 20T20:17:33.496508

3 Study 1

COMPUTATION INFORMATION

Computation time	24 min 38 s

3.1 PARAMETRIC SWEEP

Parameter name	Parameter value list	Parameter unit
v_sweep	0.025,0.1,0.5	V/s

STUDY SETTINGS

Description	Value
Sweep type	Specified combinations
Parameter name	v_sweep
Unit	V/s

PARAMETERS

Parameter name	Parameter value list	Parameter unit
v_sweep (scan rate)	0.025,0.1,0.5	V/s

3.2 CYCLIC VOLTAMMETRY

STUDY SETTINGS

Description	Value
Include geometric nonlinearity	Off

VALUES OF DEPENDENT VARIABLES

Description	Value
Settings	User controlled
Method	Solution
Study	Study 1
Settings	User controlled
Method	Solution
Study	Study 1

PHYSICS AND VARIABLES SELECTION

Physics interface	Discretization
Creeping Flow (spf)	physics
Electrostatics (es)	physics
Electroanalysis (elan)	physics

MESH SELECTION

Geometry	Mesh
Geometry 1 (geom1)	mesh1

3.3 TIME DEPENDENT

Times	Unit
range(0,0.5,t_acc)	s

STUDY SETTINGS

Description	Value
Include geometric nonlinearity	Off

STUDY SETTINGS

Description	Value
Output times	{0, 0.5, 1, 1.5, 2, 2.5, 3, 3.5, 4, 4.5, 5, 5.5, 6, 6.5, 7, 7.5, 8, 8.5, 9, 9.5, 10, 10.5, 11, 11.5, 12, 12.5, 13, 13.5, 14, 14.5, 15}

PHYSICS AND VARIABLES SELECTION

Physics interface	Discretization
Creeping Flow (spf)	physics
Electrostatics (es)	physics
Electroanalysis (elan)	physics

MESH SELECTION

Geometry	Mesh
Geometry 1 (geom1)	mesh1

3.4 SOLVER CONFIGURATIONS

3.4.1 Solution 1

Compile Equations: Cyclic Voltammetry (st1)

STUDY AND STEP

Description	Value
Use study	Study 1
Use study step	Cyclic Voltammetry

Dependent Variables 1 (v1)

GENERAL

Description	Value
Defined by study step	Cyclic Voltammetry

INITIAL VALUES OF VARIABLES SOLVED FOR

Description	Value
Method	Solution
Solution	Parametric Solutions 9 - 6um/s tacc droplet formed

RESIDUAL SCALING

Description	Value
Method	Manual

VALUES OF VARIABLES NOT SOLVED FOR

Description	Value
Method	Solution
Solution	Parametric Solutions 9 - 6um/s tacc droplet formed

INITIAL VALUE CALCULATION CONSTANTS

Constant name	Initial value source
t	0 1e4
timestep	1e-3[V]/abs(v_sweep)

Concentration (comp1.c1) (comp1_c1)

GENERAL

Description	Value
Field components	comp1.c1
Internal variables	{comp1.uflux.c1, comp1.dflux.c1}

Concentration (comp1.c2) (comp1_c2)

GENERAL

Description	Value
Field components	comp1.c2
Internal variables	{comp1.uflux.c2, comp1.dflux.c2}

Pressure (comp1.p) (comp1_p)

GENERAL

Description	Value
Field components	comp1.p

Velocity field (comp1.u) (comp1_u)

GENERAL

Description	Value
Field components	{comp1.u, comp1.w}

Description	Value
Internal variables	comp1.spf.isFluidHasBeenSolved

Electric potential (comp1.V) (comp1_V)

GENERAL

Description	Value
Field components	comp1.V

Time-Dependent Solver 1 (t1)

GENERAL

Description	Value
Defined by study step	Cyclic Voltammetry
Output times	{0, 10000}
Times to store	Steps taken by solver
Relative tolerance	0.001

ABSOLUTE TOLERANCE

Description	Value
Tolerance factor	0.05

FIELD TOLERANCE METHOD

Field	Value
Concentration (comp1.c1)	Use_global
Concentration (comp1.c2)	Use_global
Pressure (comp1.p)	Scaled
Velocity field (comp1.u)	Use_global
Electric potential (comp1.V)	Use_global

FIELD TOLERANCE FACTOR

Field	Value
Concentration (comp1.c1)	0.1
Concentration (comp1.c2)	0.1
Pressure (comp1.p)	1
Velocity field (comp1.u)	0.1
Electric potential (comp1.V)	0.1

TIME STEPPING

Description	Value
Initial step	1e-3[V]/abs(v_sweep)

Description	Value
Initial step	On
Maximum step constraint	Constant
Maximum step	min(1e100, abs((E_start - E_vertex)/v_sweep))
Maximum BDF order	2
Nonlinear controller	On
Fraction of initial step for Backward Euler	0.01
Error estimation	Exclude algebraic

Advanced (aDef)

ASSEMBLY SETTINGS

Description	Value
Reuse sparsity pattern	On

Stop Condition 1 (st1)

STOP EXPRESSIONS

Stop expression	Stop if	Active	Description
comp1.elan.stopcond	True (>=1)	On	elan (Cyclic voltammetry)

OUTPUT AT STOP

Description	Value
Add warning	Off

Fully Coupled 1 (fc1)

GENERAL

Description	Value
Linear solver	Direct 1

METHOD AND TERMINATION

Description	Value
Damping factor	0.9
Jacobian update	Once per time step
Maximum number of iterations	8
Tolerance factor	0.5
Stabilization and acceleration	Anderson acceleration
Dimension of iteration space	5
Mixing parameter	0.9

3.4.2 Parametric Solutions 9- 6um/s tacc droplet formed

t_acc=15 (2) (su11)

GENERAL

Description	Value
Solution	t_acc=15 (2)

t_acc=20 (2) (su21)

GENERAL

Description	Value
Solution	t_acc=20 (2)

t_acc=25 (2) (su31)

GENERAL

Description	Value
Solution	t_acc=25 (2)

t_acc=30 (2) (su41)

GENERAL

Description	Value
Solution	t_acc=30 (2)

3.4.3 Parametric Solutions 10

v_sweep=0.025 (su1)

GENERAL

Description	Value
Solution	v_sweep=0.025

v_sweep=0.1 (su2)

GENERAL

Description	Value
Solution	v_sweep=0.1

v_sweep=0.5 (su3)

GENERAL

Description	Value
Solution	v_sweep=0.5

4 Results

4.1 DATA SETS

4.1.1 Study 1/Solution 1

SOLUTION

Description	Value
Solution	Solution 1
Component	Component 1 (comp1)
Frame	Material (R, PHI, Z)

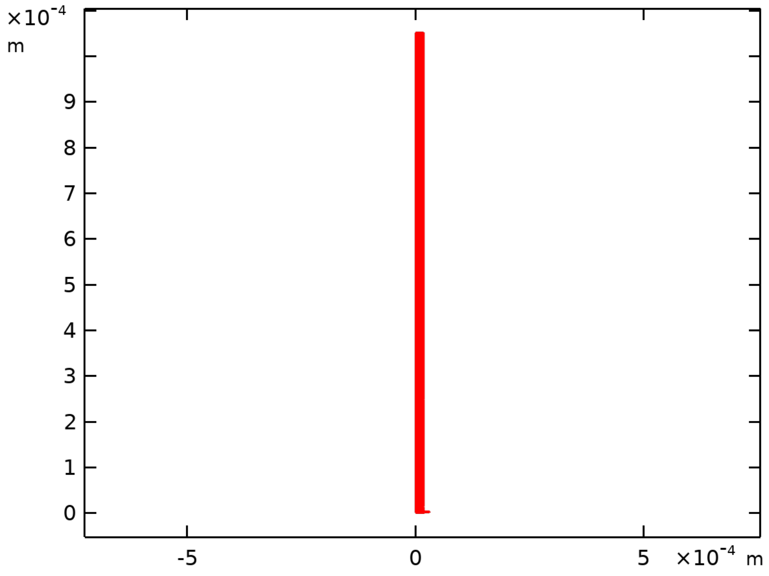

Dataset: Study 1/Solution 1

4.1.2 Revolution 2D

DATA

Description	Value
Dataset	Study 1/Solution 1

AXIS DATA

Description	Value
Axis entry method	Two points
Points	{{0, 0}, {0, 1}}

REVOLUTION LAYERS

Description	Value

Description	Value
Start angle	-90
Revolution angle	225

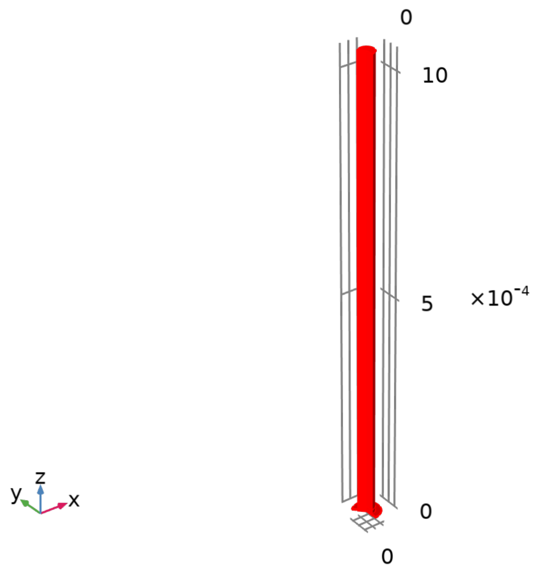

Dataset: Revolution 2D

4.1.3 Mesh 1

MESH

Description	Value
Mesh	Mesh 1

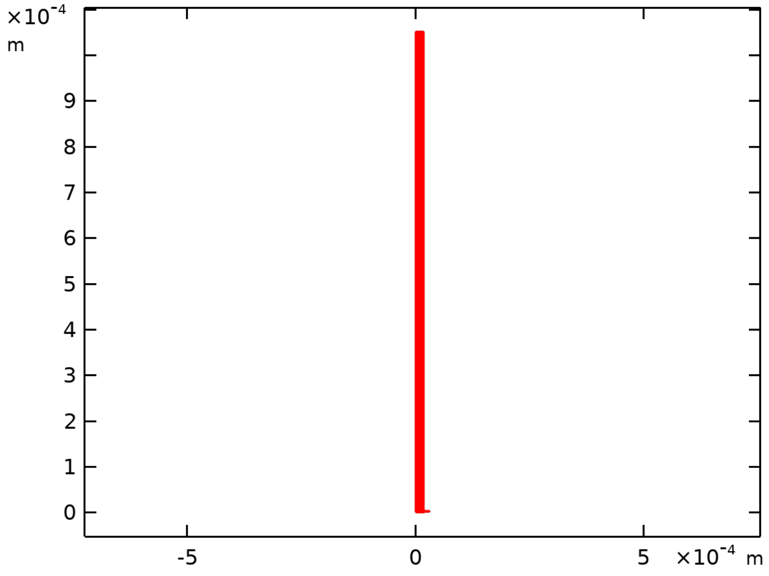

Dataset: Mesh 1

4.1.4 Revolution 2D 8

DATA

Description	Value
Dataset	Study 1/Solution 1

AXIS DATA

Description	Value
Axis entry method	Two points
Points	{{0, 0}, {0, 1}}

REVOLUTION LAYERS

Description	Value
Start angle	-90
Revolution angle	225

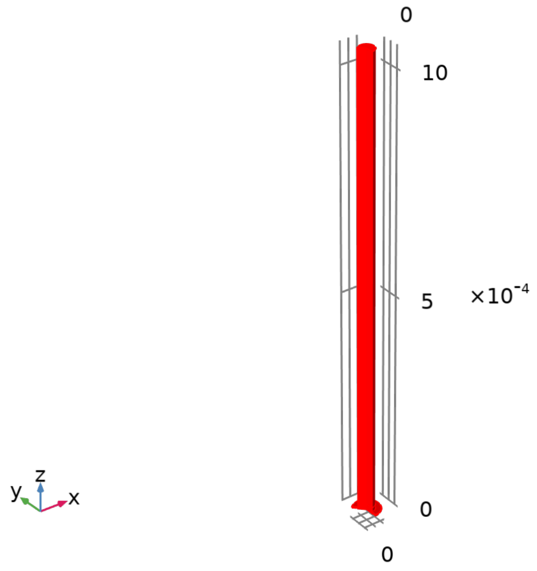

Dataset: Revolution 2D 8

4.1.5 Cut Line 2D 1

DATA

Description	Value
Dataset	Study 1/Solution 1

LINE DATA

Description	Value
Line entry method	Two points

Description	Value
Points	{{0, de+SiNx}, {rpipet, de+SiNx}}
Additional parallel lines	On
Distances	{0, 3E-6, 6E-6, 9E-6, 1.2E-5, 1.5E-5, 1.8E-5, 2.1E-5, 2.4E-5, 2.7E-5, 3E-5, 3.3E-5, 3.6E-5, 3.9E-5, 4.2E-5, 4.5E-5, 4.8E-5, 5.1E-5, 5.4E-5, 5.7E-5, 6E-5, 6.3E-5, 6.6E-5, 6.9E-5, 7.2E-5, 7.5E-5, 7.8E-5, 8.1E-5, 8.4E-5, 8.7E-5, 9E-5, 9.3E-5, 9.6E-5, 9.9E-5, 1.02E-4, 1.05E-4, 1.08E-4, 1.11E-4, 1.14E-4, 1.17E-4, 1.2E-4, 1.23E-4, 1.26E-4, 1.29E-4, 1.32E-4, 1.35E-4, 1.38E-4, 1.41E-4, 1.44E-4, 1.47E-4, 1.5E-4, 1.53E-4, 1.56E-4, 1.59E-4, 1.62E-4, 1.65E-4, 1.68E-4, 1.71E-4, 1.74E-4, 1.77E-4, 1.8E-4, 1.83E-4, 1.86E-4, 1.89E-4, 1.92E-4, 1.95E-4, 1.98E-4}

ADVANCED

Description	Value
Space variable	cln1x
Normal variables	{cln1nx, cln1ny}
Tangent variables	{cln1tx, cln1ty}

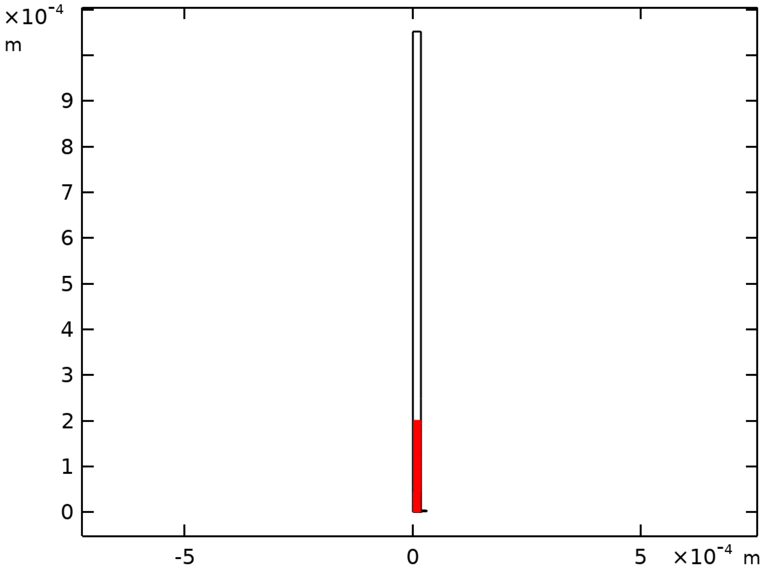

Dataset: Cut Line 2D 1

4.1.6 Study 1/Parametric Solutions 9- 6um/s tacc droplet formed

SOLUTION

Description	Value
Solution	Parametric Solutions 9 - 6um/s tacc droplet formed
Component	Component 1 (comp1)

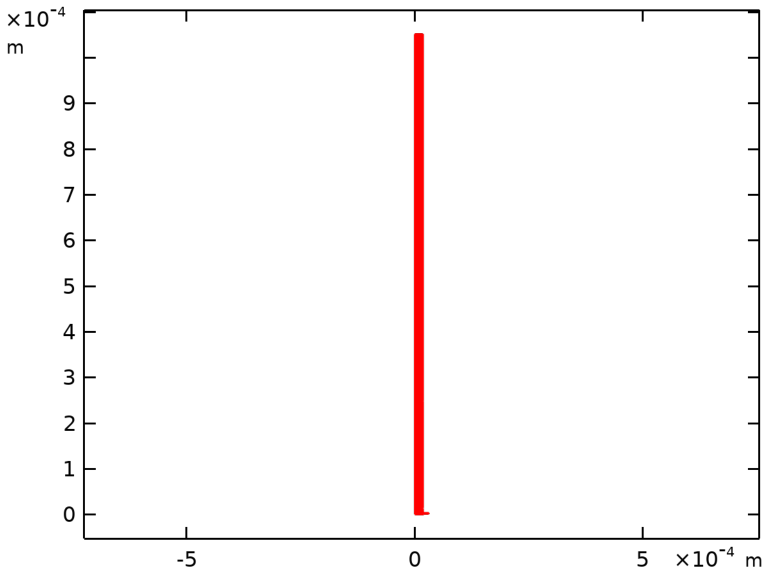

Dataset: Study 1/Parametric Solutions 9- 6um/s tacc droplet formed

4.1.7 Study 1/Solution 1 - Copy 2 8um/s 2 CV cycles after tacc 15 s

SOLUTION

Description	Value
Solution	Solution 1 - Copy 2 8um/s 2 CV cycles after tacc 15 s
Component	Component 1 (comp1)

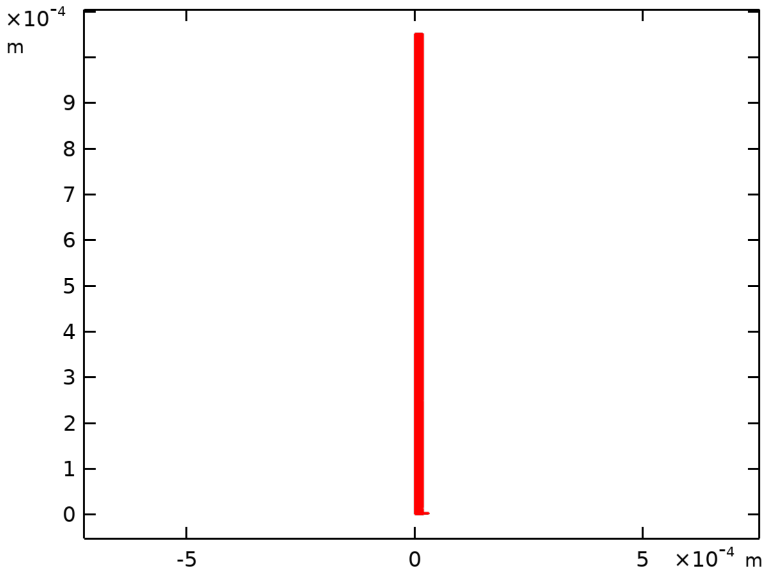

Dataset: Study 1/Solution 1 - Copy 2 8um/s 2 CV cycles after tacc 15 s

4.1.8 Study 1/Parametric Solutions 10

SOLUTION

Description	Value
Solution	Parametric Solutions 10
Component	Component 1 (comp1)

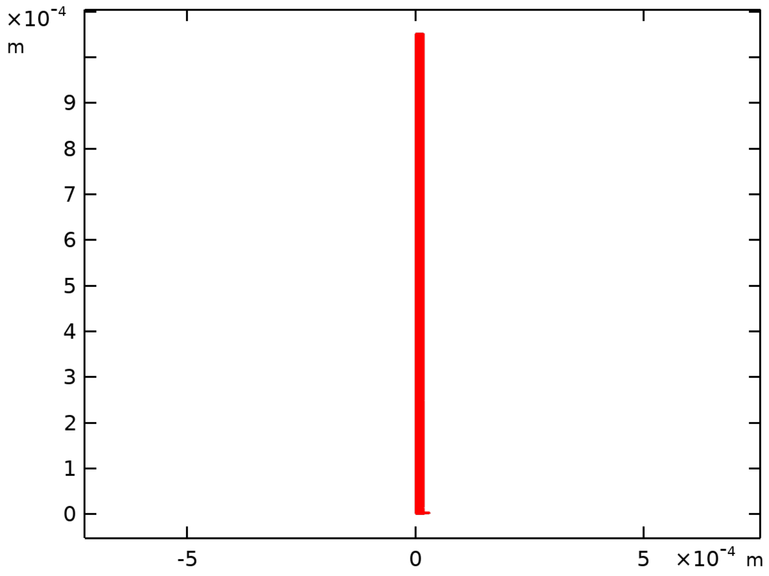

Dataset: Study 1/Parametric Solutions 10

4.1.9 Revolution 2D 9

DATA

Description	Value
Dataset	Study 1/Parametric Solutions 10

AXIS DATA

Description	Value
Axis entry method	Two points
Points	{{0, 0}, {0, 1}}

REVOLUTION LAYERS

Description	Value
Start angle	-90
Revolution angle	225

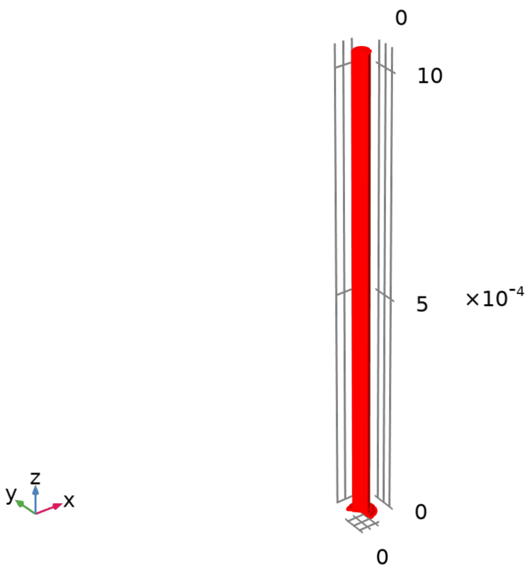

Dataset: Revolution 2D 9

4.2 DERIVED VALUES

4.2.1 Global Evaluation 1

DATA

Description	Value
Dataset	Study 1/Solution 1

4.3 TABLES

4.3.1 Evaluation 2D

Interactive 2D values

x	y	Value
2.1029E-5	2.2471E-6	1.6849E-5
2.0713E-5	2.3735E-6	1.7019E-5
2.0649E-5	2.3103E-6	1.7101E-5
2.0713E-5	2.1838E-6	1.6774E-5
2.0523E-5	2.3735E-6	1.6907E-5
2.0649E-5	2.3735E-6	1.6982E-5
1.144E-6	1.3433E-5	5.3697E-6
3.9808E-6	3.0881E-7	2.4604
2.9956E-5	2.6027E-6	2.6698
3.0496E-5	1.9955E-6	2.676

x	y	Value
3.6434E-6	8.4855E-7	2.46
3.0362E-6	1.1184E-6	2.6943
2.9013E-6	6.4615E-7	2.6944
2.9013E-6	1.7387E-7	2.6944
6.0738E-7	1.7387E-7	2.6935
2.9888E-5	2.4003E-6	2.9248
3.0158E-5	2.4003E-6	2.9282
3.0428E-5	2.4003E-6	2.9317
6.7485E-7	1.7387E-7	2.6936
7.0843E-6	7.1362E-7	2.8921
6.8144E-6	6.4615E-7	2.8916
4.0482E-6	5.7868E-7	2.8878
2.9754E-5	2.2654E-6	3.1323
3.0361E-5	2.4678E-6	3.1406
1.8218E-6	3.7628E-7	2.8863
3.0362E-6	3.0881E-7	2.887
8.0979E-7	1.7387E-7	2.886
2.9013E-6	7.8108E-7	2.8868
3.0361E-5	2.5353E-6	3.1406
2.0917E-6	3.0881E-7	2.8864
6.0738E-7	1.064E-7	2.8859
3.0226E-5	2.6702E-6	3.1388
6.7639E-8	2.4134E-7	2.8859
3.0023E-5	2.063E-6	3.136
1.6058E-5	1.4558E-6	2.9296
7.4232E-7	9.8349E-7	2.8858
6.151E-7	5.7868E-7	3.1952
6.151E-7	5.7868E-7	3.1952
1.7617E-5	9.1602E-7	3.3196
1.8224E-5	7.1362E-7	3.3329
3.3138E-6	7.8108E-7	3.1977
2.5042E-6	5.7868E-7	3.1966
6.8257E-7	3.0881E-7	3.1953
5.9451E-6	1.2534E-6	3.2034
9.9257E-6	1.2534E-6	3.2214

x	y	Value
1.3771E-5	1.3208E-6	3.2539
1.8157E-5	6.4615E-7	3.3314
1.9506E-5	1.7387E-7	3.3556
2.9829E-5	2.9401E-6	3.7266
3.0301E-5	2.3328E-6	3.741
1.5593E-5	9.1602E-7	3.2805
1.0874E-6	5.7868E-7	3.1954
6.8257E-7	2.4134E-7	3.1953
6.8257E-7	2.4134E-7	3.1953
6.8257E-7	2.4134E-7	3.1953
2.3018E-6	3.7628E-7	3.1965
4.127E-7	1.064E-7	3.1953

4.4 PLOT GROUPS

4.4.1 Concentration, c1 (tcd)

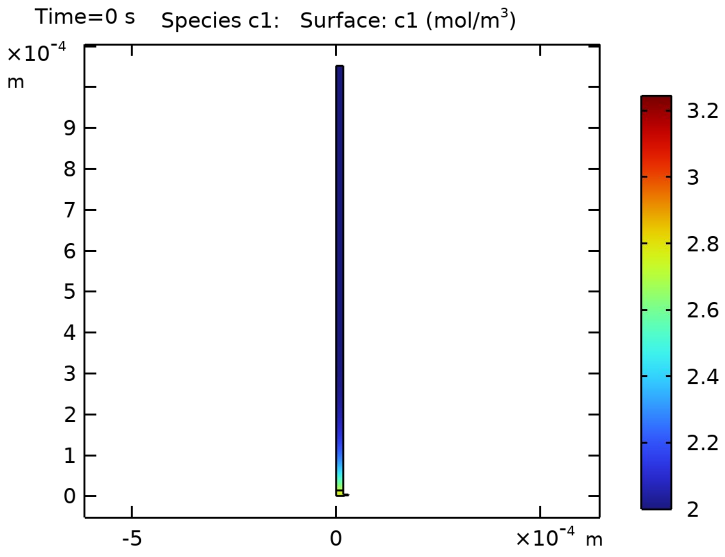

Species c1: Surface: c1 (mol/m^3)

4.4.2 Concentration, c1, 3D (tcd)

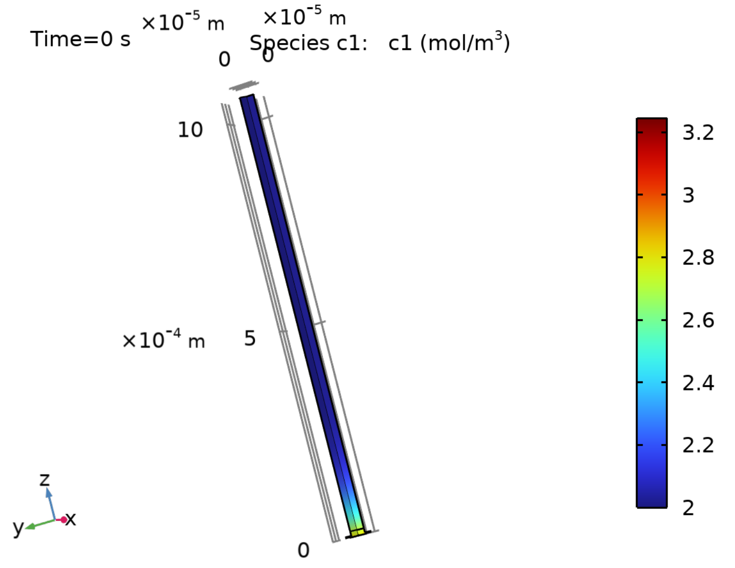

Species c1: c1 (mol/m³)

4.4.3 Concentration, c2 (tcd)

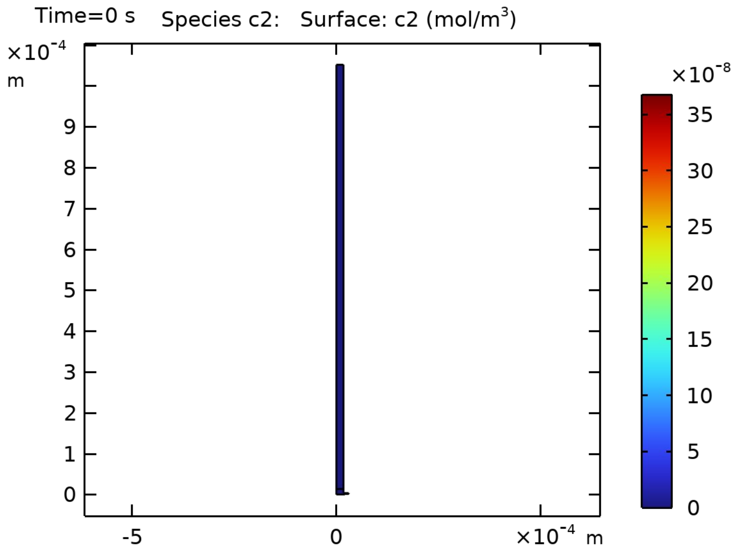

Species c2: Surface: c2 (mol/m³) Streamline:

4.4.4 Concentration, c2, 3D (tcd)

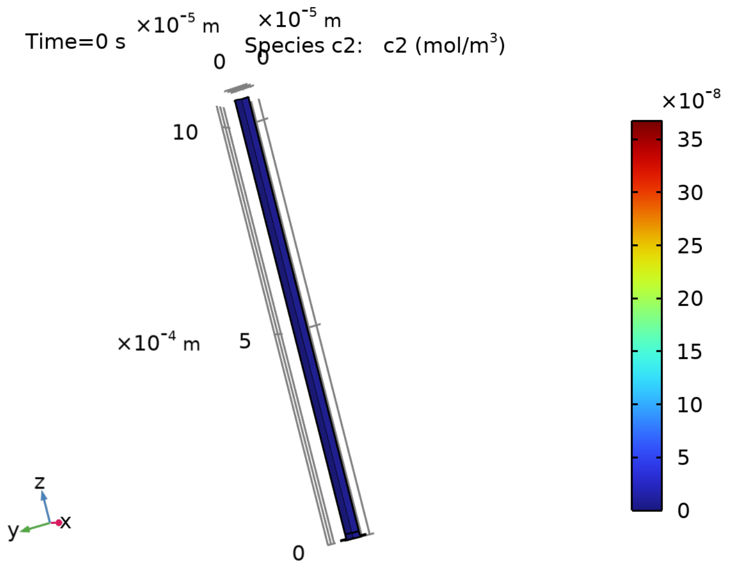

Species c2: c2 (mol/m³)

4.4.5 Cyclic Voltammograms (elan)

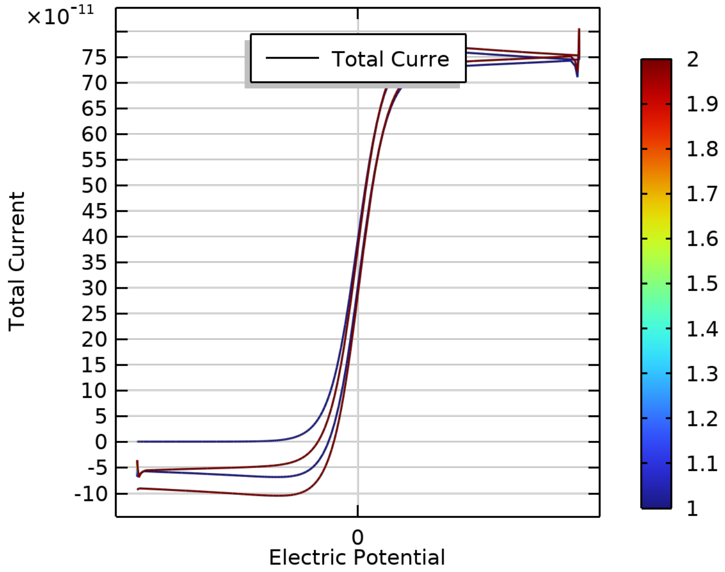

4.4.6 Concentration (elan)

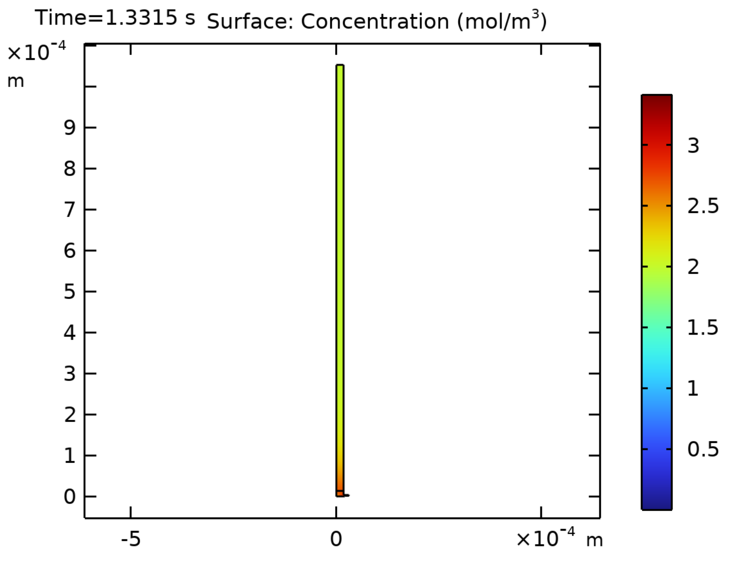

Surface: Concentration (mol/m³)

4.4.7 Velocity (spf) 2

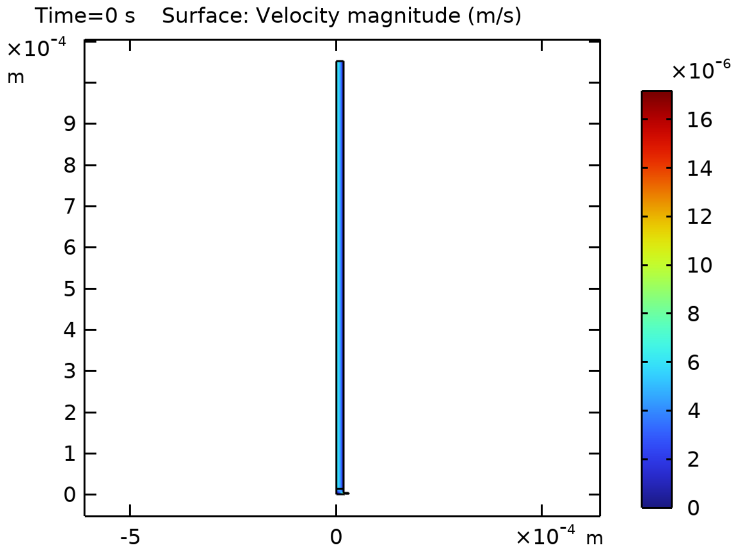

Surface: Velocity magnitude (m/s)

4.4.8 Electrode Potential (elan)

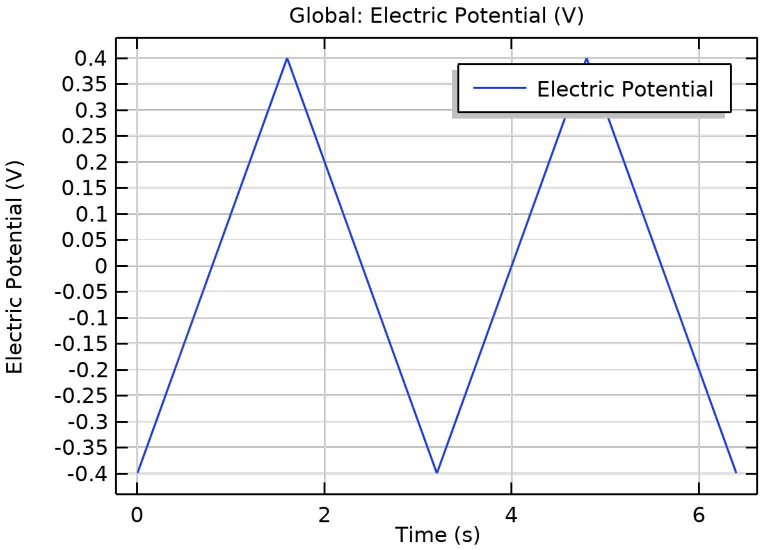

Global: Electric Potential (V)
